# Supplementary material for: Targeting myoferlin in ER/Golgi vesicle trafficking reprograms pancreatic cancer-associated fibroblasts
Source: EMBO J. 2025 Oct 8;44(22):6425–65. doi: 10.1038/s44318-025-00570-6 (PMC12623807; doi:10.1038/s44318-025-00570-6)
Supplement: Supplementary file 1 — Appendix [file 44318_2025_570_MOESM1_ESM.pdf]

Table of contents

Appendix Table S1 - List of qPCR primer pairs (p. 2)

Appendix Table S1. List of qPCR primer pairs

Target	Forward primer	Reverse primer
18S	CTTCCACAGGAGGCCTACAC	CGCAAAATATGCTGGAACTTT
ACTA2	CTATGCCTCTGGACGCACAACT	CAGATCCAGACGCATGATGGCA
AREG	ACACCTACTCTGGGAAGCGT	GCCAGGTATTTGTGGTTCGT
CCN2	CCTGCAGGCTAGAGAAGCAG	TGGAGATTTTGGGAGTACGG
COL1A1	GATTCCCTGGACCTAAAGGTGC	AGCCTCTCCATCTTTGCCAGCA
COL1A2	CCTGGTGCTAAAGGAGAAAGAGG	ATCACCACGACTTCCAGCAGGA
COL2A1	CCTGGCAAAGATGGTGAGACAG	CCTGGTTTTCCACCTTCACCTG
COL3A1	TGGTCTGCAAGGAATGCCTGGA	TCTTTCCCTGGGACACCATCAG
COL6A3	TCTTTTGCCTCTTTCTCTCAGG	CTCTTGTTGACTTCAATGACT
FN1	CAAACAGAAATGACTATTGAAGGC	TGAGTGAACTTCAGGTCAGTTGGT
GLI2	ACGAGGGTCATCTGGTGGT	AGGGAGAGGGGACTGTTTGG
MYOF	TTCTCATCTTCGGGAACTGG	CGTTGGAACAAAGCCTACCT
PAI1	CTCATCAGCCACTGGAAAGGCA	GACTCGTGAAGTCAGCCTGAAAC
PLIN1	GCGGAATTTGCTGCCAACACTC	AGACTTCTGGGCTTGCTGGTGT
PLIN2	GATGGCAGAGAACGGTGTGAAG	CAGGCATAGGTATTGGCAACTGC
TGFBI	GGACATGCTCACTATCAACGGG	CTGTGGACACATCAGACTCTGC
