# Supplementary figures and images for: Targeting myoferlin in ER/Golgi vesicle trafficking reprograms pancreatic cancer-associated fibroblasts (part 1 of 2)
Source: EMBO J. 2025 Oct 8;44(22):6425–65. doi: 10.1038/s44318-025-00570-6 (PMC12623807; doi:10.1038/s44318-025-00570-6)

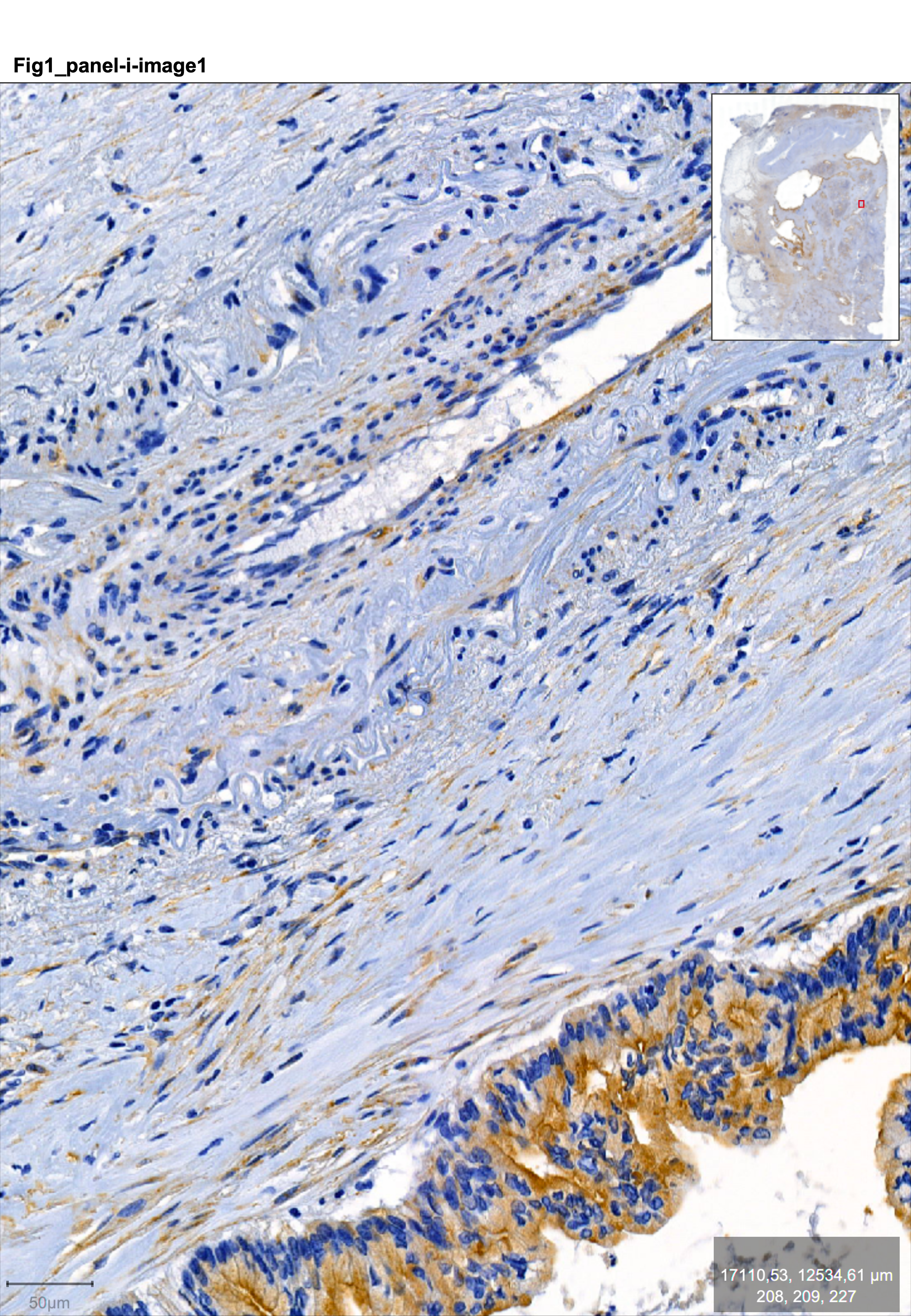

Supplement: Supplementary file 3 — Source data Fig. 1 [file 44318_2025_570_MOESM3_ESM.zip › Fig1/Images/I/Fig_1_panel_i_image_1.tiff]

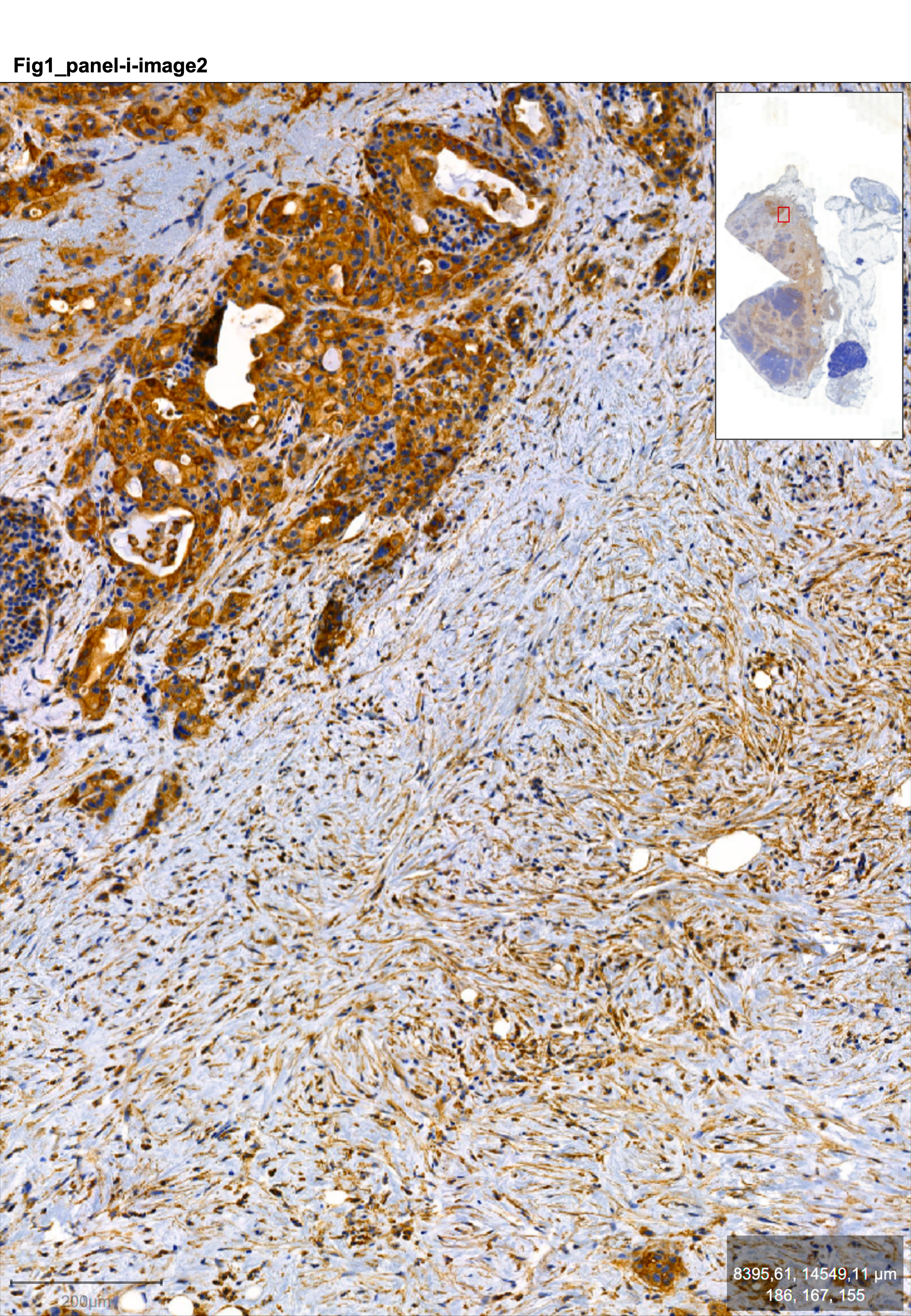

Supplement: Supplementary file 3 — Source data Fig. 1 [file 44318_2025_570_MOESM3_ESM.zip › Fig1/Images/I/Fig_1_panel_i_image_2.tiff]

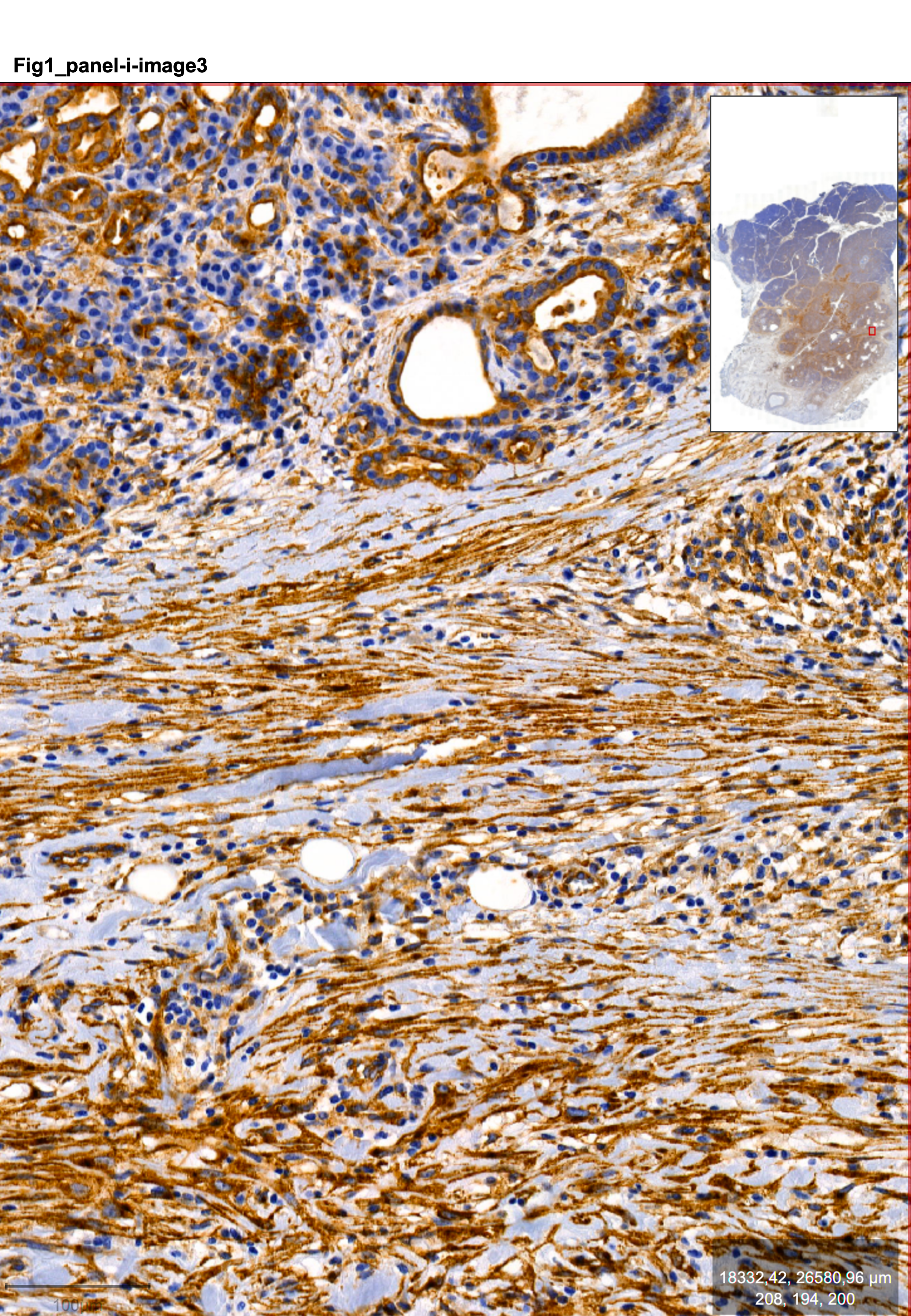

Supplement: Supplementary file 3 — Source data Fig. 1 [file 44318_2025_570_MOESM3_ESM.zip › Fig1/Images/I/Fig_1_panel_i_image_3.tiff]

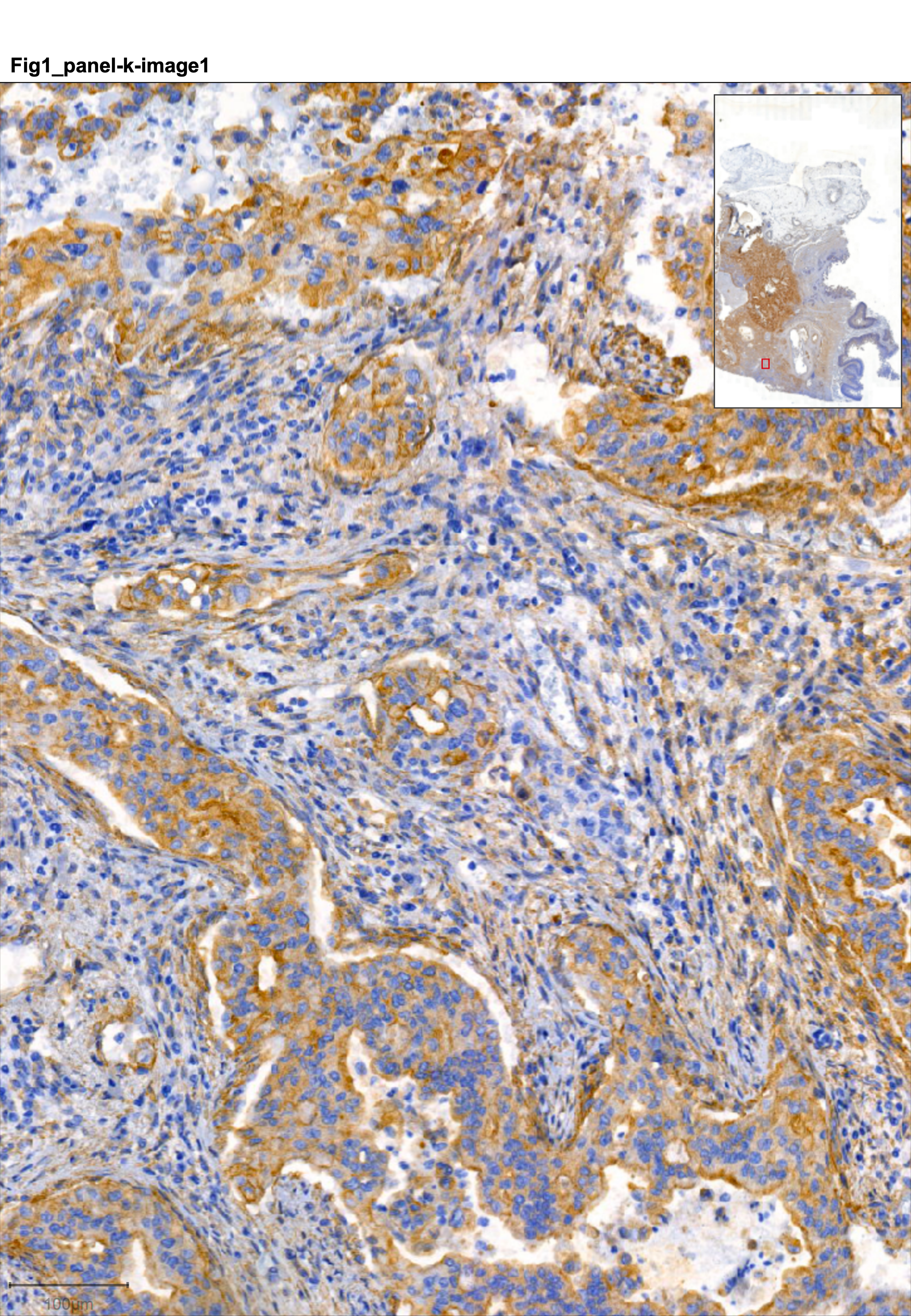

Supplement: Supplementary file 3 — Source data Fig. 1 [file 44318_2025_570_MOESM3_ESM.zip › Fig1/Images/K/Fig_1_panel_k_image_1.tiff]

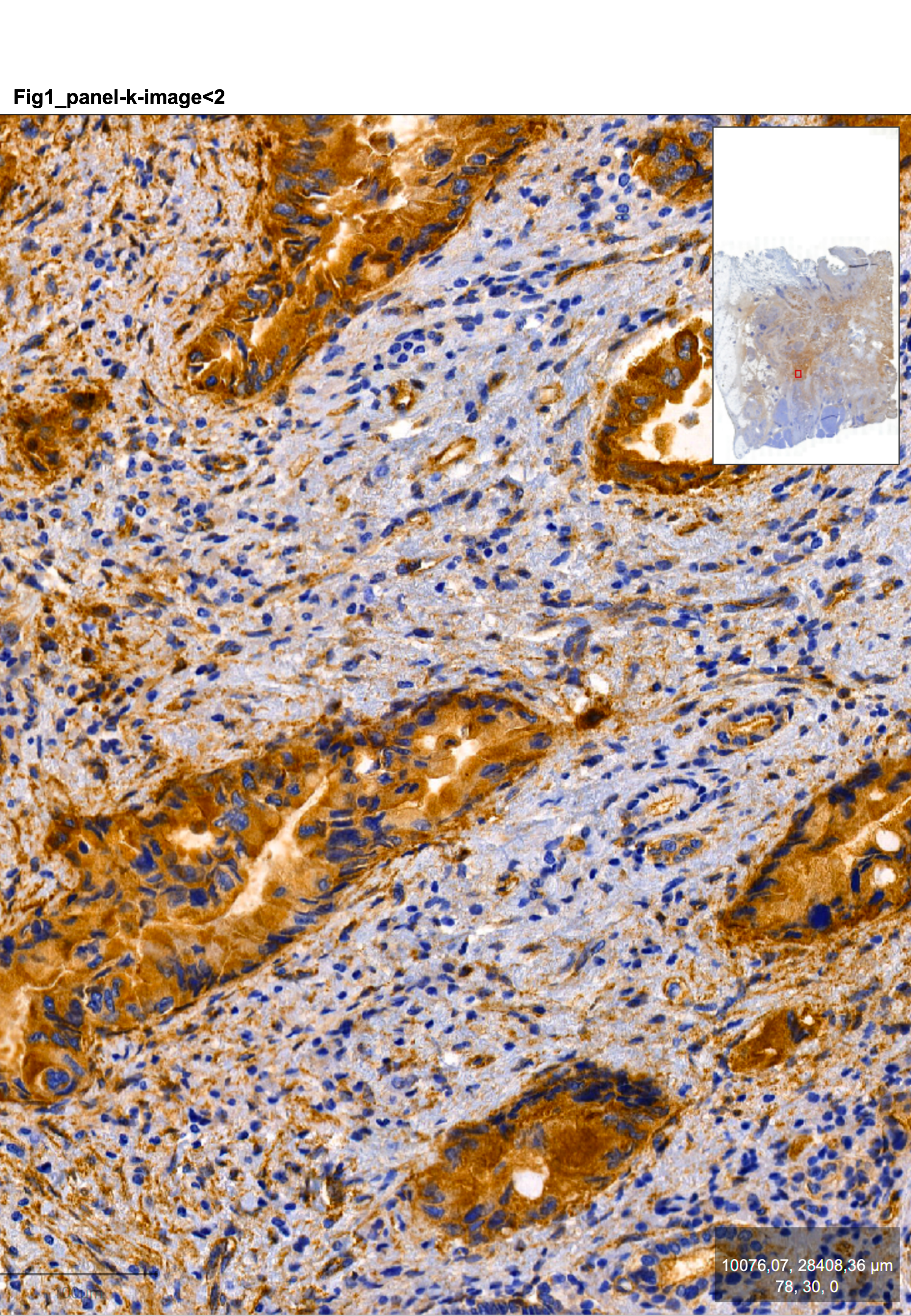

Supplement: Supplementary file 3 — Source data Fig. 1 [file 44318_2025_570_MOESM3_ESM.zip › Fig1/Images/K/Fig_1_panel_k_image_2.tiff]

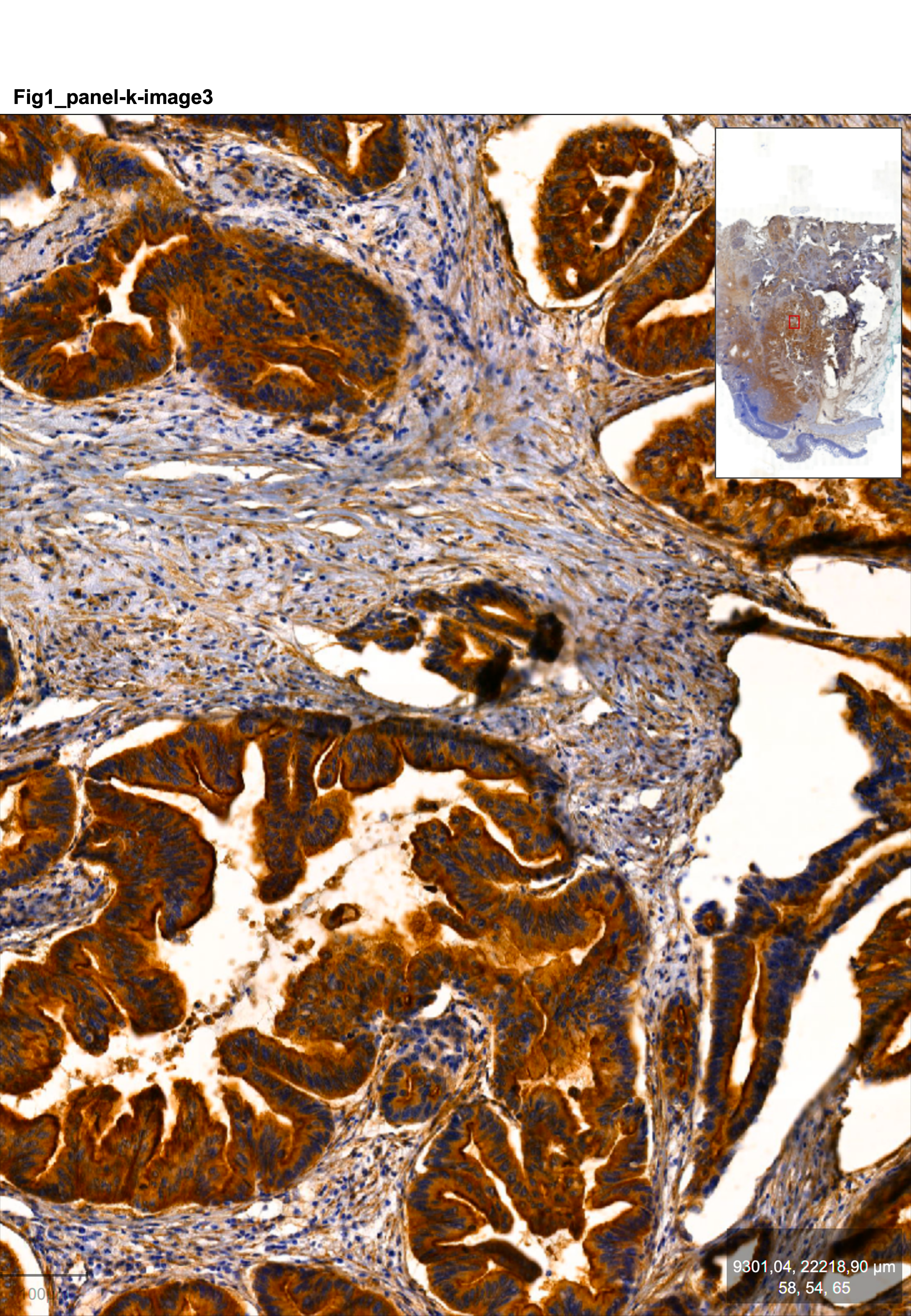

Supplement: Supplementary file 3 — Source data Fig. 1 [file 44318_2025_570_MOESM3_ESM.zip › Fig1/Images/K/Fig_1_panel_k_image_3.tiff]

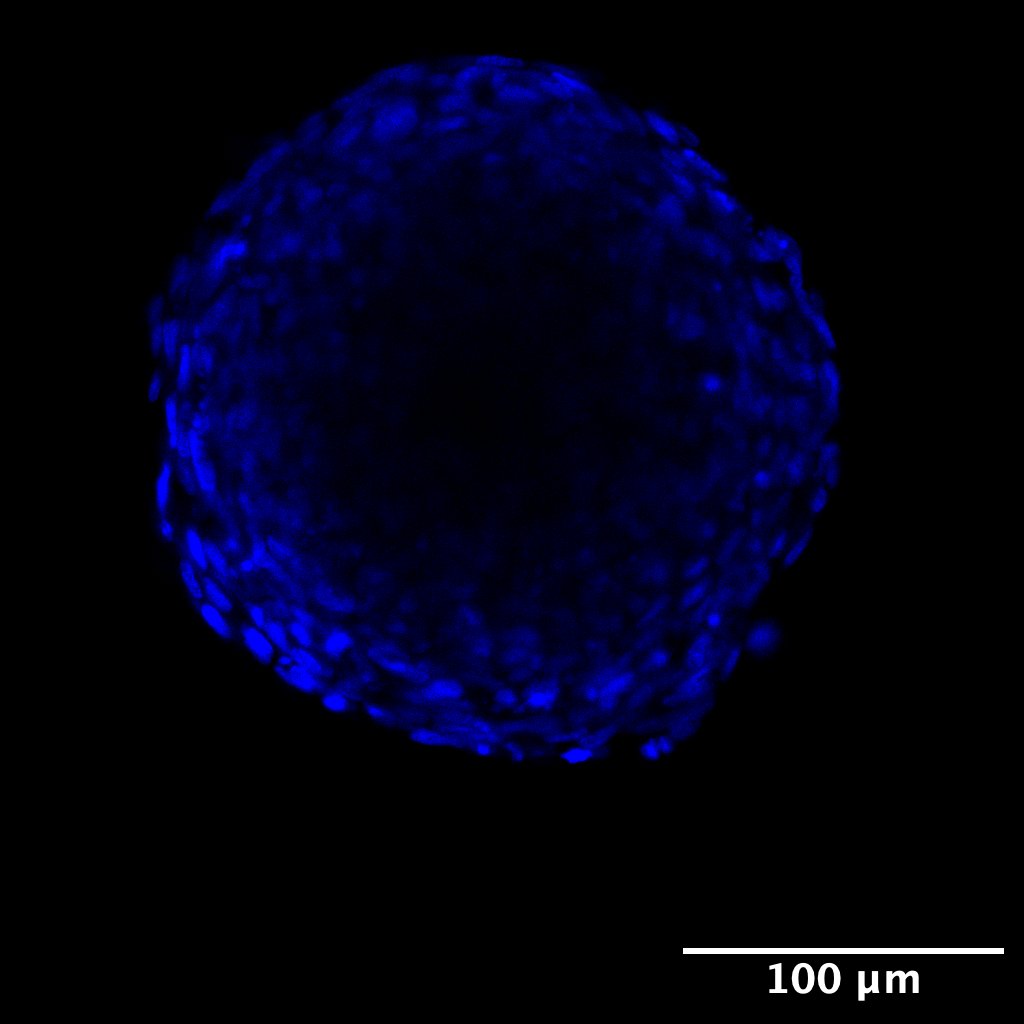

Supplement: Supplementary file 5 — Source data Fig. 3 [file 44318_2025_570_MOESM5_ESM.zip › Fig3/Images/H/Fig_3_panel_h_RP234_sh#1_6_blue.jpg]

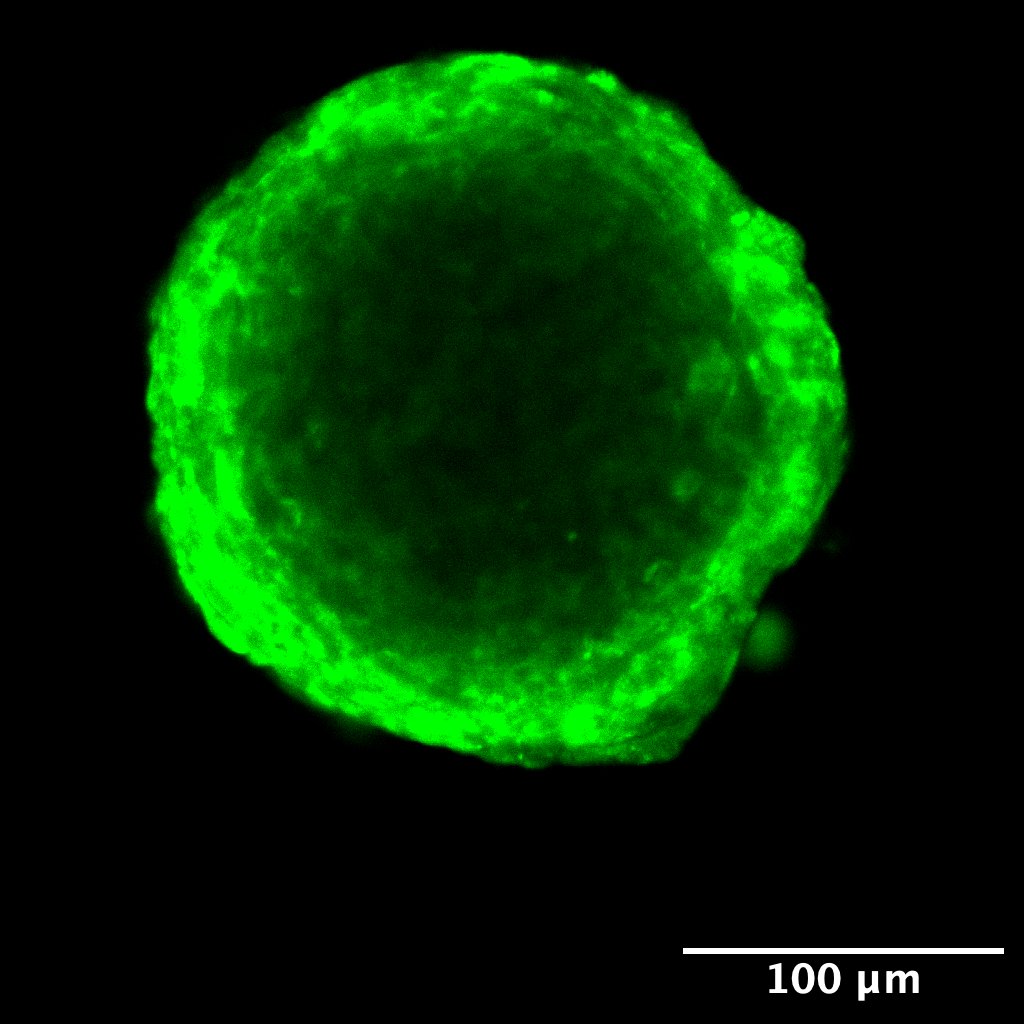

Supplement: Supplementary file 5 — Source data Fig. 3 [file 44318_2025_570_MOESM5_ESM.zip › Fig3/Images/H/Fig_3_panel_h_RP234_sh#1_6_green.jpg]

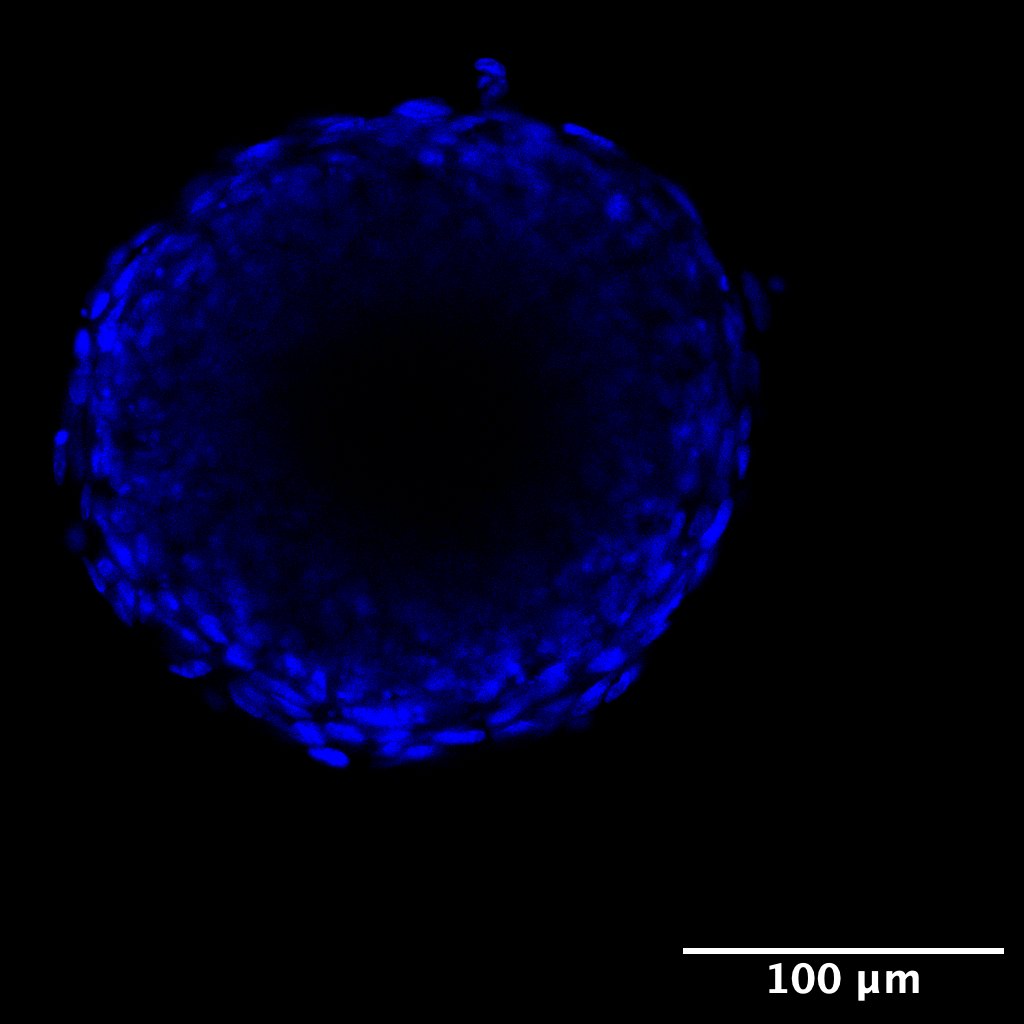

Supplement: Supplementary file 5 — Source data Fig. 3 [file 44318_2025_570_MOESM5_ESM.zip › Fig3/Images/H/Fig_3_panel_h_RP234_sh#5_9_blue.jpg]

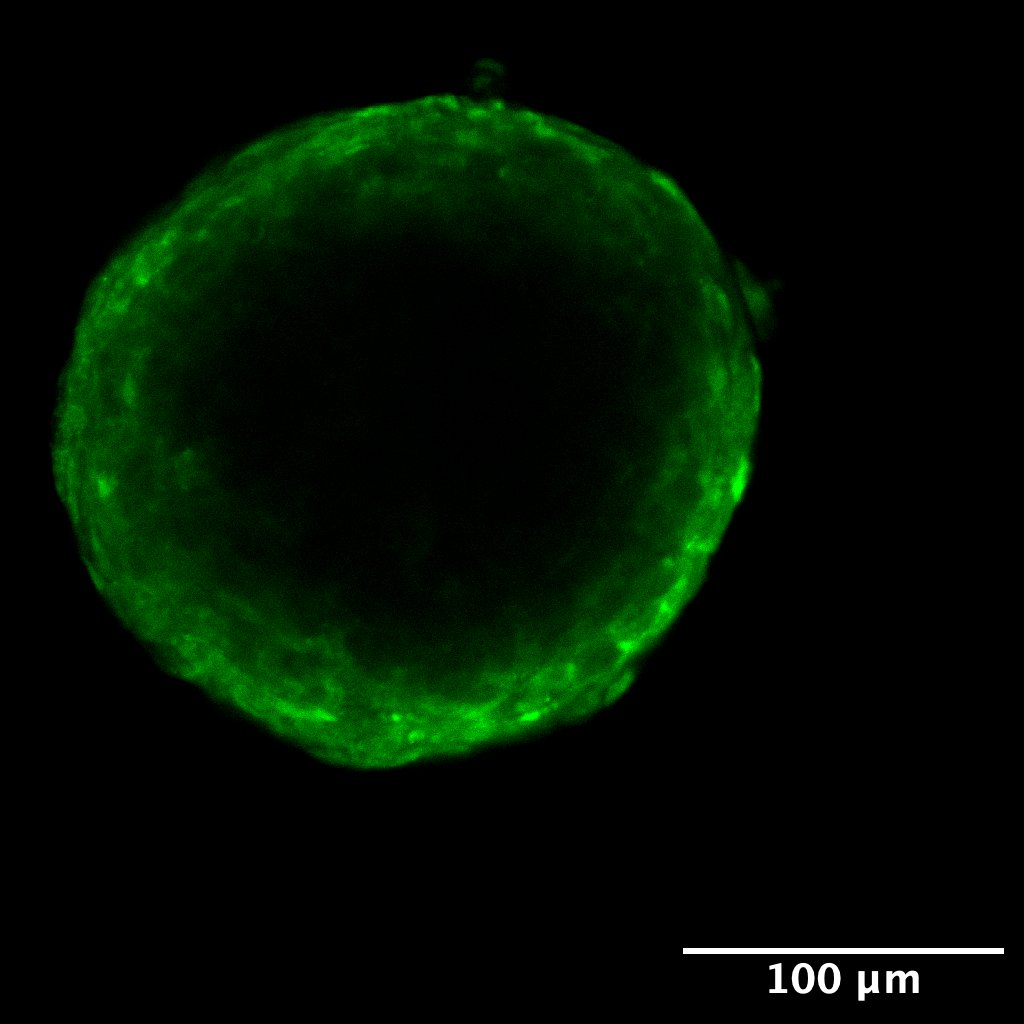

Supplement: Supplementary file 5 — Source data Fig. 3 [file 44318_2025_570_MOESM5_ESM.zip › Fig3/Images/H/Fig_3_panel_h_RP234_sh#5_9_green.jpg]

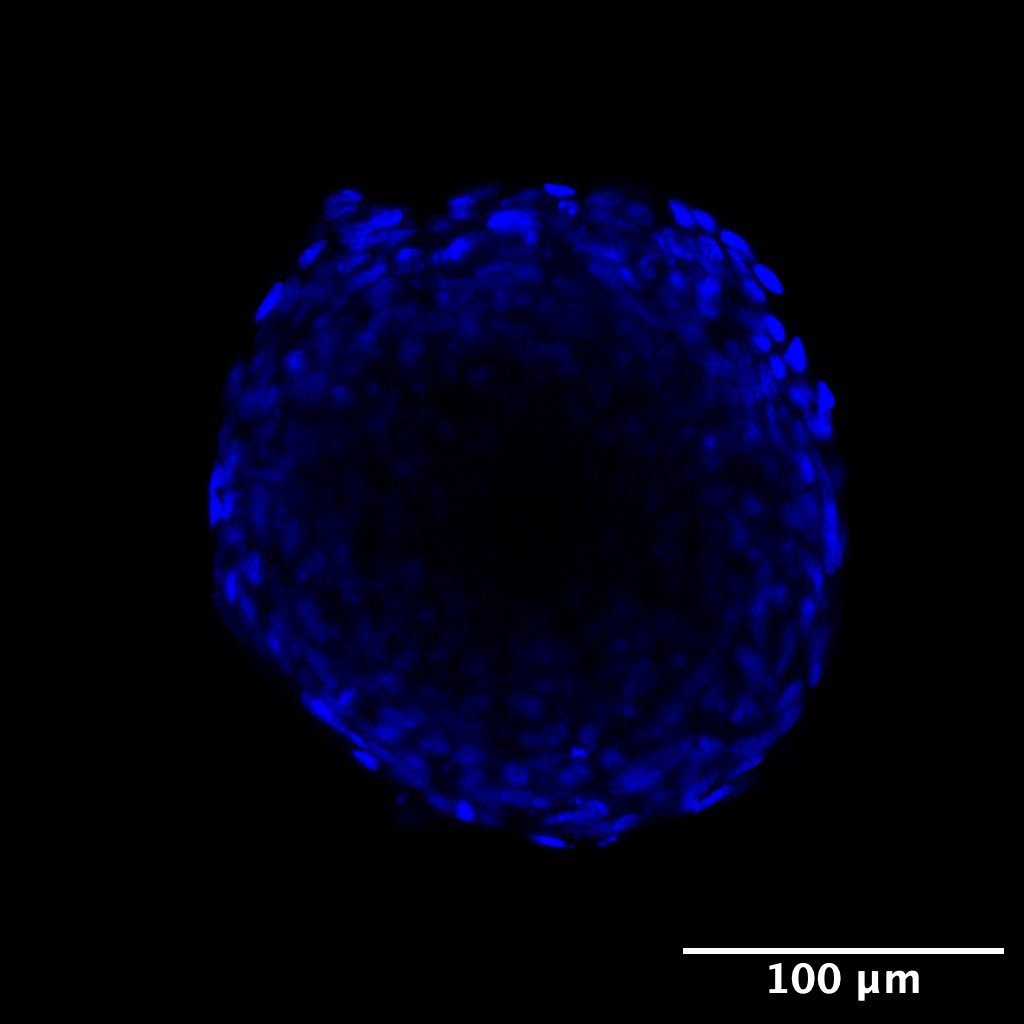

Supplement: Supplementary file 5 — Source data Fig. 3 [file 44318_2025_570_MOESM5_ESM.zip › Fig3/Images/H/Fig_3_panel_h_RP234_shNT_3_blue.jpg]

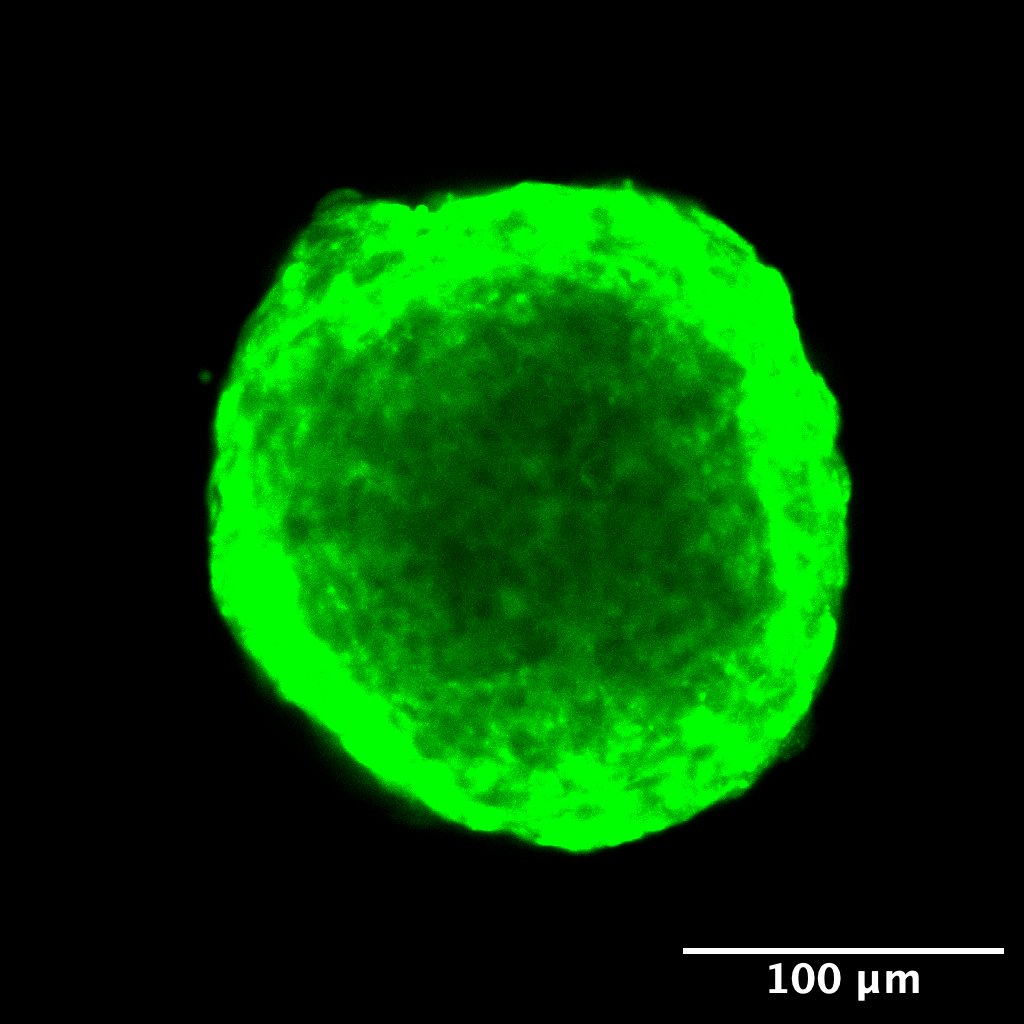

Supplement: Supplementary file 5 — Source data Fig. 3 [file 44318_2025_570_MOESM5_ESM.zip › Fig3/Images/H/Fig_3_panel_h_RP234_shNT_3_green.jpg]

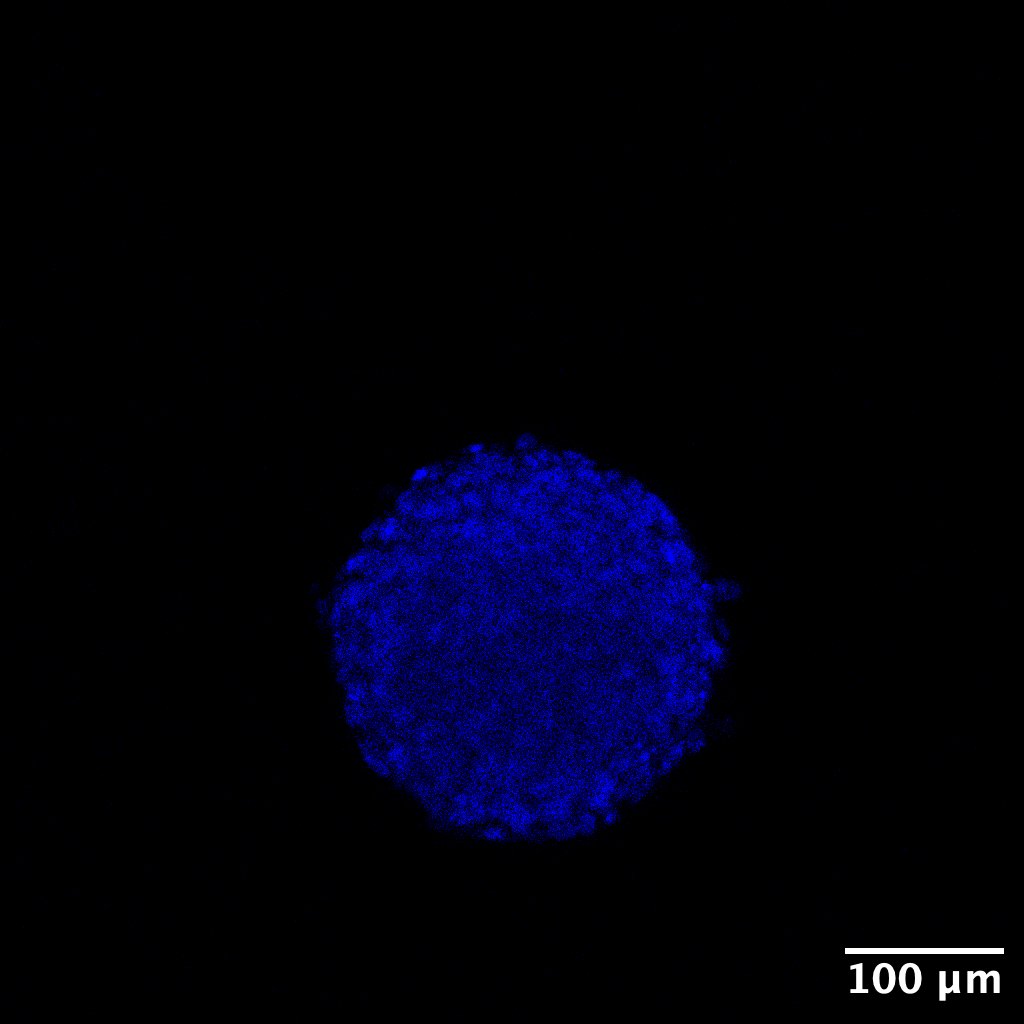

Supplement: Supplementary file 5 — Source data Fig. 3 [file 44318_2025_570_MOESM5_ESM.zip › Fig3/Images/H/Fig_3_panel_h_RP254_sh#1_5_blue.jpg]

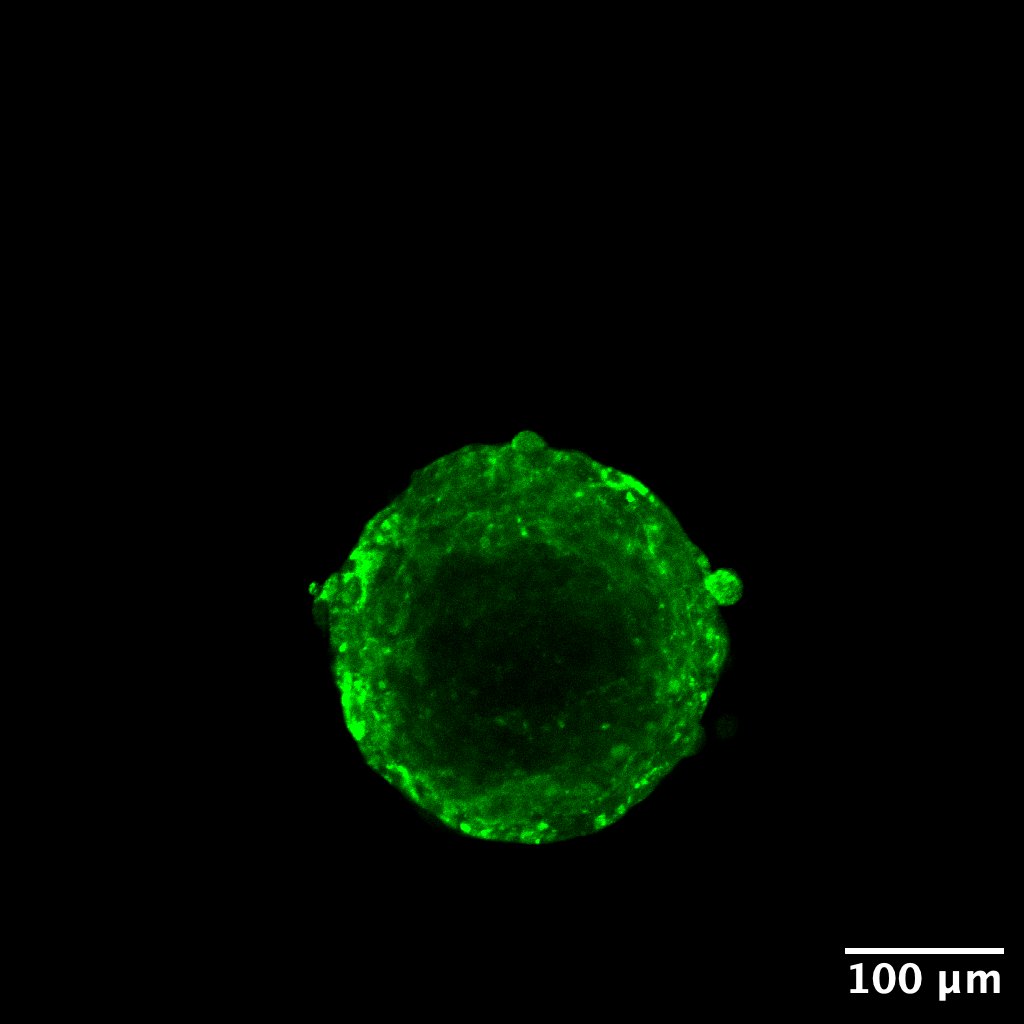

Supplement: Supplementary file 5 — Source data Fig. 3 [file 44318_2025_570_MOESM5_ESM.zip › Fig3/Images/H/Fig_3_panel_h_RP254_sh#1_5_green.jpg]

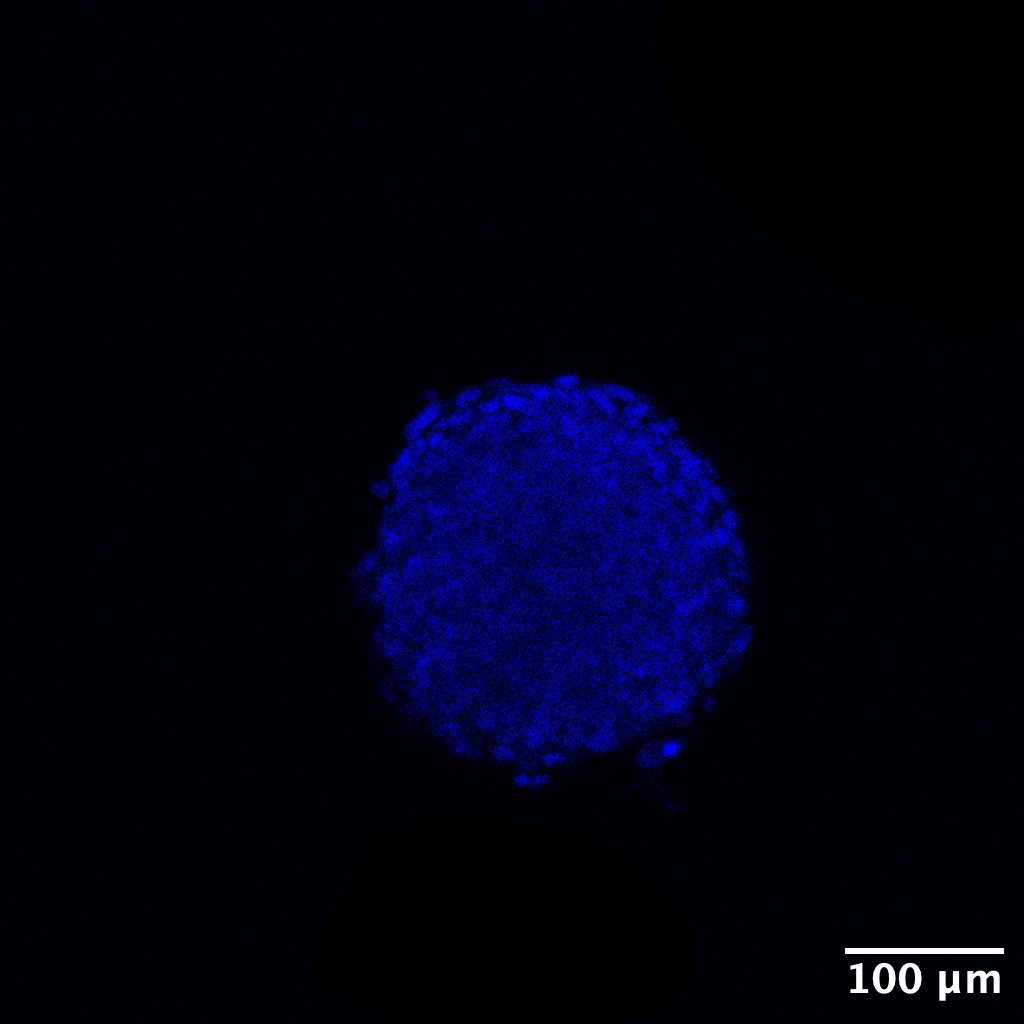

Supplement: Supplementary file 5 — Source data Fig. 3 [file 44318_2025_570_MOESM5_ESM.zip › Fig3/Images/H/Fig_3_panel_h_RP254_sh#5_5_blue.jpg]

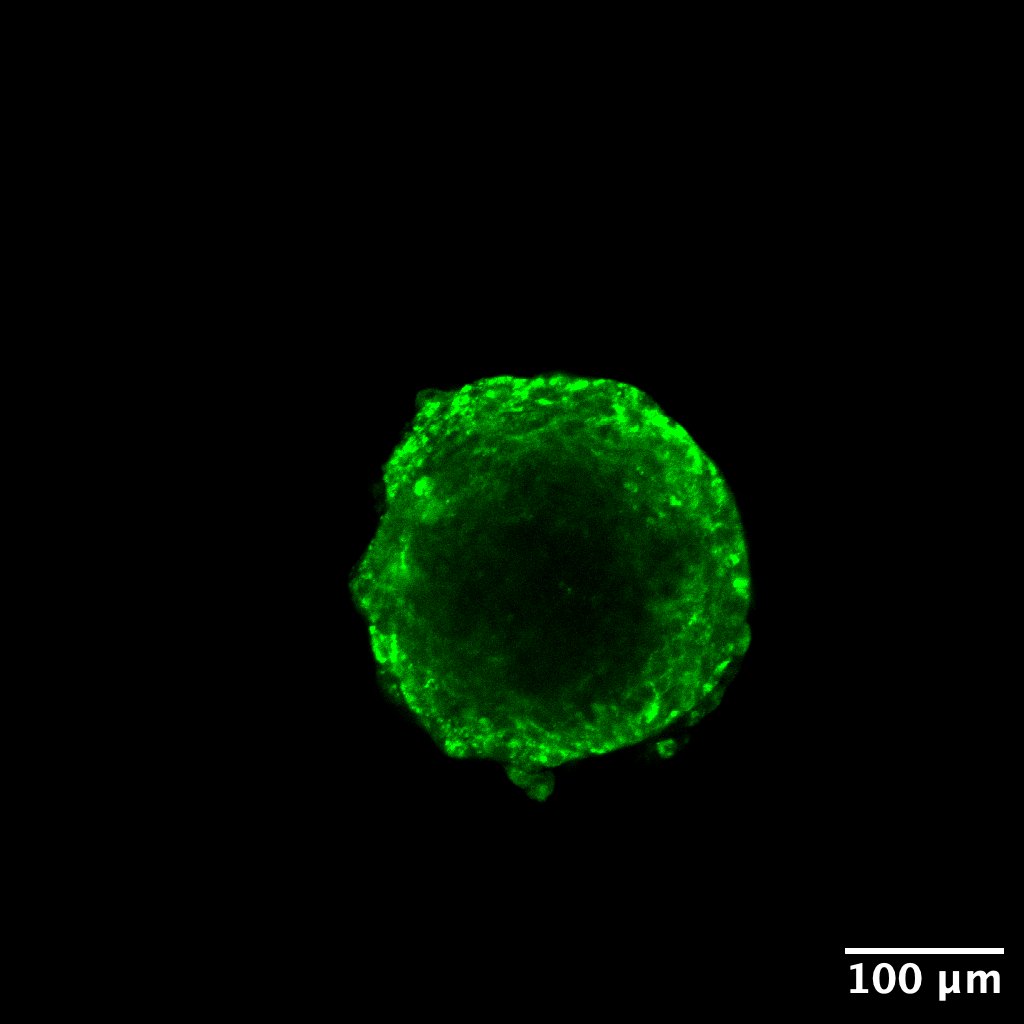

Supplement: Supplementary file 5 — Source data Fig. 3 [file 44318_2025_570_MOESM5_ESM.zip › Fig3/Images/H/Fig_3_panel_h_RP254_sh#5_5_green.jpg]

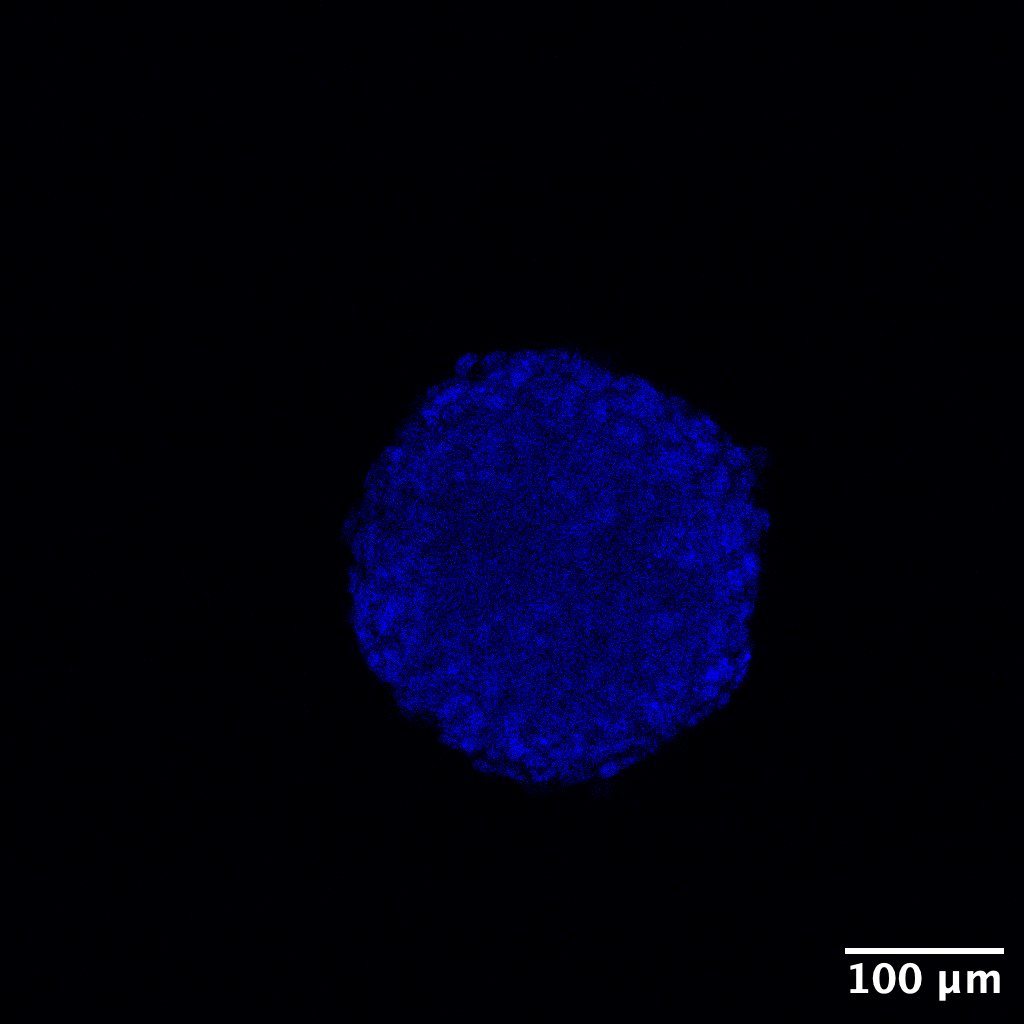

Supplement: Supplementary file 5 — Source data Fig. 3 [file 44318_2025_570_MOESM5_ESM.zip › Fig3/Images/H/Fig_3_panel_h_RP254_shNT_11_blue.jpg]

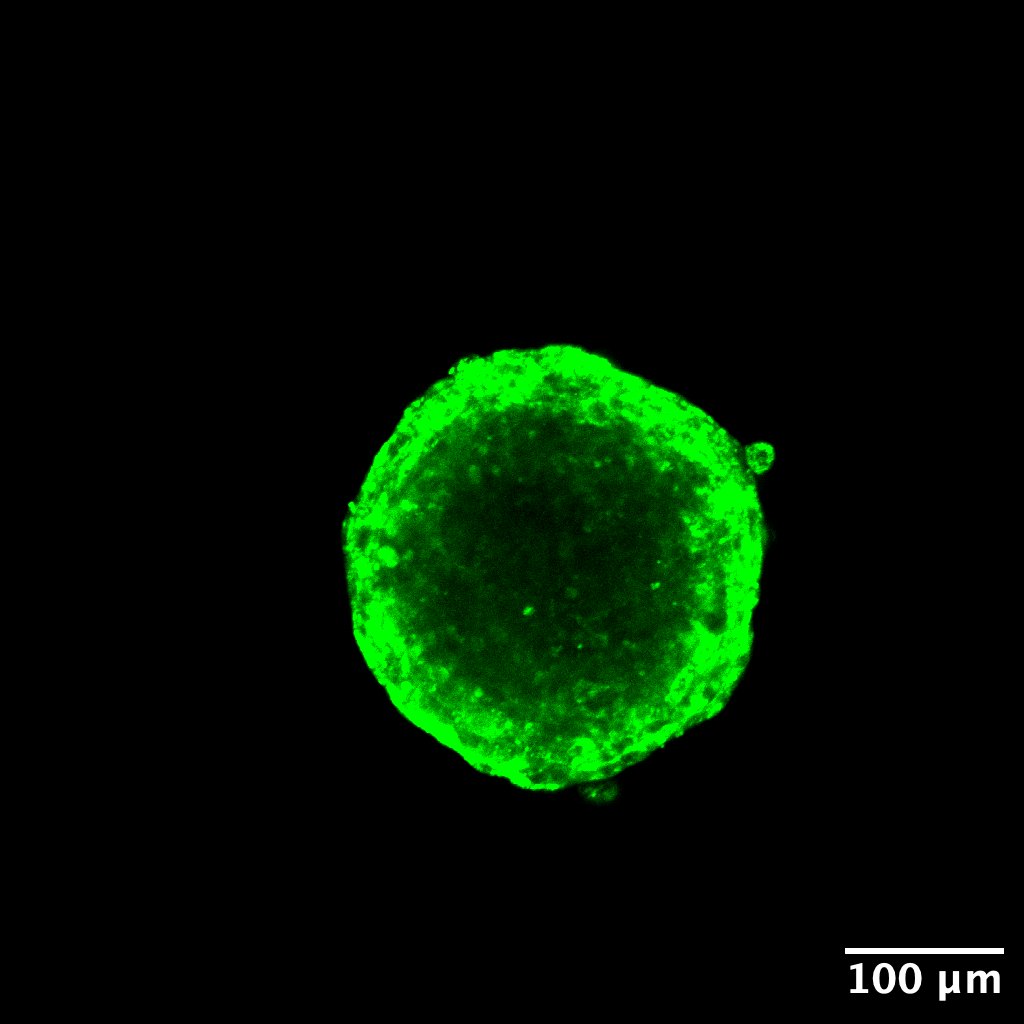

Supplement: Supplementary file 5 — Source data Fig. 3 [file 44318_2025_570_MOESM5_ESM.zip › Fig3/Images/H/Fig_3_panel_h_RP254_shNT_11_green.jpg]

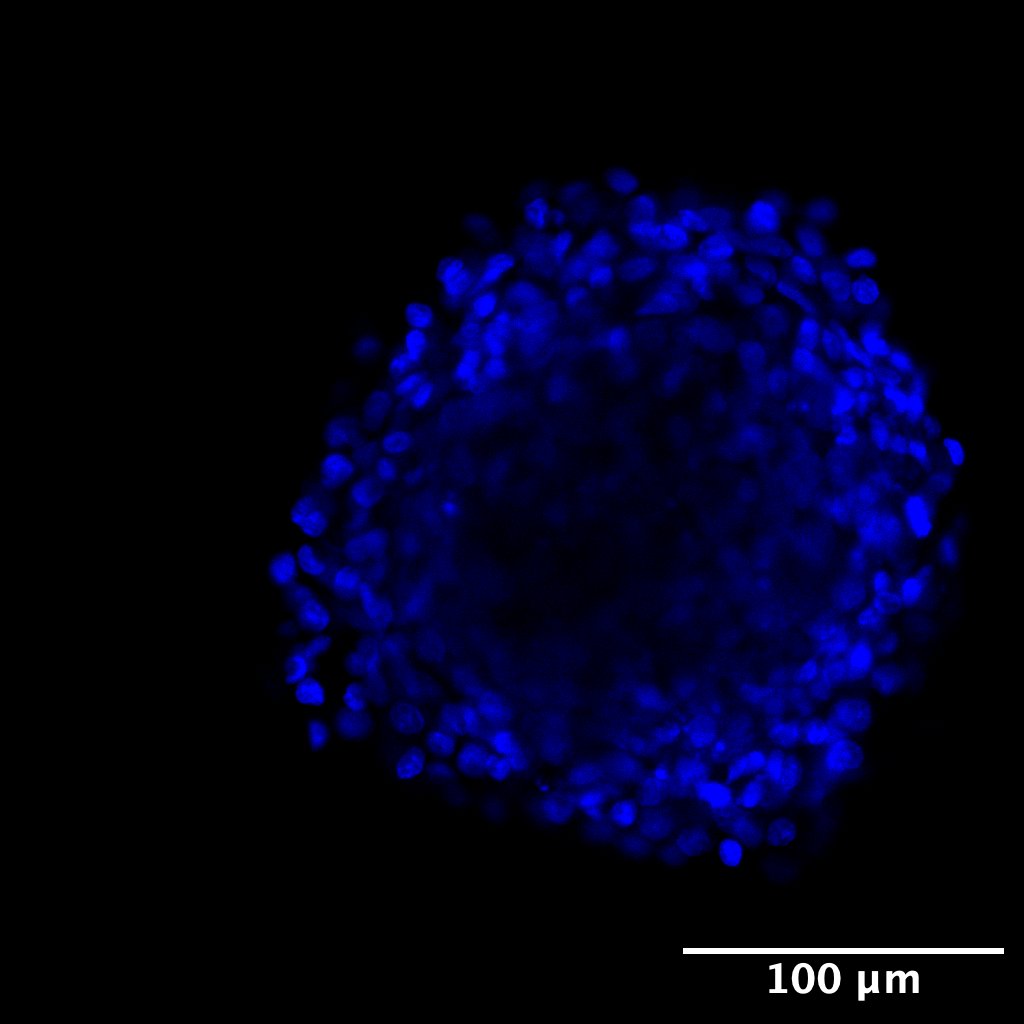

Supplement: Supplementary file 5 — Source data Fig. 3 [file 44318_2025_570_MOESM5_ESM.zip › Fig3/Images/J/Fig_3_panel_j_RP227_sh#1_12_blue.jpg]

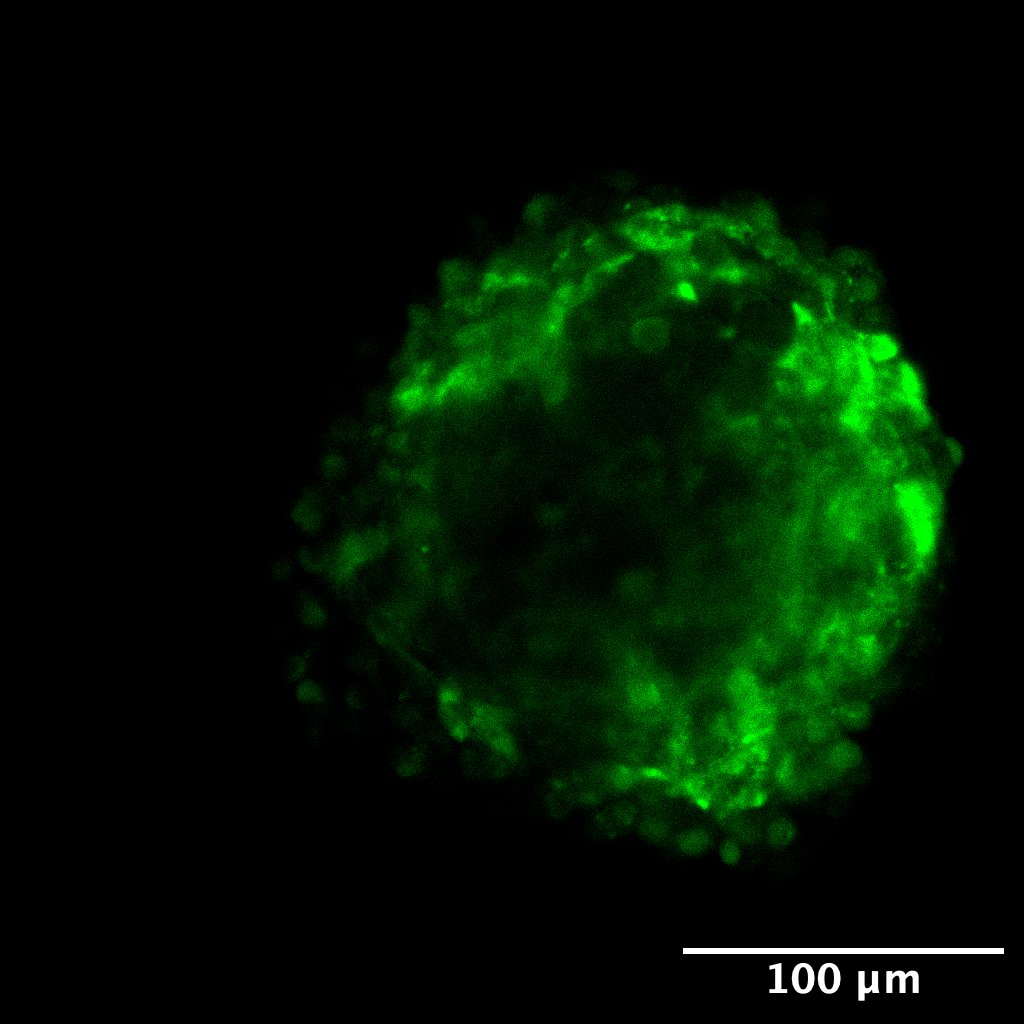

Supplement: Supplementary file 5 — Source data Fig. 3 [file 44318_2025_570_MOESM5_ESM.zip › Fig3/Images/J/Fig_3_panel_j_RP227_sh#1_12_green.jpg]

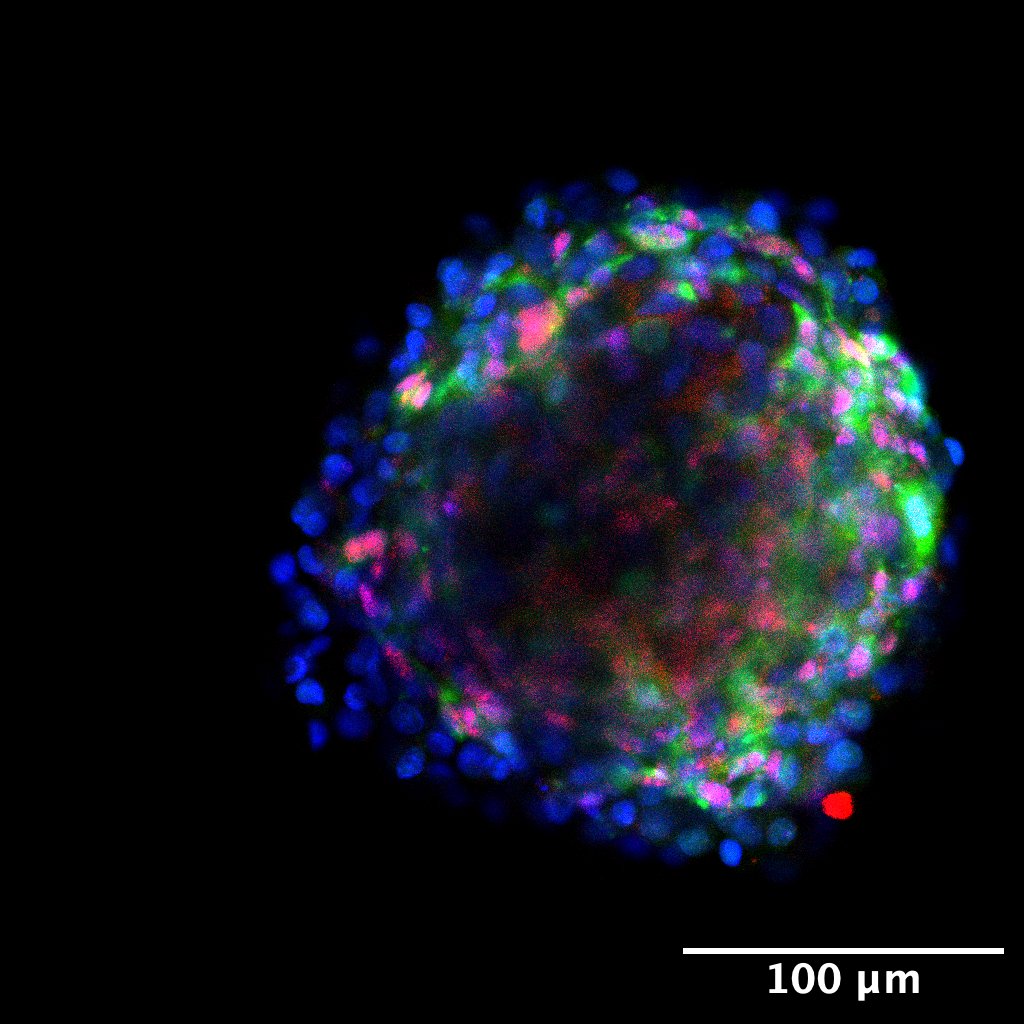

Supplement: Supplementary file 5 — Source data Fig. 3 [file 44318_2025_570_MOESM5_ESM.zip › Fig3/Images/J/Fig_3_panel_j_RP227_sh#1_12_merge.jpg]

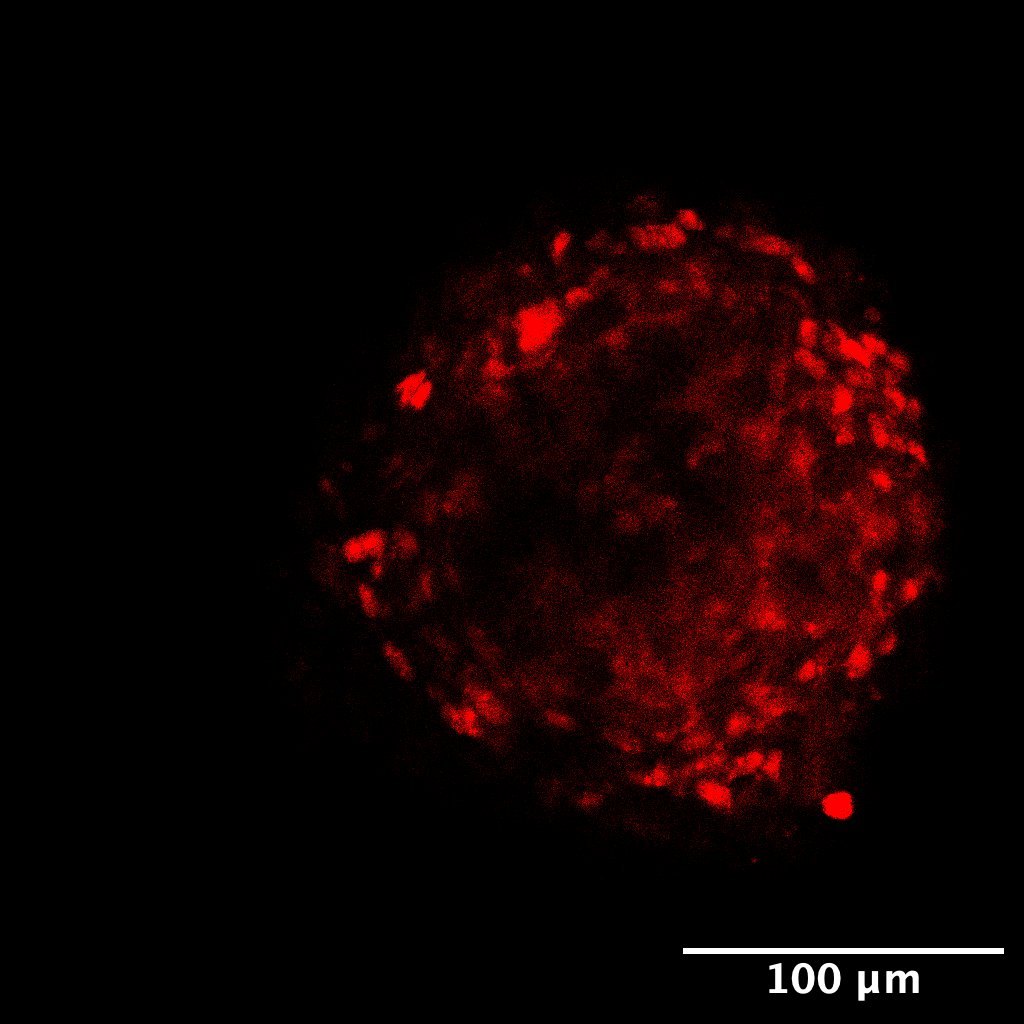

Supplement: Supplementary file 5 — Source data Fig. 3 [file 44318_2025_570_MOESM5_ESM.zip › Fig3/Images/J/Fig_3_panel_j_RP227_sh#1_12_red.jpg]

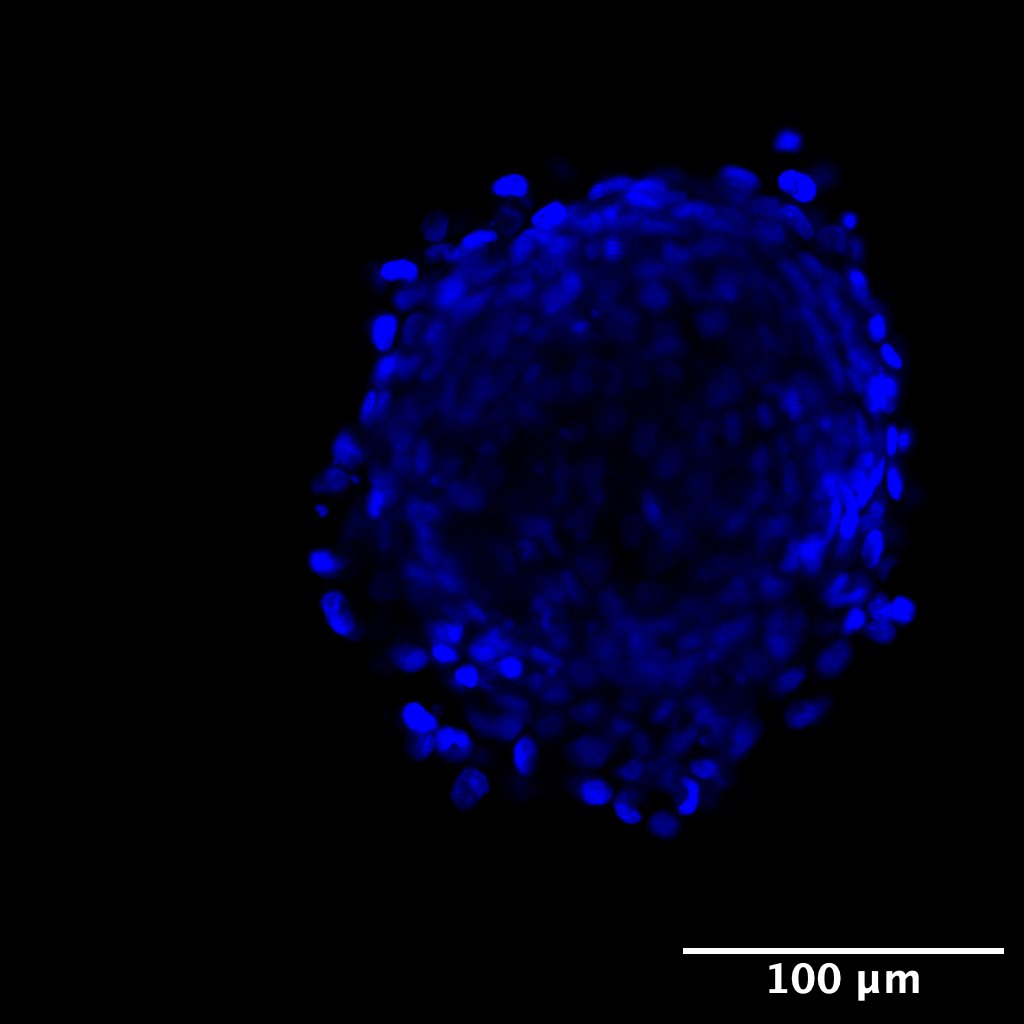

Supplement: Supplementary file 5 — Source data Fig. 3 [file 44318_2025_570_MOESM5_ESM.zip › Fig3/Images/J/Fig_3_panel_j_RP227_sh#5_10_blue.jpg]

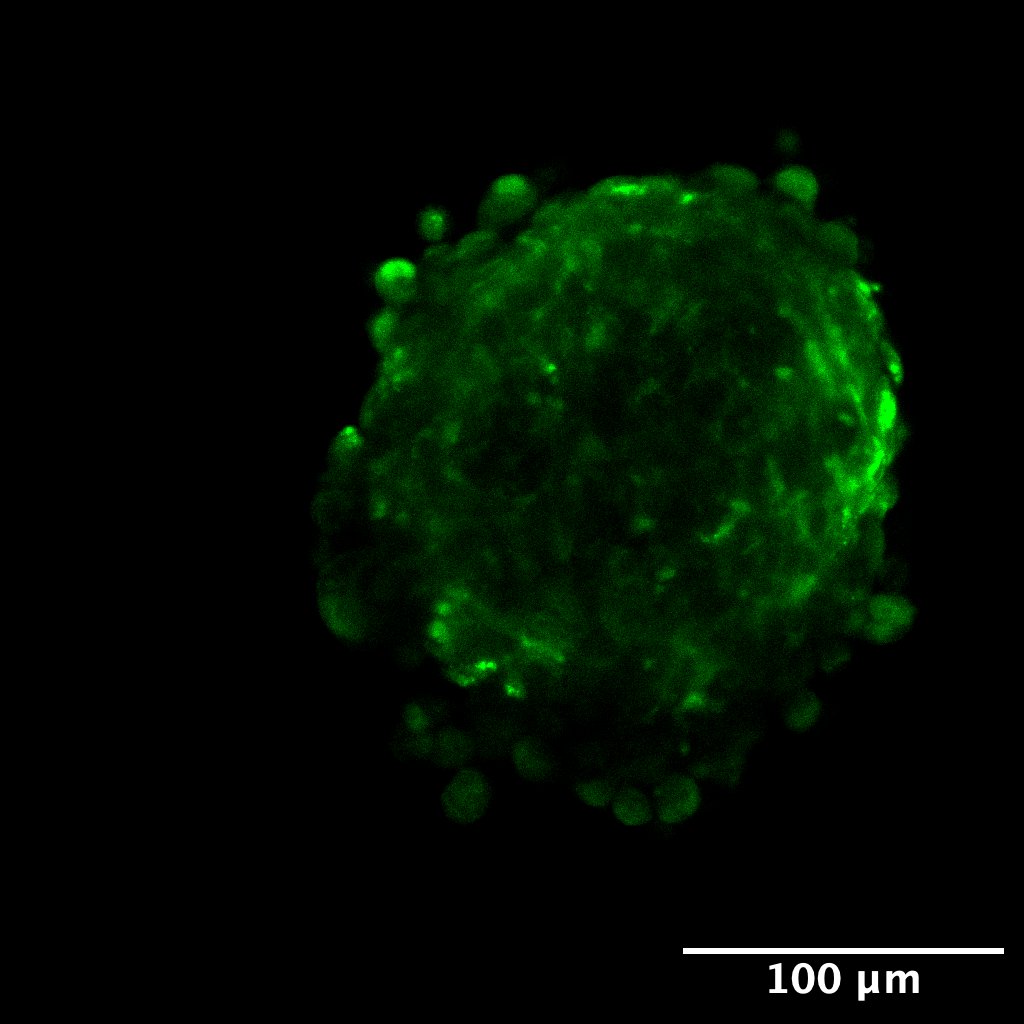

Supplement: Supplementary file 5 — Source data Fig. 3 [file 44318_2025_570_MOESM5_ESM.zip › Fig3/Images/J/Fig_3_panel_j_RP227_sh#5_10_green.jpg]

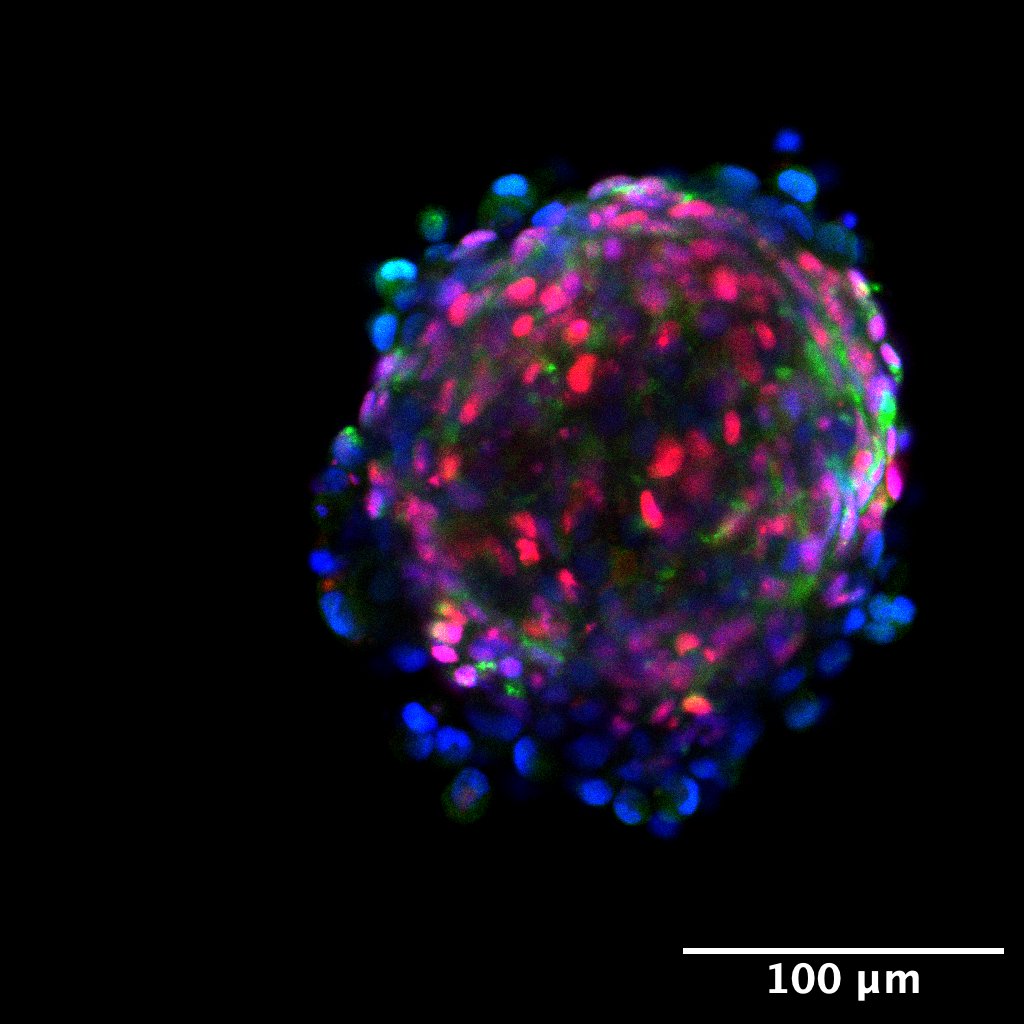

Supplement: Supplementary file 5 — Source data Fig. 3 [file 44318_2025_570_MOESM5_ESM.zip › Fig3/Images/J/Fig_3_panel_j_RP227_sh#5_10_merge.jpg]

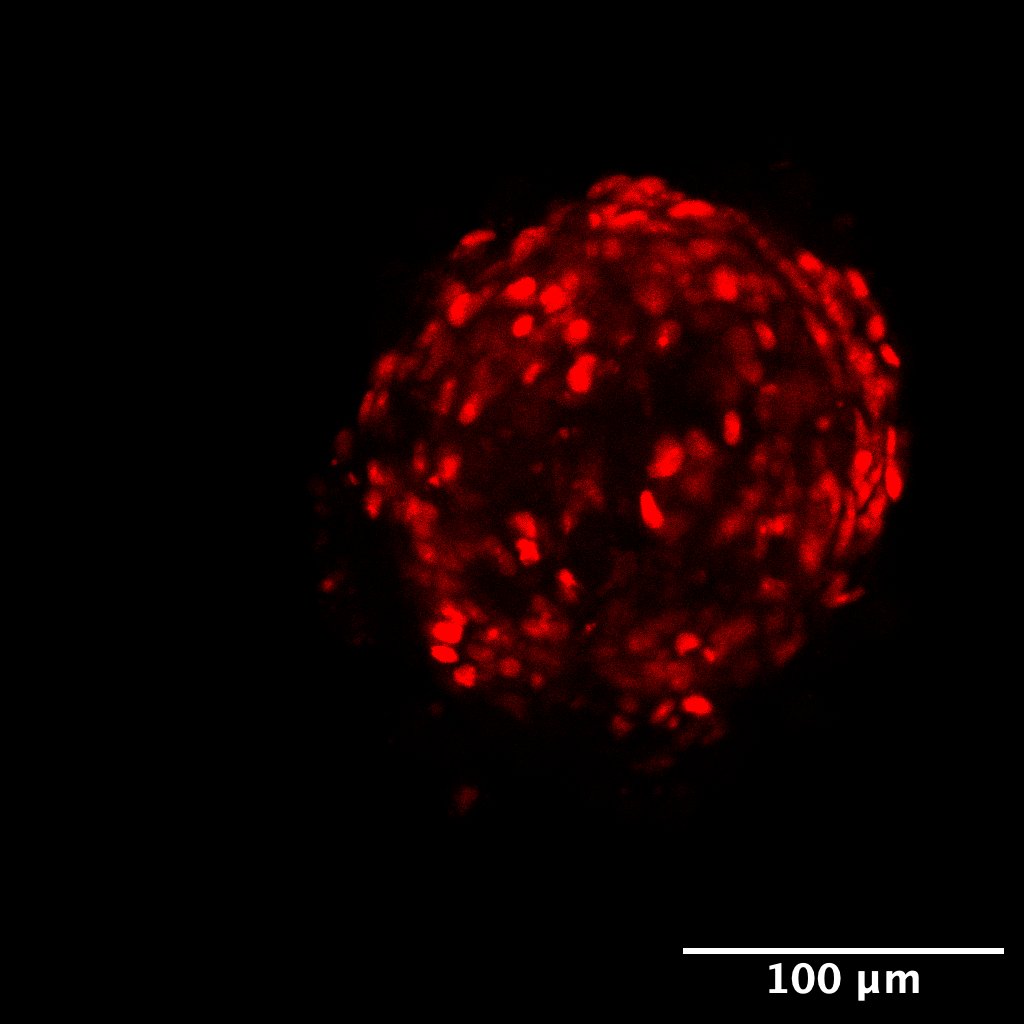

Supplement: Supplementary file 5 — Source data Fig. 3 [file 44318_2025_570_MOESM5_ESM.zip › Fig3/Images/J/Fig_3_panel_j_RP227_sh#5_10_red.jpg]

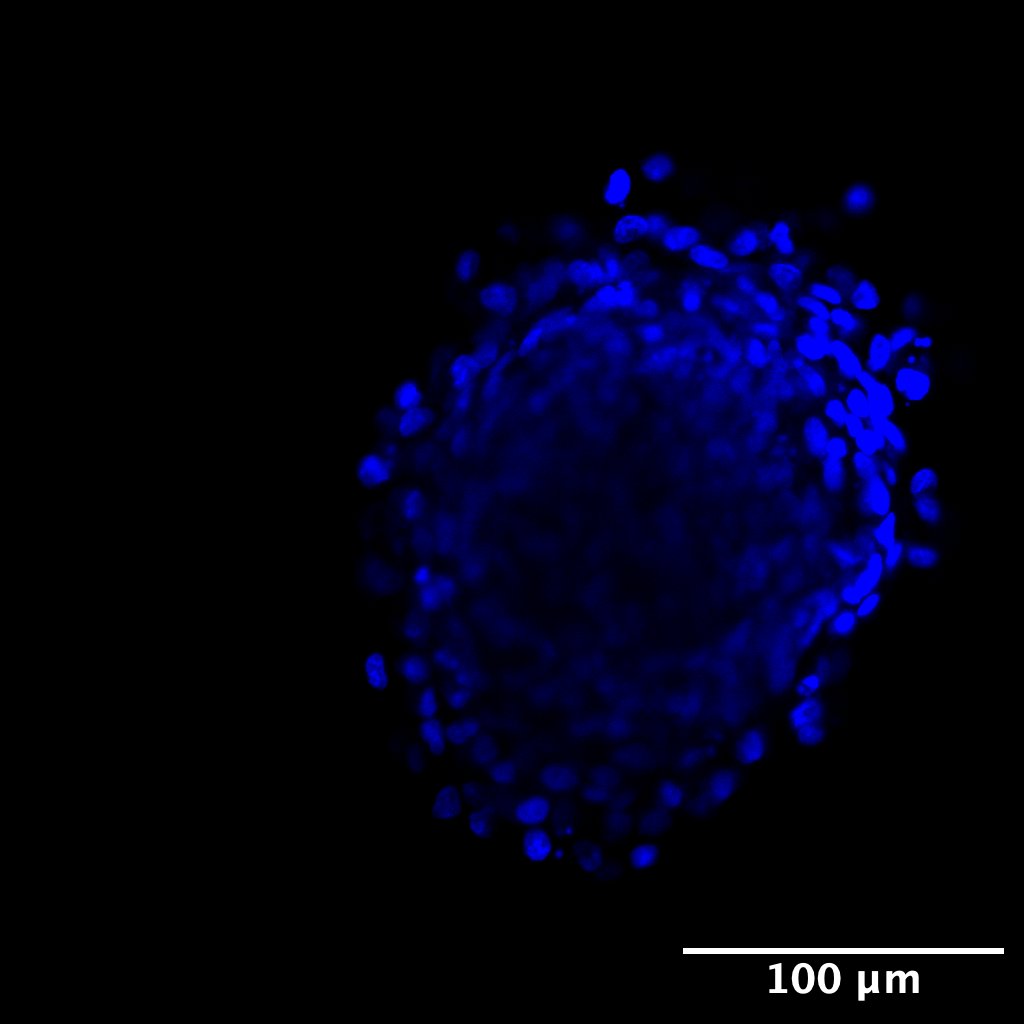

Supplement: Supplementary file 5 — Source data Fig. 3 [file 44318_2025_570_MOESM5_ESM.zip › Fig3/Images/J/Fig_3_panel_j_RP227_shNT_6_blue.jpg]

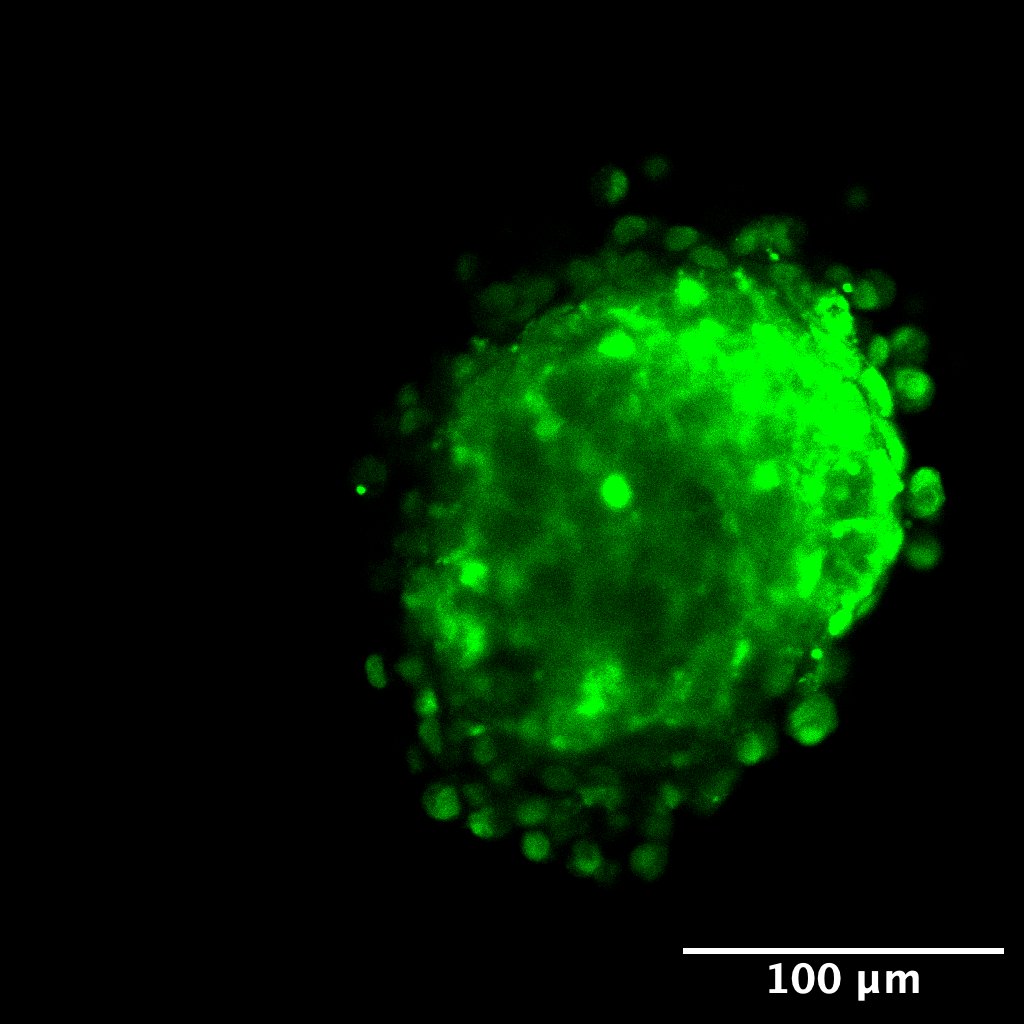

Supplement: Supplementary file 5 — Source data Fig. 3 [file 44318_2025_570_MOESM5_ESM.zip › Fig3/Images/J/Fig_3_panel_j_RP227_shNT_6_green.jpg]

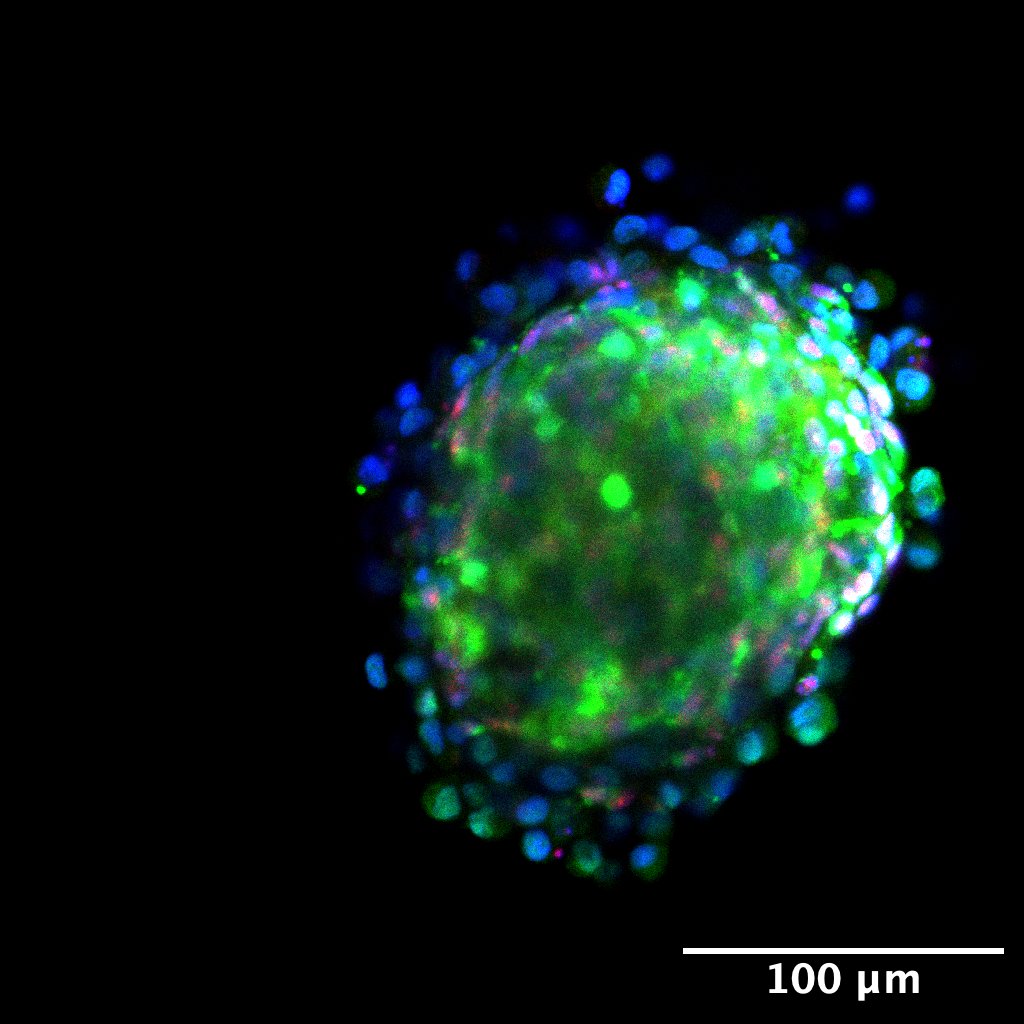

Supplement: Supplementary file 5 — Source data Fig. 3 [file 44318_2025_570_MOESM5_ESM.zip › Fig3/Images/J/Fig_3_panel_j_RP227_shNT_6_merge.jpg]

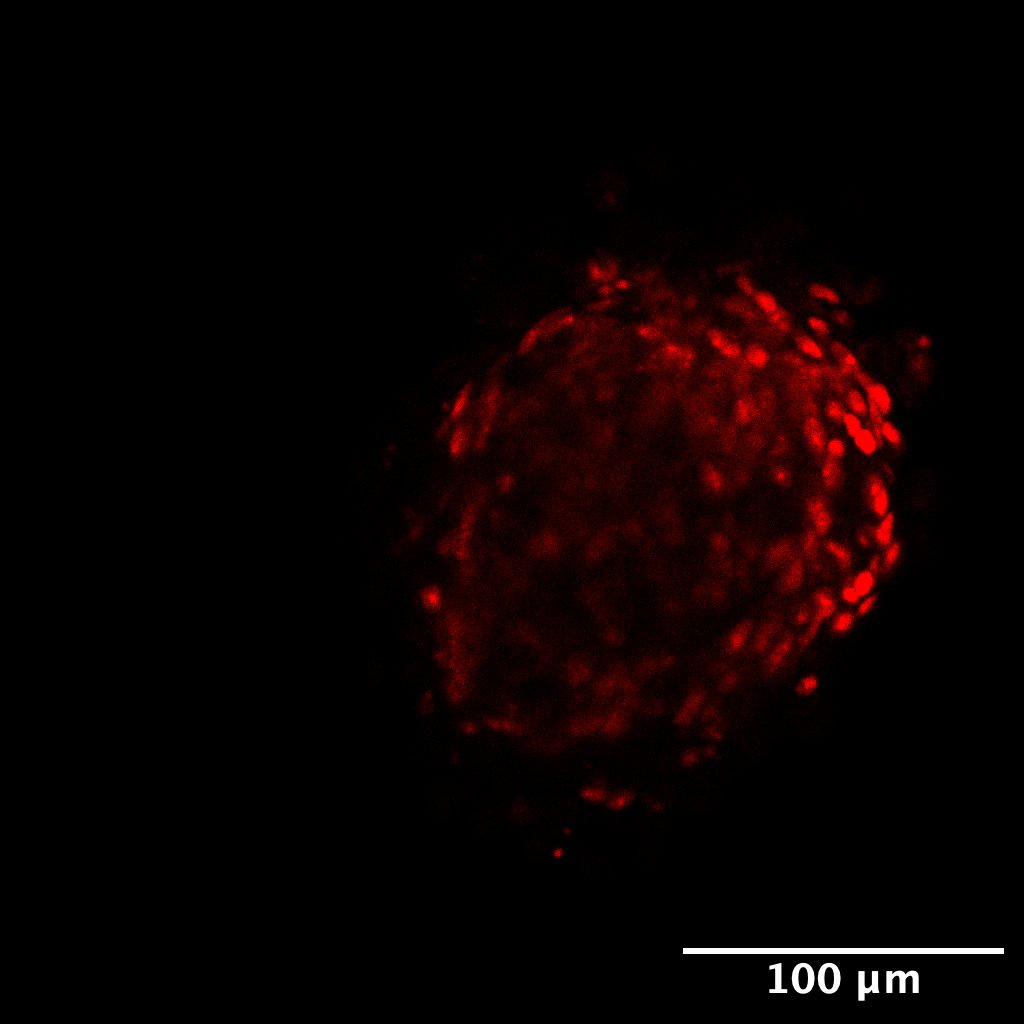

Supplement: Supplementary file 5 — Source data Fig. 3 [file 44318_2025_570_MOESM5_ESM.zip › Fig3/Images/J/Fig_3_panel_j_RP227_shNT_6_red.jpg]

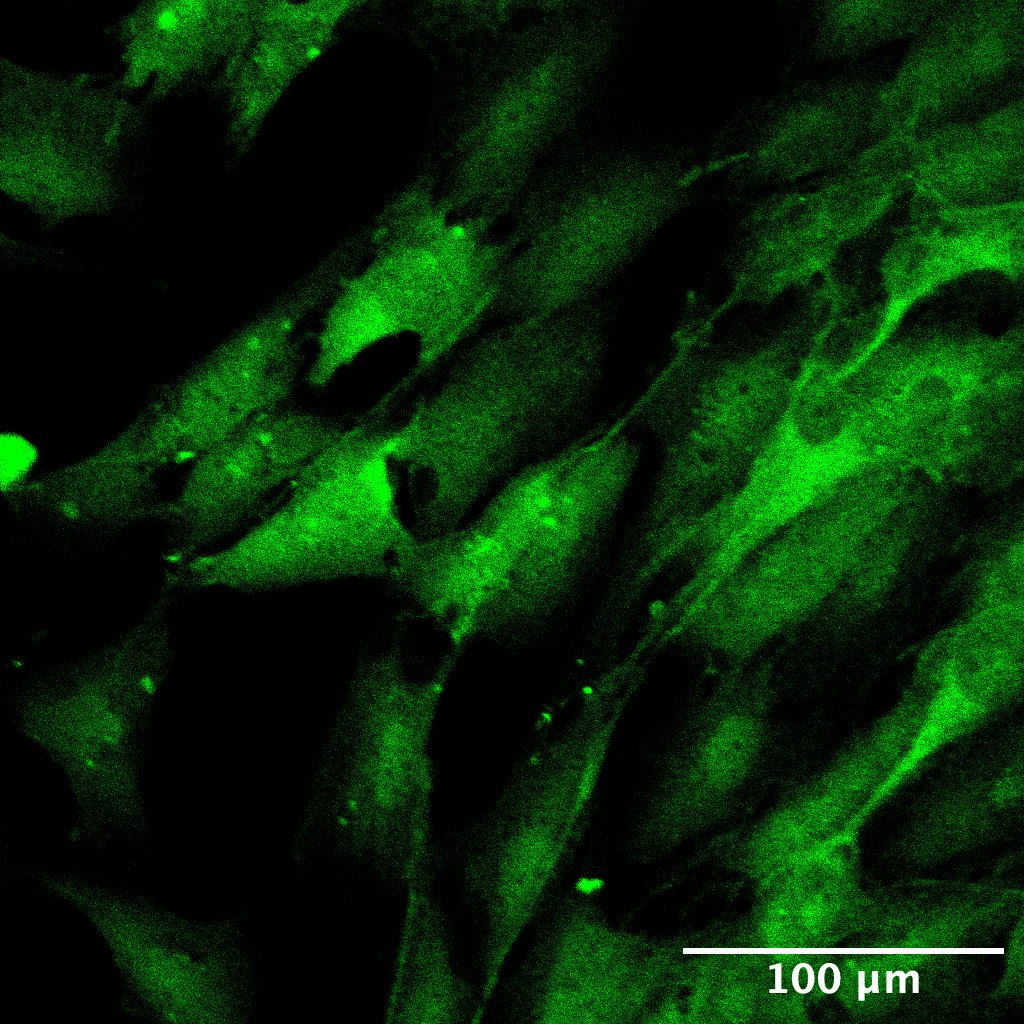

Supplement: Supplementary file 6 — Source data Fig. 4 [file 44318_2025_570_MOESM6_ESM.zip › Fig4/Images/F/Fig_4_panel_f_RP259_0min_sh#1_3.jpg]

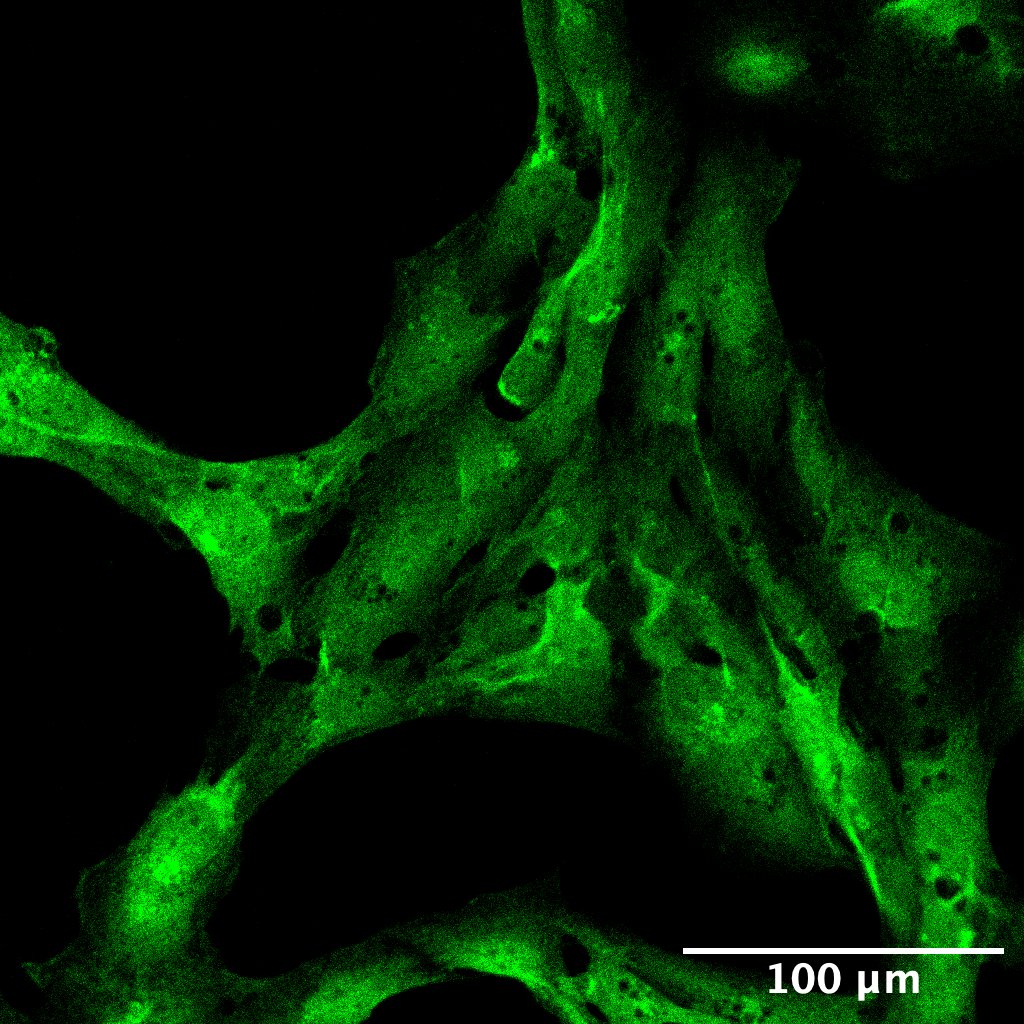

Supplement: Supplementary file 6 — Source data Fig. 4 [file 44318_2025_570_MOESM6_ESM.zip › Fig4/Images/F/Fig_4_panel_f_RP259_0min_sh#5_1.jpg]

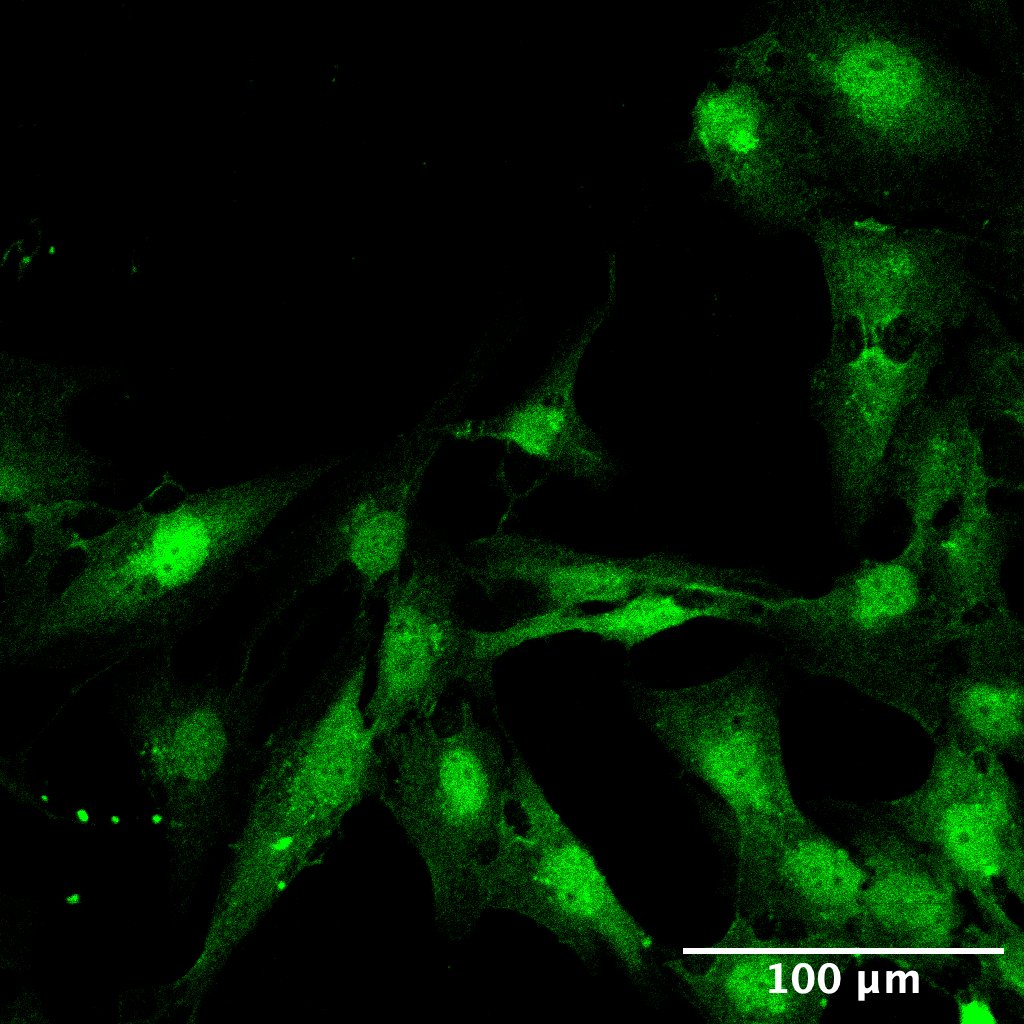

Supplement: Supplementary file 6 — Source data Fig. 4 [file 44318_2025_570_MOESM6_ESM.zip › Fig4/Images/F/Fig_4_panel_f_RP259_0min_shNT_1.jpg]

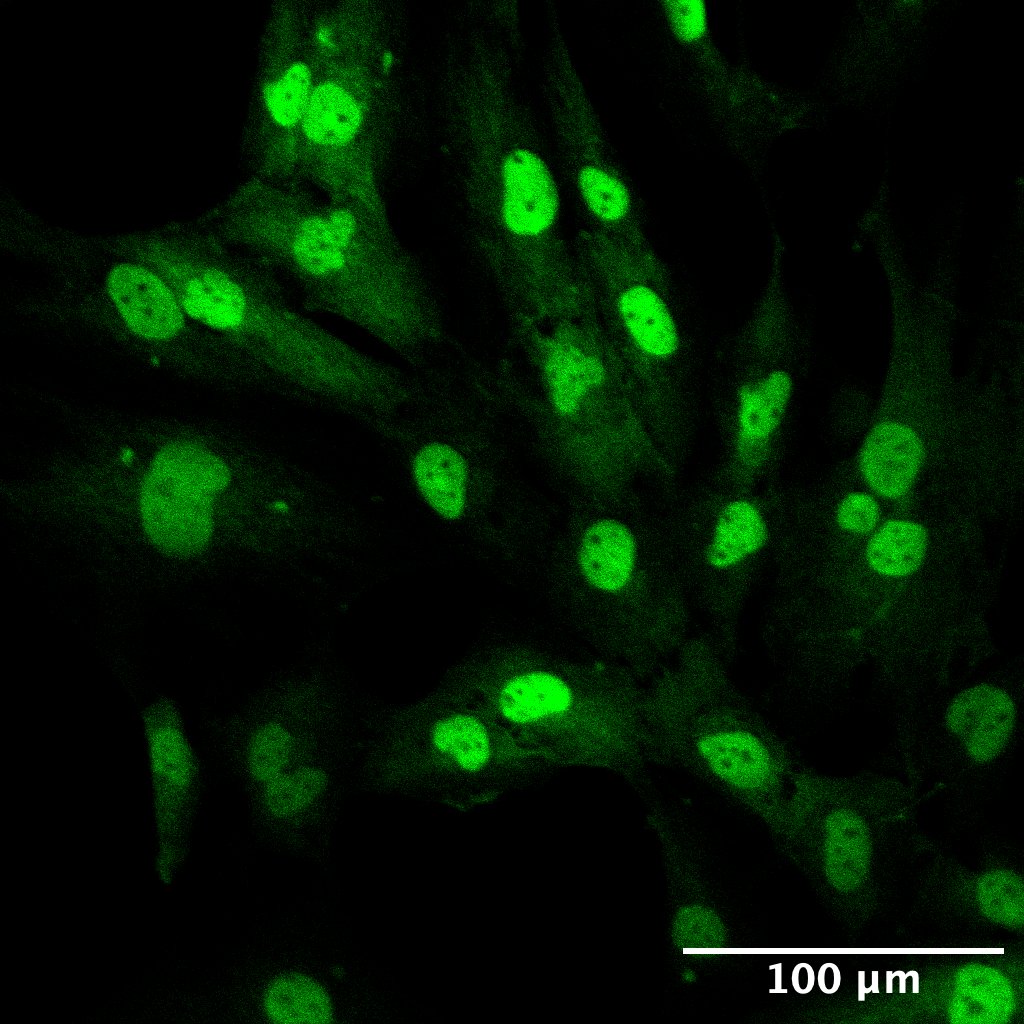

Supplement: Supplementary file 6 — Source data Fig. 4 [file 44318_2025_570_MOESM6_ESM.zip › Fig4/Images/F/Fig_4_panel_f_RP259_30min_sh#1_1.jpg]

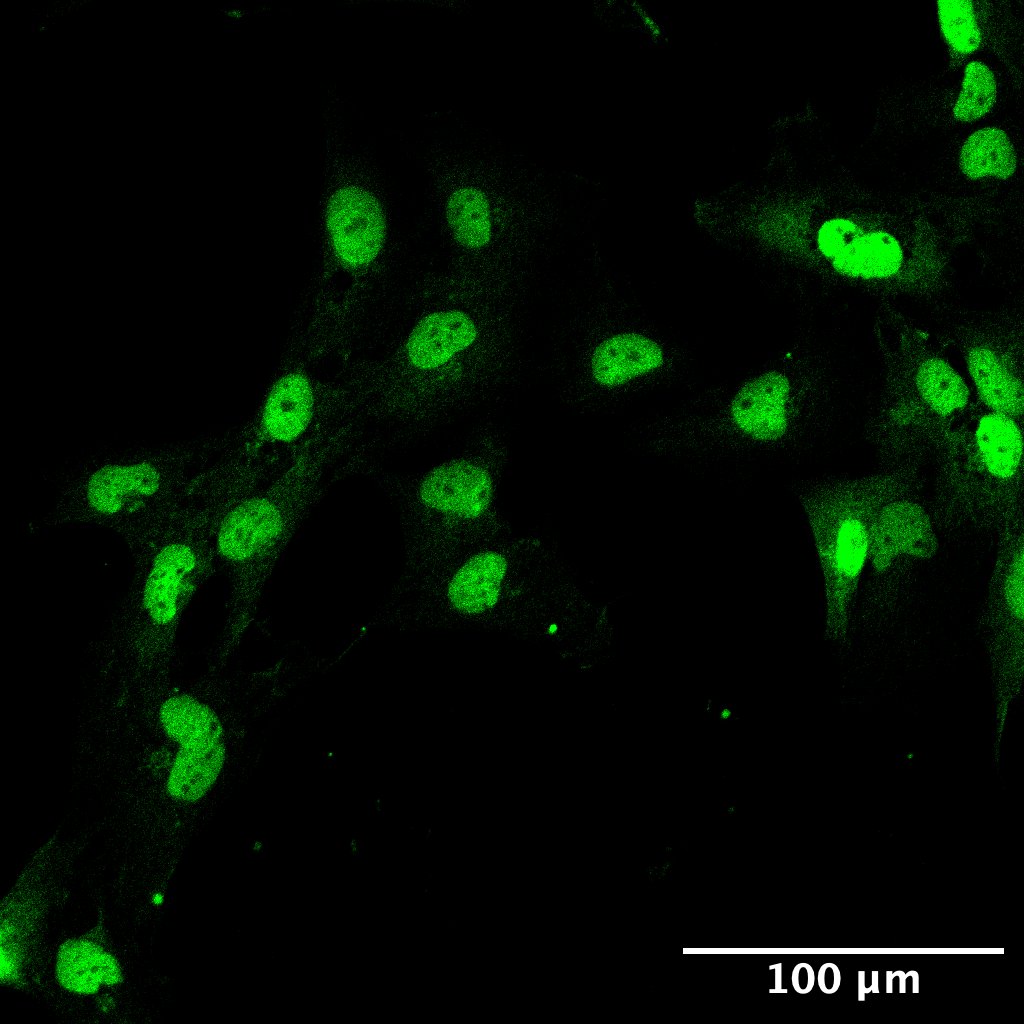

Supplement: Supplementary file 6 — Source data Fig. 4 [file 44318_2025_570_MOESM6_ESM.zip › Fig4/Images/F/Fig_4_panel_f_RP259_30min_sh#5_2.jpg]

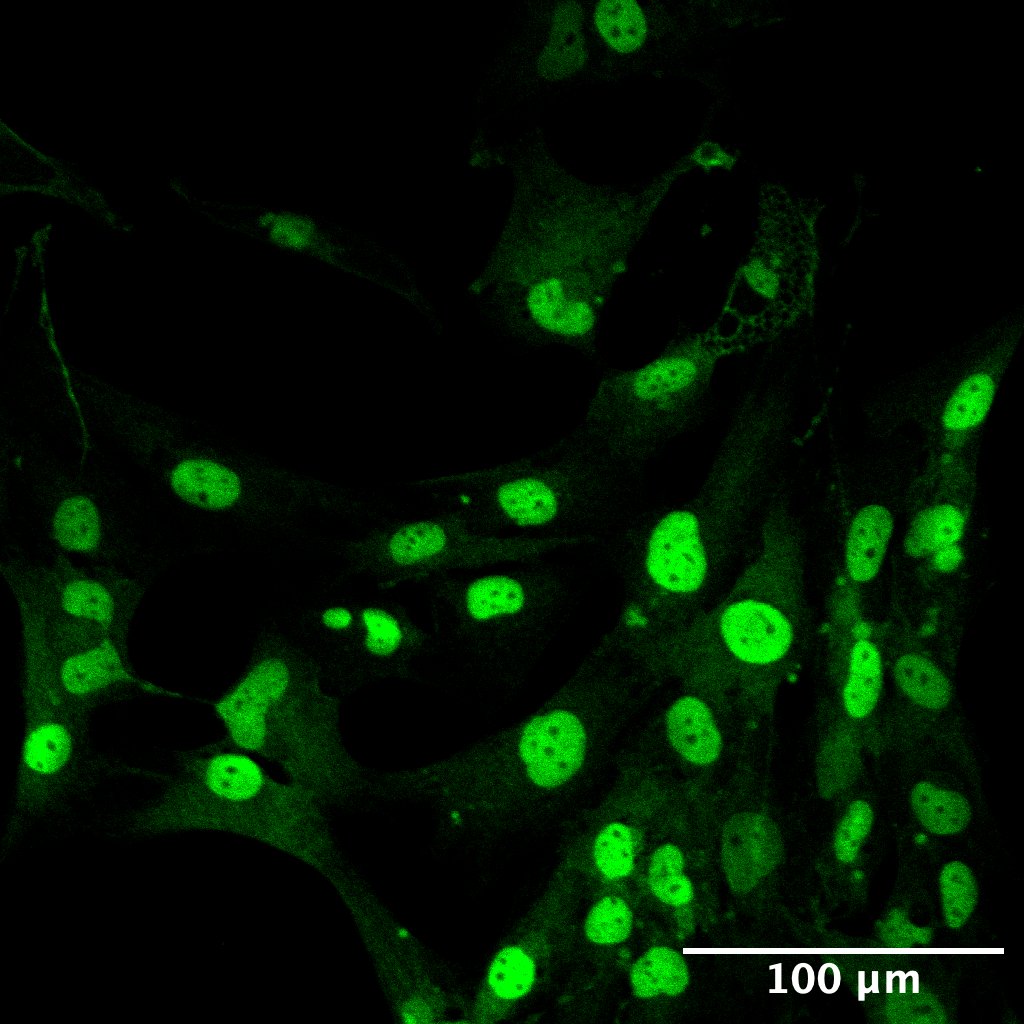

Supplement: Supplementary file 6 — Source data Fig. 4 [file 44318_2025_570_MOESM6_ESM.zip › Fig4/Images/F/Fig_4_panel_f_RP259_30min_shNT_1.jpg]

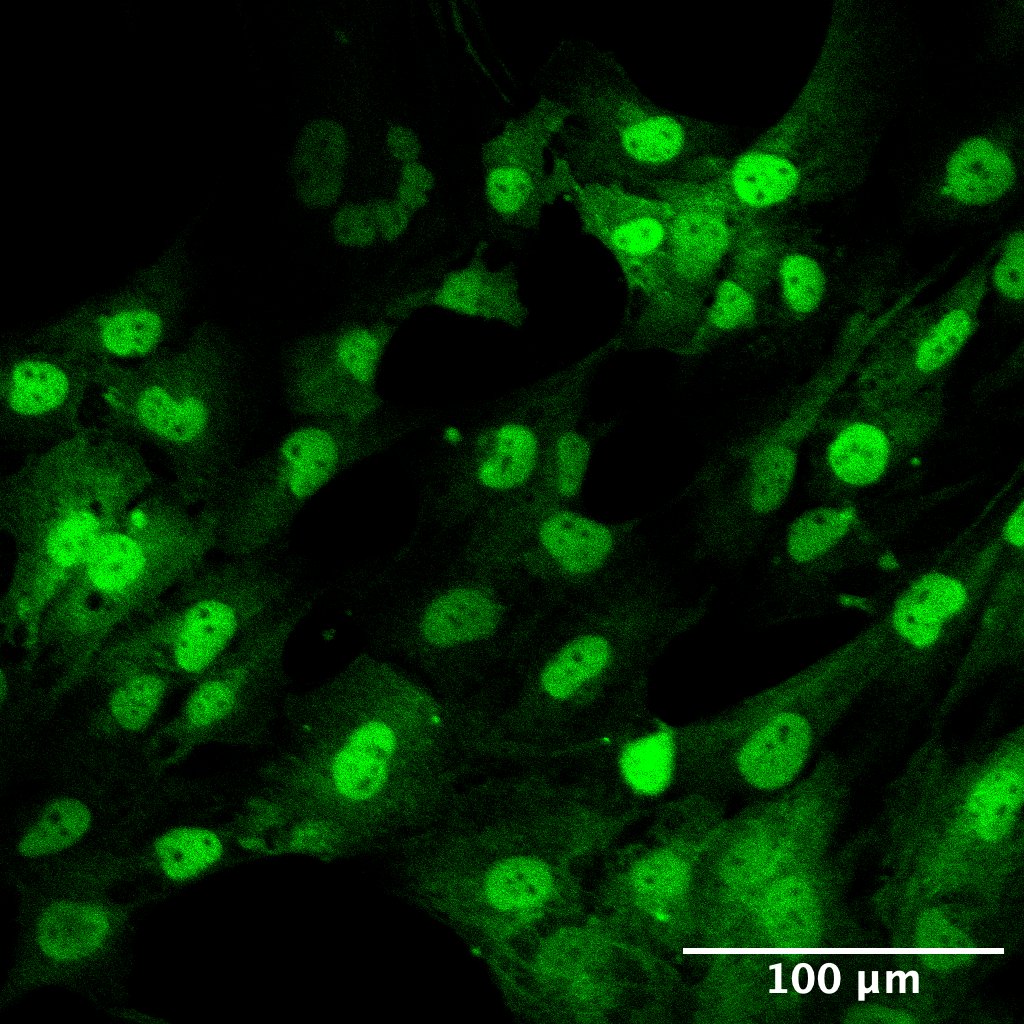

Supplement: Supplementary file 6 — Source data Fig. 4 [file 44318_2025_570_MOESM6_ESM.zip › Fig4/Images/F/Fig_4_panel_f_RP259_60min_sh#1_2.jpg]

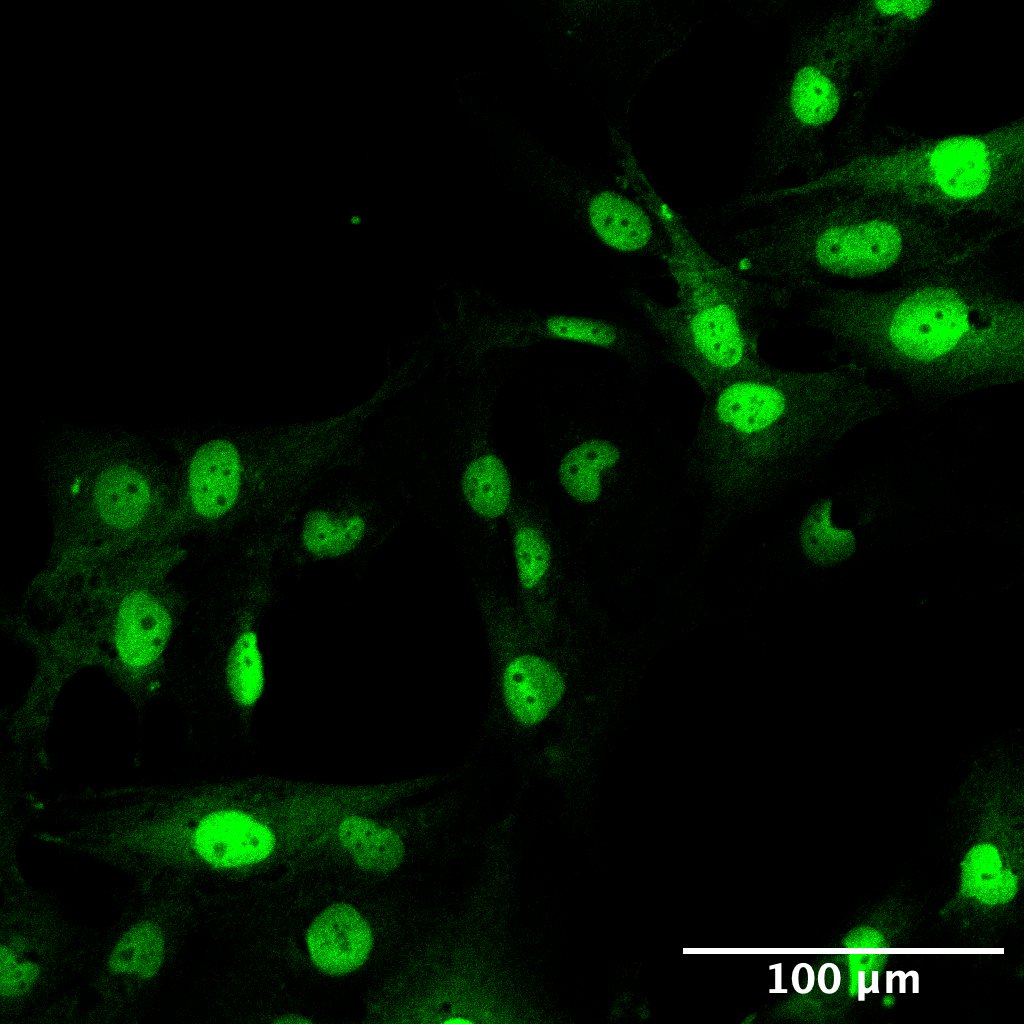

Supplement: Supplementary file 6 — Source data Fig. 4 [file 44318_2025_570_MOESM6_ESM.zip › Fig4/Images/F/Fig_4_panel_f_RP259_60min_sh#5_3.jpg]

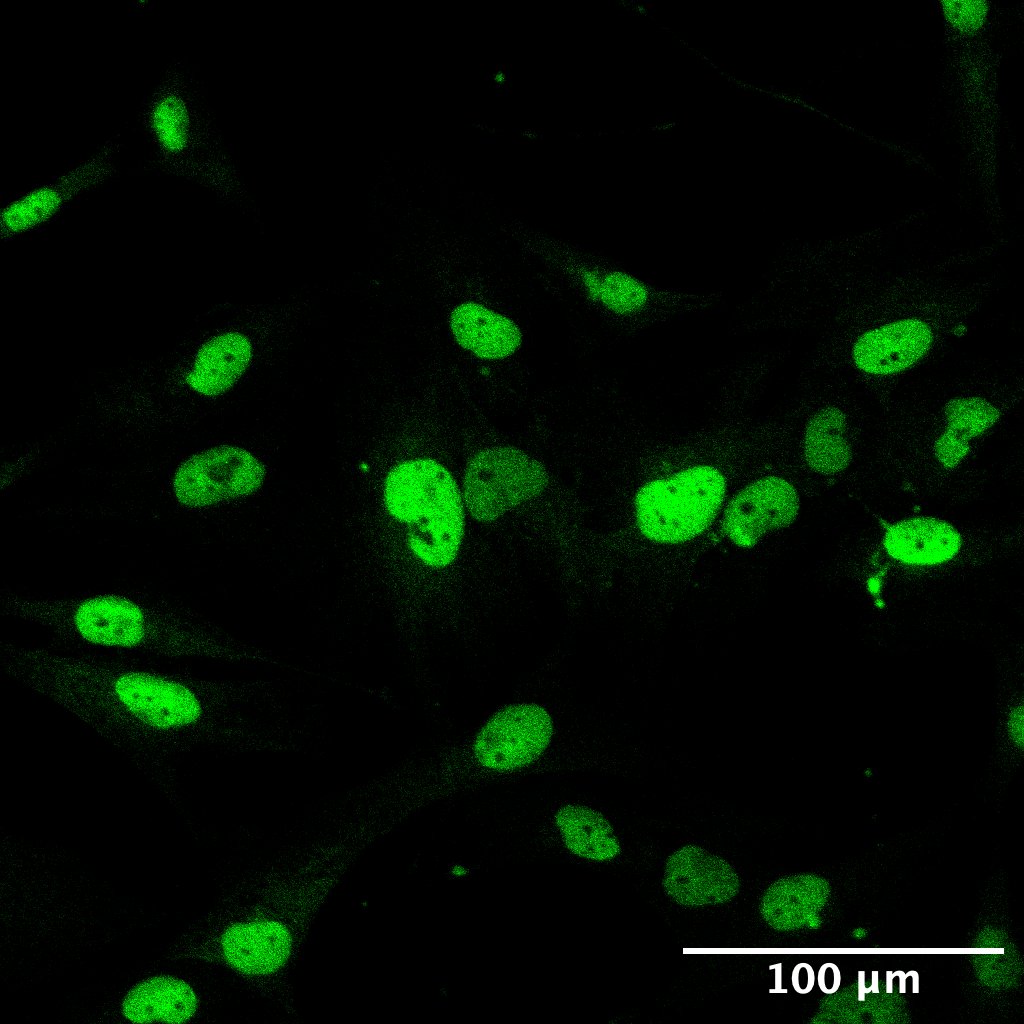

Supplement: Supplementary file 6 — Source data Fig. 4 [file 44318_2025_570_MOESM6_ESM.zip › Fig4/Images/F/Fig_4_panel_f_RP259_60min_shNT_1.jpg]

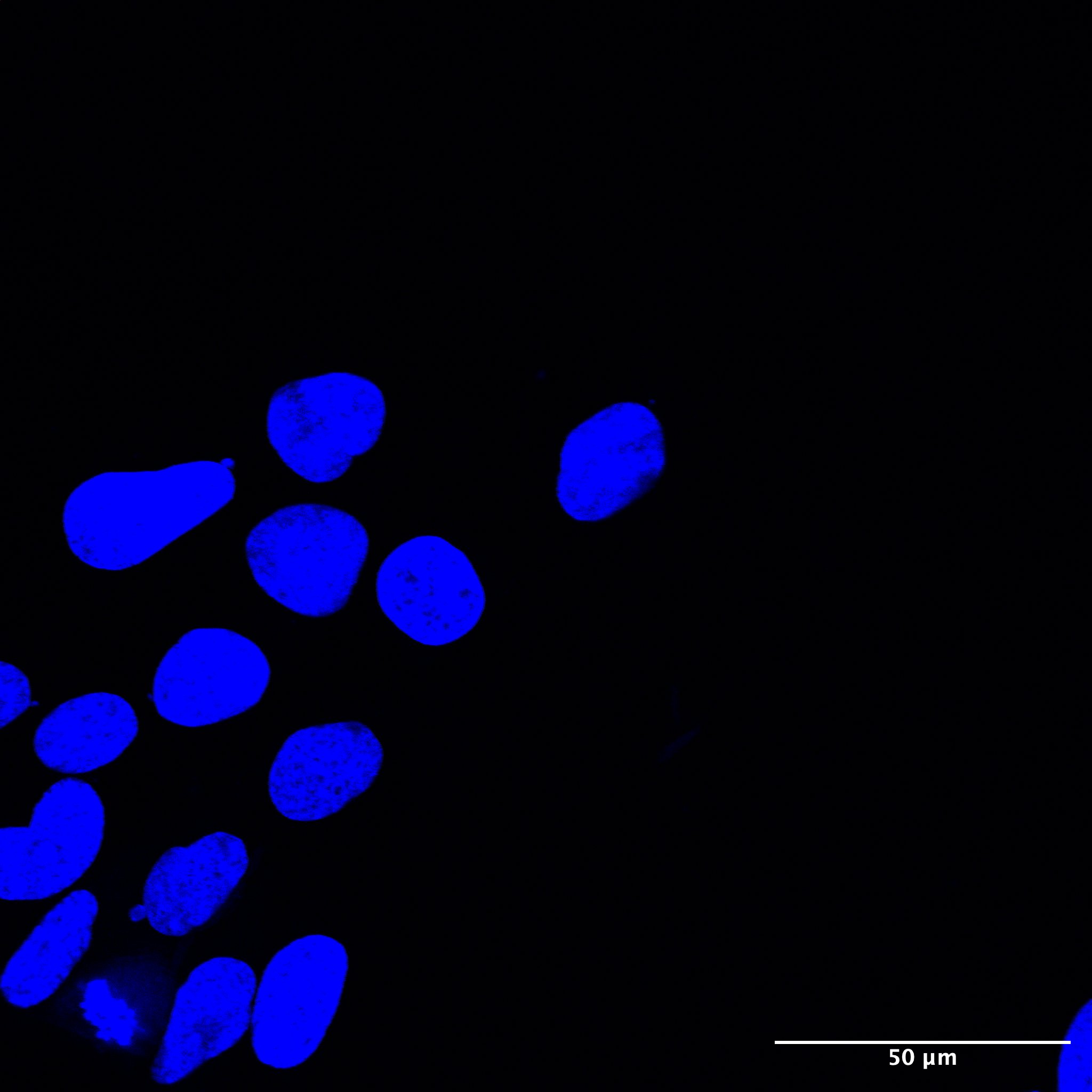

Supplement: Supplementary file 7 — Source data Fig. 5 [file 44318_2025_570_MOESM7_ESM.zip › Fig5/Images/C/Fig_5_panel_c_Sec24C_Golgin97_BFA_30min_1_blue.jpg]

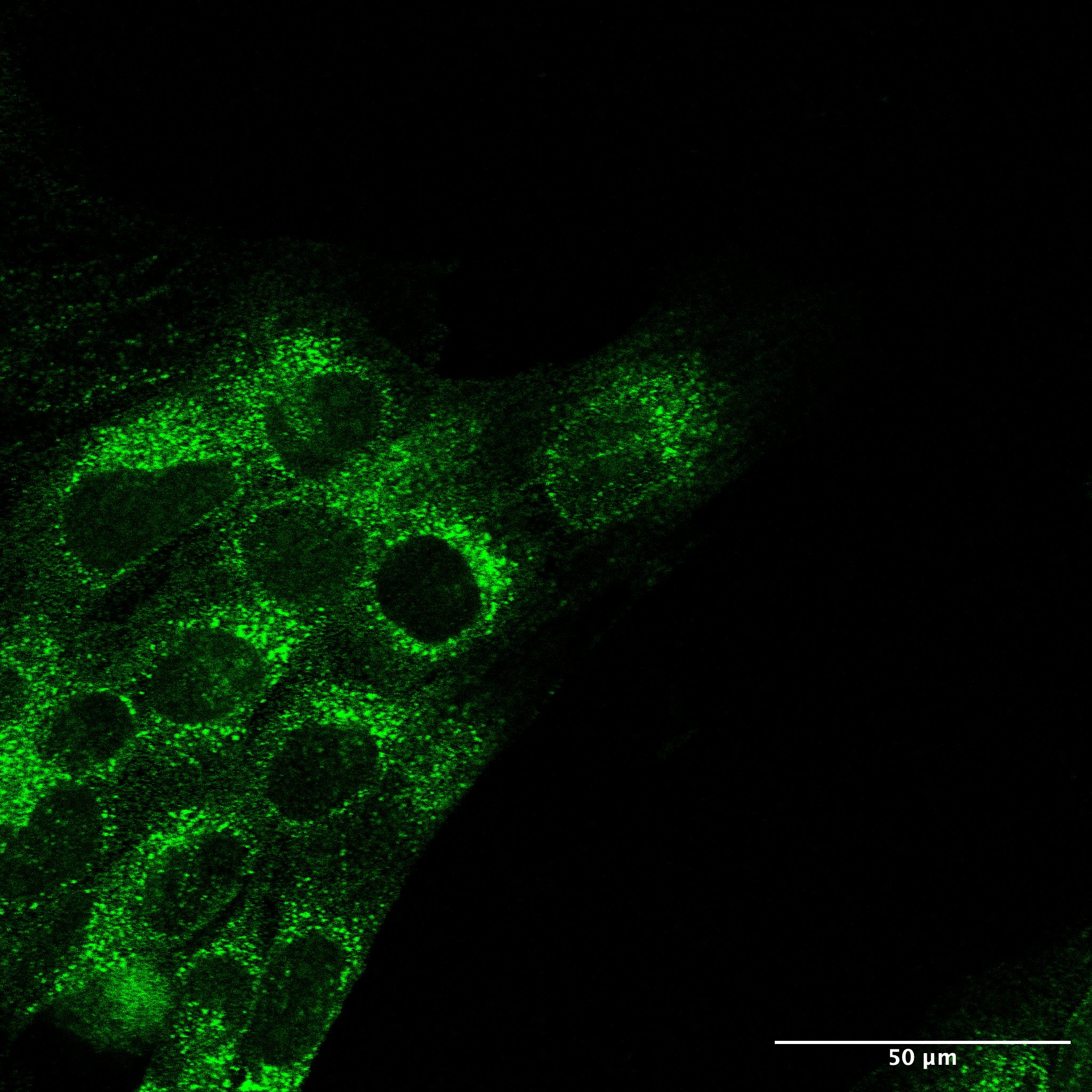

Supplement: Supplementary file 7 — Source data Fig. 5 [file 44318_2025_570_MOESM7_ESM.zip › Fig5/Images/C/Fig_5_panel_c_Sec24C_Golgin97_BFA_30min_1_green.jpg]

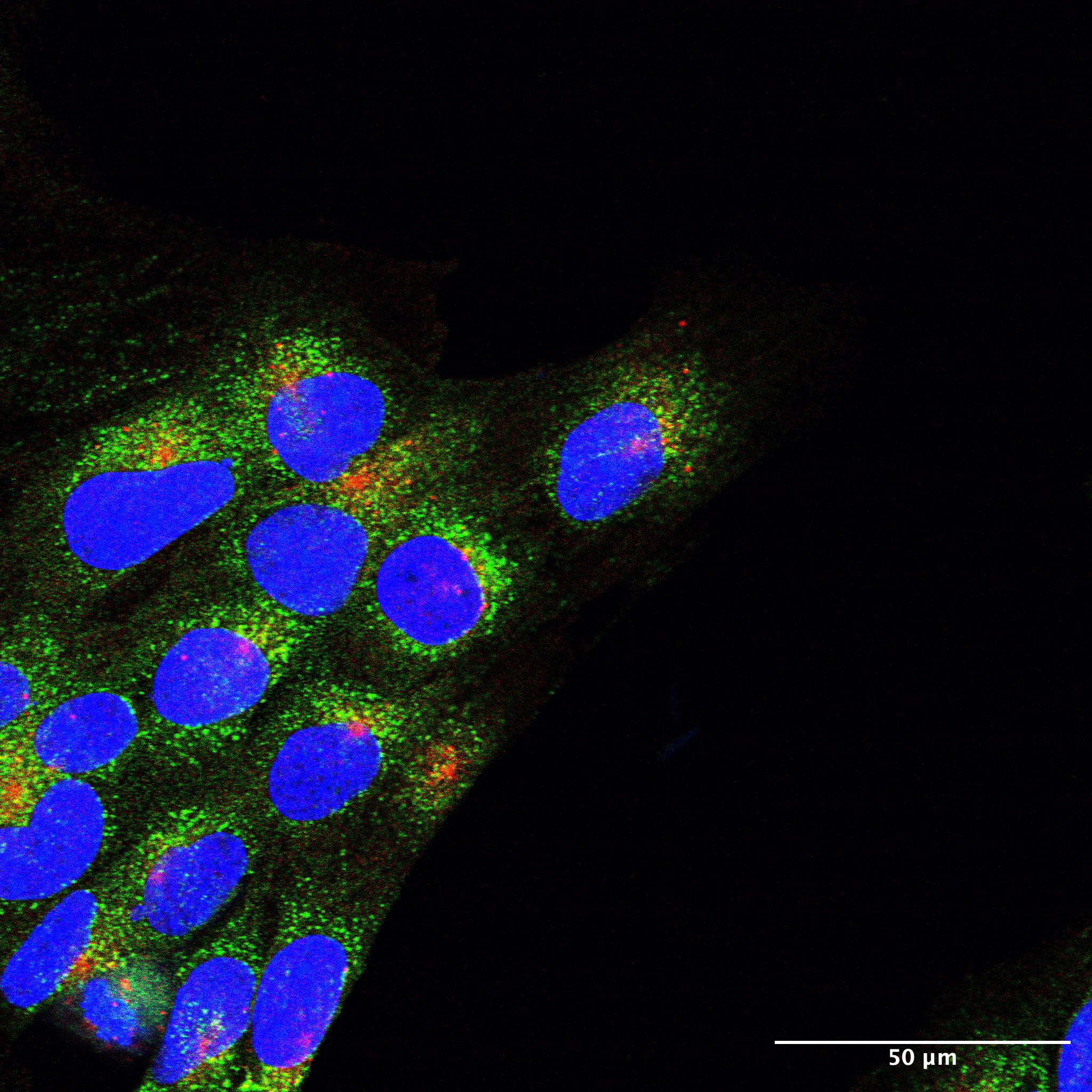

Supplement: Supplementary file 7 — Source data Fig. 5 [file 44318_2025_570_MOESM7_ESM.zip › Fig5/Images/C/Fig_5_panel_c_Sec24C_Golgin97_BFA_30min_1_merge.jpg]

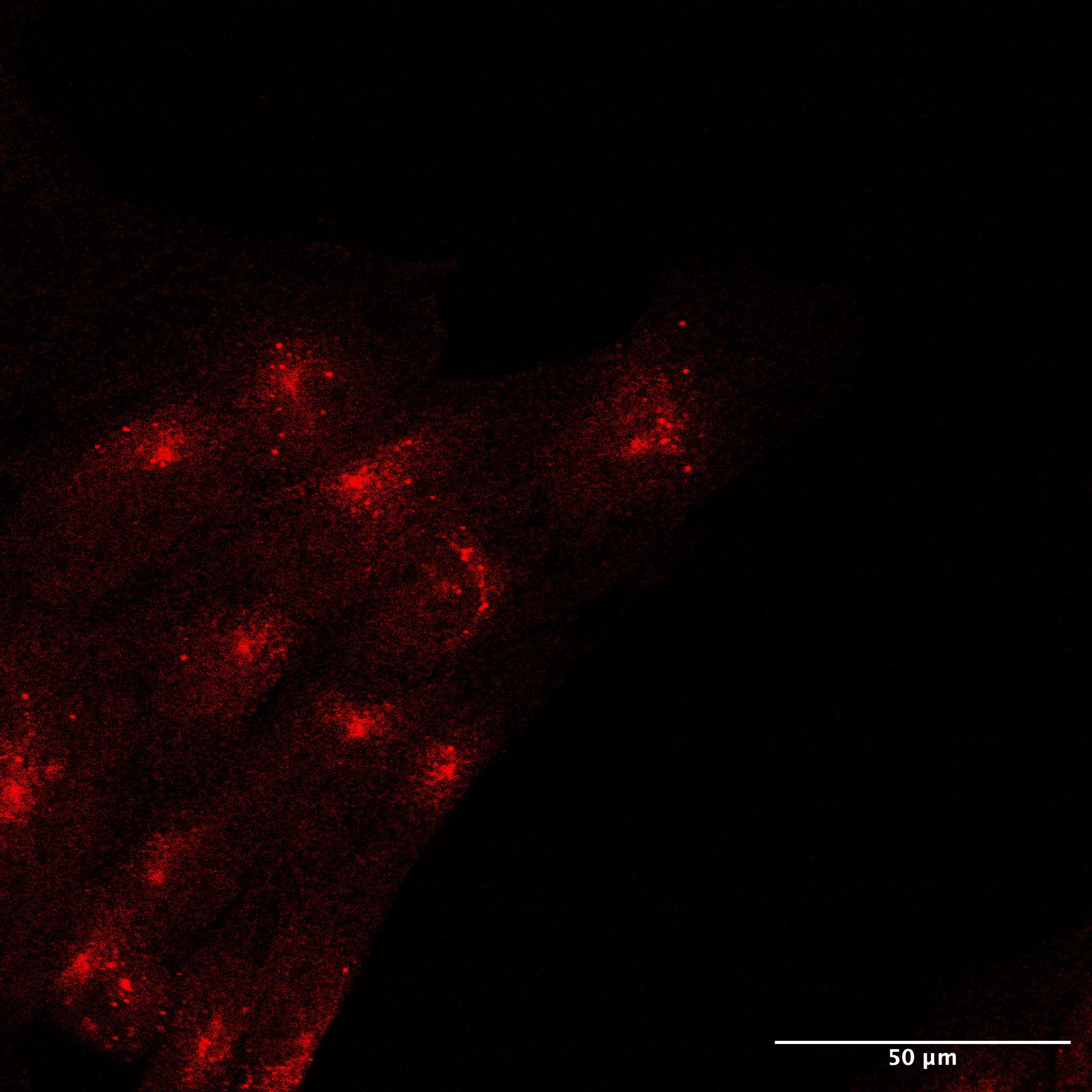

Supplement: Supplementary file 7 — Source data Fig. 5 [file 44318_2025_570_MOESM7_ESM.zip › Fig5/Images/C/Fig_5_panel_c_Sec24C_Golgin97_BFA_30min_1_red.jpg]

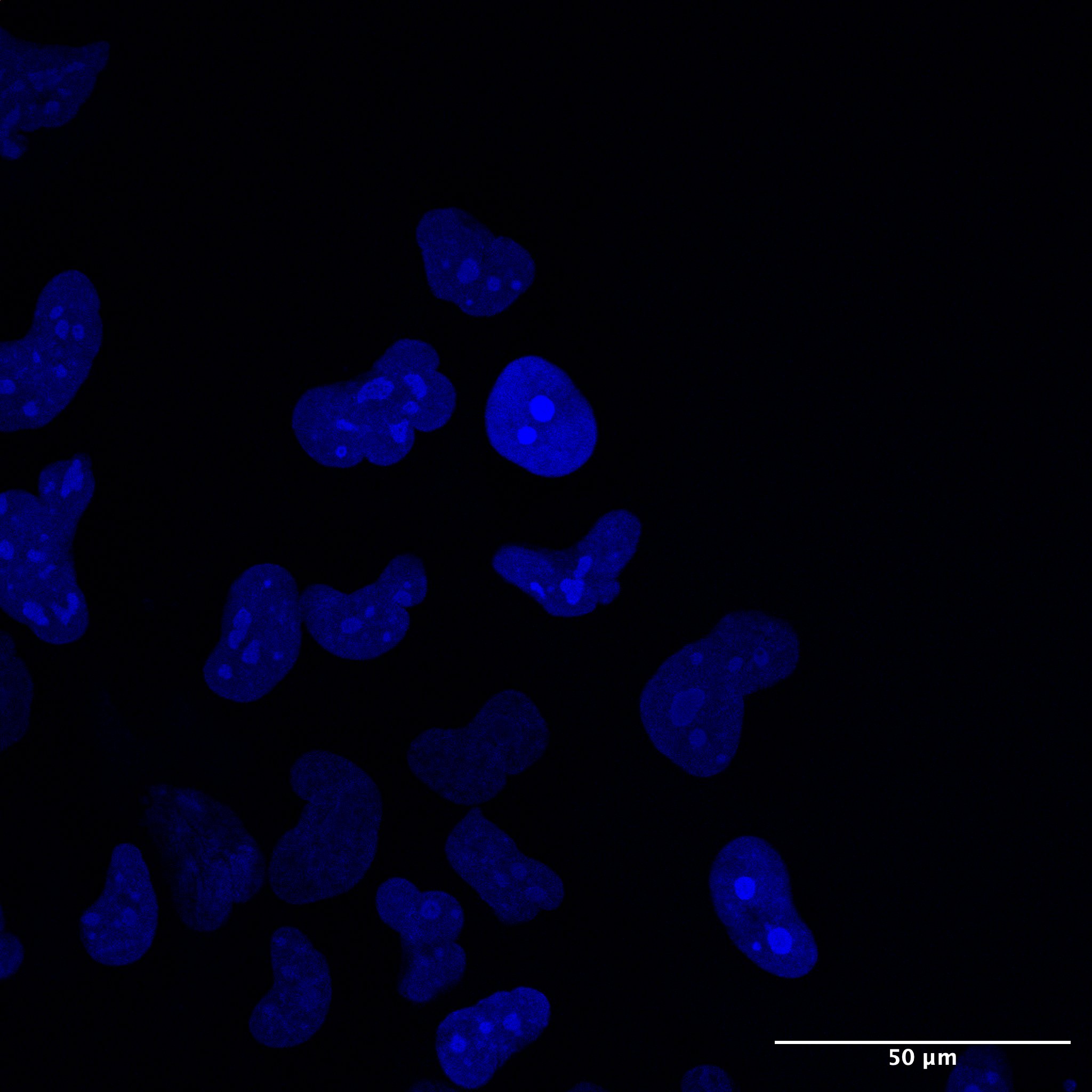

Supplement: Supplementary file 7 — Source data Fig. 5 [file 44318_2025_570_MOESM7_ESM.zip › Fig5/Images/C/Fig_5_panel_c_Sec24C_Golgin_sh#1_3_blue.jpg]

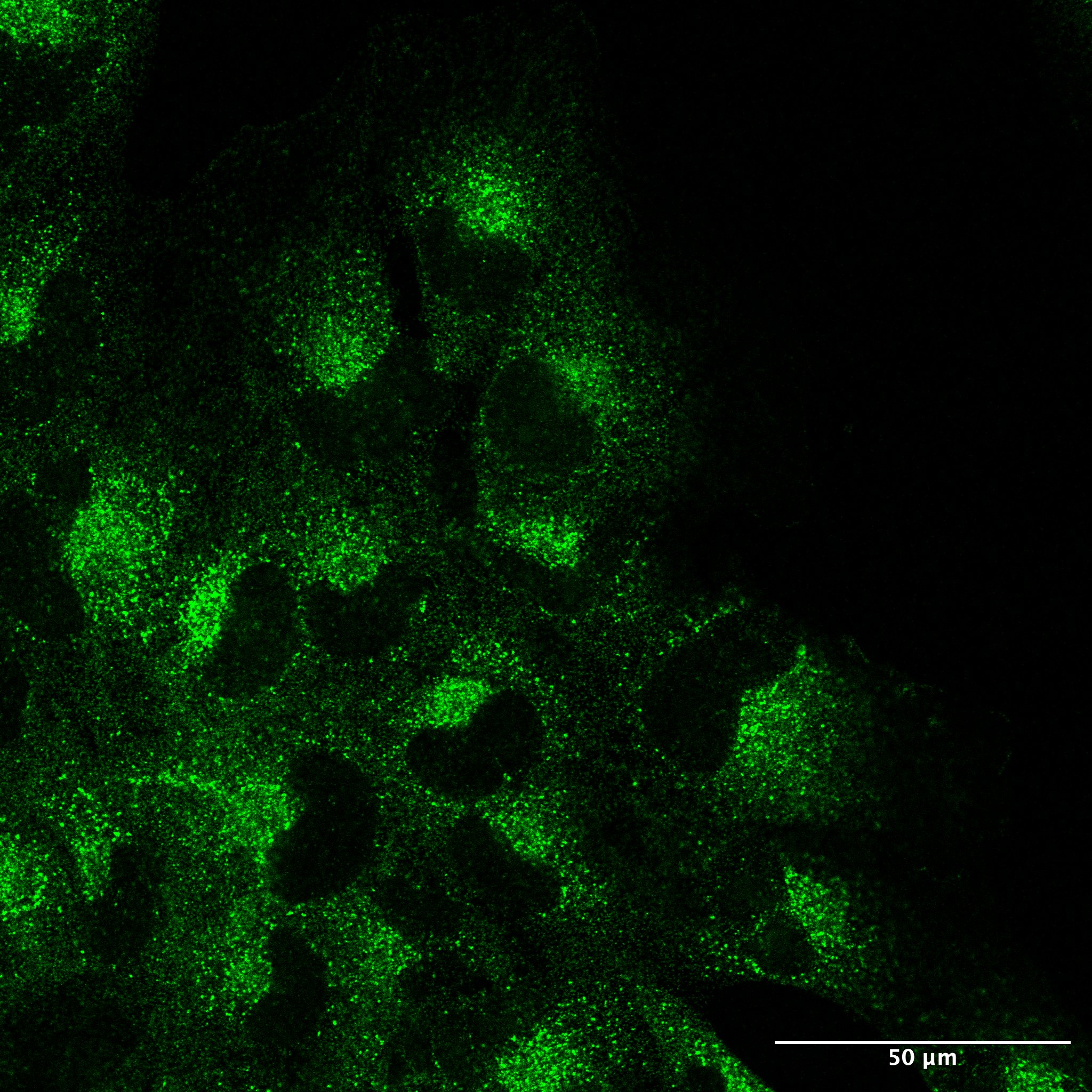

Supplement: Supplementary file 7 — Source data Fig. 5 [file 44318_2025_570_MOESM7_ESM.zip › Fig5/Images/C/Fig_5_panel_c_Sec24C_Golgin_sh#1_3_green.jpg]

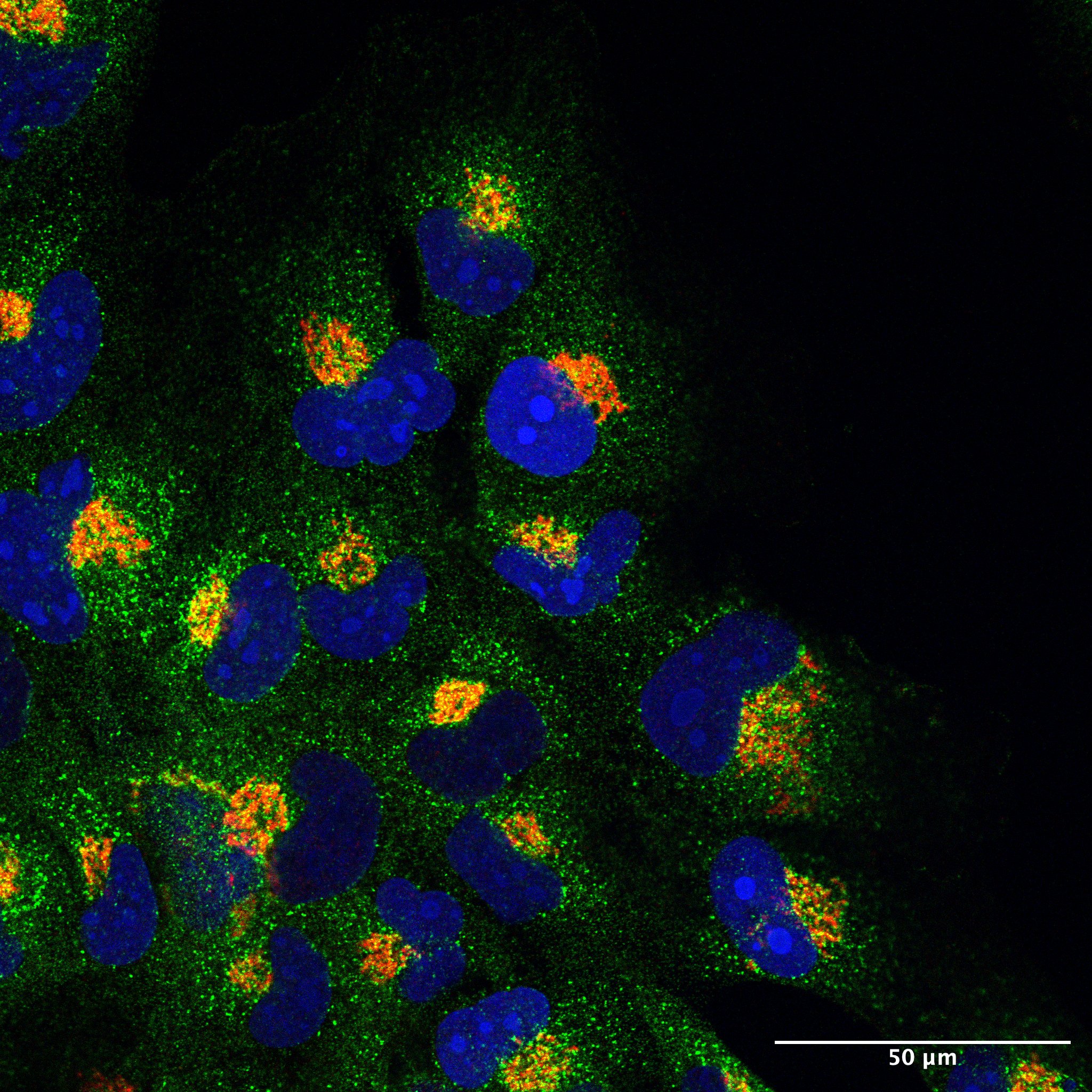

Supplement: Supplementary file 7 — Source data Fig. 5 [file 44318_2025_570_MOESM7_ESM.zip › Fig5/Images/C/Fig_5_panel_c_Sec24C_Golgin_sh#1_3_merge.jpg]

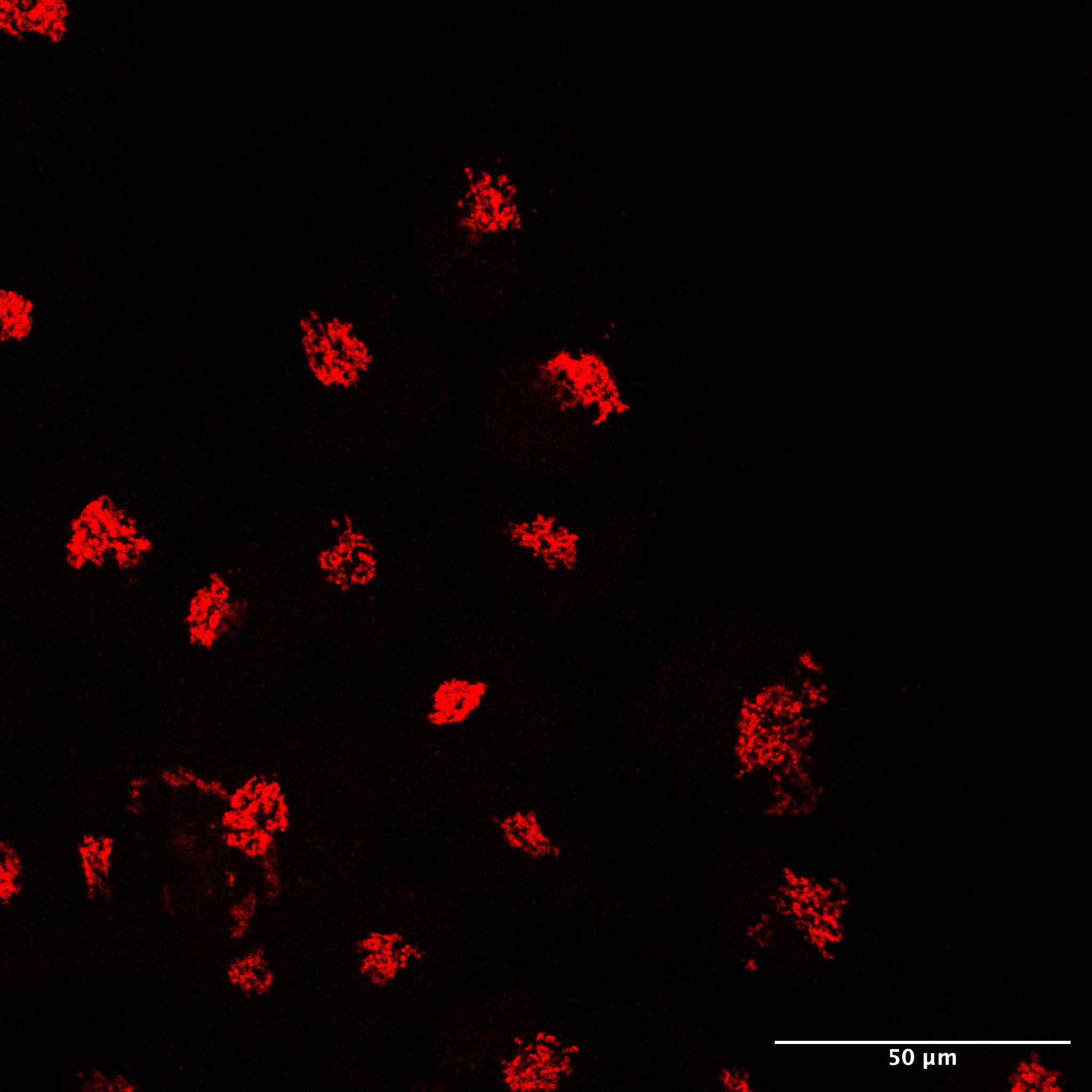

Supplement: Supplementary file 7 — Source data Fig. 5 [file 44318_2025_570_MOESM7_ESM.zip › Fig5/Images/C/Fig_5_panel_c_Sec24C_Golgin_sh#1_3_red.jpg]

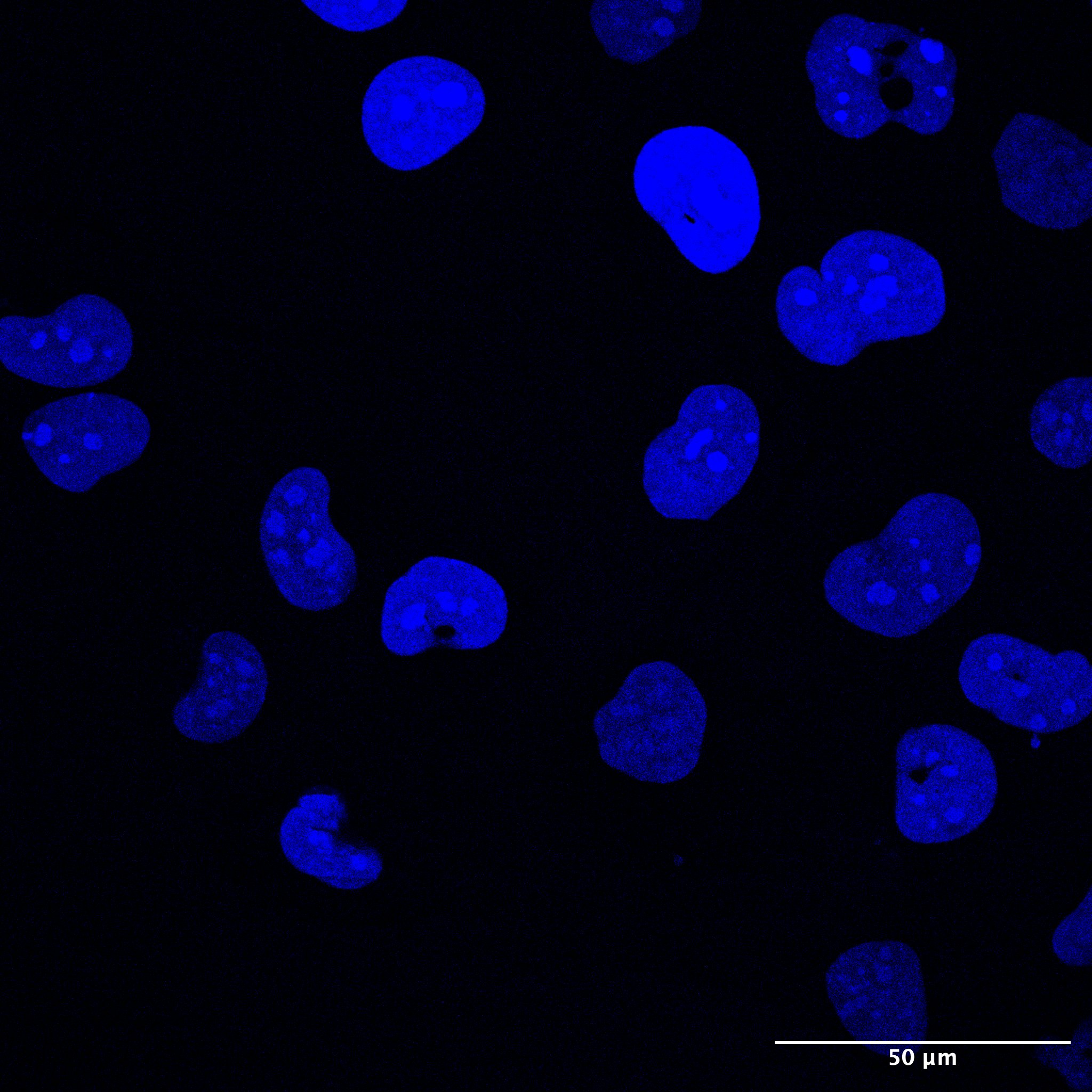

Supplement: Supplementary file 7 — Source data Fig. 5 [file 44318_2025_570_MOESM7_ESM.zip › Fig5/Images/C/Fig_5_panel_c_Sec24C_Golgin_sh#5_2_blue.jpg]

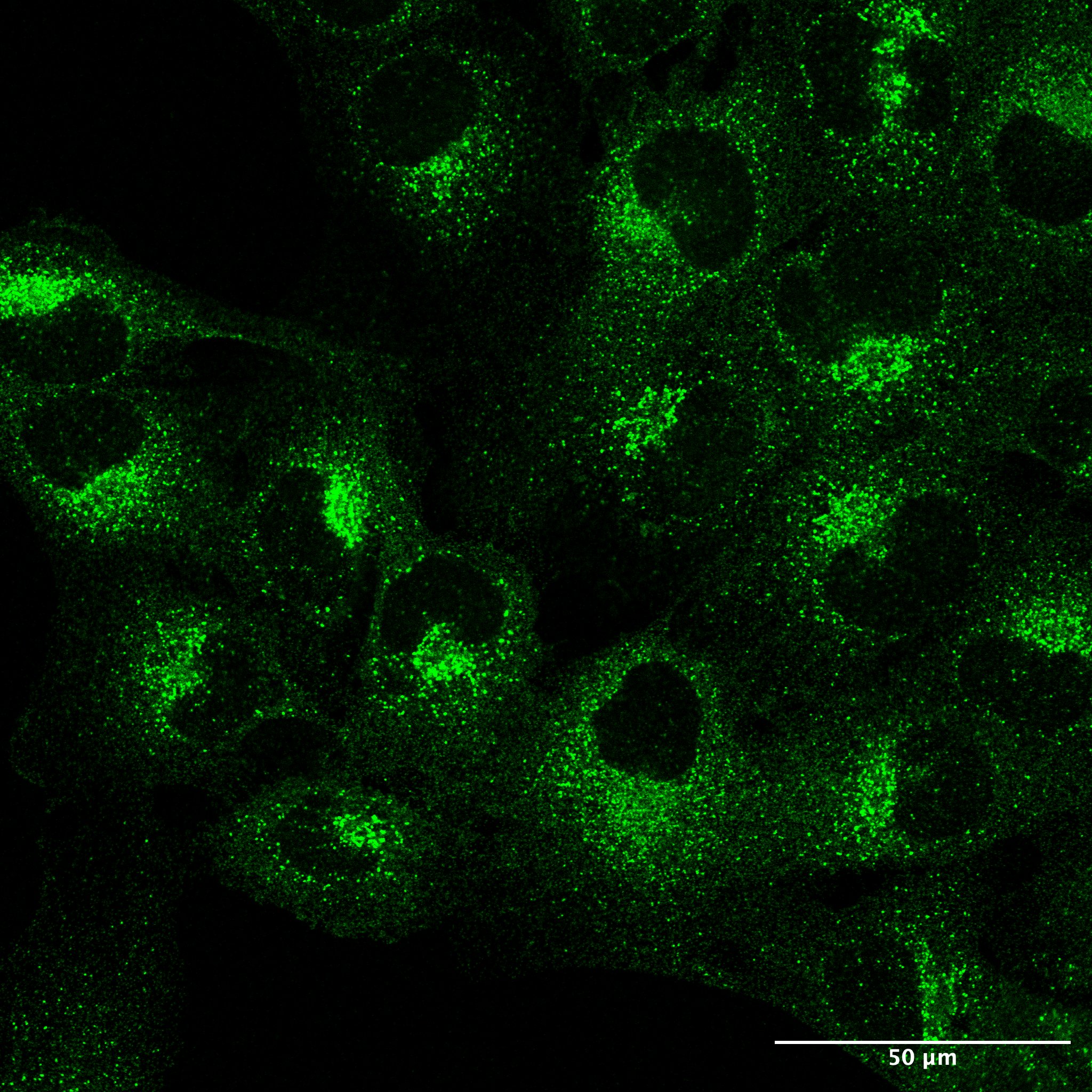

Supplement: Supplementary file 7 — Source data Fig. 5 [file 44318_2025_570_MOESM7_ESM.zip › Fig5/Images/C/Fig_5_panel_c_Sec24C_Golgin_sh#5_2_green.jpg]

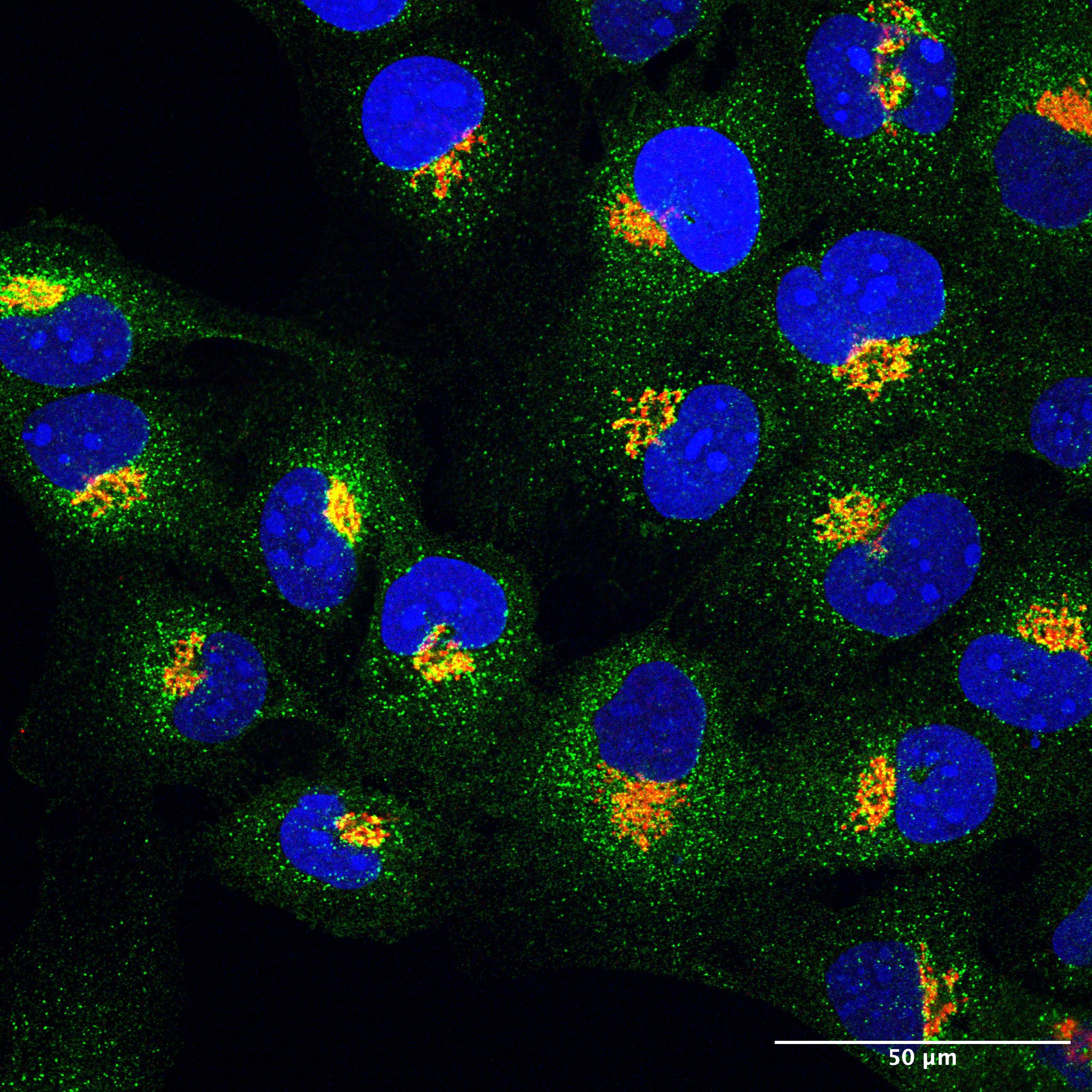

Supplement: Supplementary file 7 — Source data Fig. 5 [file 44318_2025_570_MOESM7_ESM.zip › Fig5/Images/C/Fig_5_panel_c_Sec24C_Golgin_sh#5_2_merge.jpg]

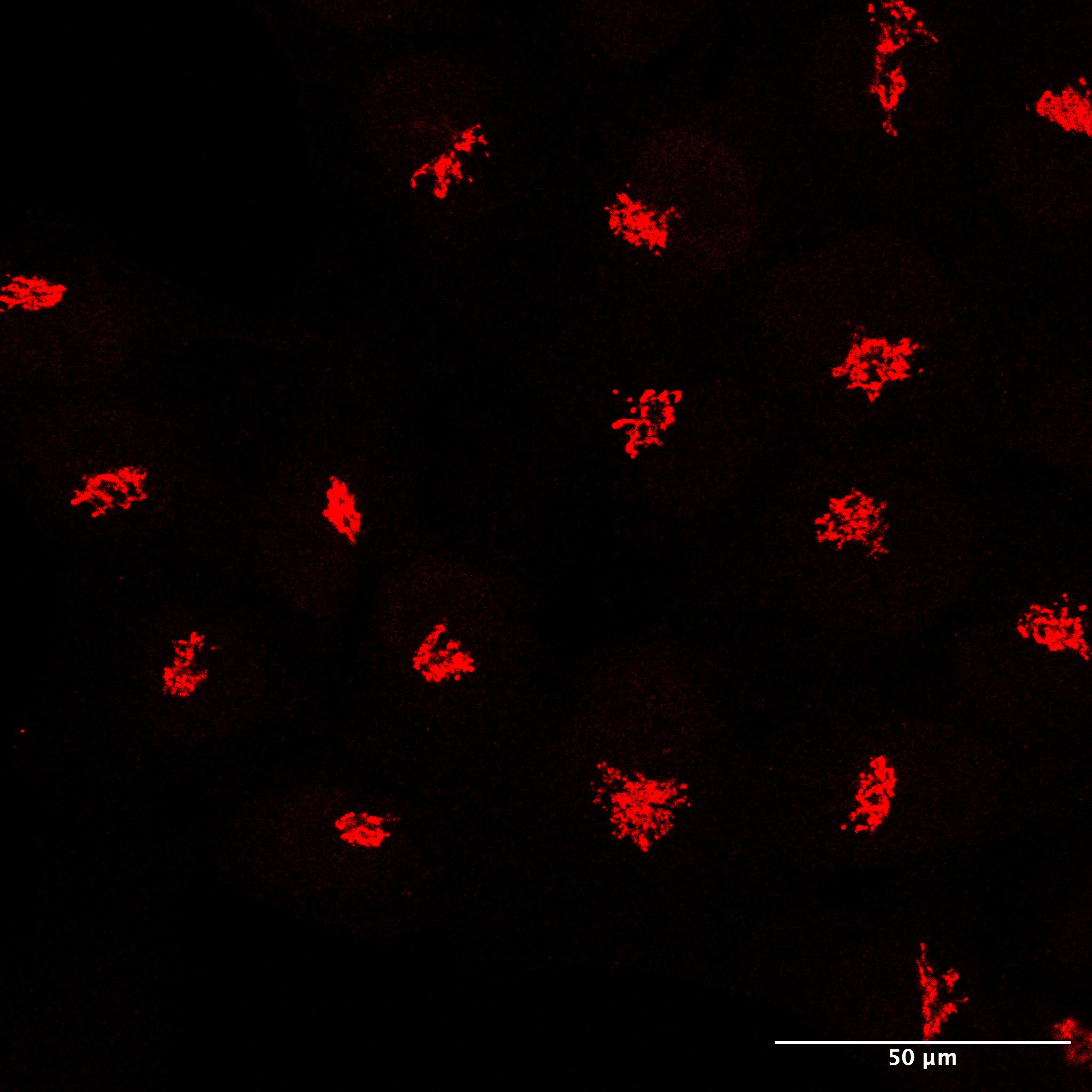

Supplement: Supplementary file 7 — Source data Fig. 5 [file 44318_2025_570_MOESM7_ESM.zip › Fig5/Images/C/Fig_5_panel_c_Sec24C_Golgin_sh#5_2_red.jpg]

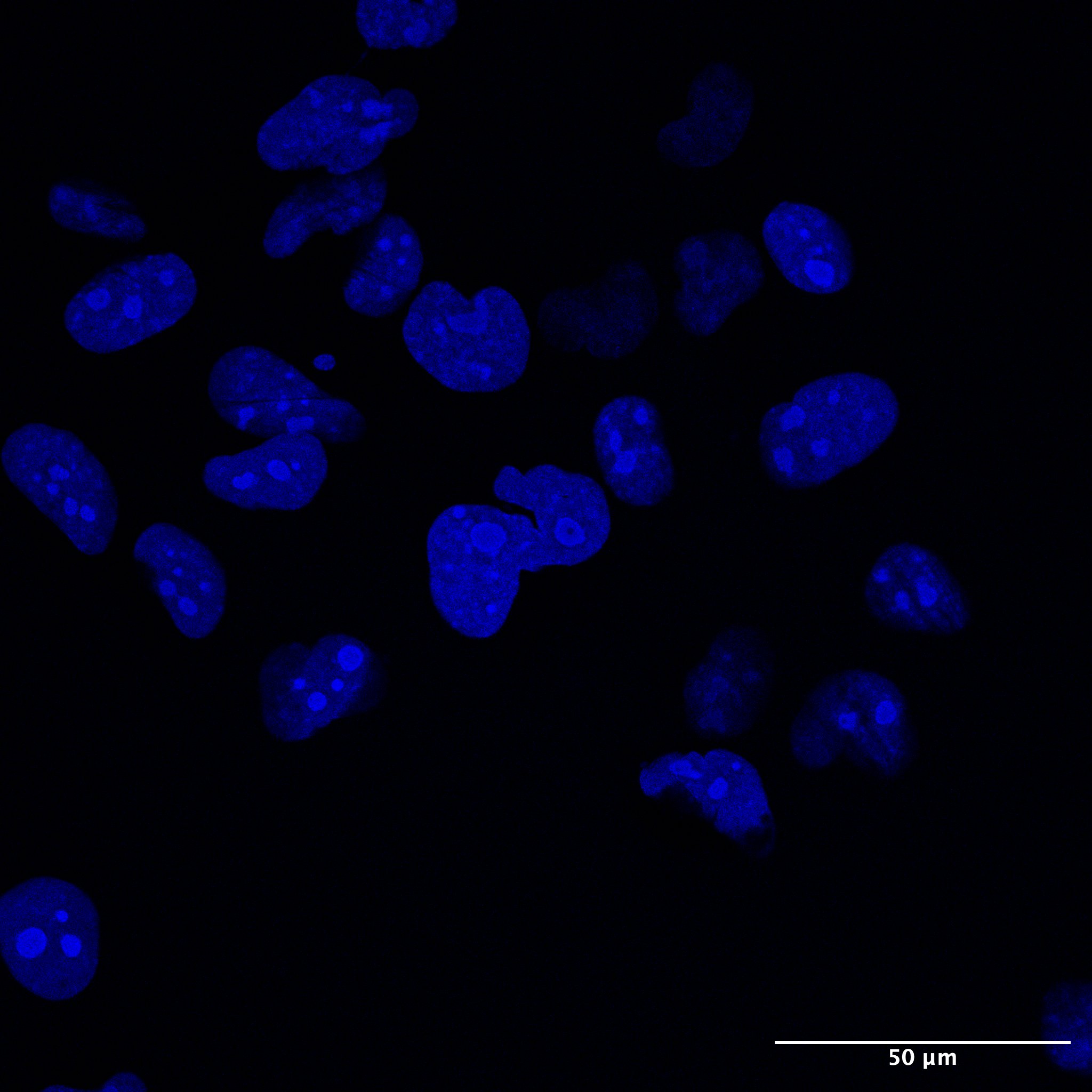

Supplement: Supplementary file 7 — Source data Fig. 5 [file 44318_2025_570_MOESM7_ESM.zip › Fig5/Images/C/Fig_5_panel_c_Sec24C_Golgin_shNT_5_blue.jpg]

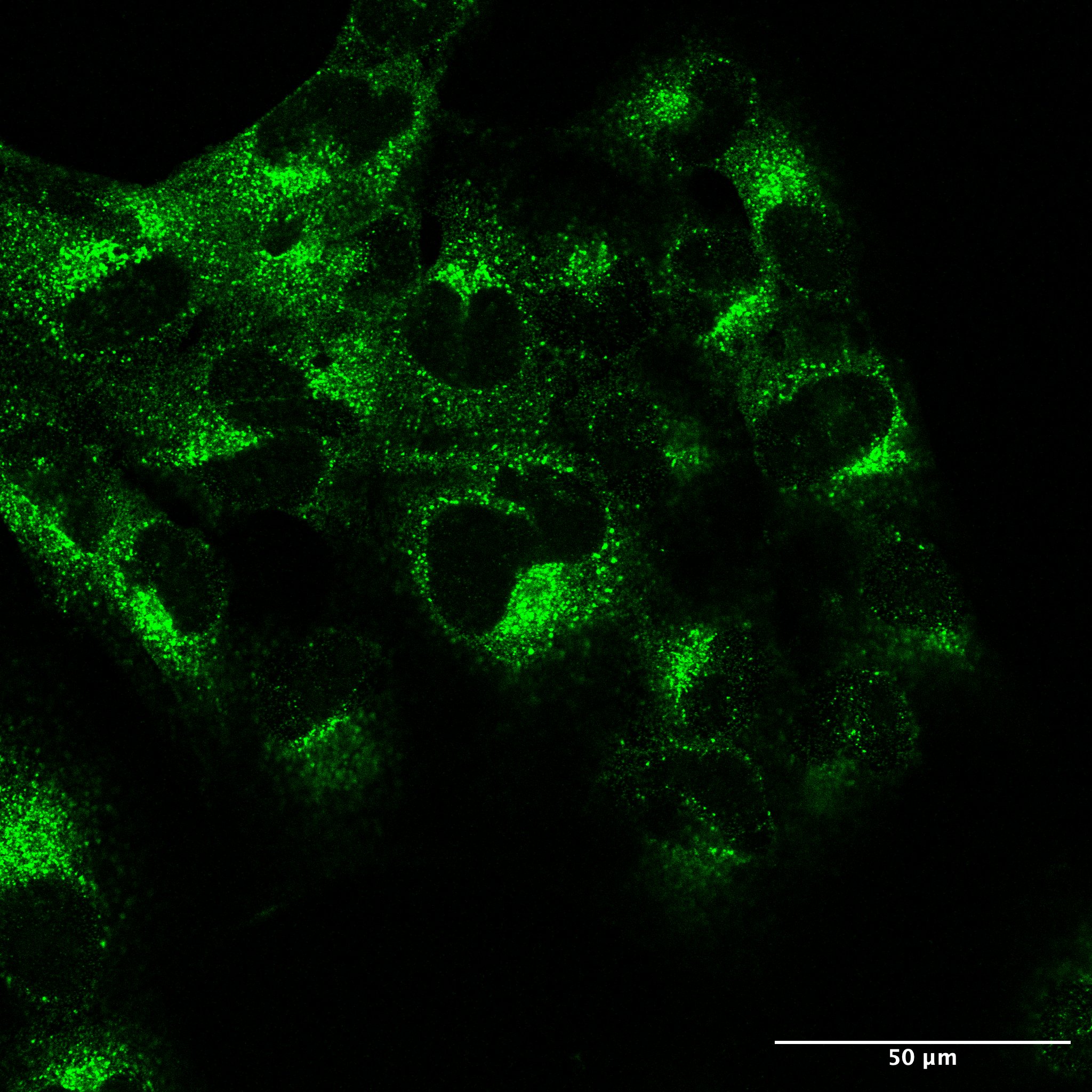

Supplement: Supplementary file 7 — Source data Fig. 5 [file 44318_2025_570_MOESM7_ESM.zip › Fig5/Images/C/Fig_5_panel_c_Sec24C_Golgin_shNT_5_green.jpg]

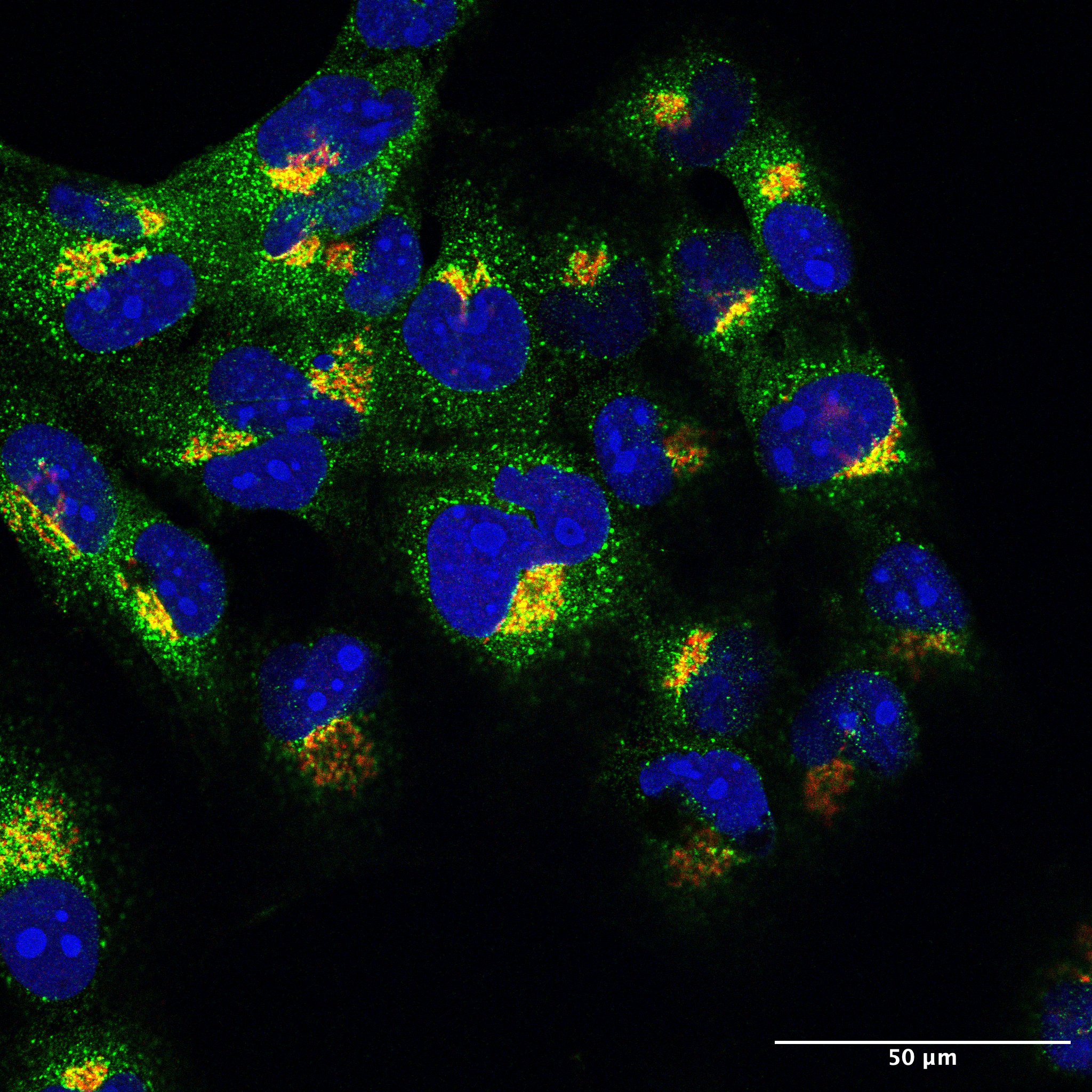

Supplement: Supplementary file 7 — Source data Fig. 5 [file 44318_2025_570_MOESM7_ESM.zip › Fig5/Images/C/Fig_5_panel_c_Sec24C_Golgin_shNT_5_merge.jpg]

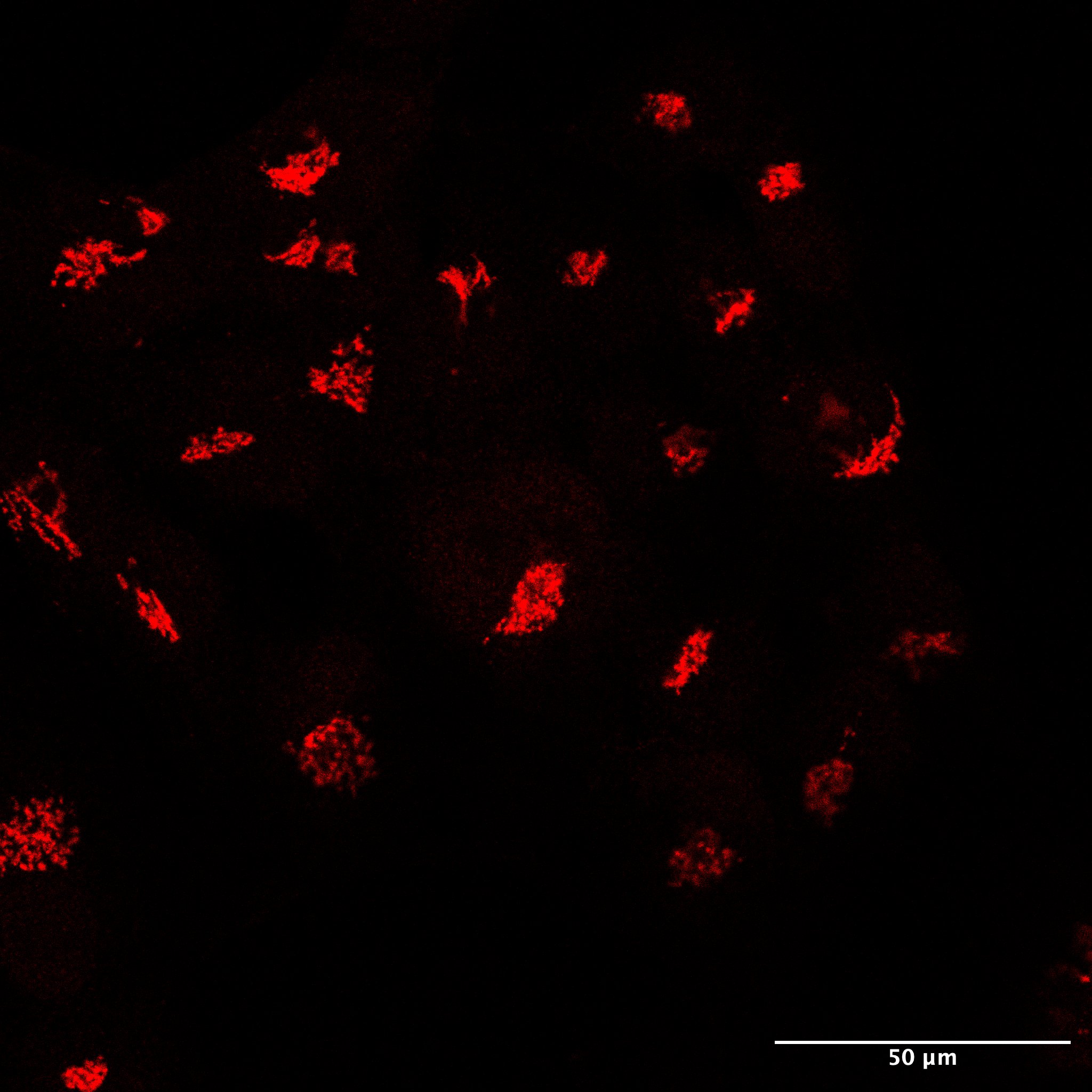

Supplement: Supplementary file 7 — Source data Fig. 5 [file 44318_2025_570_MOESM7_ESM.zip › Fig5/Images/C/Fig_5_panel_c_Sec24C_Golgin_shNT_5_red.jpg]

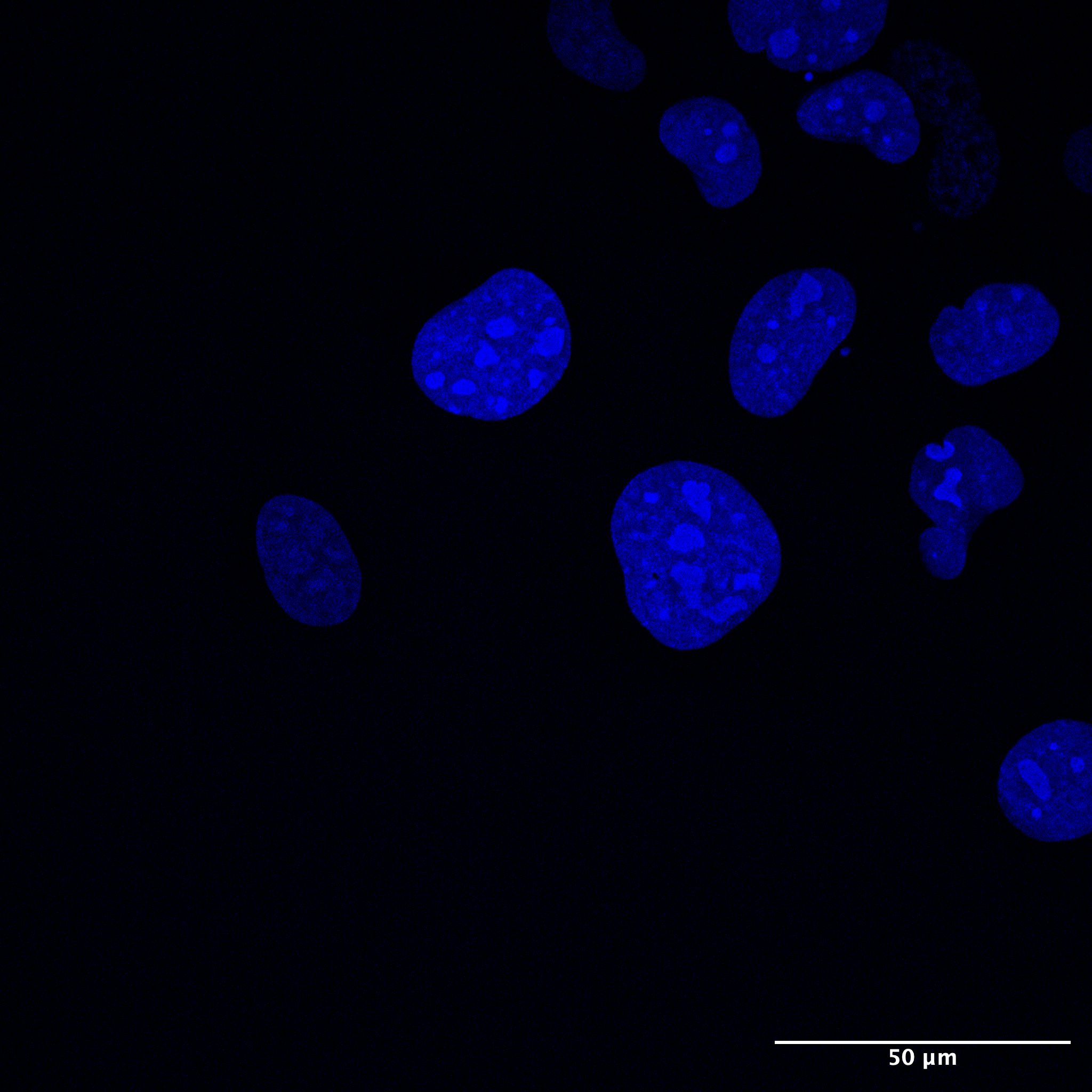

Supplement: Supplementary file 7 — Source data Fig. 5 [file 44318_2025_570_MOESM7_ESM.zip › Fig5/Images/D/Fig_5_panel_d_Sec24C_MYOF_3_blue.jpg]

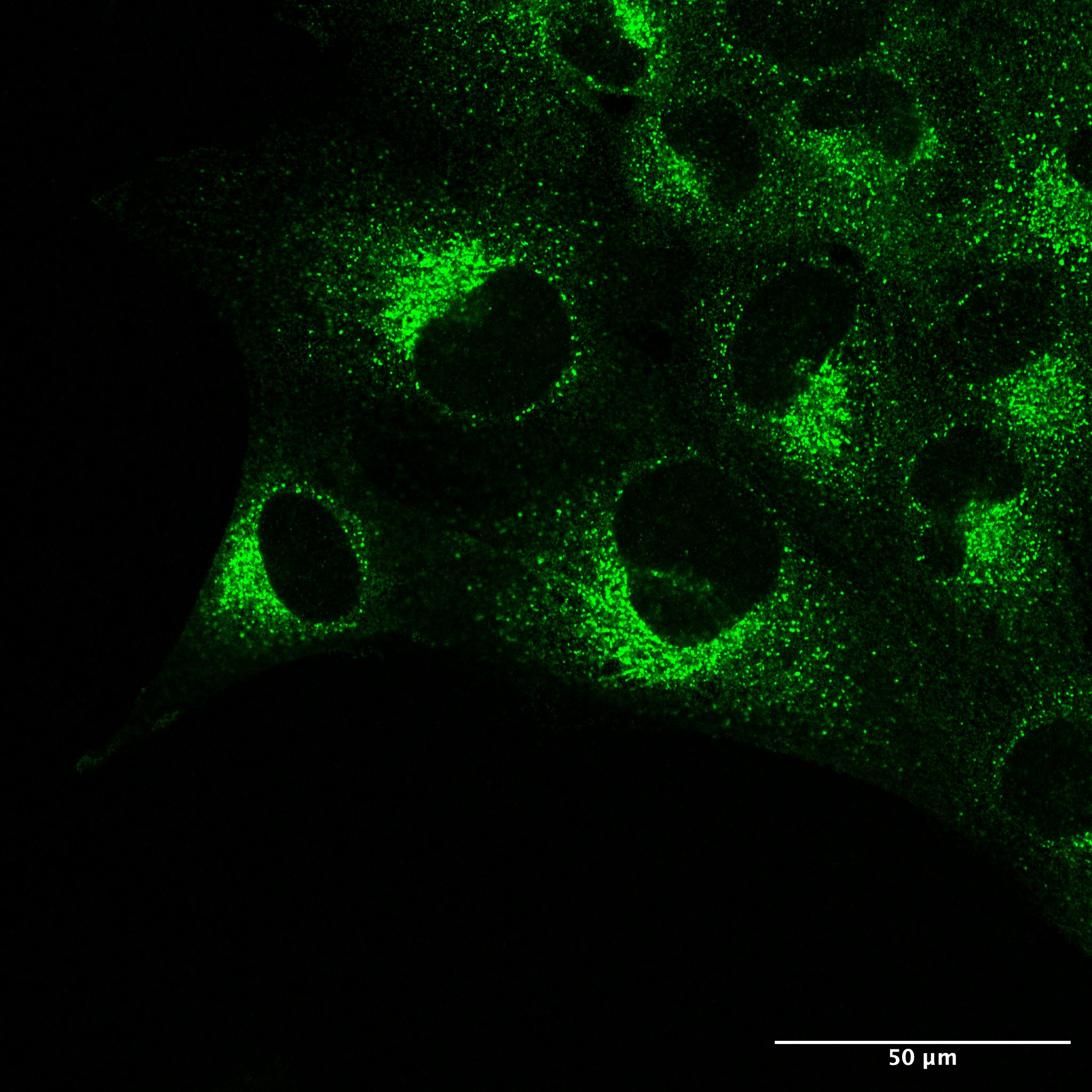

Supplement: Supplementary file 7 — Source data Fig. 5 [file 44318_2025_570_MOESM7_ESM.zip › Fig5/Images/D/Fig_5_panel_d_Sec24C_MYOF_3_green.jpg]

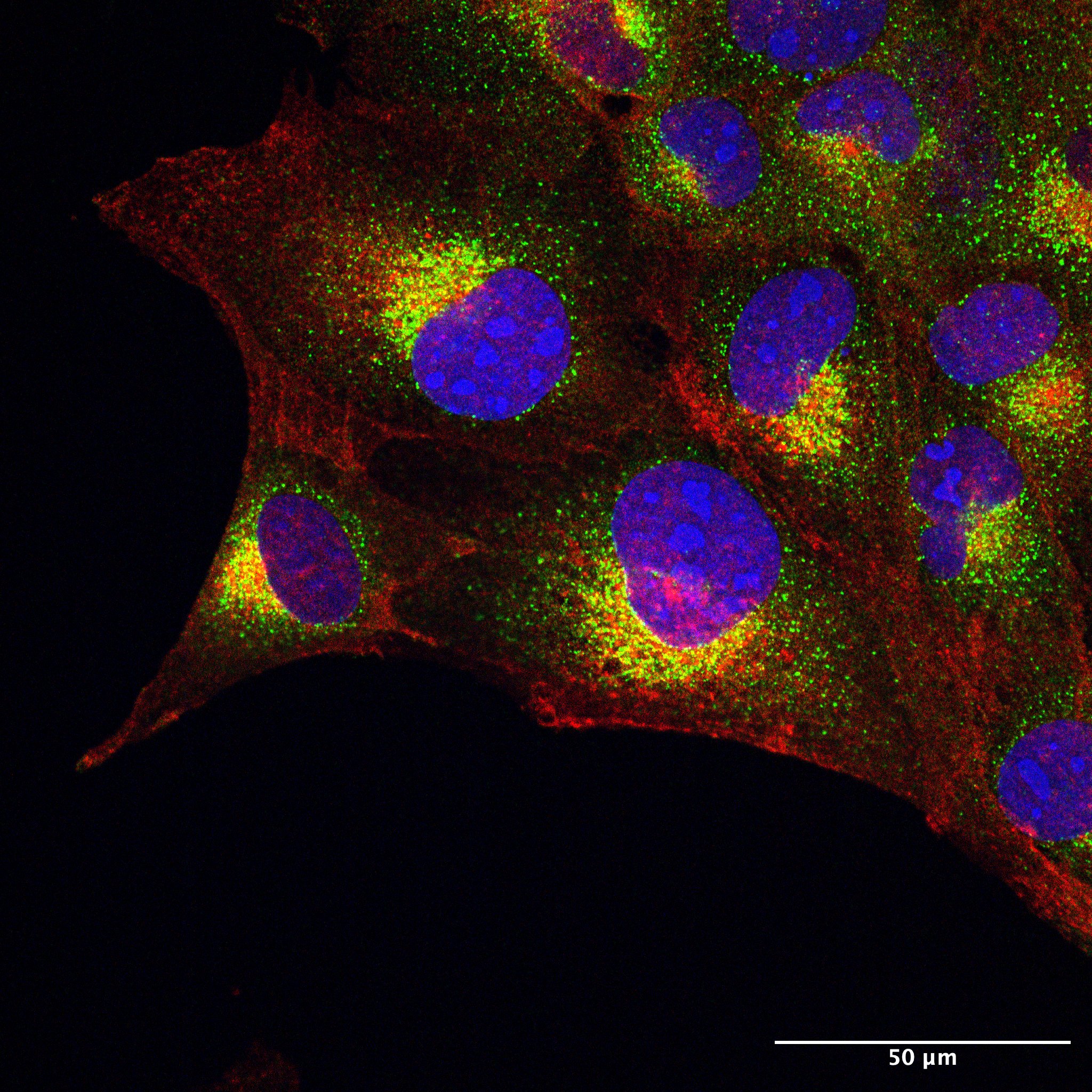

Supplement: Supplementary file 7 — Source data Fig. 5 [file 44318_2025_570_MOESM7_ESM.zip › Fig5/Images/D/Fig_5_panel_d_Sec24C_MYOF_3_merge.jpg]

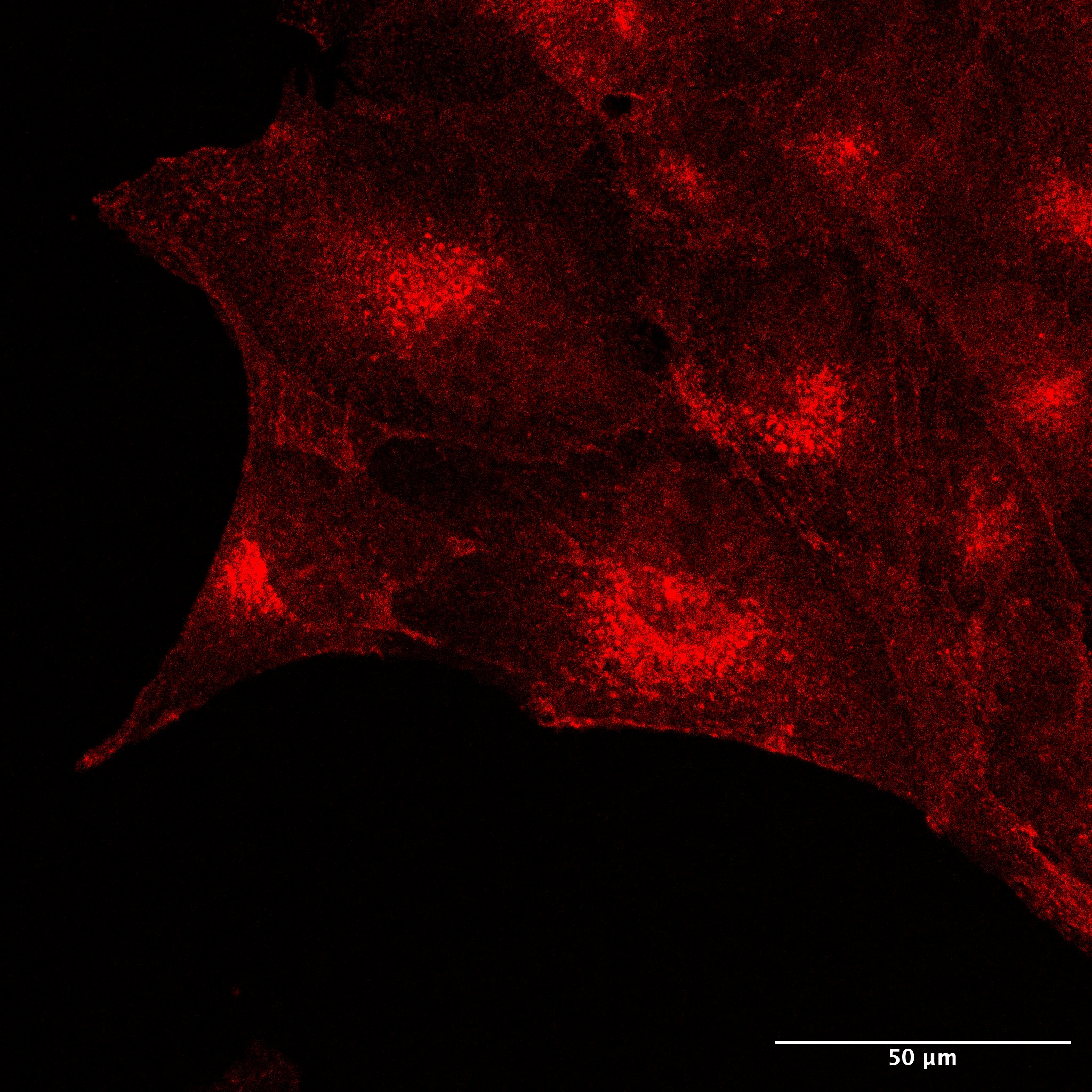

Supplement: Supplementary file 7 — Source data Fig. 5 [file 44318_2025_570_MOESM7_ESM.zip › Fig5/Images/D/Fig_5_panel_d_Sec24C_MYOF_3_red.jpg]

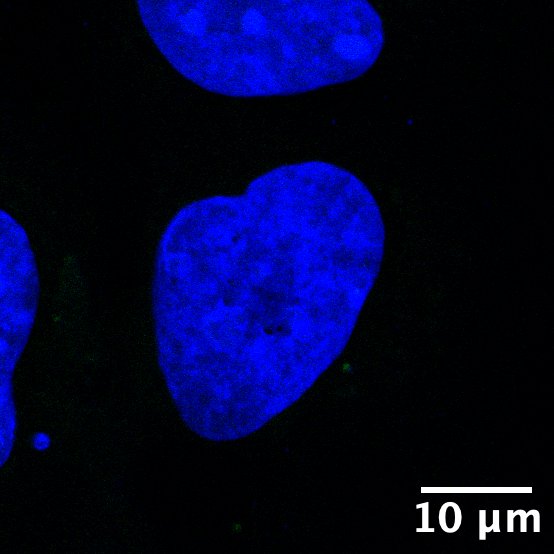

Supplement: Supplementary file 7 — Source data Fig. 5 [file 44318_2025_570_MOESM7_ESM.zip › Fig5/Images/E/Fig_5_panel_e_MYOF_Sec24C_CTRL_2.jpg]

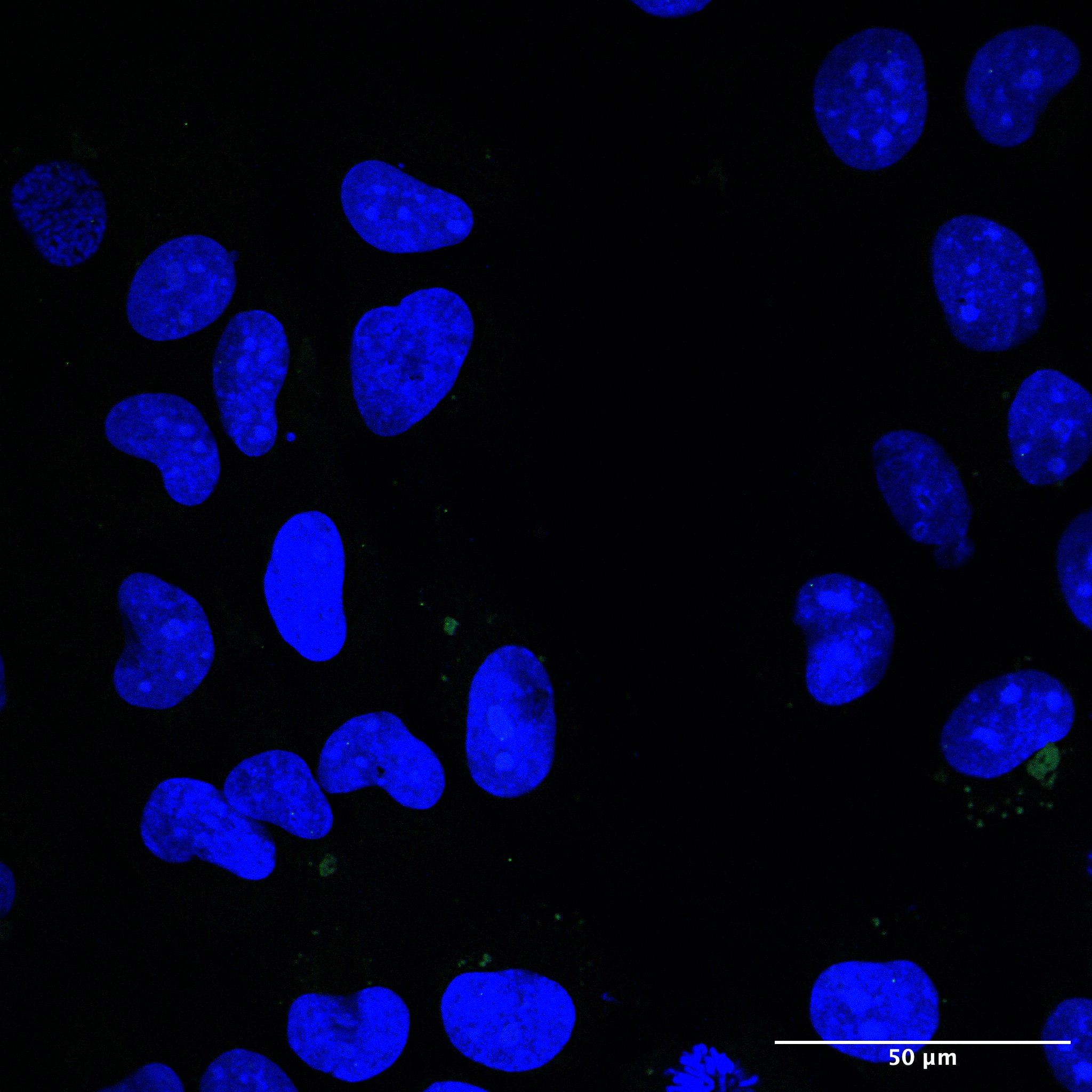

Supplement: Supplementary file 7 — Source data Fig. 5 [file 44318_2025_570_MOESM7_ESM.zip › Fig5/Images/E/Fig_5_panel_e_MYOF_Sec24C_CTRL_2_big.jpg]

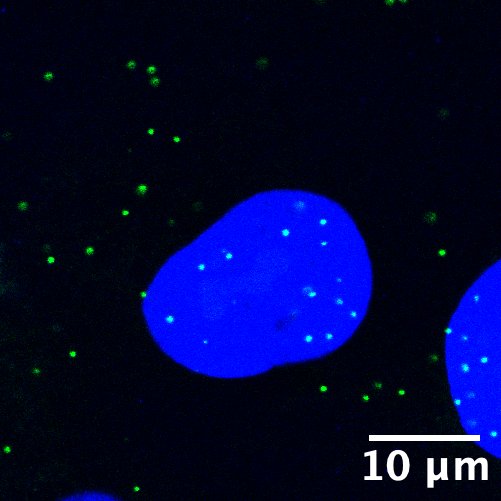

Supplement: Supplementary file 7 — Source data Fig. 5 [file 44318_2025_570_MOESM7_ESM.zip › Fig5/Images/E/Fig_5_panel_e_MYOF_Sec24C_sh#1_5.jpg]

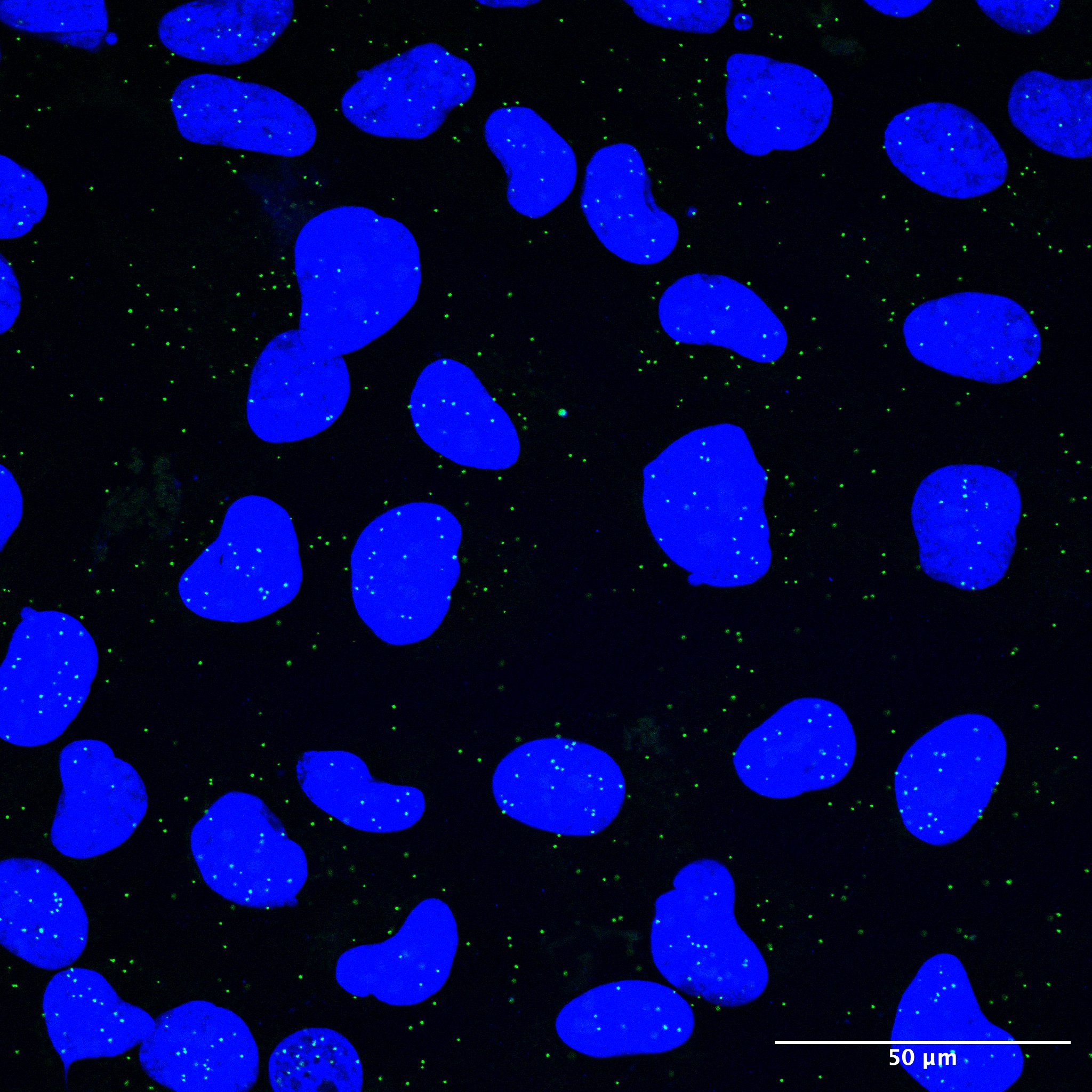

Supplement: Supplementary file 7 — Source data Fig. 5 [file 44318_2025_570_MOESM7_ESM.zip › Fig5/Images/E/Fig_5_panel_e_MYOF_Sec24C_sh#1_5_big.jpg]

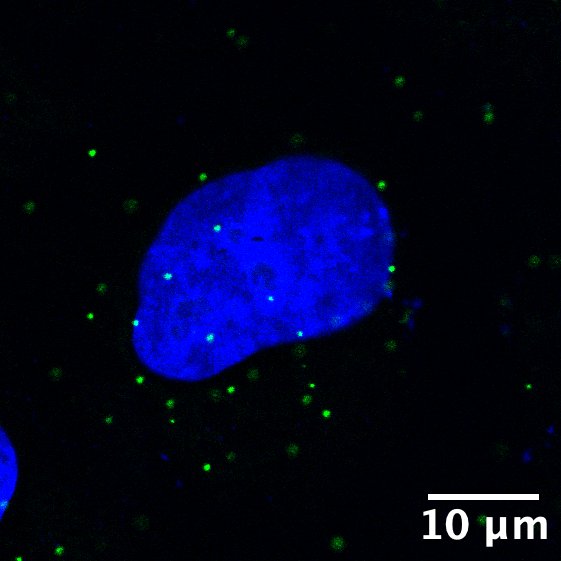

Supplement: Supplementary file 7 — Source data Fig. 5 [file 44318_2025_570_MOESM7_ESM.zip › Fig5/Images/E/Fig_5_panel_e_MYOF_Sec24C_sh#5_3.jpg]

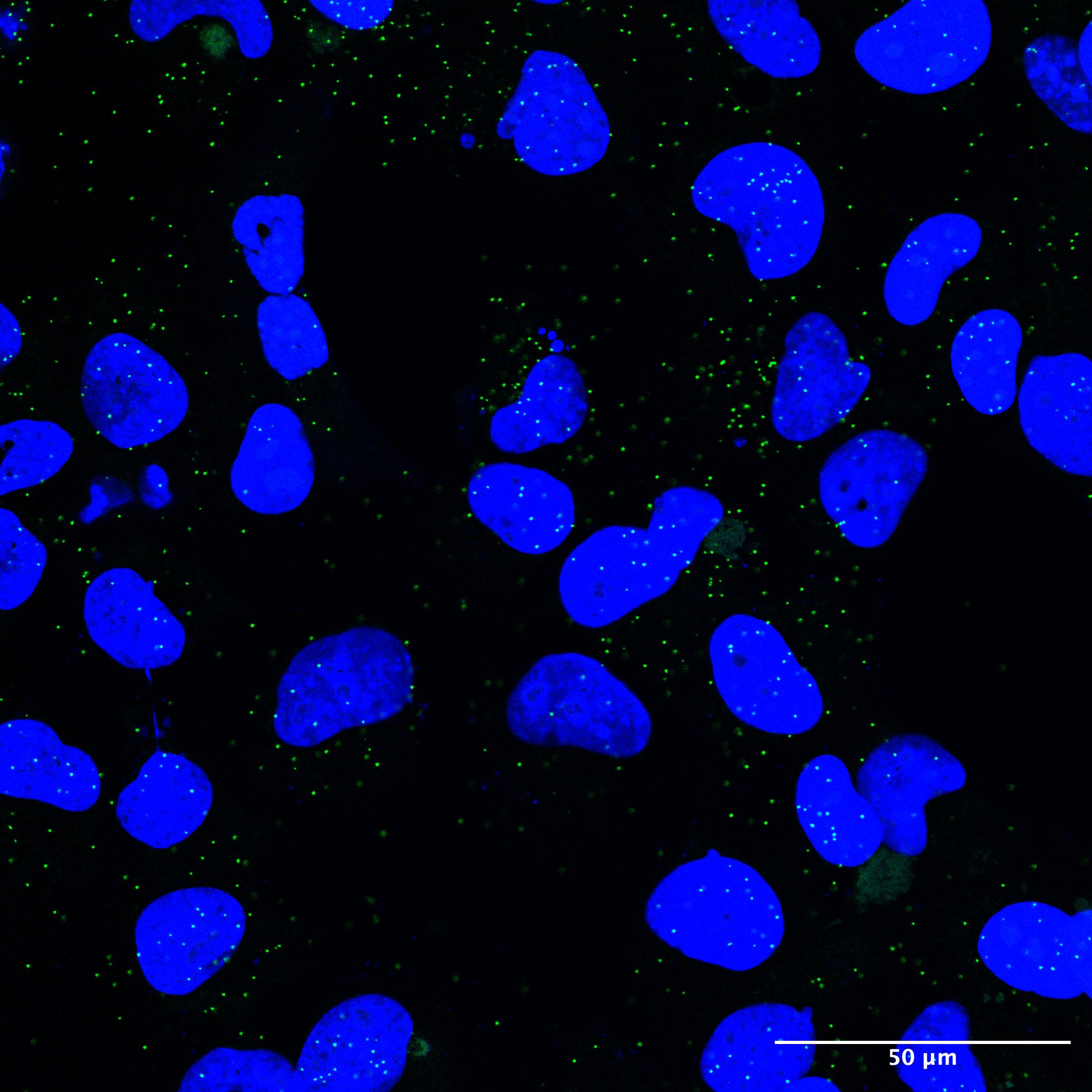

Supplement: Supplementary file 7 — Source data Fig. 5 [file 44318_2025_570_MOESM7_ESM.zip › Fig5/Images/E/Fig_5_panel_e_MYOF_Sec24C_sh#5_3_big.jpg]

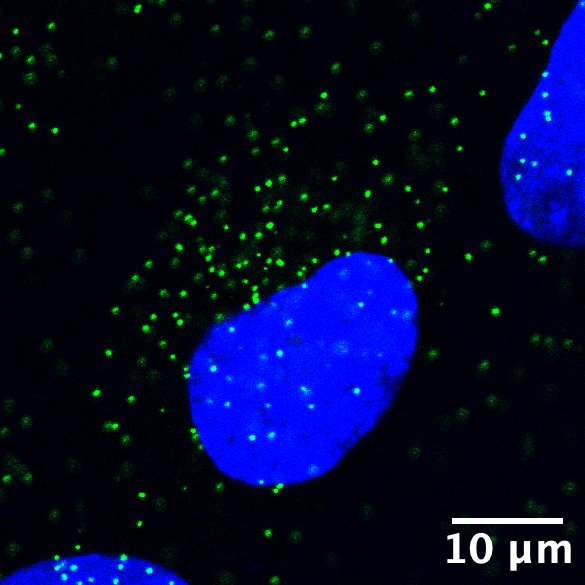

Supplement: Supplementary file 7 — Source data Fig. 5 [file 44318_2025_570_MOESM7_ESM.zip › Fig5/Images/E/Fig_5_panel_e_MYOF_Sec24C_shNT_3.jpg]

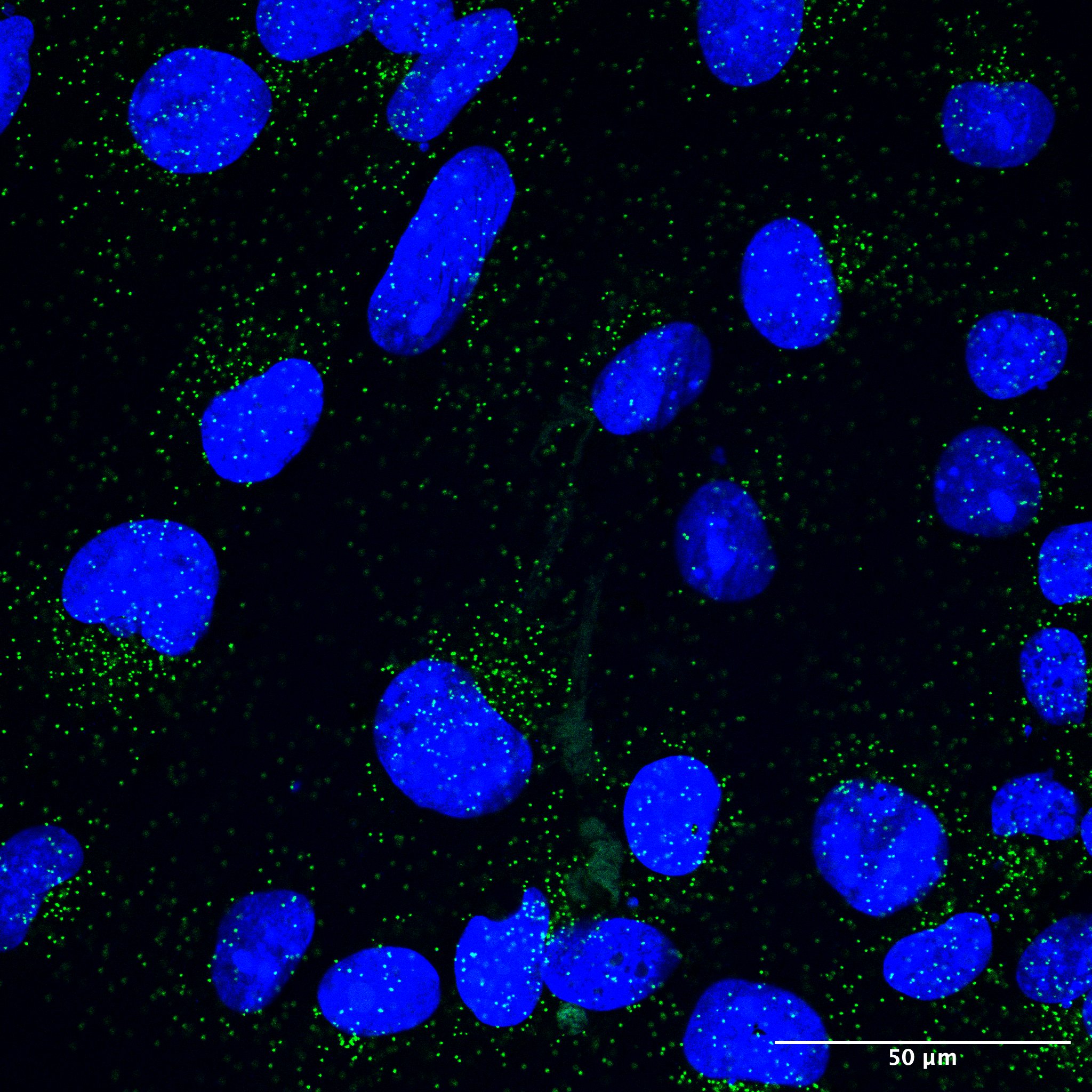

Supplement: Supplementary file 7 — Source data Fig. 5 [file 44318_2025_570_MOESM7_ESM.zip › Fig5/Images/E/Fig_5_panel_e_MYOF_Sec24C_shNT_3_big.jpg]

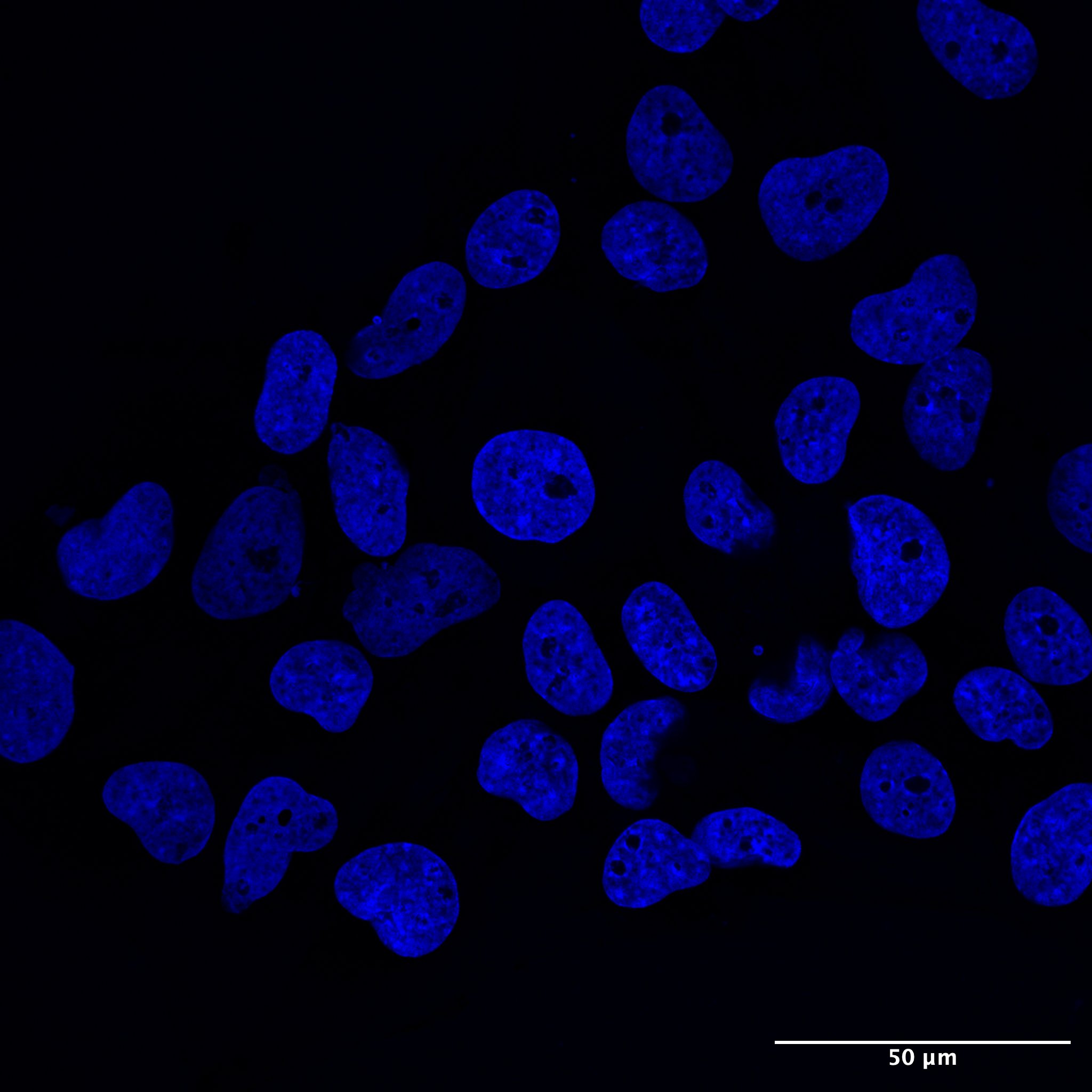

Supplement: Supplementary file 7 — Source data Fig. 5 [file 44318_2025_570_MOESM7_ESM.zip › Fig5/Images/I/Fig_5_panel_i_shNT_R1_MYOF_9_blue.jpg]

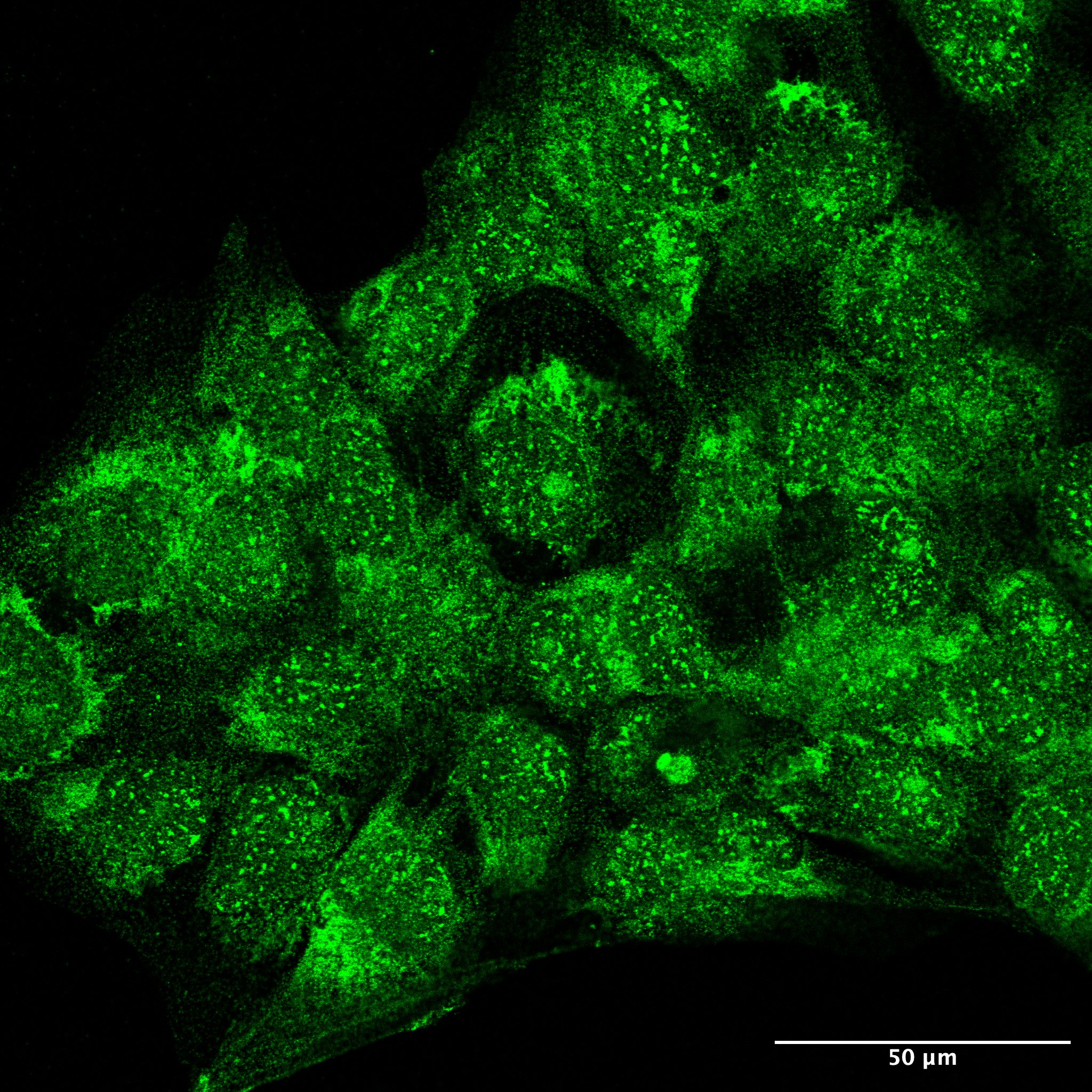

Supplement: Supplementary file 7 — Source data Fig. 5 [file 44318_2025_570_MOESM7_ESM.zip › Fig5/Images/I/Fig_5_panel_i_shNT_R1_MYOF_9_green.jpg]

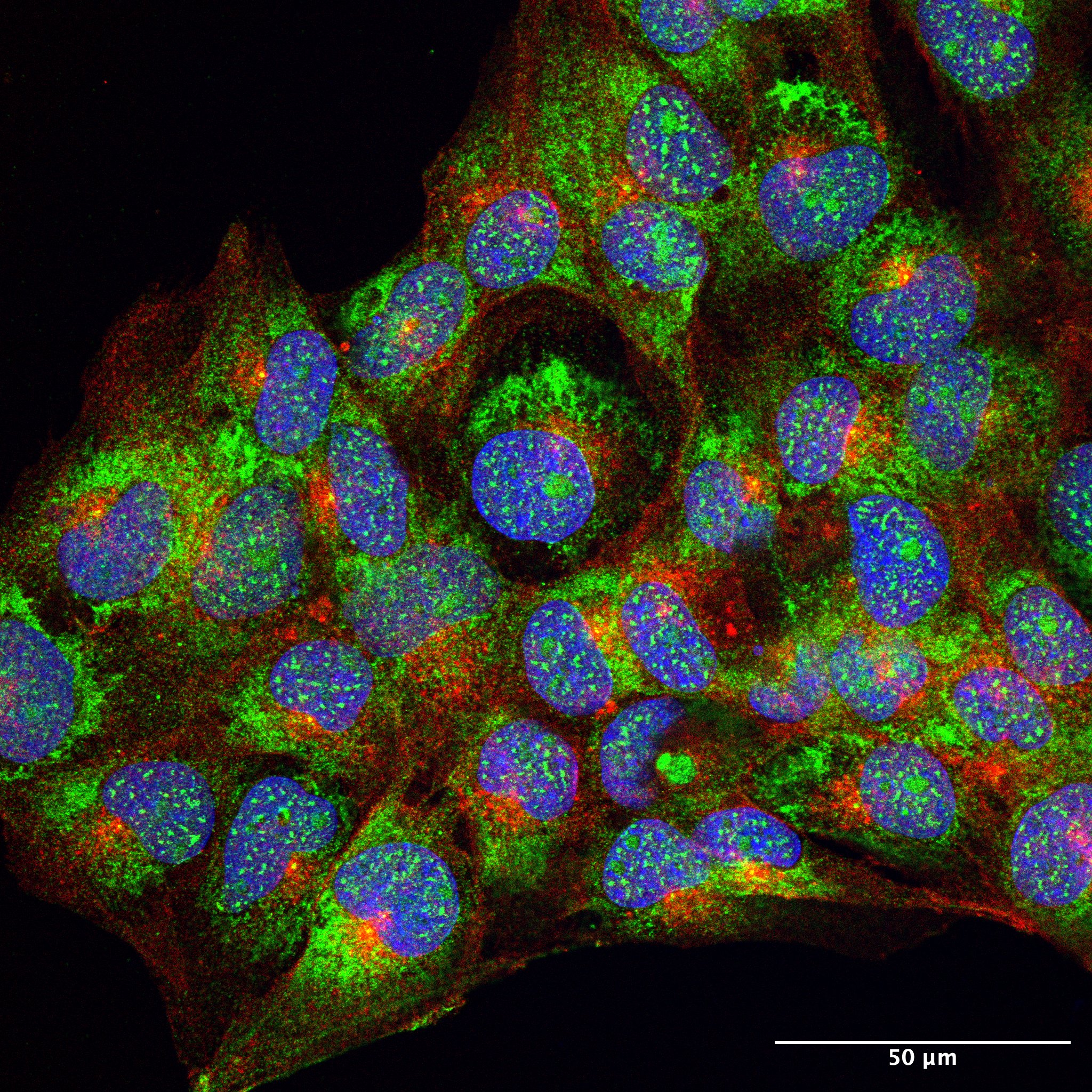

Supplement: Supplementary file 7 — Source data Fig. 5 [file 44318_2025_570_MOESM7_ESM.zip › Fig5/Images/I/Fig_5_panel_i_shNT_R1_MYOF_9_merge.jpg]

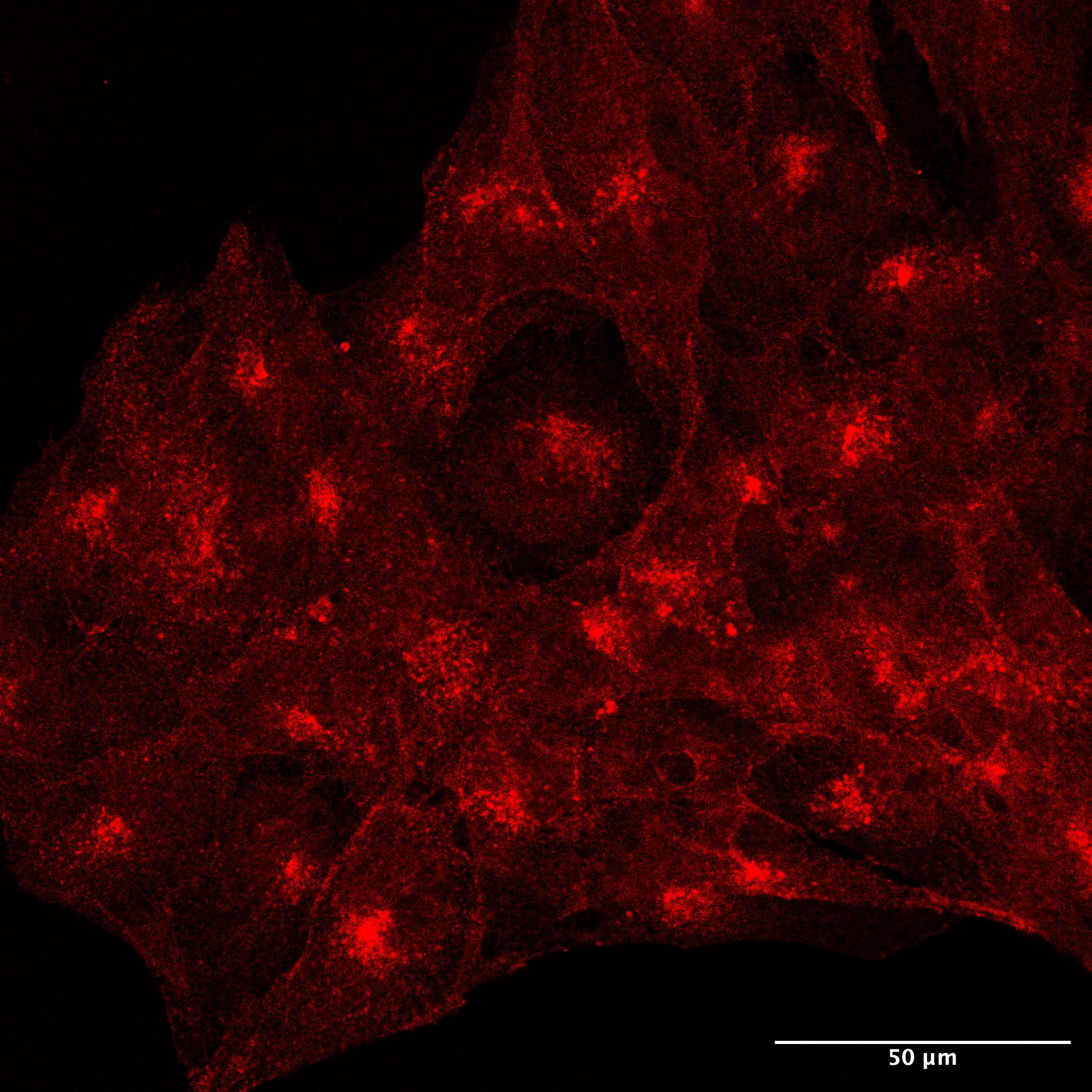

Supplement: Supplementary file 7 — Source data Fig. 5 [file 44318_2025_570_MOESM7_ESM.zip › Fig5/Images/I/Fig_5_panel_i_shNT_R1_MYOF_9_red.jpg]

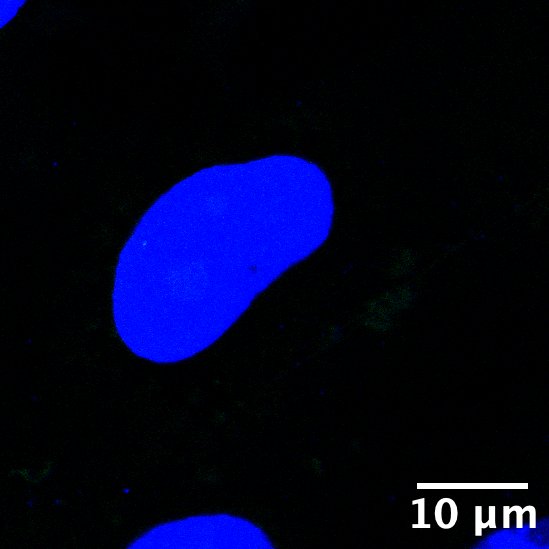

Supplement: Supplementary file 7 — Source data Fig. 5 [file 44318_2025_570_MOESM7_ESM.zip › Fig5/Images/J/Fig_5_panel_j_MYOF_TGFBR1_CTRL_4.jpg]

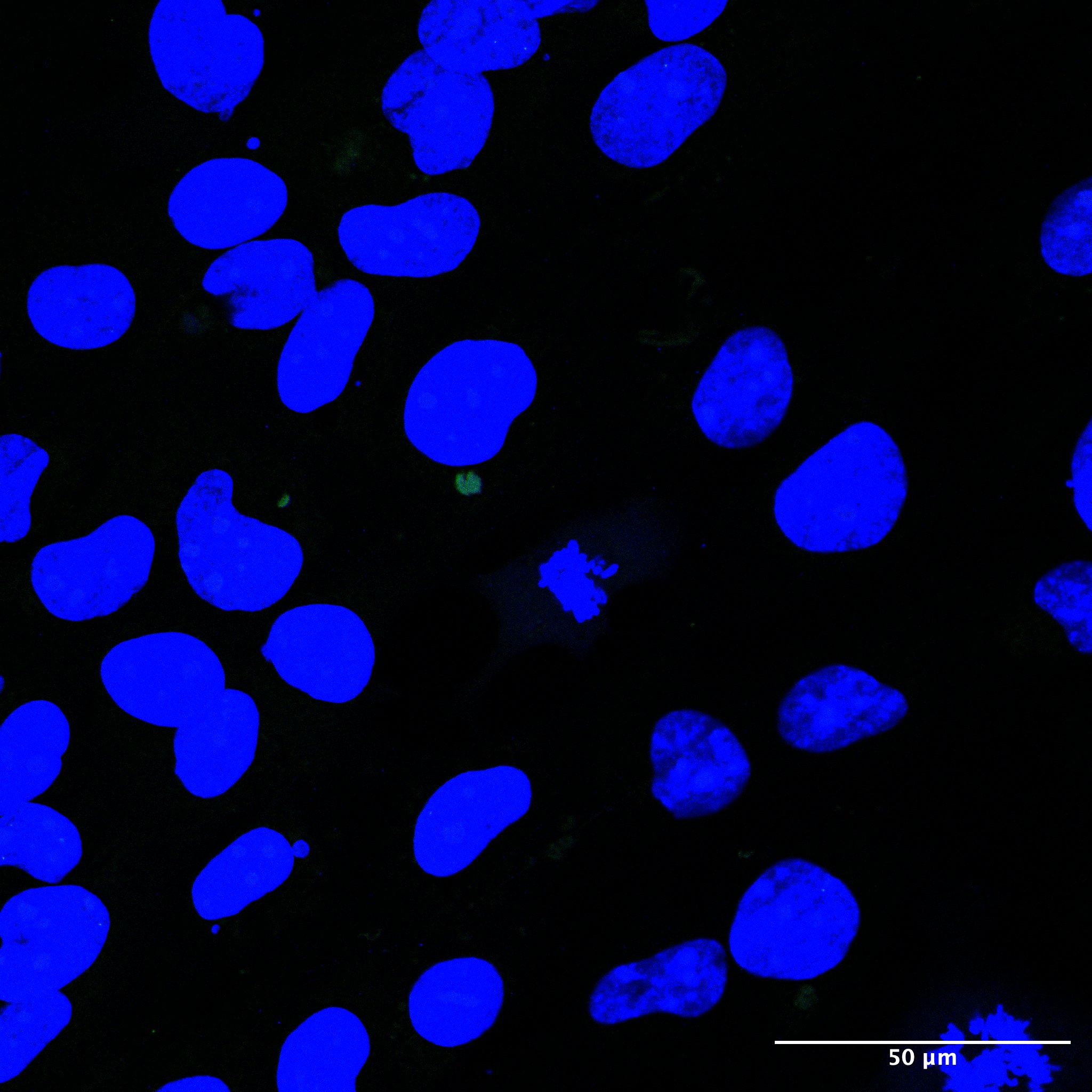

Supplement: Supplementary file 7 — Source data Fig. 5 [file 44318_2025_570_MOESM7_ESM.zip › Fig5/Images/J/Fig_5_panel_j_MYOF_TGFBR1_CTRL_4_big.jpg]

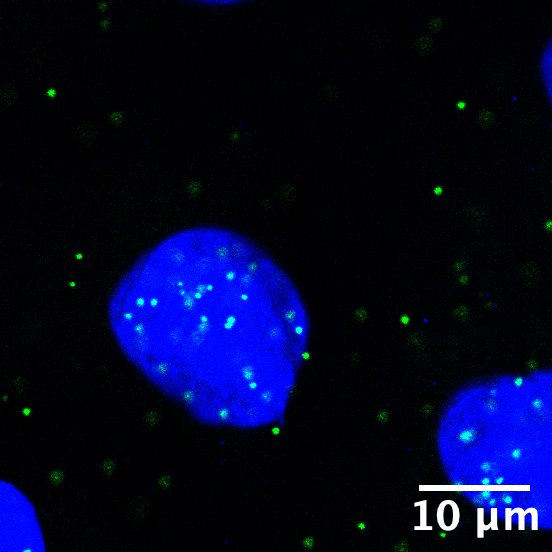

Supplement: Supplementary file 7 — Source data Fig. 5 [file 44318_2025_570_MOESM7_ESM.zip › Fig5/Images/J/Fig_5_panel_j_MYOF_TGFBR1_sh#1_4.jpg]

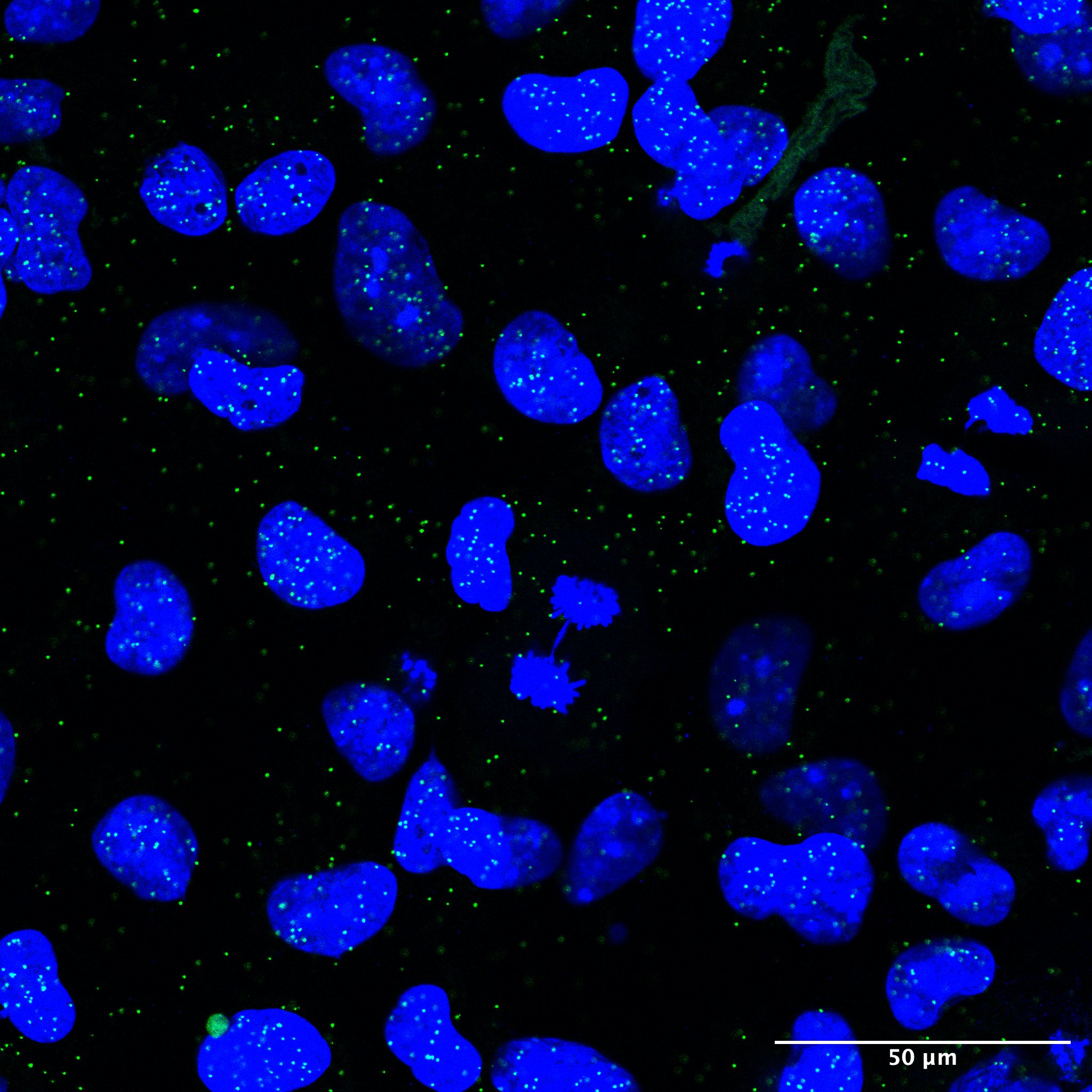

Supplement: Supplementary file 7 — Source data Fig. 5 [file 44318_2025_570_MOESM7_ESM.zip › Fig5/Images/J/Fig_5_panel_j_MYOF_TGFBR1_sh#1_4_big.jpg]

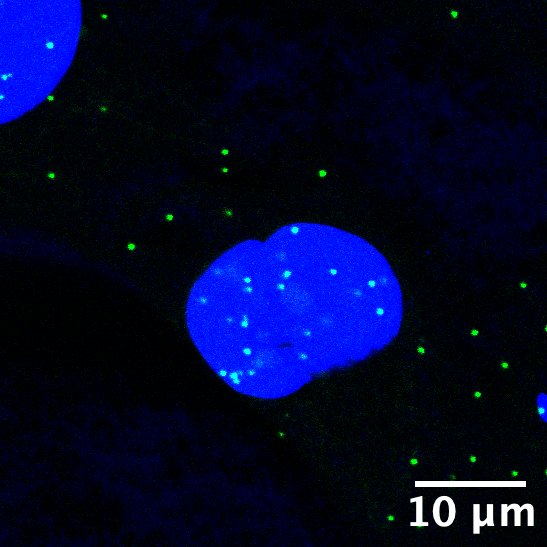

Supplement: Supplementary file 7 — Source data Fig. 5 [file 44318_2025_570_MOESM7_ESM.zip › Fig5/Images/J/Fig_5_panel_j_MYOF_TGFBR1_sh#5_6.jpg]

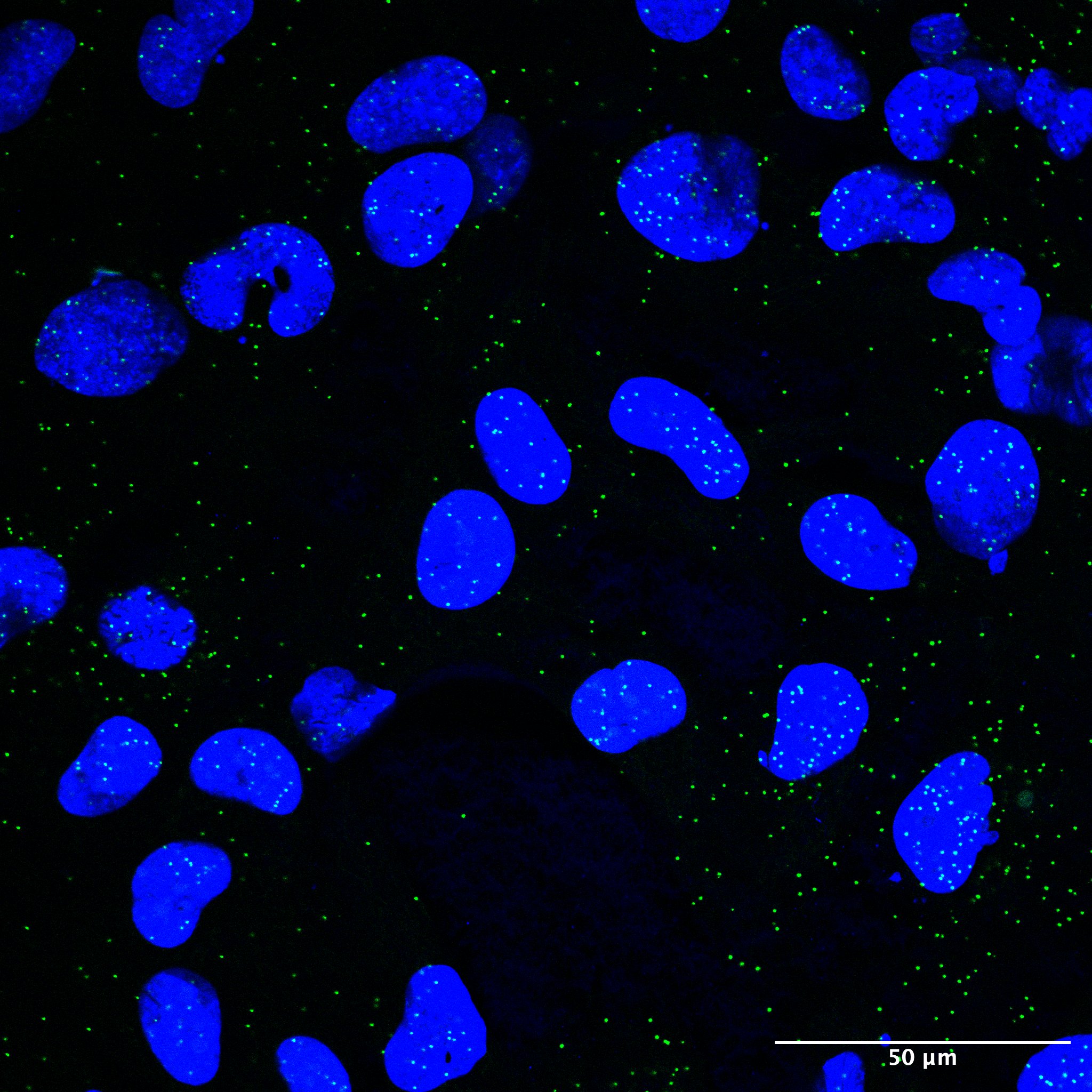

Supplement: Supplementary file 7 — Source data Fig. 5 [file 44318_2025_570_MOESM7_ESM.zip › Fig5/Images/J/Fig_5_panel_j_MYOF_TGFBR1_sh#5_6_big.jpg]

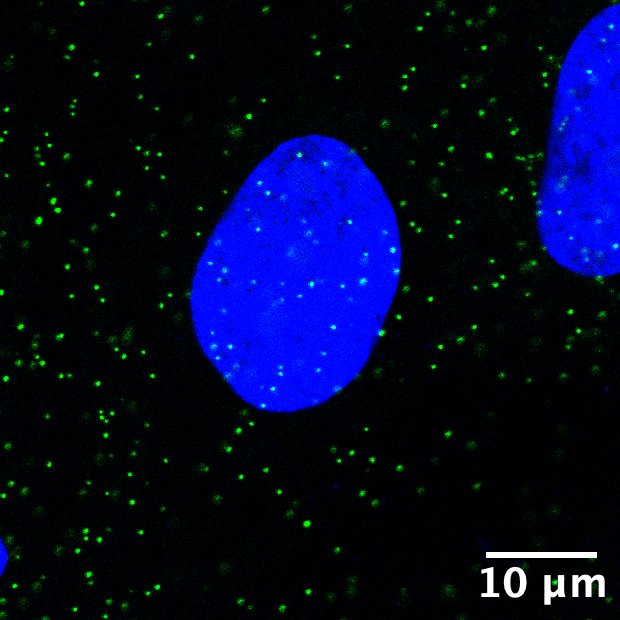

Supplement: Supplementary file 7 — Source data Fig. 5 [file 44318_2025_570_MOESM7_ESM.zip › Fig5/Images/J/Fig_5_panel_j_MYOF_TGFBR1_shNT_3.jpg]

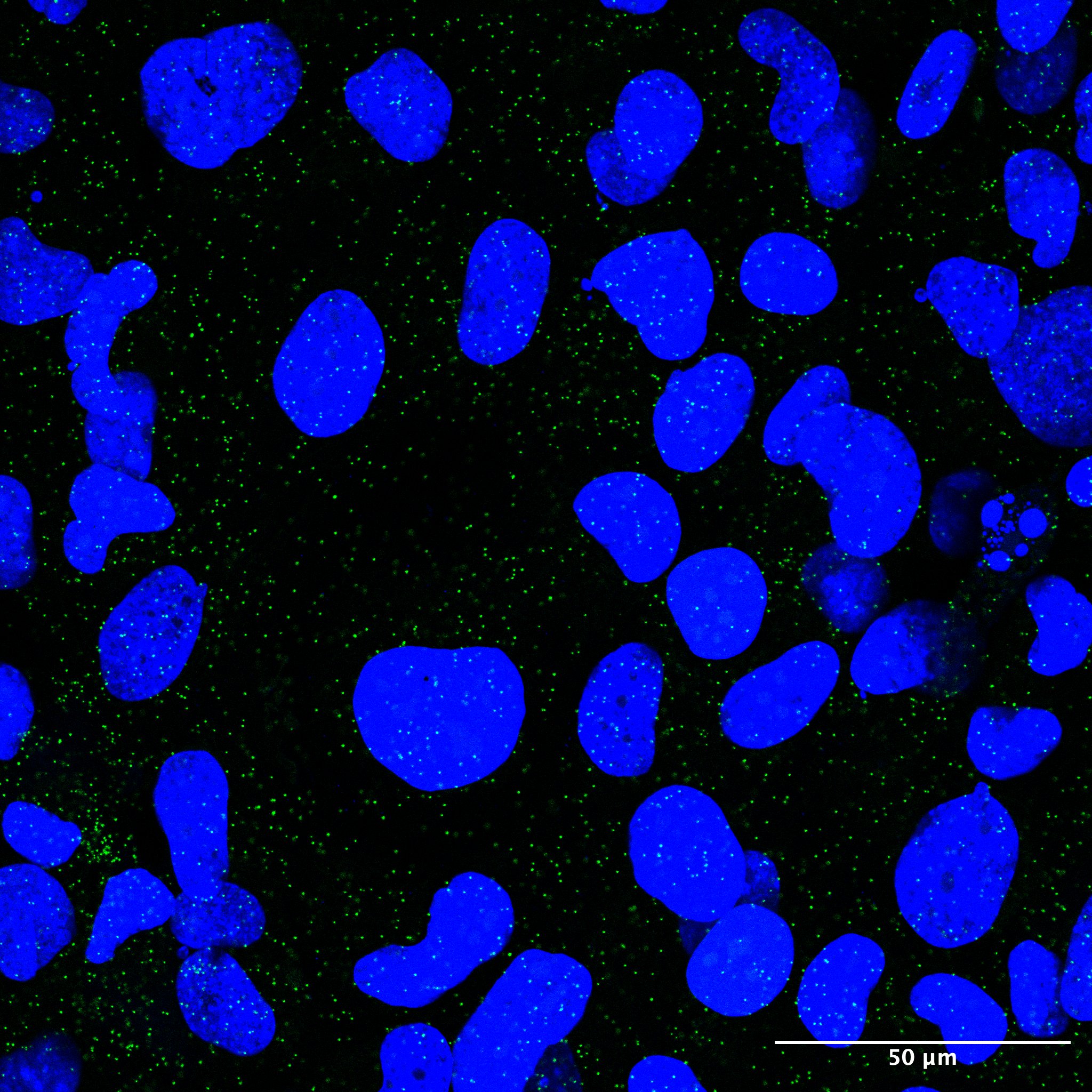

Supplement: Supplementary file 7 — Source data Fig. 5 [file 44318_2025_570_MOESM7_ESM.zip › Fig5/Images/J/Fig_5_panel_j_MYOF_TGFBR1_shNT_3_big.jpg]

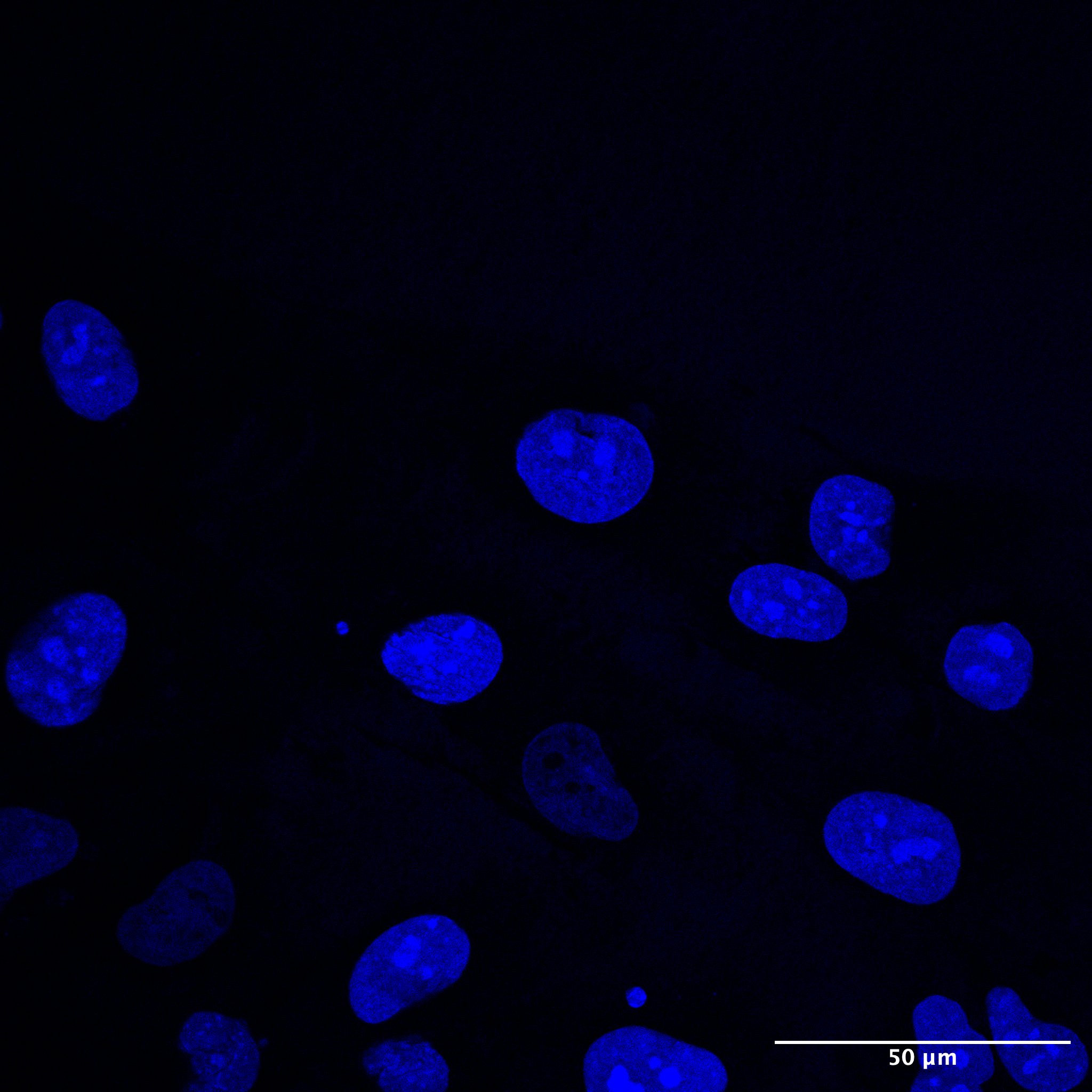

Supplement: Supplementary file 7 — Source data Fig. 5 [file 44318_2025_570_MOESM7_ESM.zip › Fig5/Images/N/Fig_5_panel_n_R1_#1_1_blue.jpg]

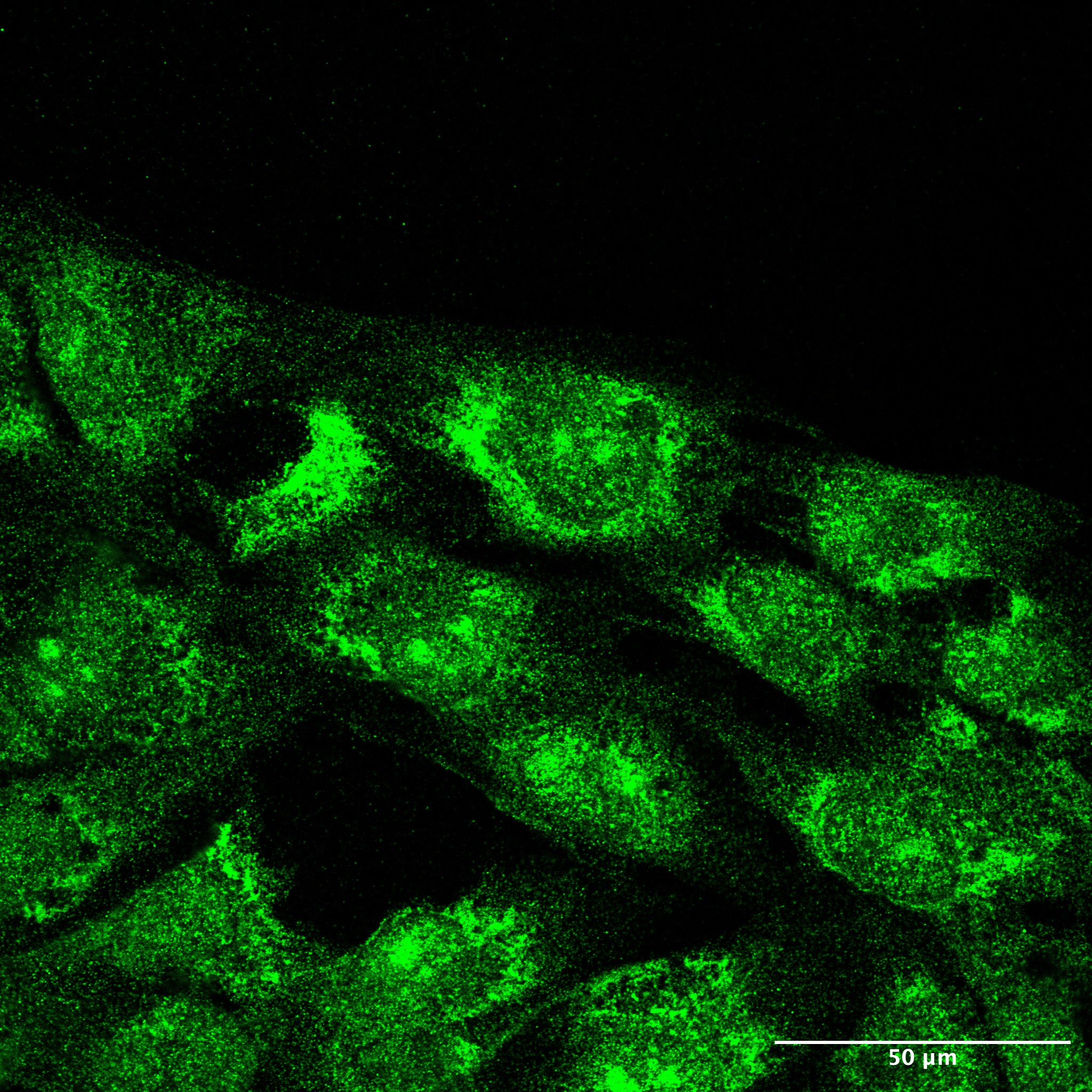

Supplement: Supplementary file 7 — Source data Fig. 5 [file 44318_2025_570_MOESM7_ESM.zip › Fig5/Images/N/Fig_5_panel_n_R1_#1_1_green.jpg]

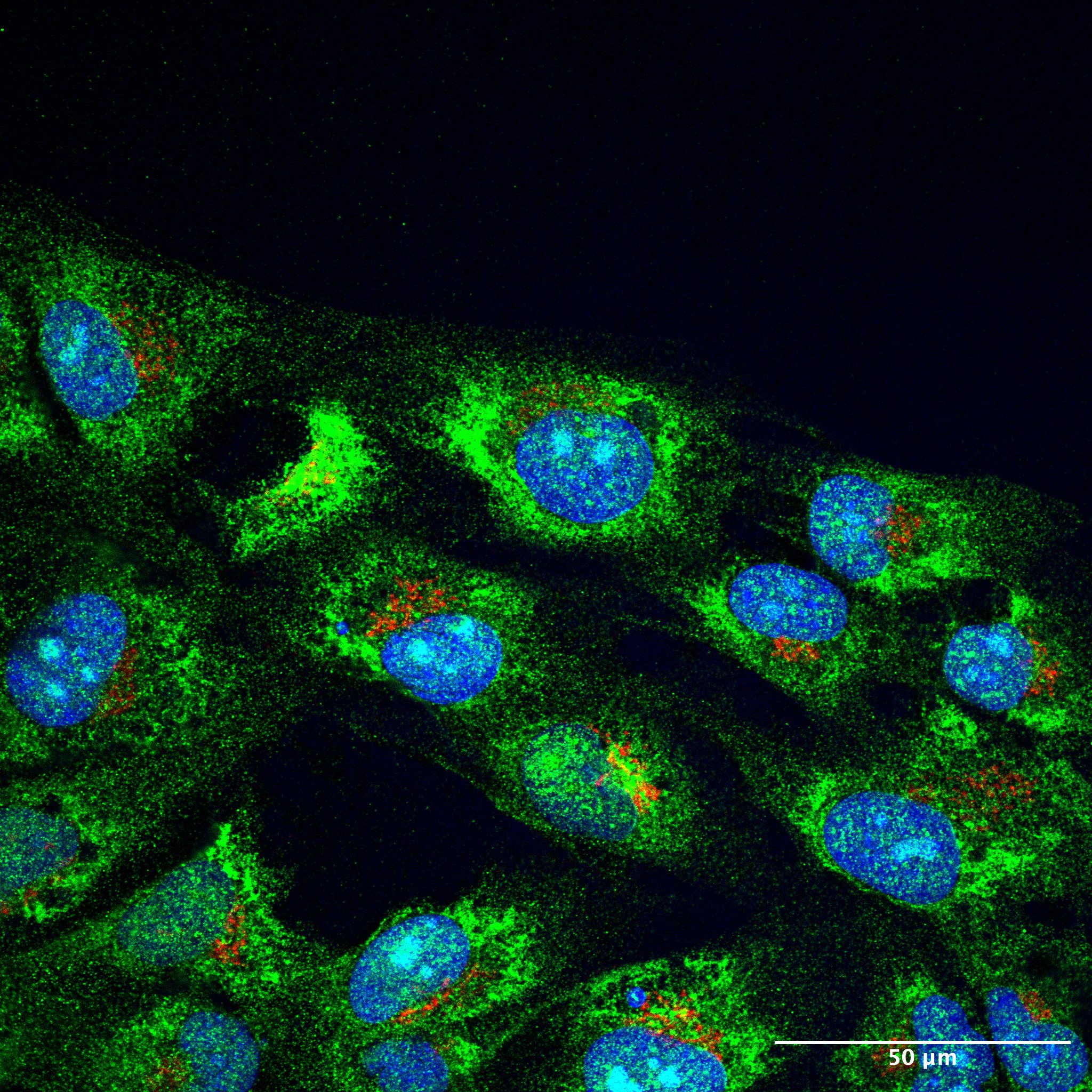

Supplement: Supplementary file 7 — Source data Fig. 5 [file 44318_2025_570_MOESM7_ESM.zip › Fig5/Images/N/Fig_5_panel_n_R1_#1_1_merge.jpg]

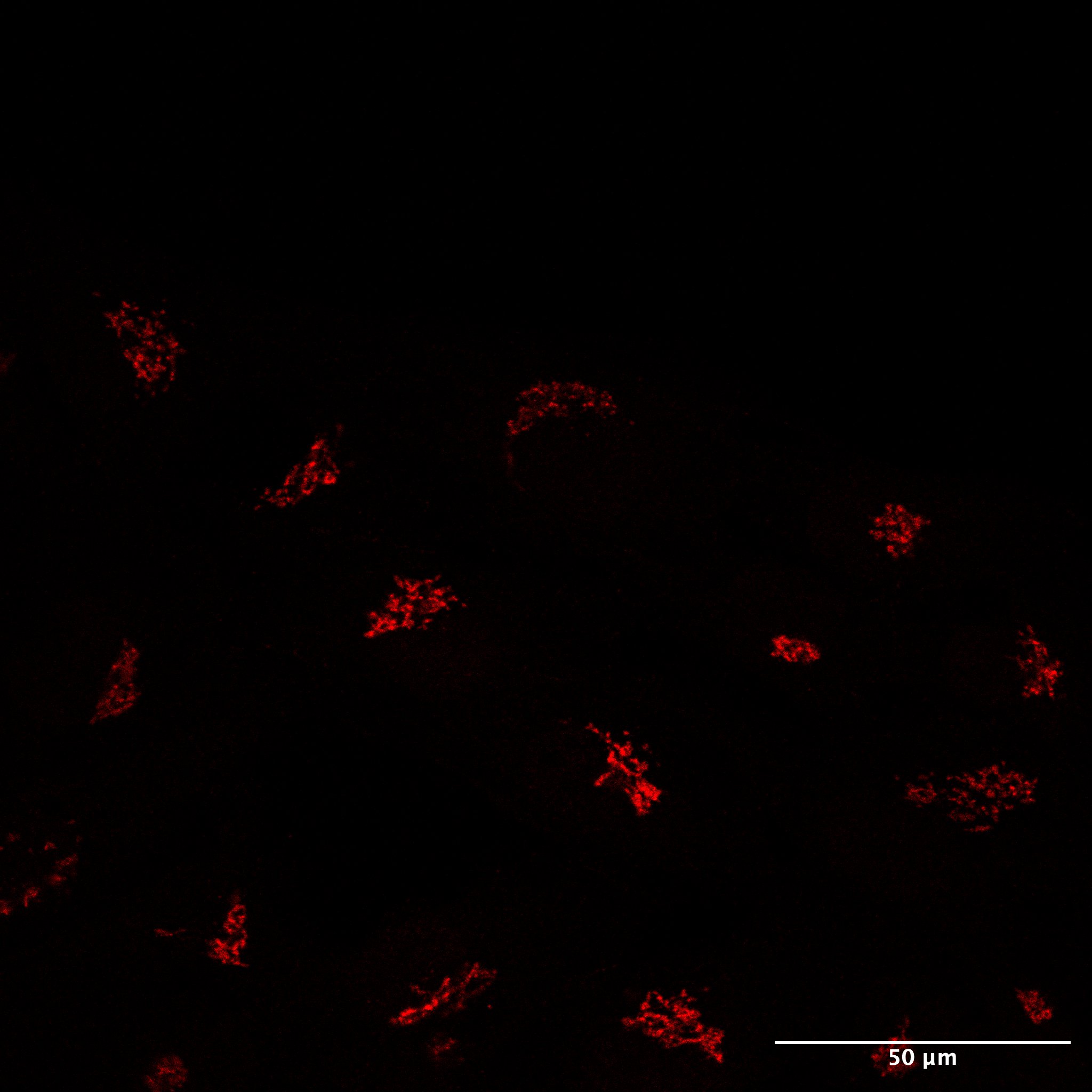

Supplement: Supplementary file 7 — Source data Fig. 5 [file 44318_2025_570_MOESM7_ESM.zip › Fig5/Images/N/Fig_5_panel_n_R1_#1_1_red.jpg]

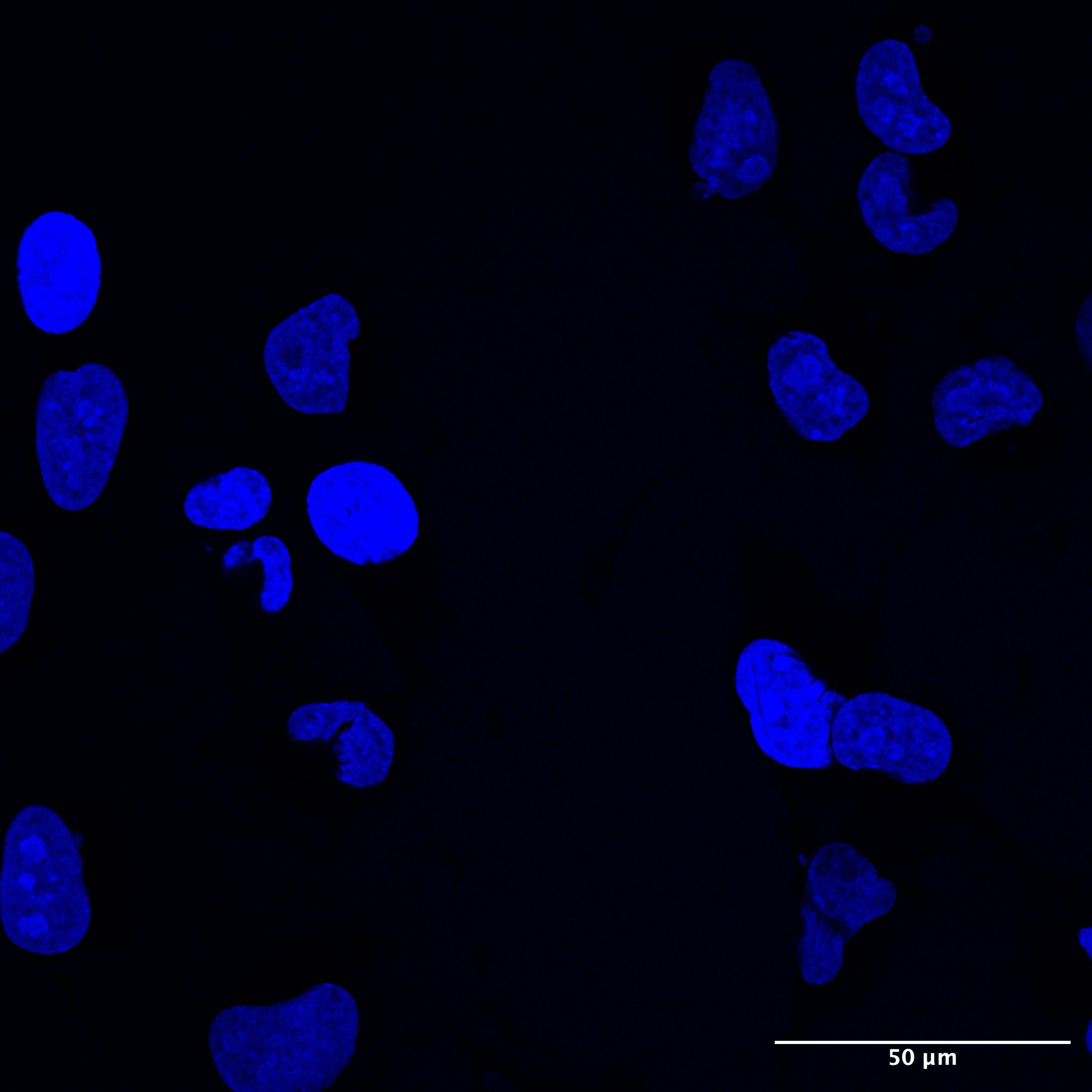

Supplement: Supplementary file 7 — Source data Fig. 5 [file 44318_2025_570_MOESM7_ESM.zip › Fig5/Images/N/Fig_5_panel_n_R1_#5_4_blue.jpg]

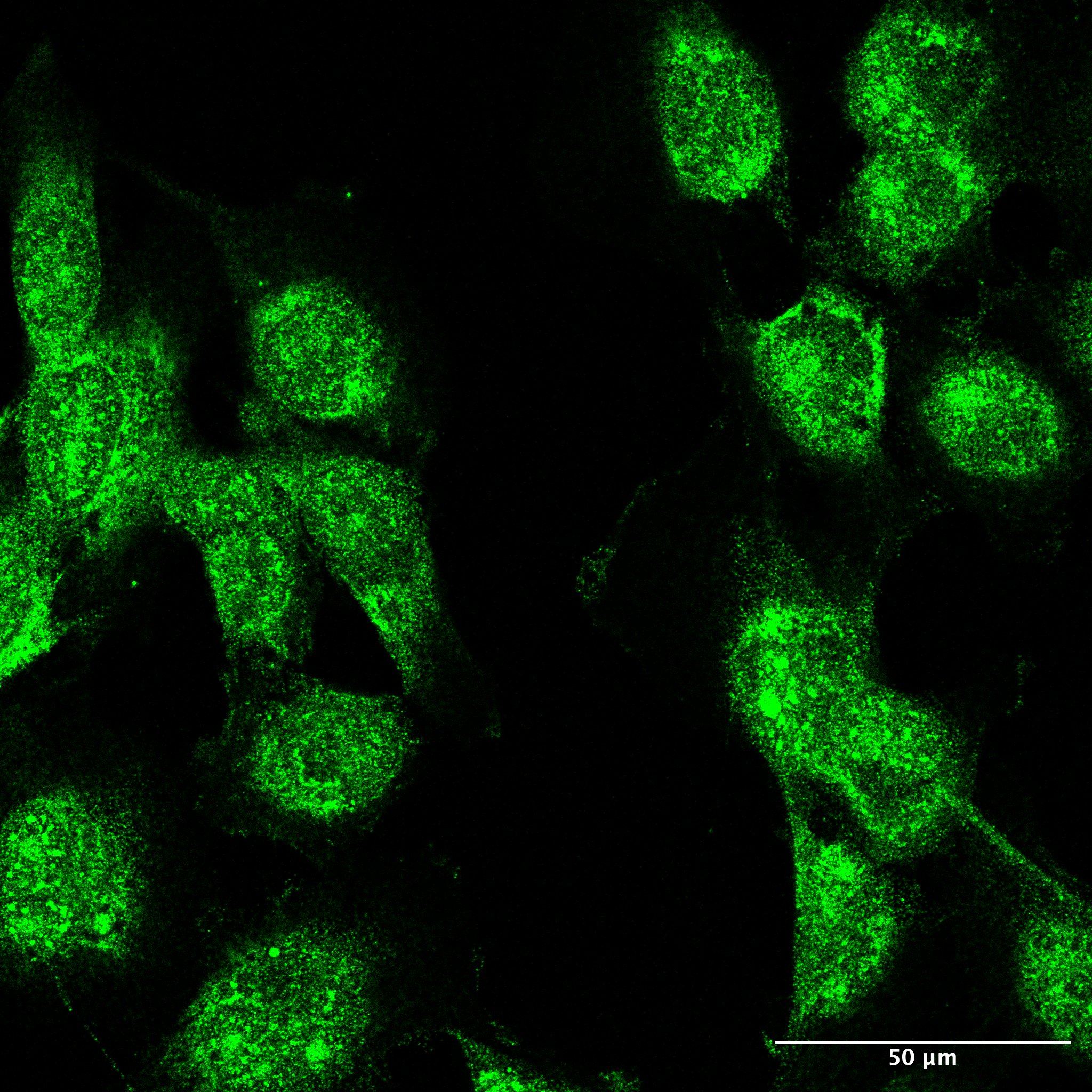

Supplement: Supplementary file 7 — Source data Fig. 5 [file 44318_2025_570_MOESM7_ESM.zip › Fig5/Images/N/Fig_5_panel_n_R1_#5_4_green.jpg]

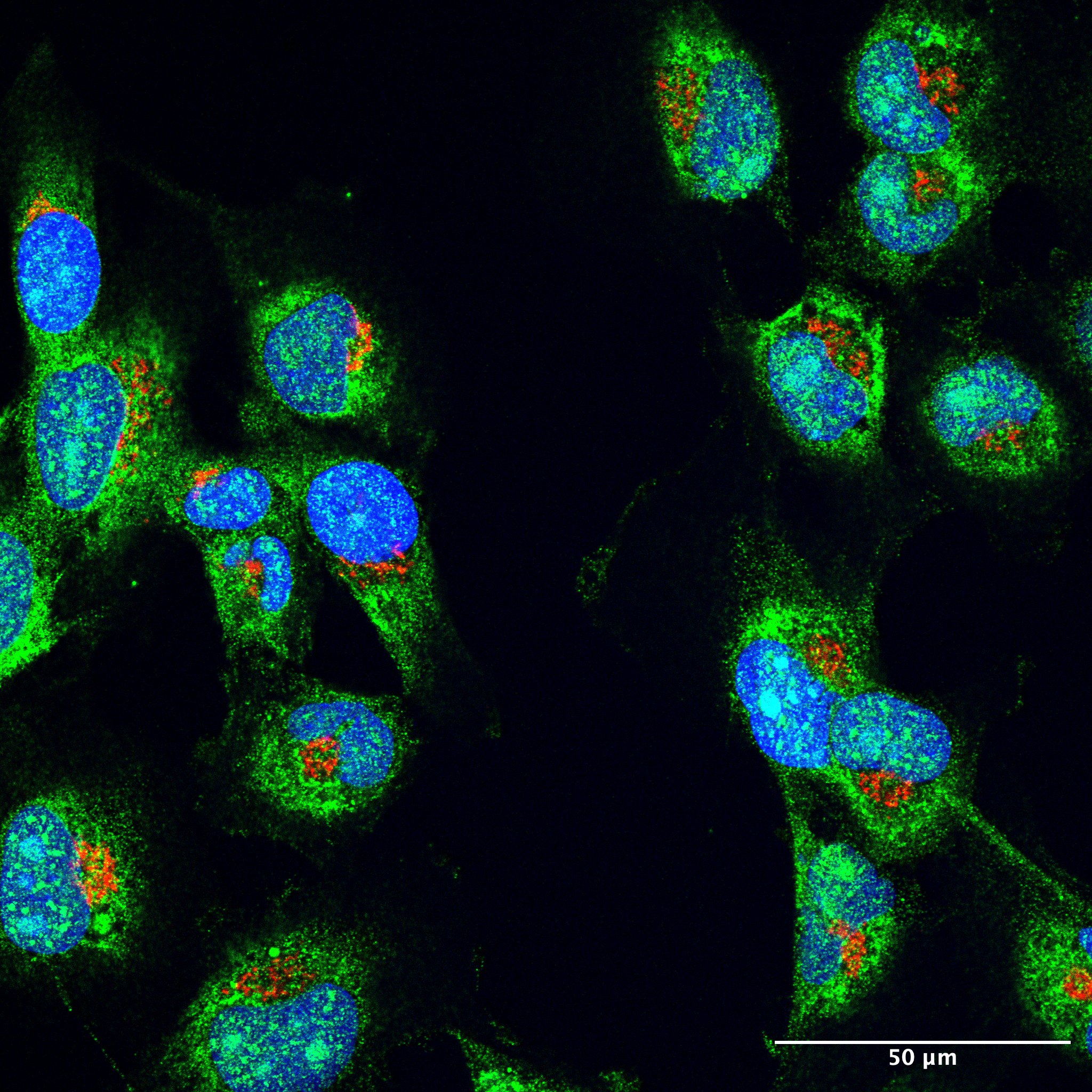

Supplement: Supplementary file 7 — Source data Fig. 5 [file 44318_2025_570_MOESM7_ESM.zip › Fig5/Images/N/Fig_5_panel_n_R1_#5_4_merge.jpg]

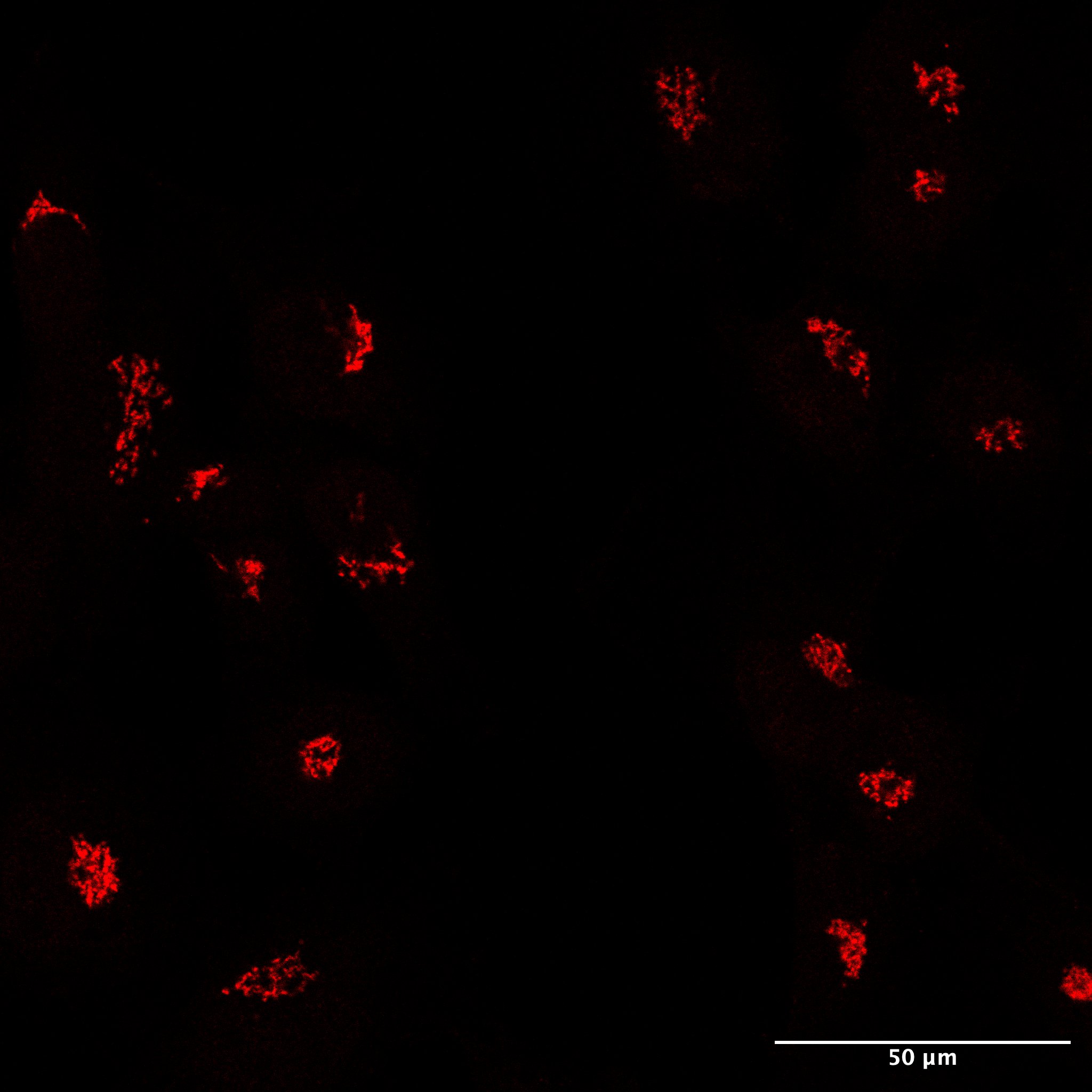

Supplement: Supplementary file 7 — Source data Fig. 5 [file 44318_2025_570_MOESM7_ESM.zip › Fig5/Images/N/Fig_5_panel_n_R1_#5_4_red.jpg]

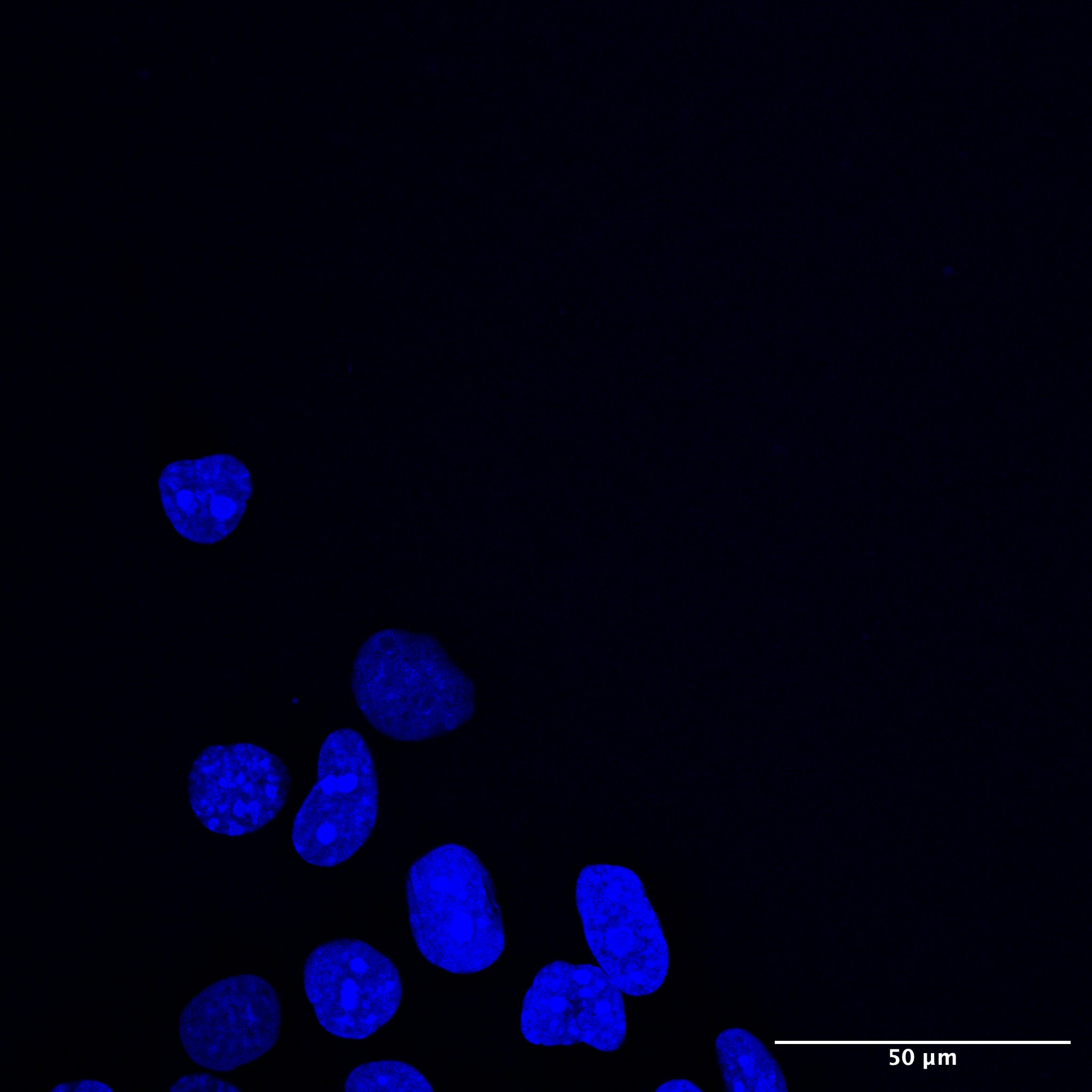

Supplement: Supplementary file 7 — Source data Fig. 5 [file 44318_2025_570_MOESM7_ESM.zip › Fig5/Images/N/Fig_5_panel_n_R1_shNT_2_blue.jpg]

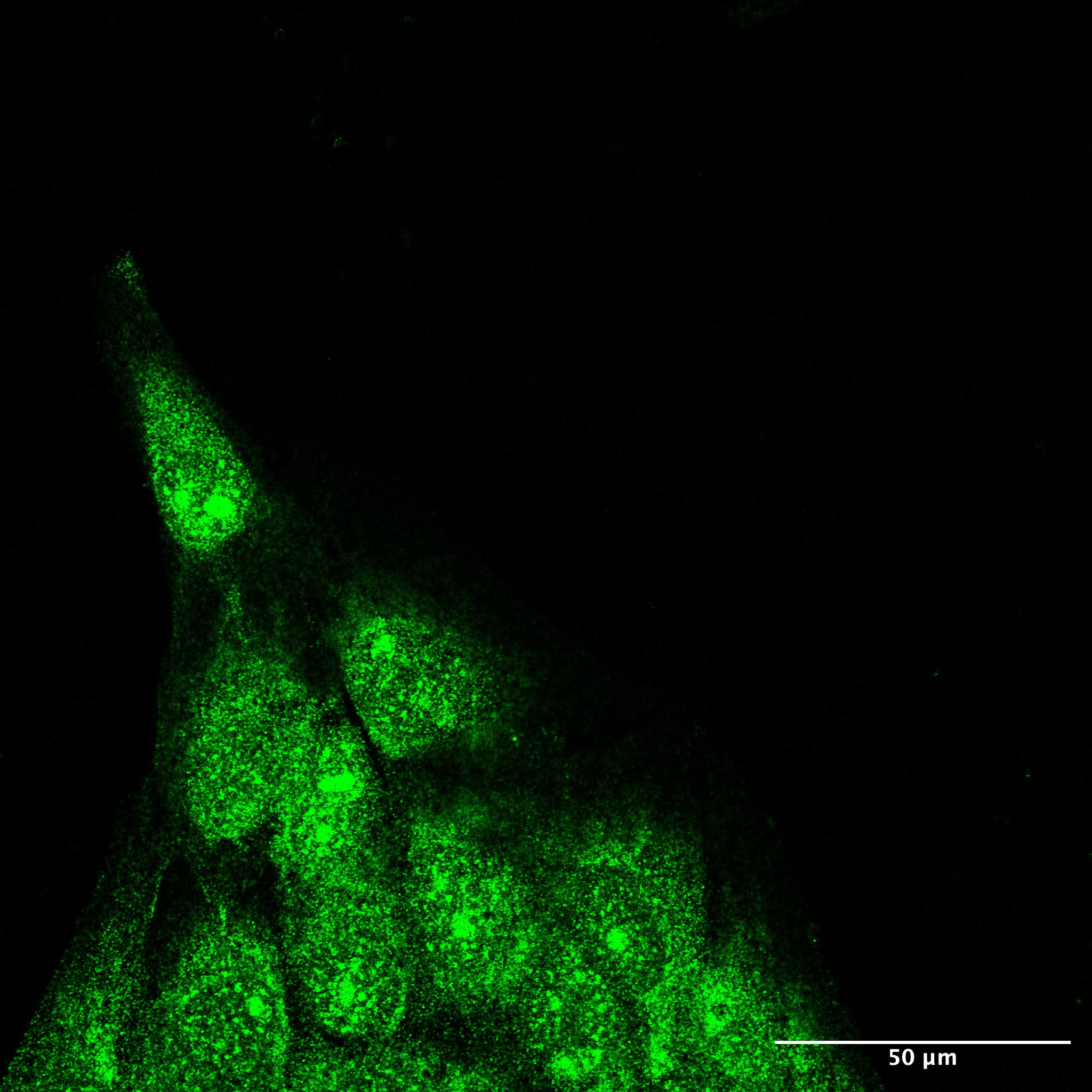

Supplement: Supplementary file 7 — Source data Fig. 5 [file 44318_2025_570_MOESM7_ESM.zip › Fig5/Images/N/Fig_5_panel_n_R1_shNT_2_green.jpg]

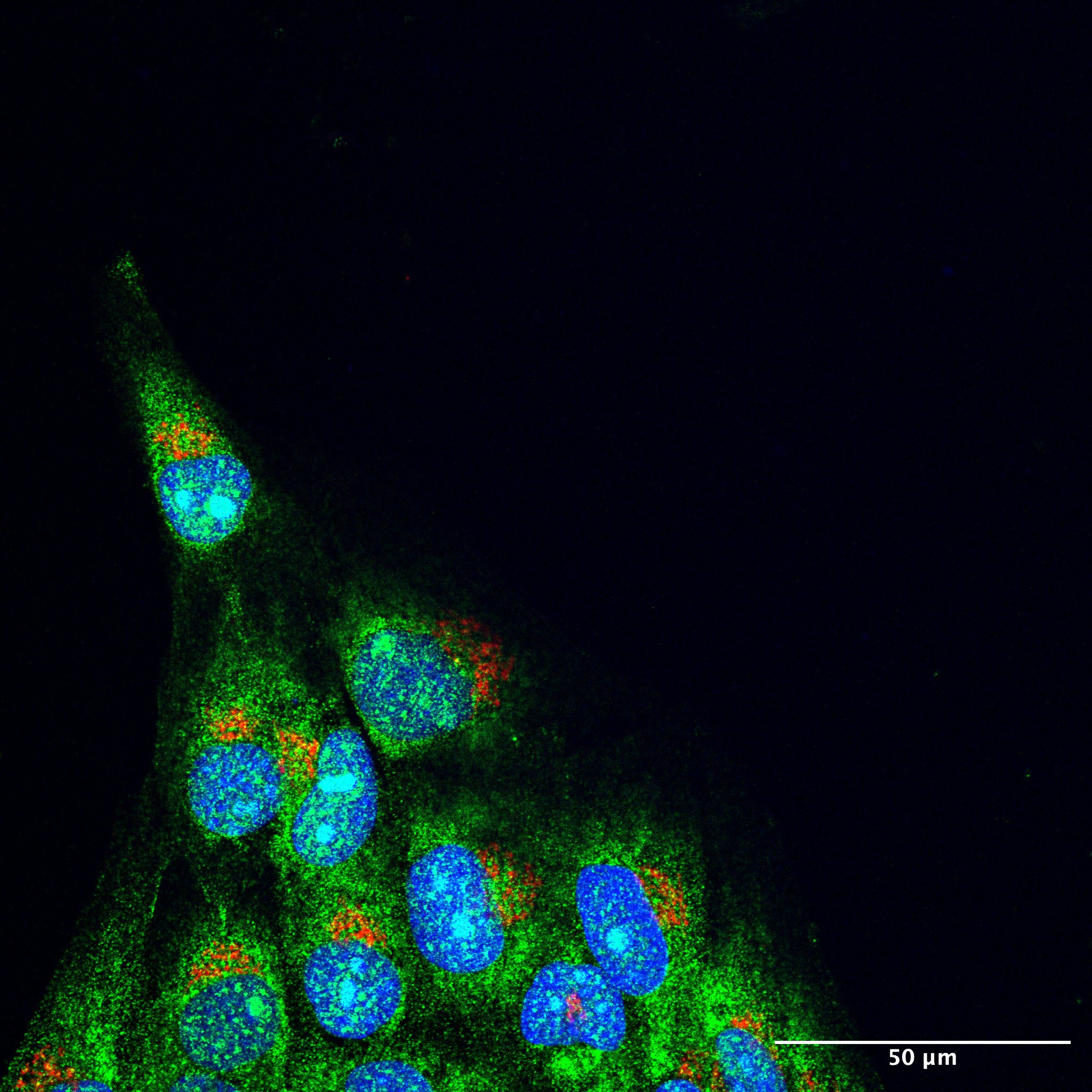

Supplement: Supplementary file 7 — Source data Fig. 5 [file 44318_2025_570_MOESM7_ESM.zip › Fig5/Images/N/Fig_5_panel_n_R1_shNT_2_merge.jpg]

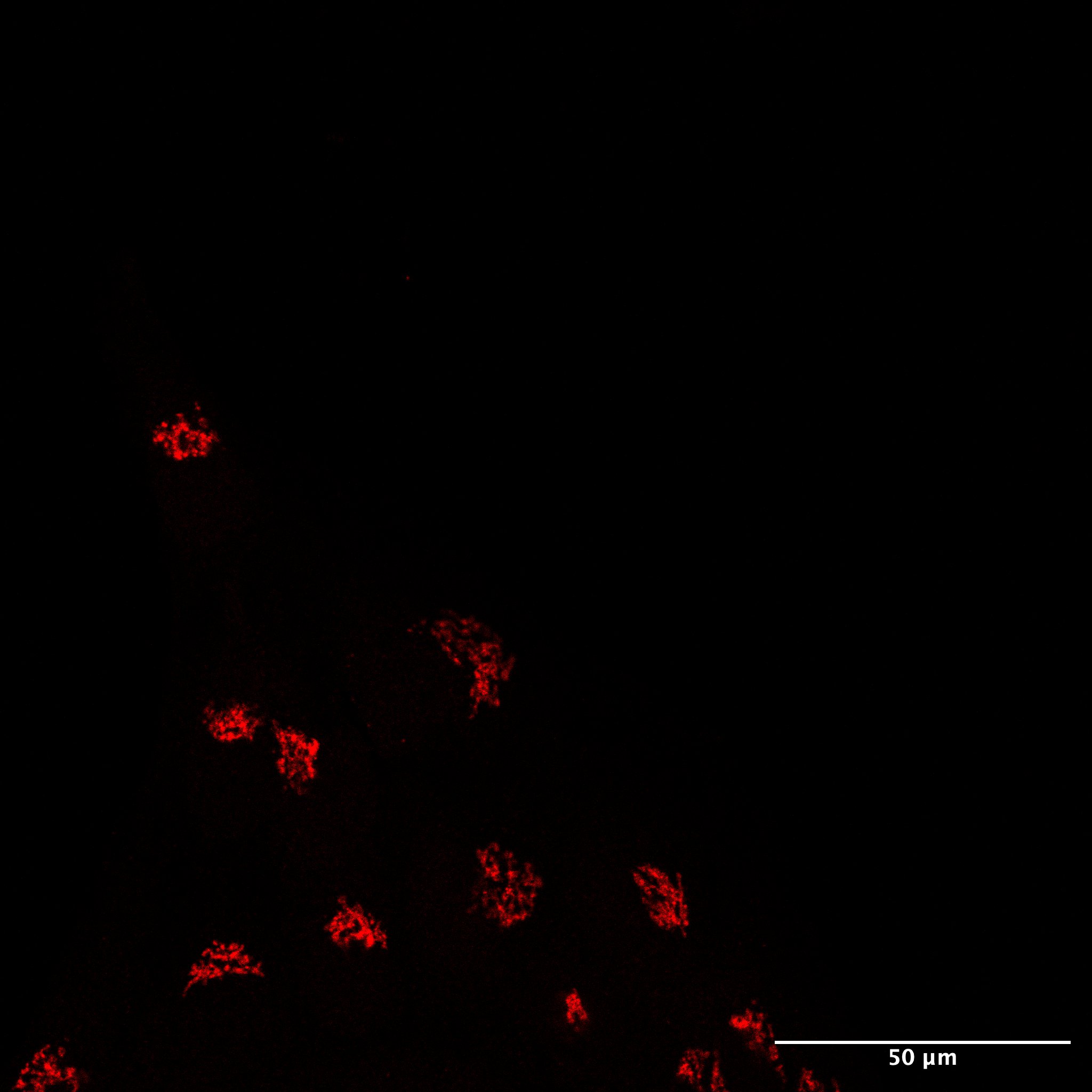

Supplement: Supplementary file 7 — Source data Fig. 5 [file 44318_2025_570_MOESM7_ESM.zip › Fig5/Images/N/Fig_5_panel_n_R1_shNT_2_red.jpg]

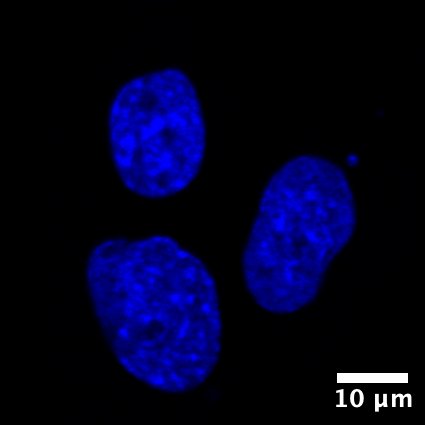

Supplement: Supplementary file 7 — Source data Fig. 5 [file 44318_2025_570_MOESM7_ESM.zip › Fig5/Images/P/Fig_5_panel_p_BFA2_blue_Big.jpg]

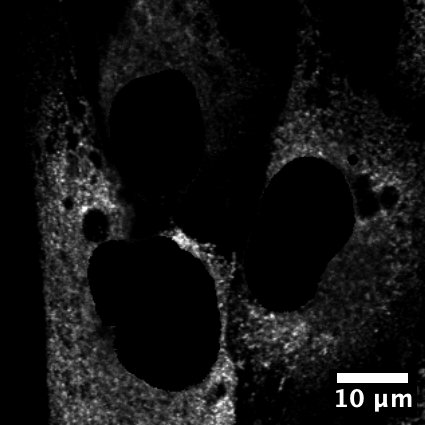

Supplement: Supplementary file 7 — Source data Fig. 5 [file 44318_2025_570_MOESM7_ESM.zip › Fig5/Images/P/Fig_5_panel_p_BFA2_gray_Big.jpg]

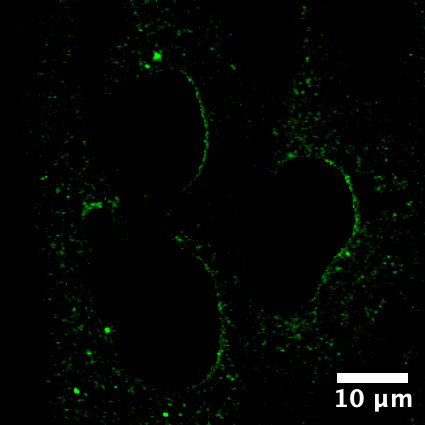

Supplement: Supplementary file 7 — Source data Fig. 5 [file 44318_2025_570_MOESM7_ESM.zip › Fig5/Images/P/Fig_5_panel_p_BFA2_green_Big.jpg]

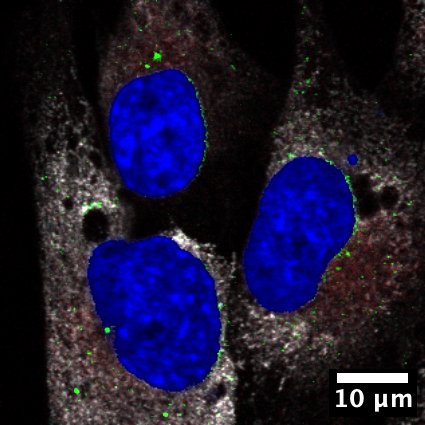

Supplement: Supplementary file 7 — Source data Fig. 5 [file 44318_2025_570_MOESM7_ESM.zip › Fig5/Images/P/Fig_5_panel_p_BFA2_merge_Big.jpg]

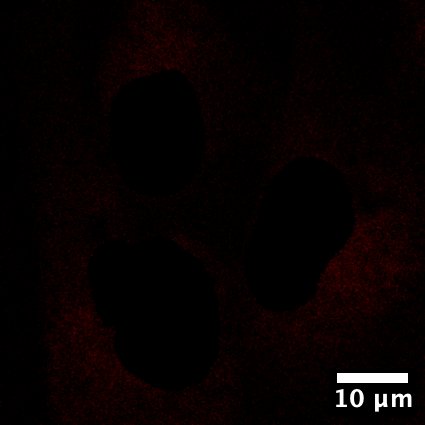

Supplement: Supplementary file 7 — Source data Fig. 5 [file 44318_2025_570_MOESM7_ESM.zip › Fig5/Images/P/Fig_5_panel_p_BFA2_red_Big.jpg]

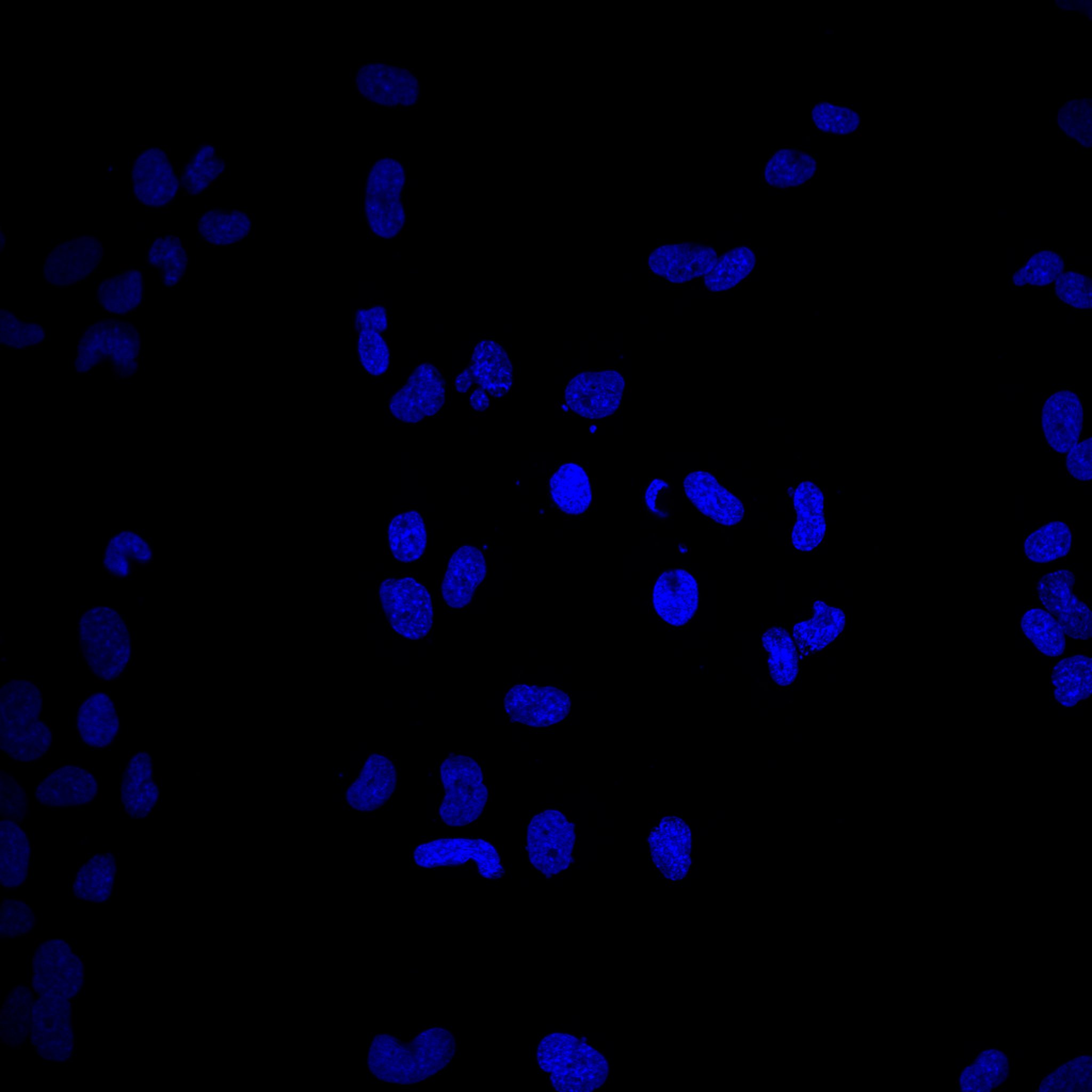

Supplement: Supplementary file 7 — Source data Fig. 5 [file 44318_2025_570_MOESM7_ESM.zip › Fig5/Images/P/Fig_5_panel_p_BFA_2_blue.jpg]

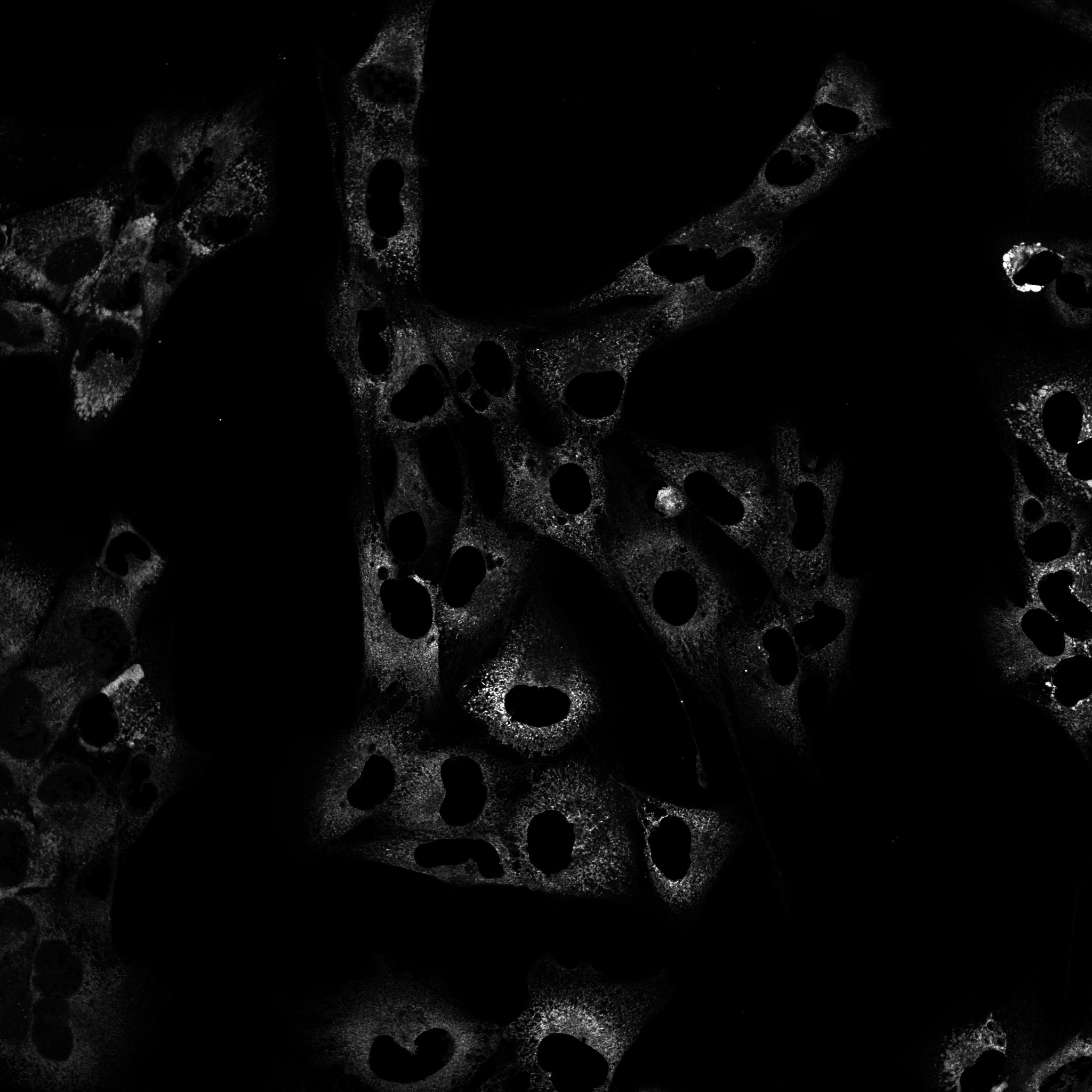

Supplement: Supplementary file 7 — Source data Fig. 5 [file 44318_2025_570_MOESM7_ESM.zip › Fig5/Images/P/Fig_5_panel_p_BFA_2_gray.jpg]

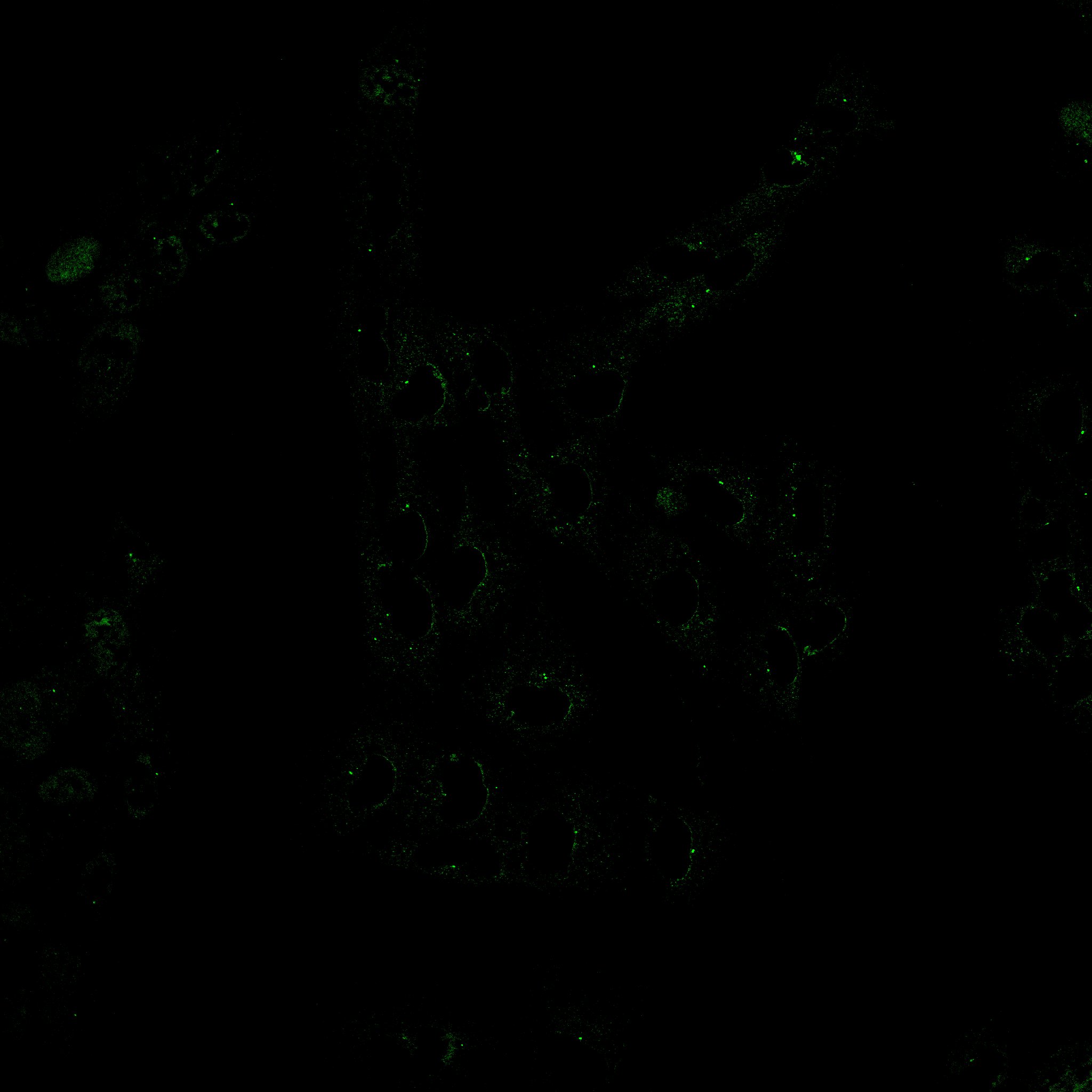

Supplement: Supplementary file 7 — Source data Fig. 5 [file 44318_2025_570_MOESM7_ESM.zip › Fig5/Images/P/Fig_5_panel_p_BFA_2_green.jpg]

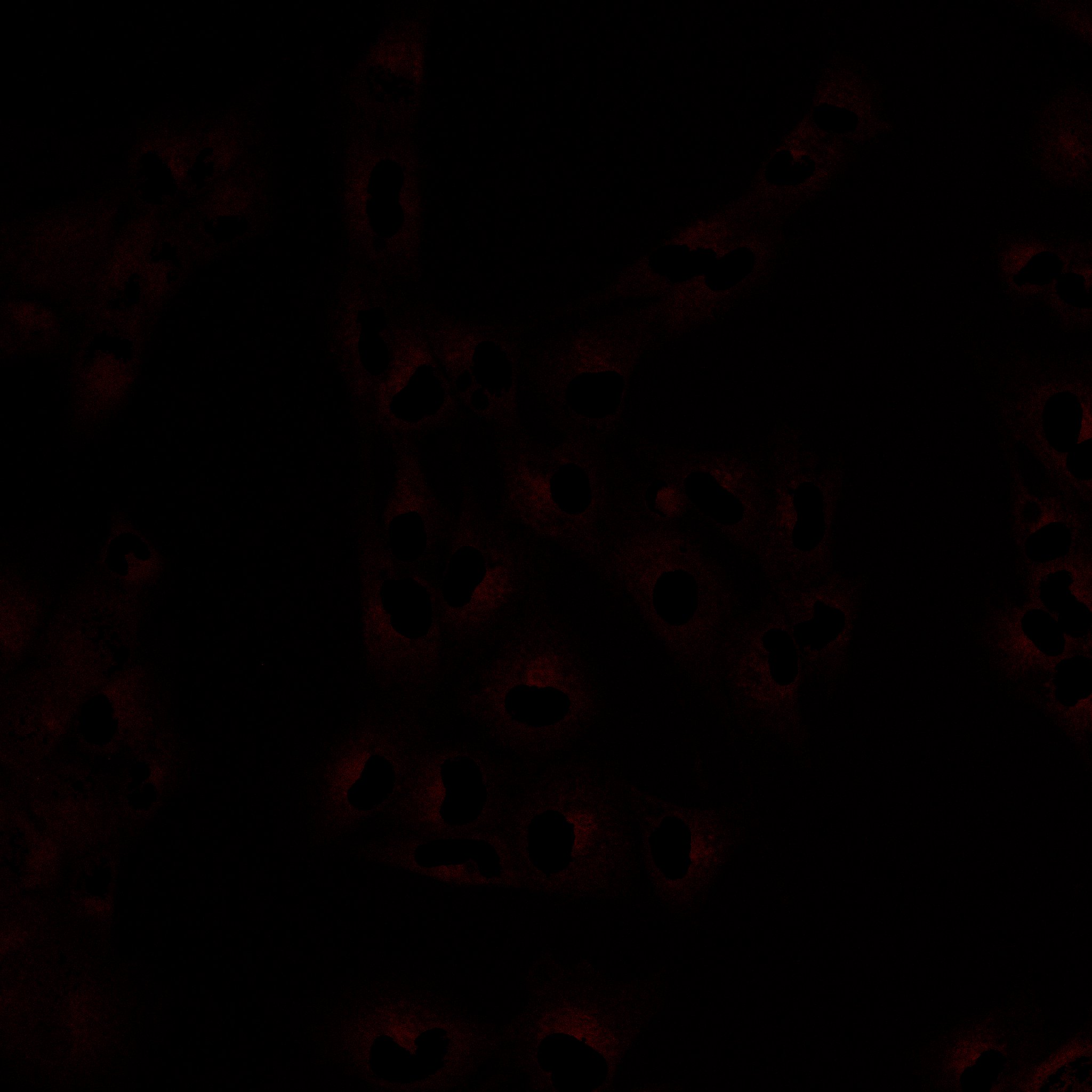

Supplement: Supplementary file 7 — Source data Fig. 5 [file 44318_2025_570_MOESM7_ESM.zip › Fig5/Images/P/Fig_5_panel_p_BFA_2_red.jpg]
