# Supplementary material for: Targeting myoferlin in ER/Golgi vesicle trafficking reprograms pancreatic cancer-associated fibroblasts
Source: EMBO J. 2025 Oct 8;44(22):6425–65. doi: 10.1038/s44318-025-00570-6 (PMC12623807; doi:10.1038/s44318-025-00570-6)
Supplement: Supplementary file 7 — Source data Fig. 5 [file 44318_2025_570_MOESM7_ESM.zip › Fig5/Western_blot/Fig5_uncropped_blots.pptx]

## Slide 1
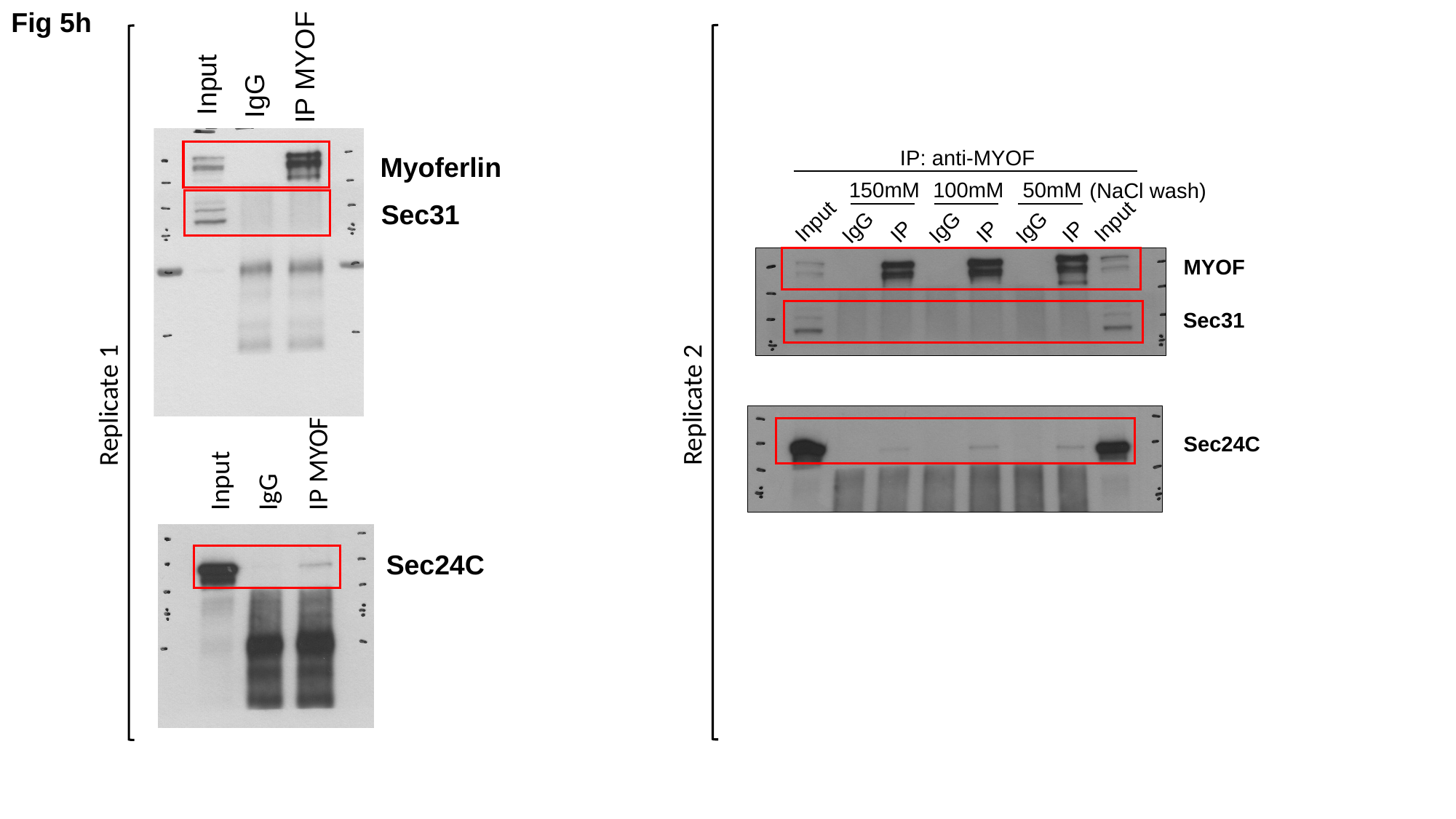

Fig 5h
IP MYOF
Input
IgG
IP: anti-MYOF
Myoferlin
150mM
100mM
50mM
(NaCl wash)
Sec31
Input
Input
IgG
IgG
IgG
IP
IP
IP
MYOF
Sec31
Replicate 2
Replicate 1
Sec24C
IP MYOF
Input
IgG
Sec24C
