# Supplementary figures and images for: Targeting myoferlin in ER/Golgi vesicle trafficking reprograms pancreatic cancer-associated fibroblasts (part 2 of 2)
Source: EMBO J. 2025 Oct 8;44(22):6425–65. doi: 10.1038/s44318-025-00570-6 (PMC12623807; doi:10.1038/s44318-025-00570-6)

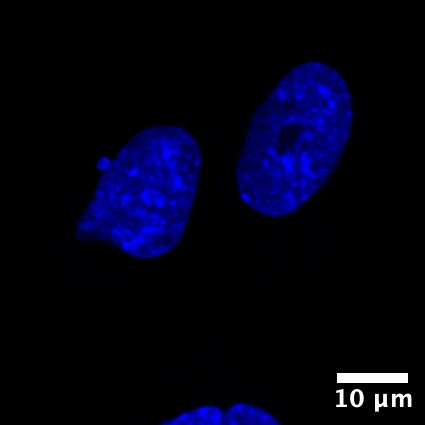

Supplement: Supplementary file 7 — Source data Fig. 5 [file 44318_2025_570_MOESM7_ESM.zip › Fig5/Images/P/Fig_5_panel_p_NTa5_blue_Big.jpg]

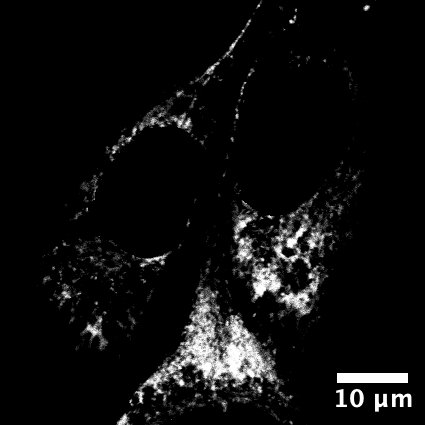

Supplement: Supplementary file 7 — Source data Fig. 5 [file 44318_2025_570_MOESM7_ESM.zip › Fig5/Images/P/Fig_5_panel_p_NTa5_gray_Big.jpg]

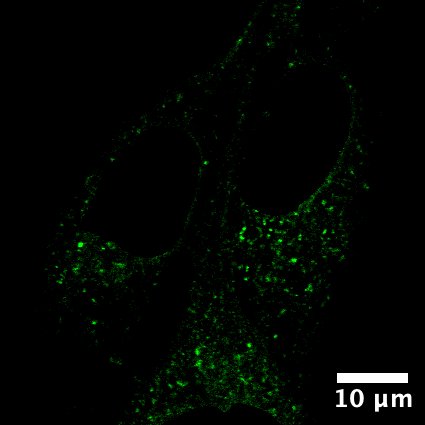

Supplement: Supplementary file 7 — Source data Fig. 5 [file 44318_2025_570_MOESM7_ESM.zip › Fig5/Images/P/Fig_5_panel_p_NTa5_green_Big.jpg]

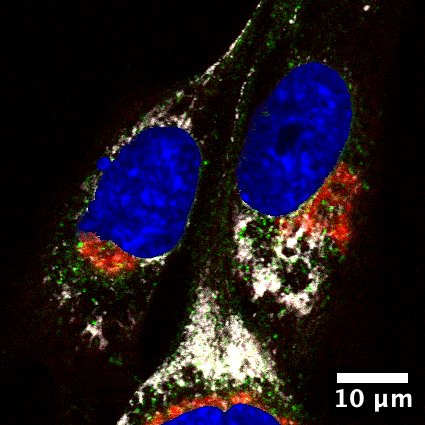

Supplement: Supplementary file 7 — Source data Fig. 5 [file 44318_2025_570_MOESM7_ESM.zip › Fig5/Images/P/Fig_5_panel_p_NTa5_merge_Big.jpg]

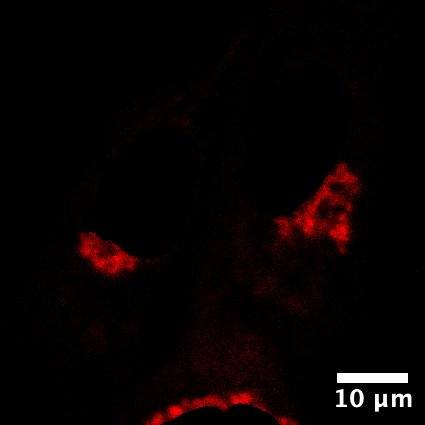

Supplement: Supplementary file 7 — Source data Fig. 5 [file 44318_2025_570_MOESM7_ESM.zip › Fig5/Images/P/Fig_5_panel_p_NTa5_red_Big.jpg]

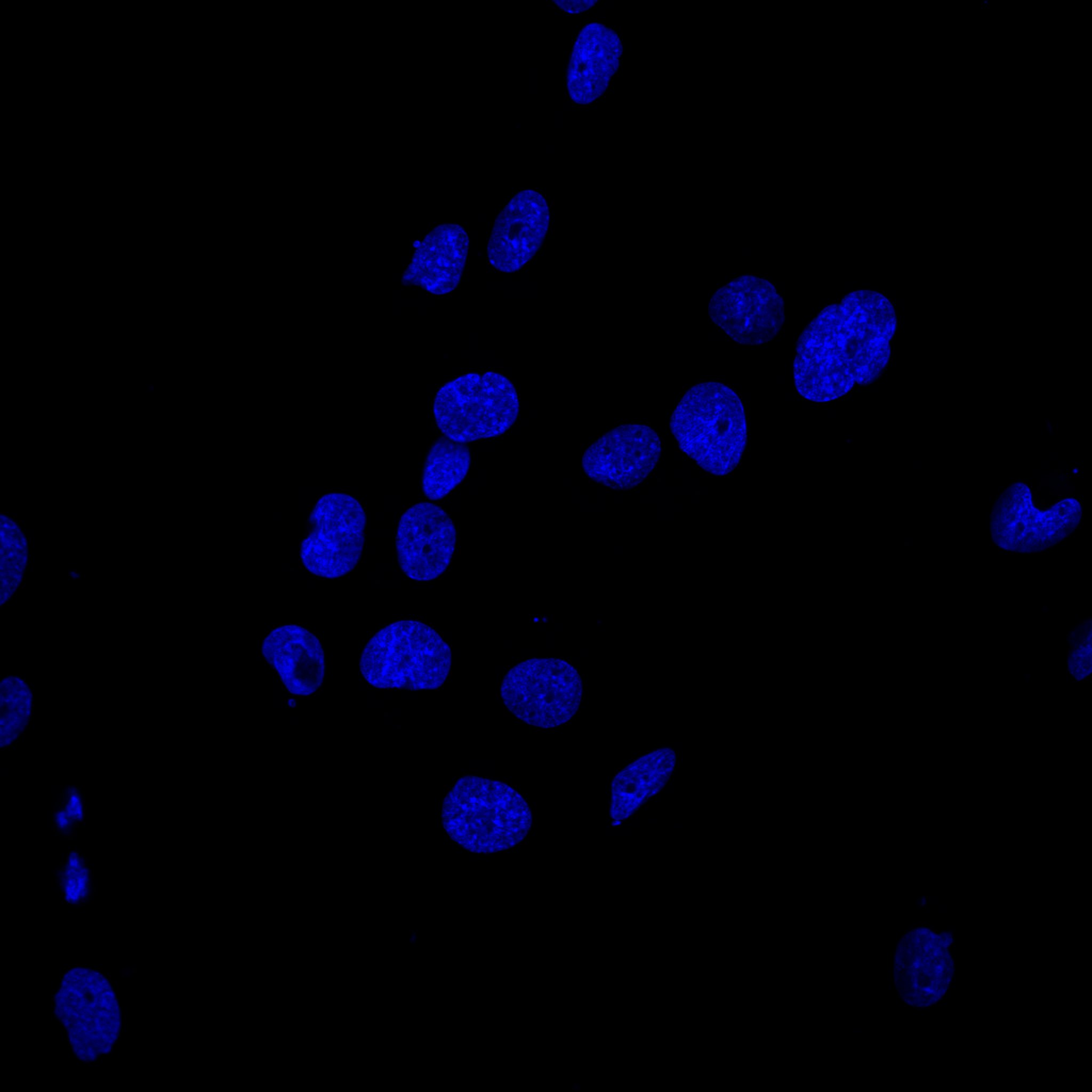

Supplement: Supplementary file 7 — Source data Fig. 5 [file 44318_2025_570_MOESM7_ESM.zip › Fig5/Images/P/Fig_5_panel_p_NTa_5_blue.jpg]

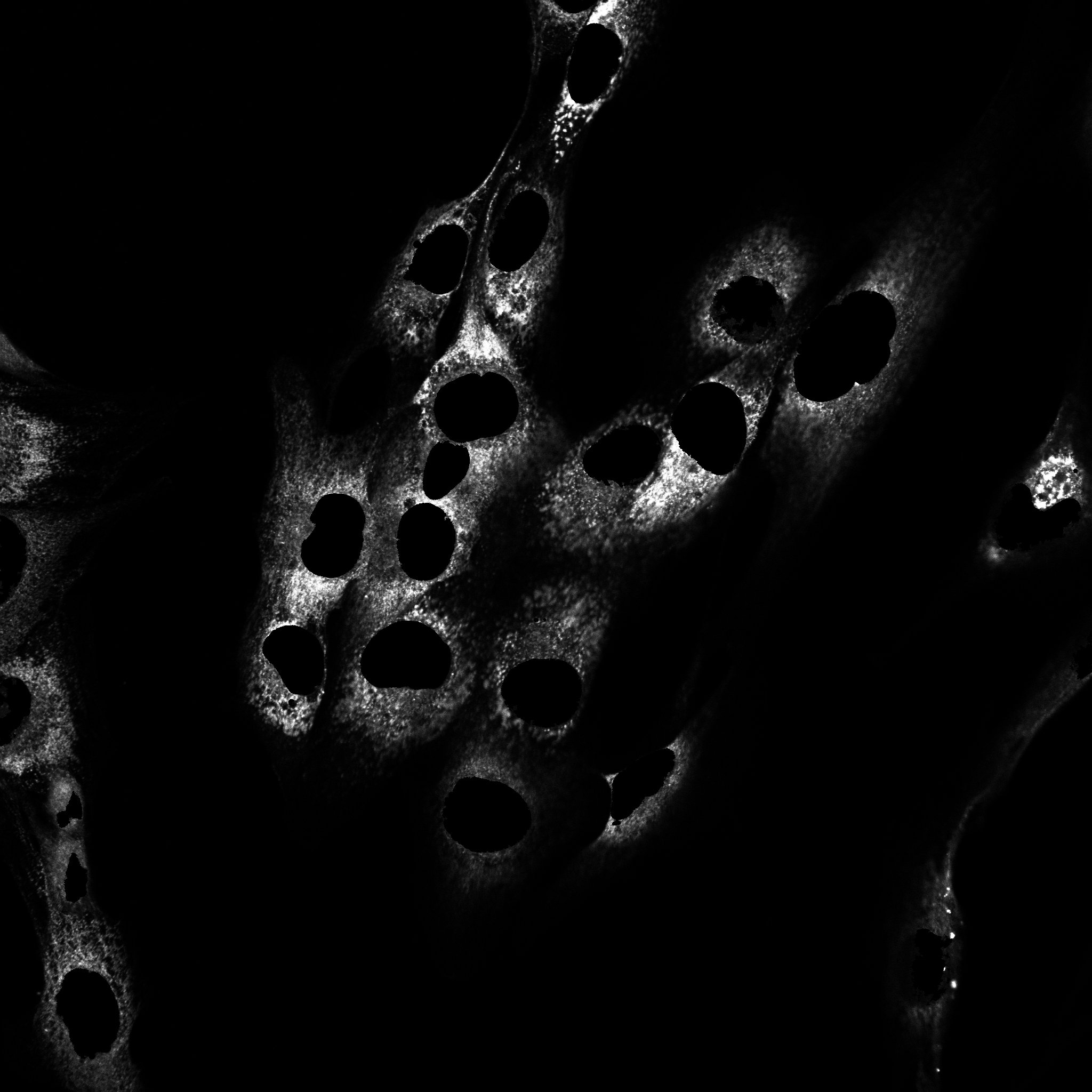

Supplement: Supplementary file 7 — Source data Fig. 5 [file 44318_2025_570_MOESM7_ESM.zip › Fig5/Images/P/Fig_5_panel_p_NTa_5_gray.jpg]

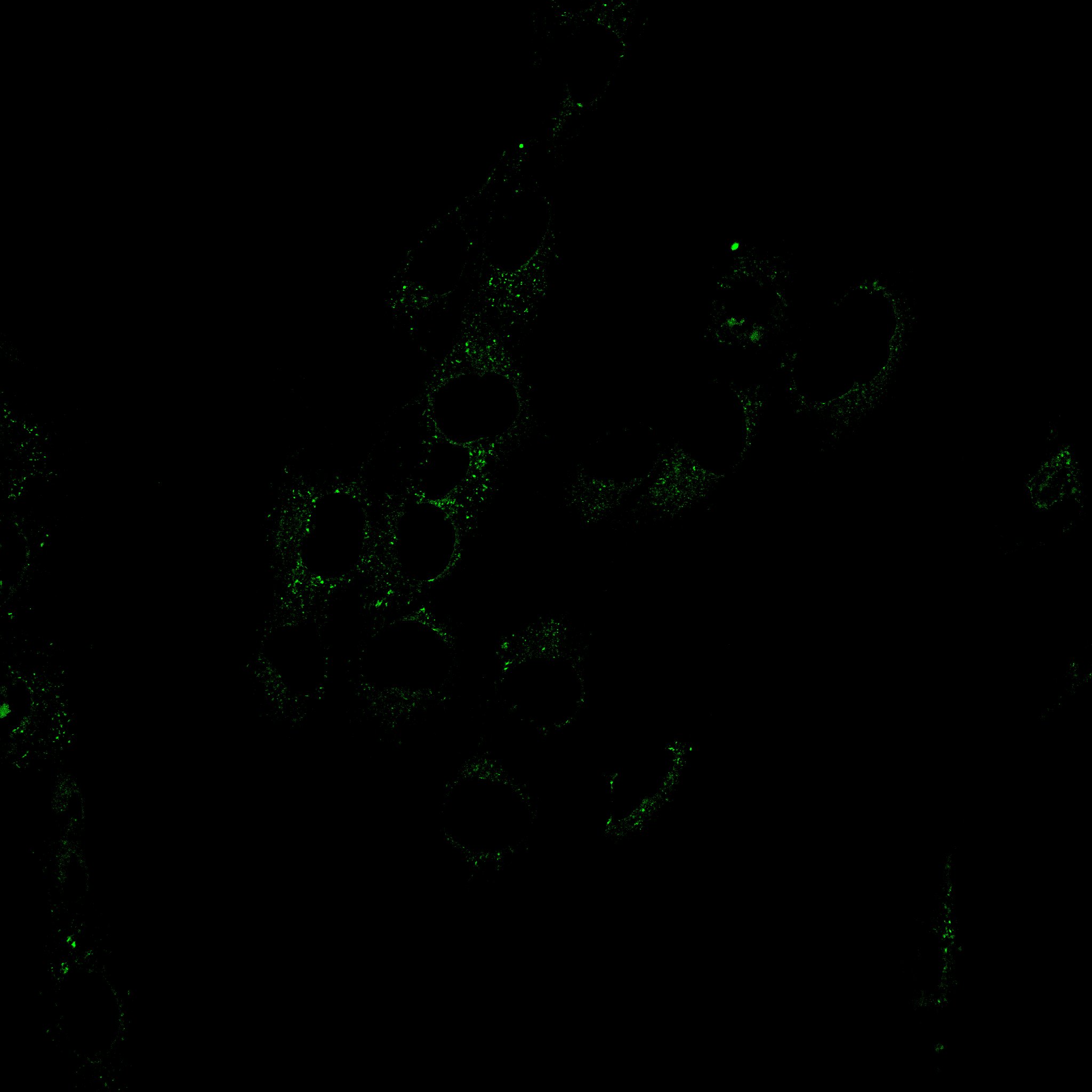

Supplement: Supplementary file 7 — Source data Fig. 5 [file 44318_2025_570_MOESM7_ESM.zip › Fig5/Images/P/Fig_5_panel_p_NTa_5_green.jpg]

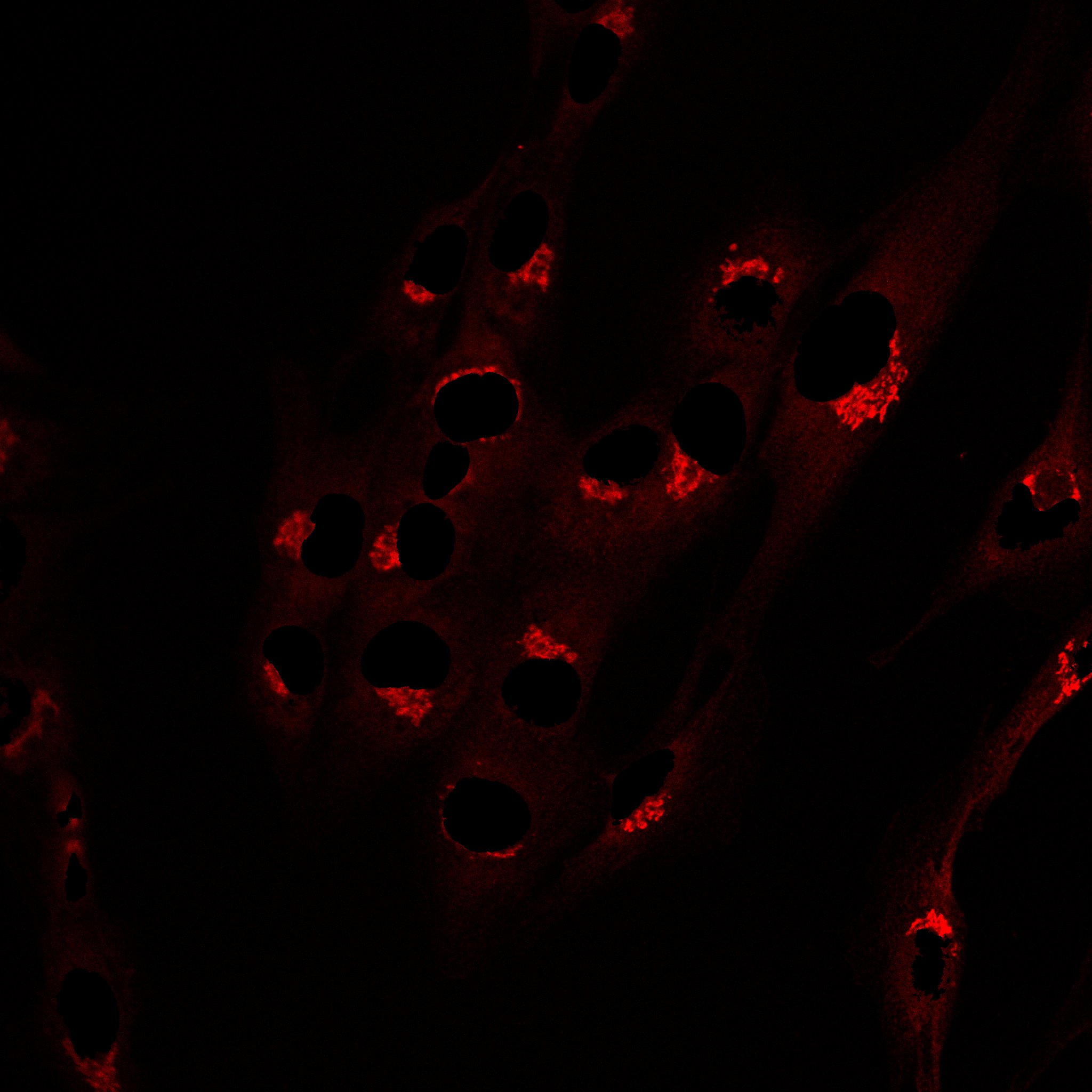

Supplement: Supplementary file 7 — Source data Fig. 5 [file 44318_2025_570_MOESM7_ESM.zip › Fig5/Images/P/Fig_5_panel_p_NTa_5_red.jpg]

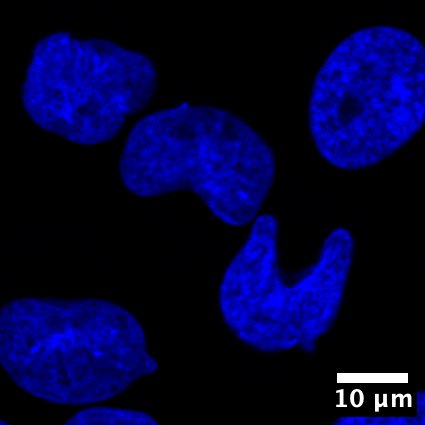

Supplement: Supplementary file 7 — Source data Fig. 5 [file 44318_2025_570_MOESM7_ESM.zip › Fig5/Images/P/Fig_5_panel_p_SH1A_blue_Big.jpg]

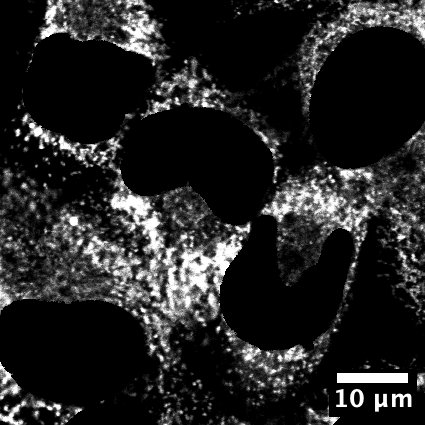

Supplement: Supplementary file 7 — Source data Fig. 5 [file 44318_2025_570_MOESM7_ESM.zip › Fig5/Images/P/Fig_5_panel_p_SH1A_gray_Big.jpg]

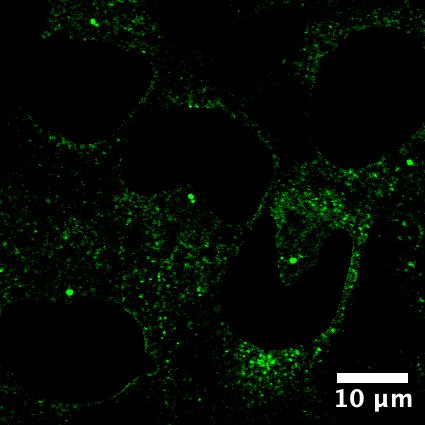

Supplement: Supplementary file 7 — Source data Fig. 5 [file 44318_2025_570_MOESM7_ESM.zip › Fig5/Images/P/Fig_5_panel_p_SH1A_green_Big.jpg]

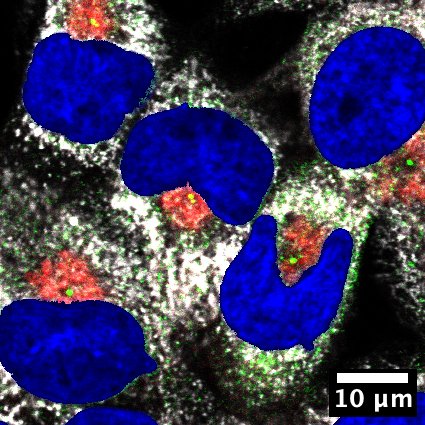

Supplement: Supplementary file 7 — Source data Fig. 5 [file 44318_2025_570_MOESM7_ESM.zip › Fig5/Images/P/Fig_5_panel_p_SH1A_merge_Big.jpg]

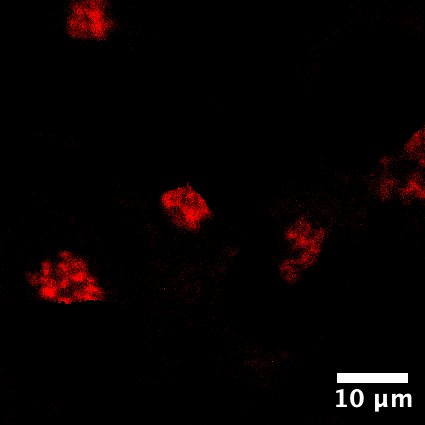

Supplement: Supplementary file 7 — Source data Fig. 5 [file 44318_2025_570_MOESM7_ESM.zip › Fig5/Images/P/Fig_5_panel_p_SH1A_red_Big.jpg]

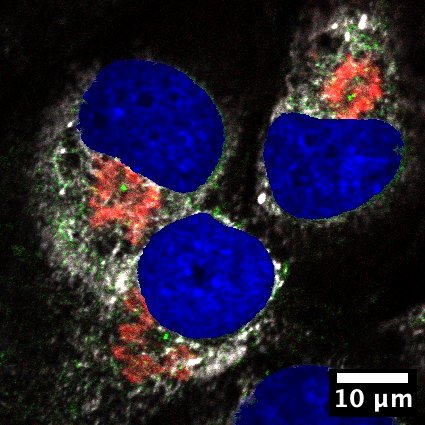

Supplement: Supplementary file 7 — Source data Fig. 5 [file 44318_2025_570_MOESM7_ESM.zip › Fig5/Images/P/Fig_5_panel_p_SH5A_merge_Big.jpg]

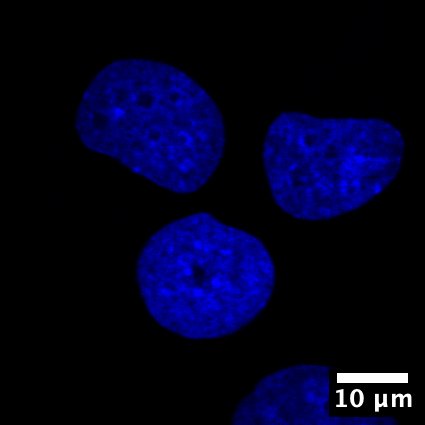

Supplement: Supplementary file 7 — Source data Fig. 5 [file 44318_2025_570_MOESM7_ESM.zip › Fig5/Images/P/Fig_5_panel_p_SH5a_blue_Big.jpg]

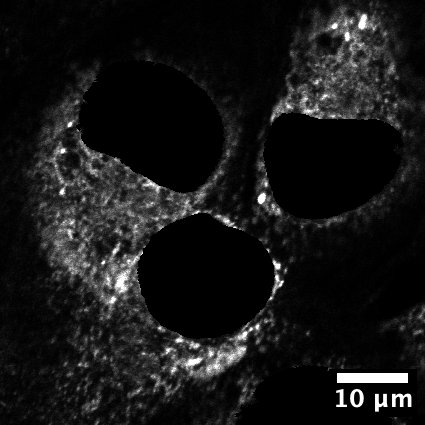

Supplement: Supplementary file 7 — Source data Fig. 5 [file 44318_2025_570_MOESM7_ESM.zip › Fig5/Images/P/Fig_5_panel_p_SH5a_gray_Big.jpg]

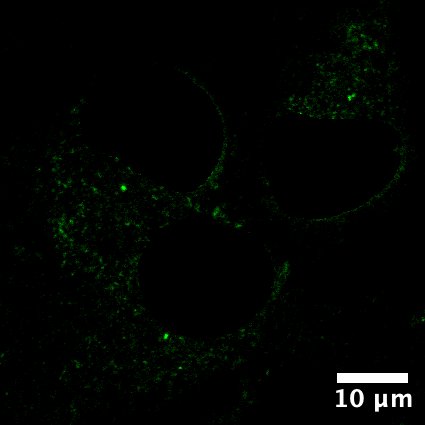

Supplement: Supplementary file 7 — Source data Fig. 5 [file 44318_2025_570_MOESM7_ESM.zip › Fig5/Images/P/Fig_5_panel_p_SH5a_green_Big.jpg]

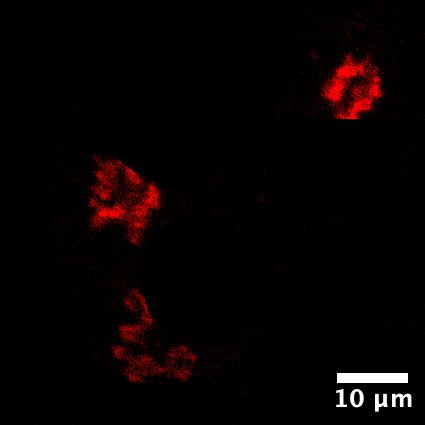

Supplement: Supplementary file 7 — Source data Fig. 5 [file 44318_2025_570_MOESM7_ESM.zip › Fig5/Images/P/Fig_5_panel_p_SH5a_red_Big.jpg]

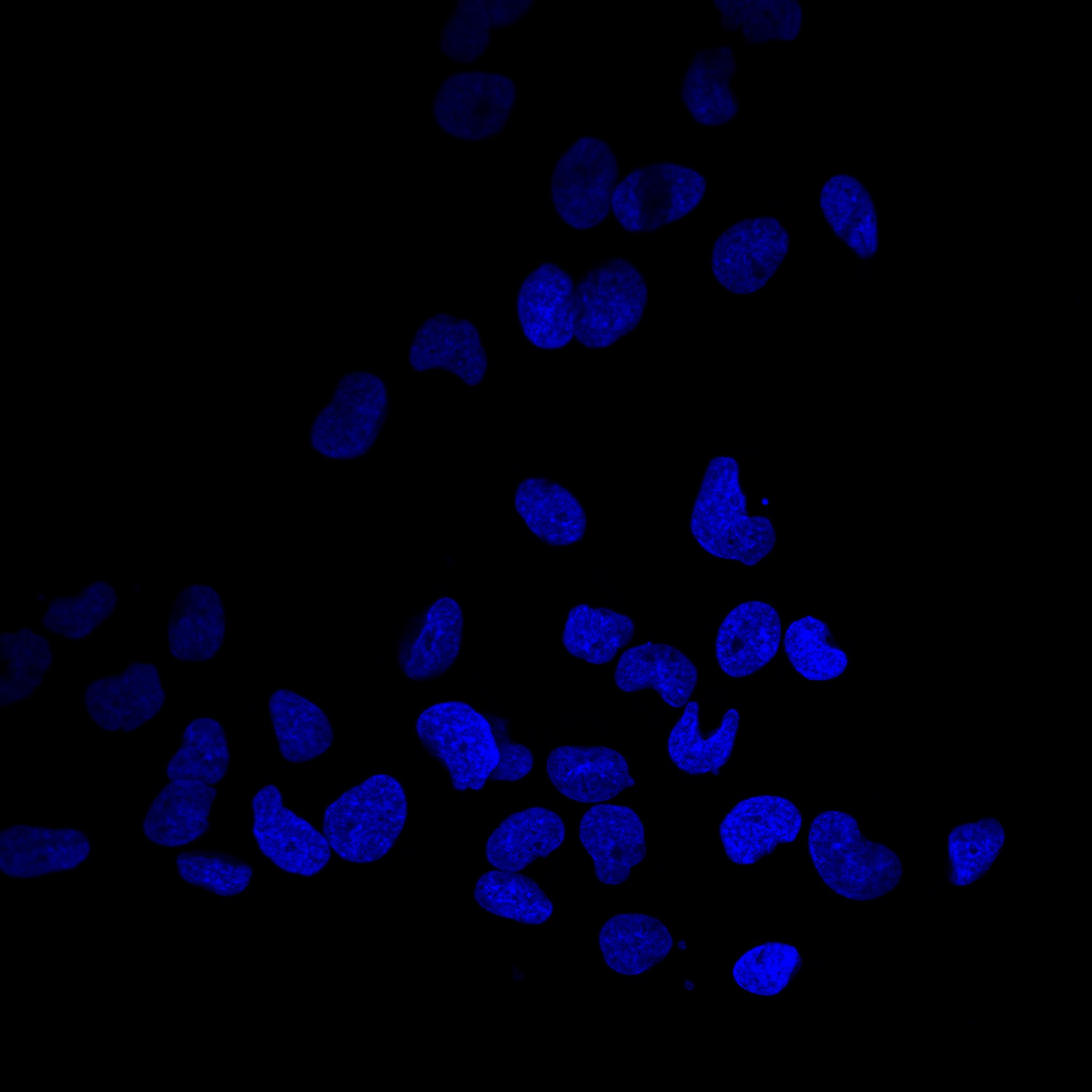

Supplement: Supplementary file 7 — Source data Fig. 5 [file 44318_2025_570_MOESM7_ESM.zip › Fig5/Images/P/Fig_5_panel_p_sh1a_1_blue.jpg]

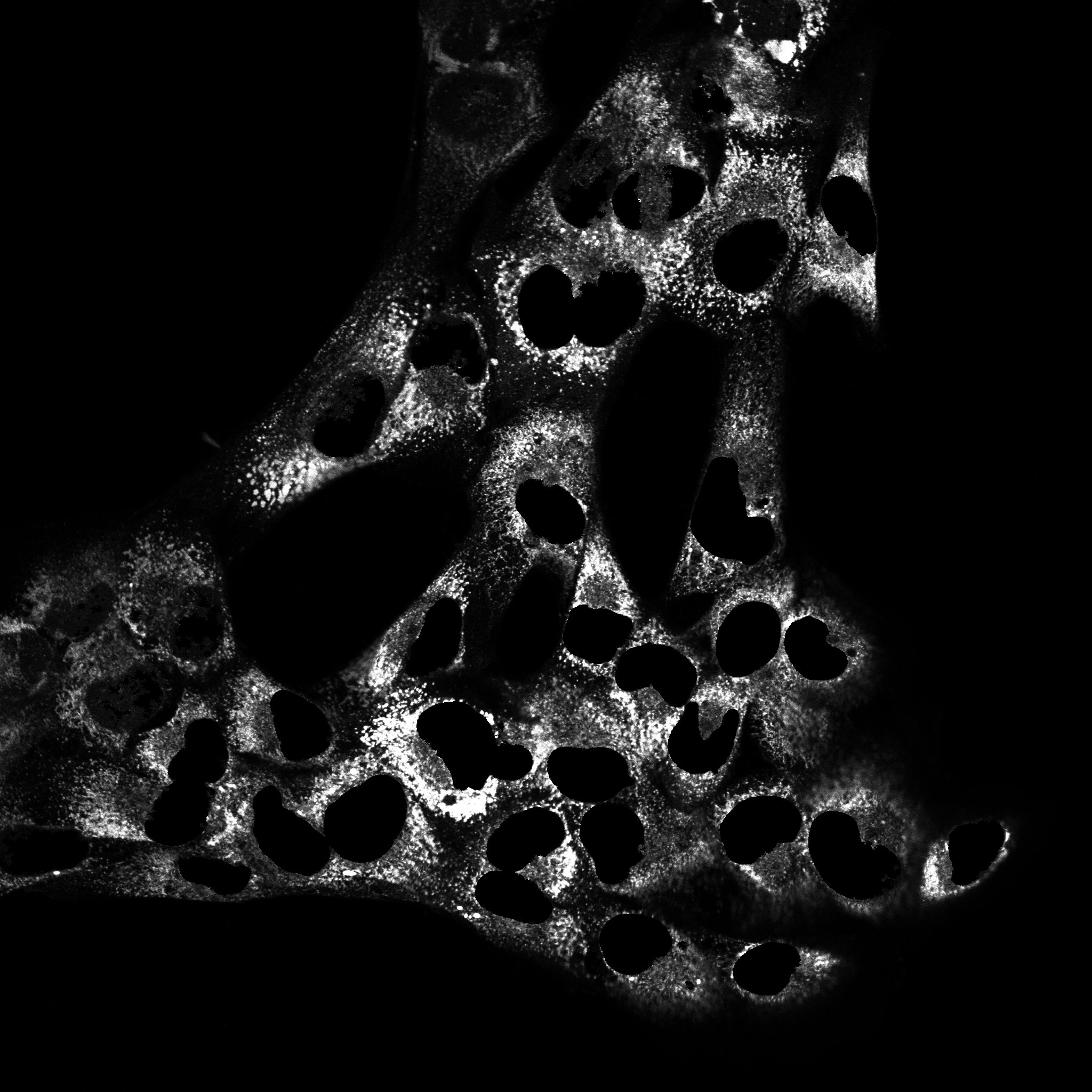

Supplement: Supplementary file 7 — Source data Fig. 5 [file 44318_2025_570_MOESM7_ESM.zip › Fig5/Images/P/Fig_5_panel_p_sh1a_1_gray.jpg]

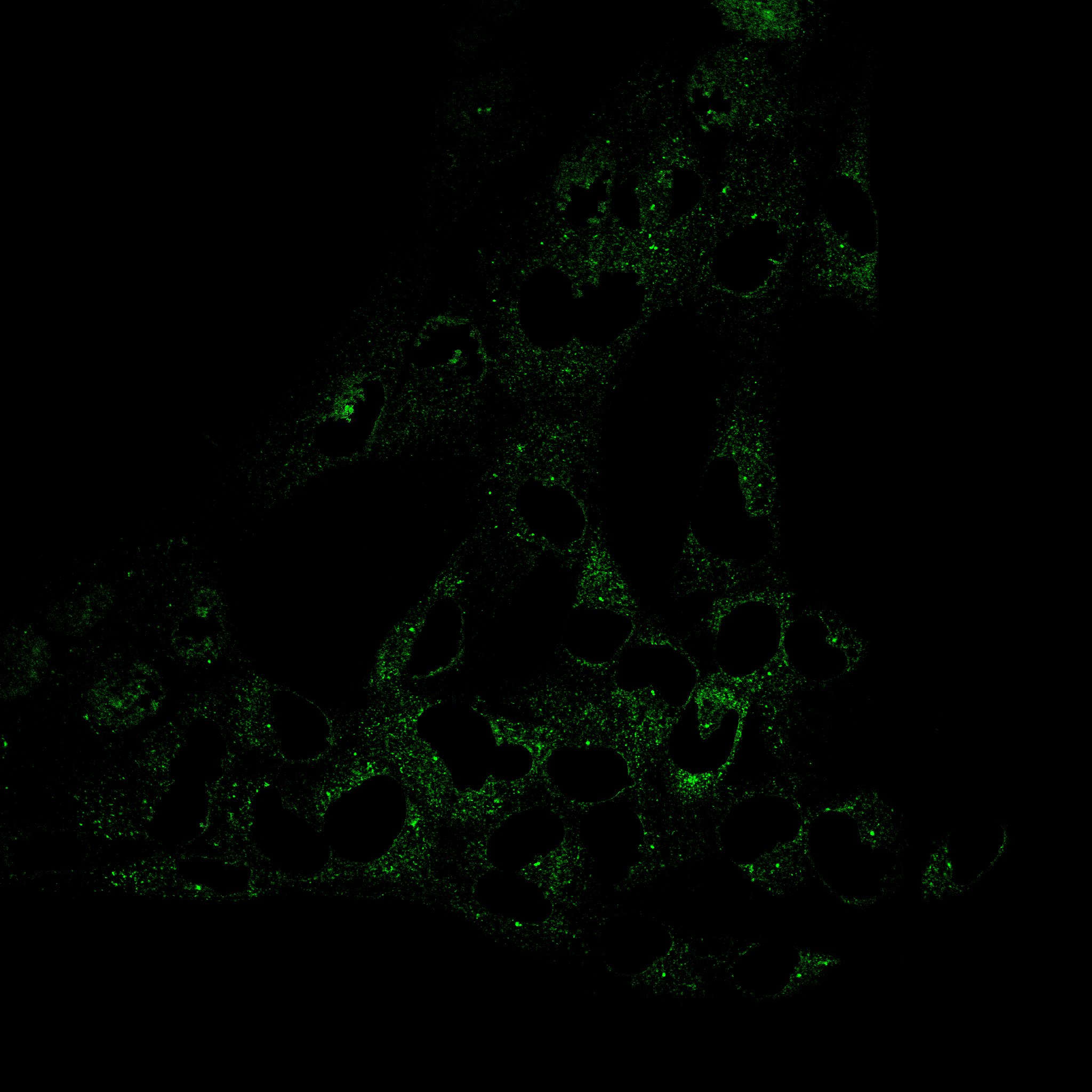

Supplement: Supplementary file 7 — Source data Fig. 5 [file 44318_2025_570_MOESM7_ESM.zip › Fig5/Images/P/Fig_5_panel_p_sh1a_1_green.jpg]

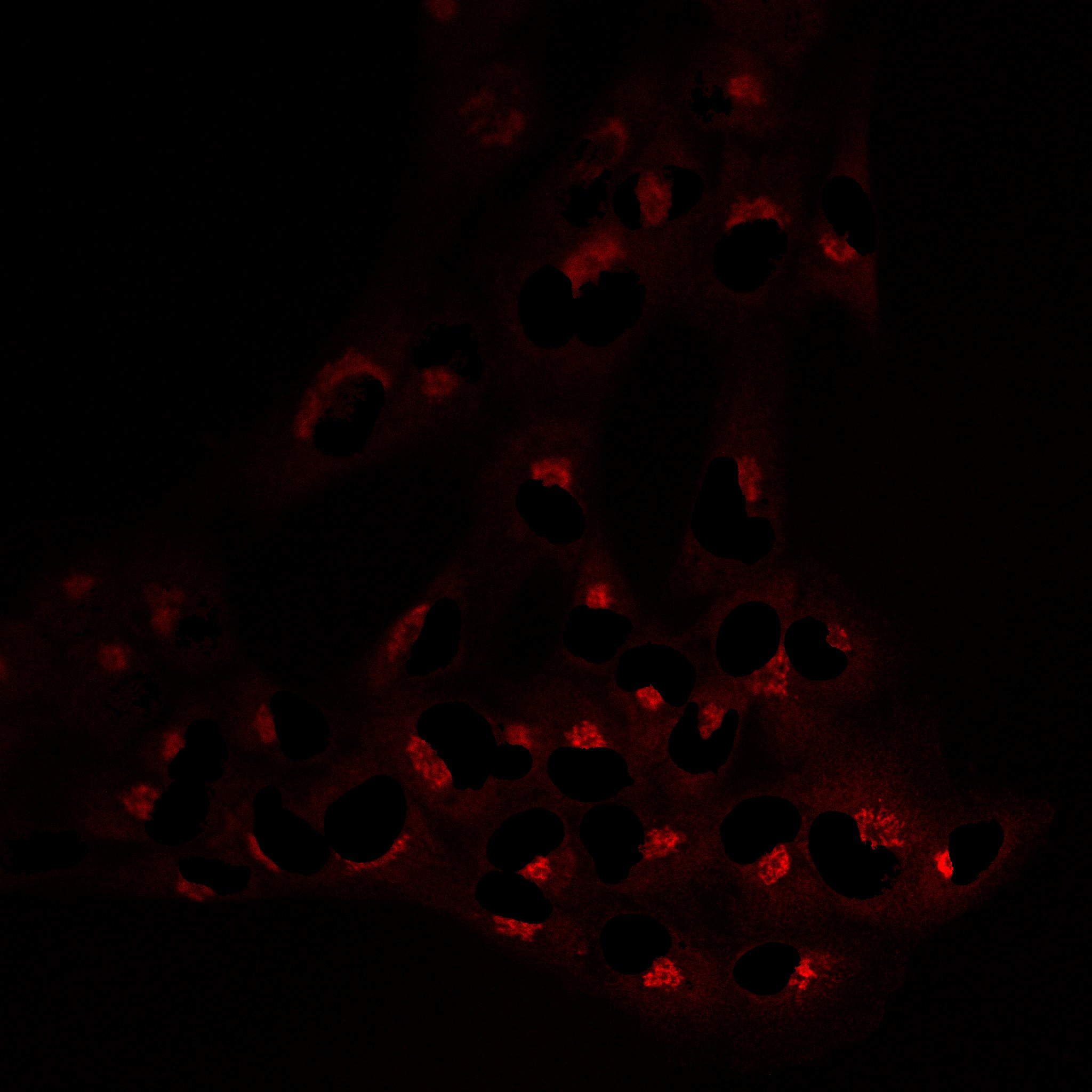

Supplement: Supplementary file 7 — Source data Fig. 5 [file 44318_2025_570_MOESM7_ESM.zip › Fig5/Images/P/Fig_5_panel_p_sh1a_1_red.jpg]

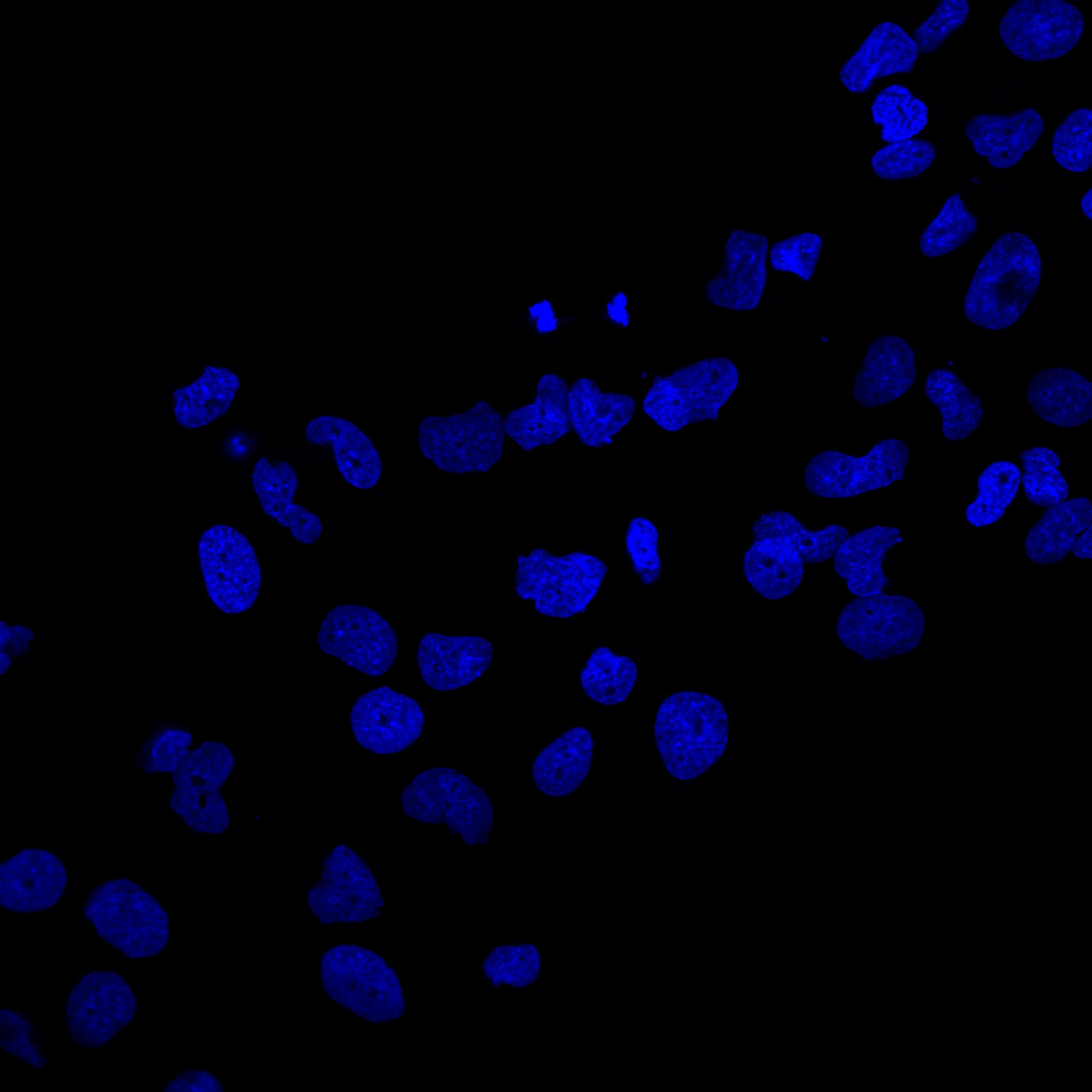

Supplement: Supplementary file 7 — Source data Fig. 5 [file 44318_2025_570_MOESM7_ESM.zip › Fig5/Images/P/Fig_5_panel_p_sh5a_1_blue.jpg]

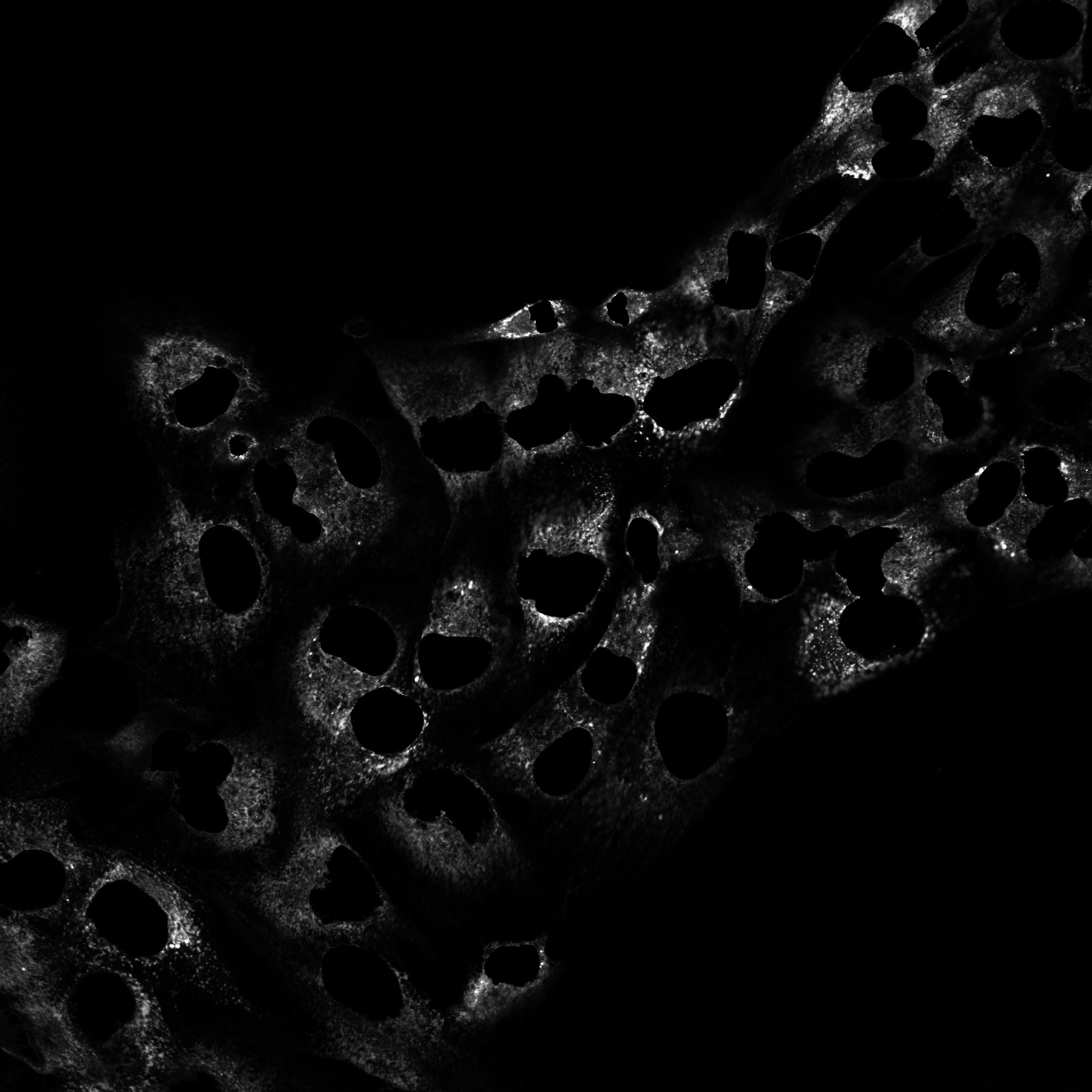

Supplement: Supplementary file 7 — Source data Fig. 5 [file 44318_2025_570_MOESM7_ESM.zip › Fig5/Images/P/Fig_5_panel_p_sh5a_1_gray.jpg]

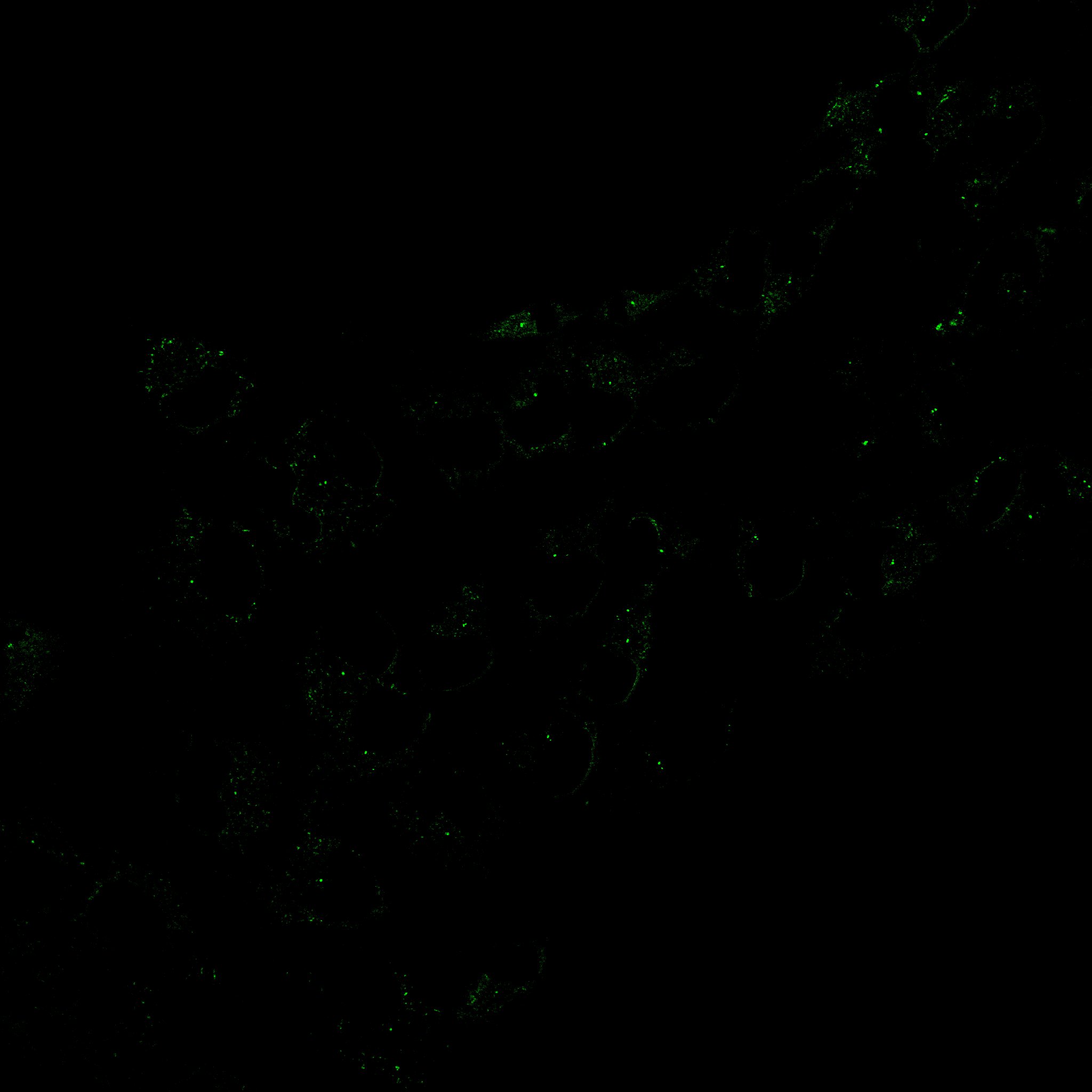

Supplement: Supplementary file 7 — Source data Fig. 5 [file 44318_2025_570_MOESM7_ESM.zip › Fig5/Images/P/Fig_5_panel_p_sh5a_1_green.jpg]

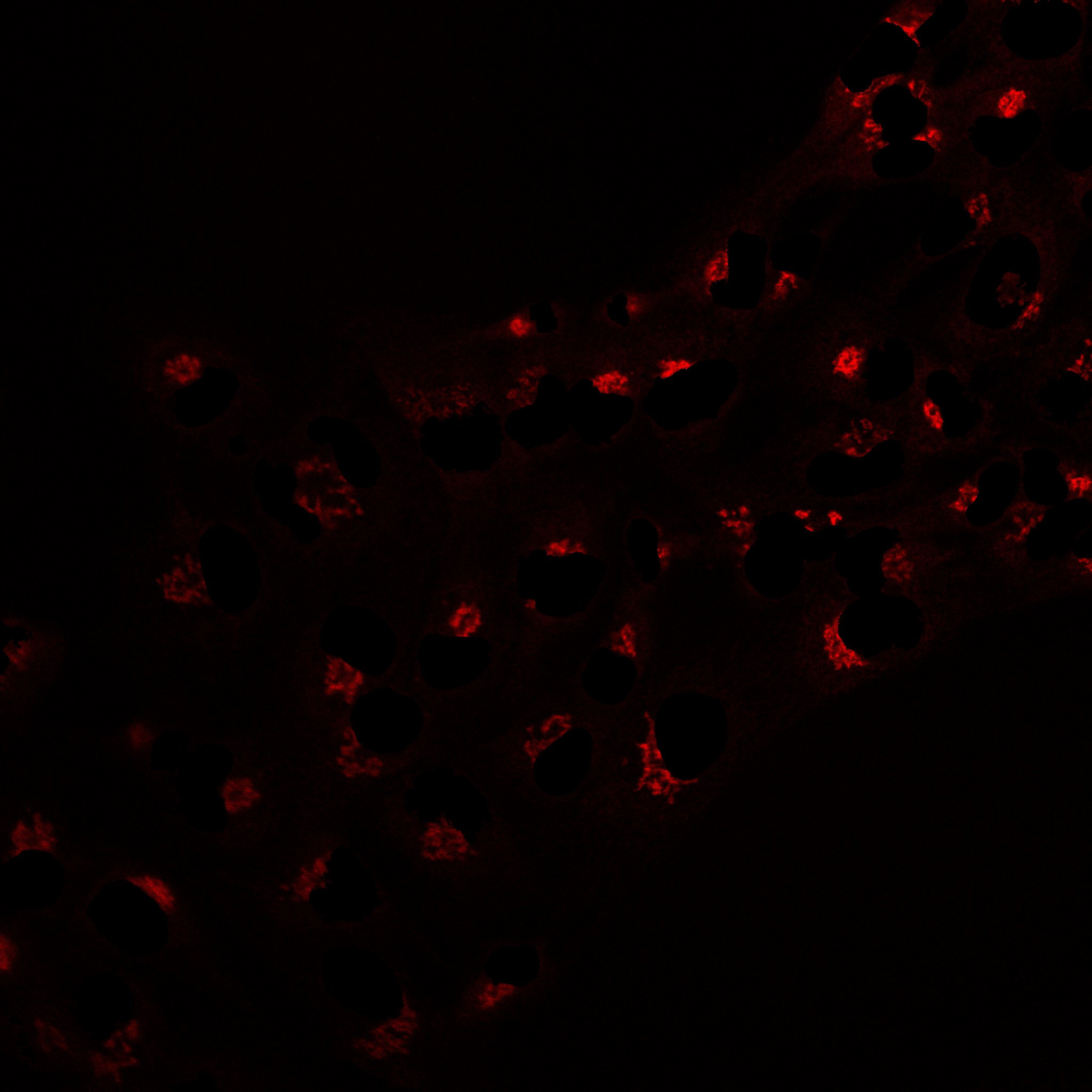

Supplement: Supplementary file 7 — Source data Fig. 5 [file 44318_2025_570_MOESM7_ESM.zip › Fig5/Images/P/Fig_5_panel_p_sh5a_1_red.jpg]

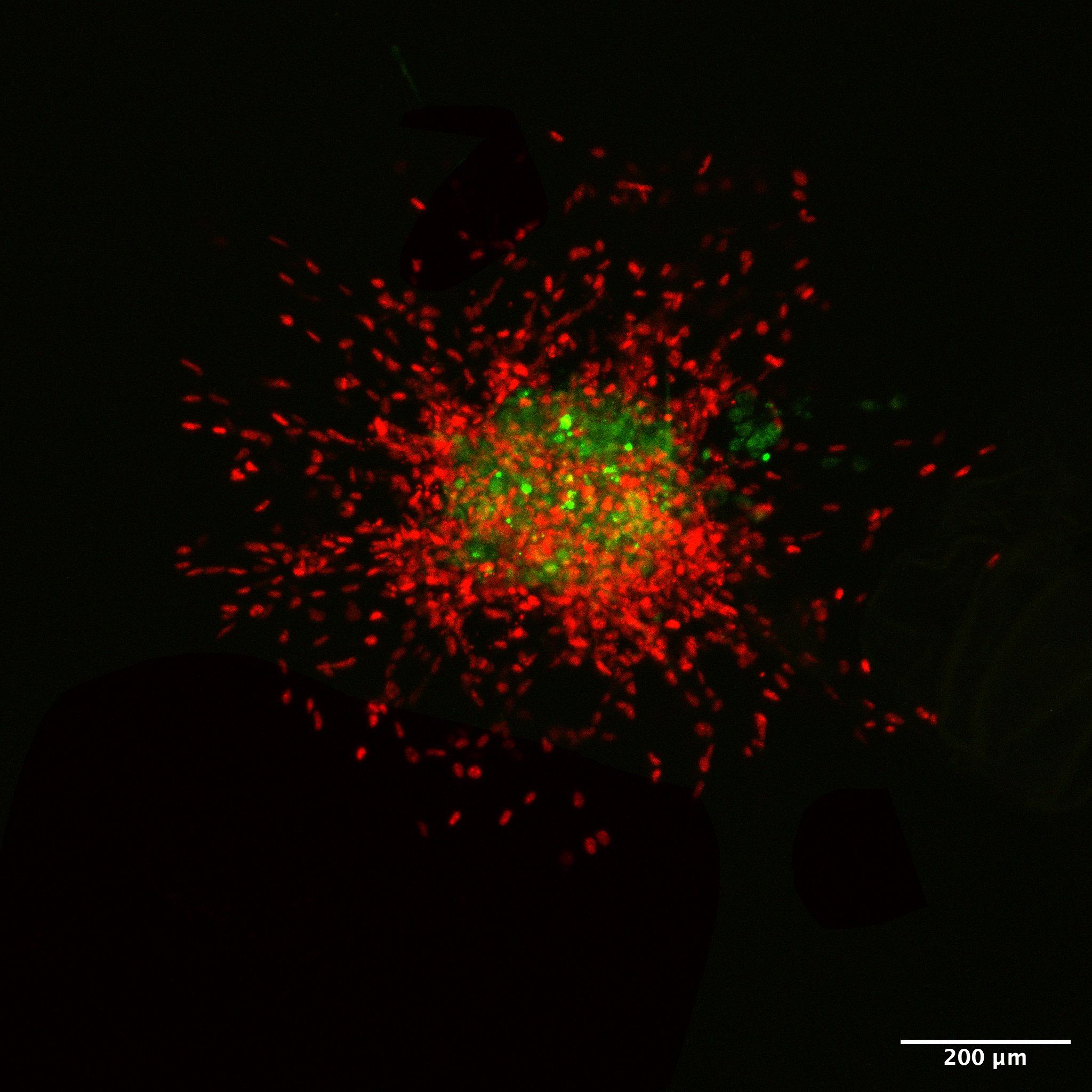

Supplement: Supplementary file 8 — Source data Fig. 6 [file 44318_2025_570_MOESM8_ESM.zip › Fig6/Images/C/Fig_6_panel_c_MAX_RP244_sh#1_001.jpg]

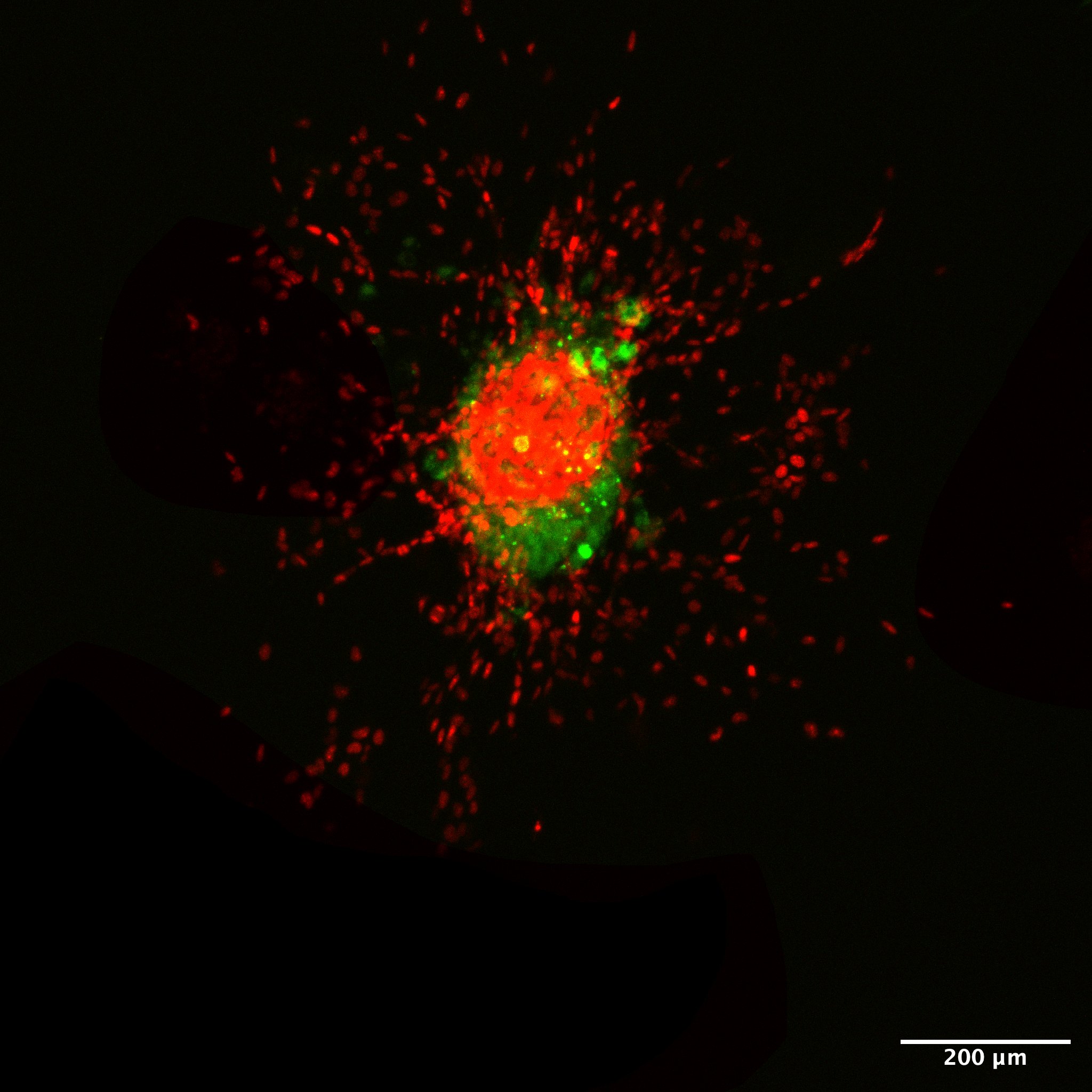

Supplement: Supplementary file 8 — Source data Fig. 6 [file 44318_2025_570_MOESM8_ESM.zip › Fig6/Images/C/Fig_6_panel_c_MAX_RP244_sh#5_005.jpg]

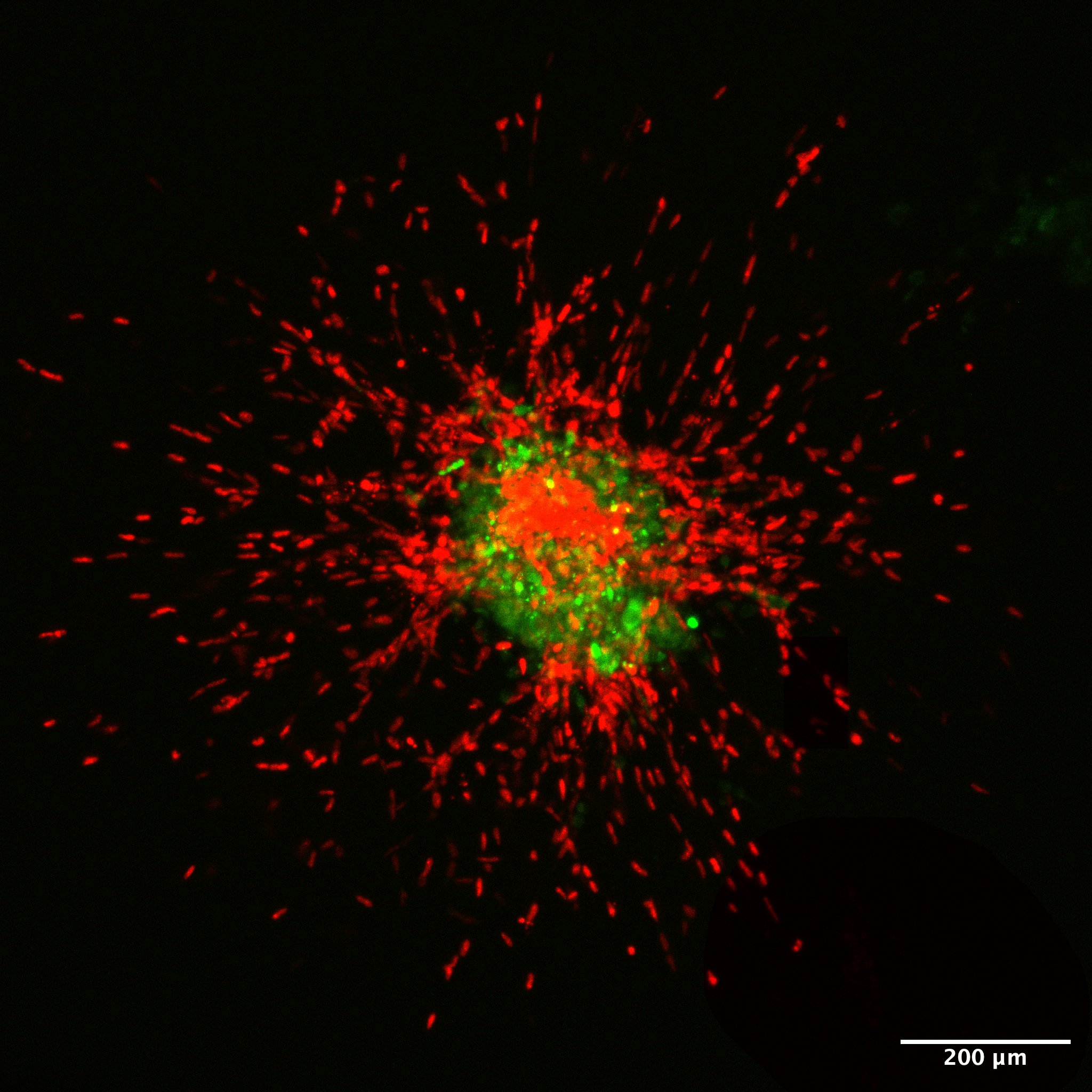

Supplement: Supplementary file 8 — Source data Fig. 6 [file 44318_2025_570_MOESM8_ESM.zip › Fig6/Images/C/Fig_6_panel_c_MAX_RP244_shNT_007.jpg]

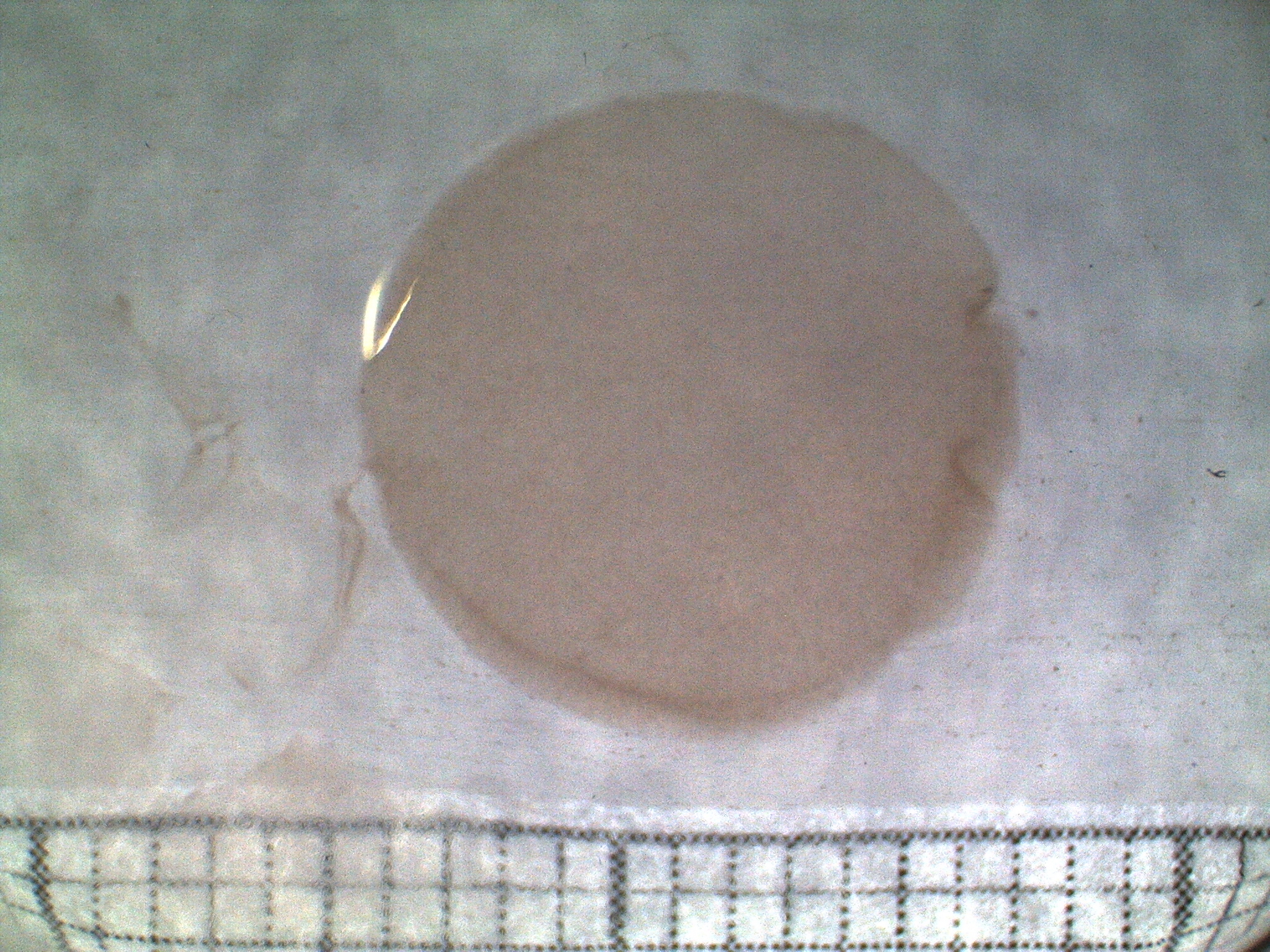

Supplement: Supplementary file 8 — Source data Fig. 6 [file 44318_2025_570_MOESM8_ESM.zip › Fig6/Images/F/Fig_6_panel_f_HPSC127 sh#1 + TGF.jpeg]

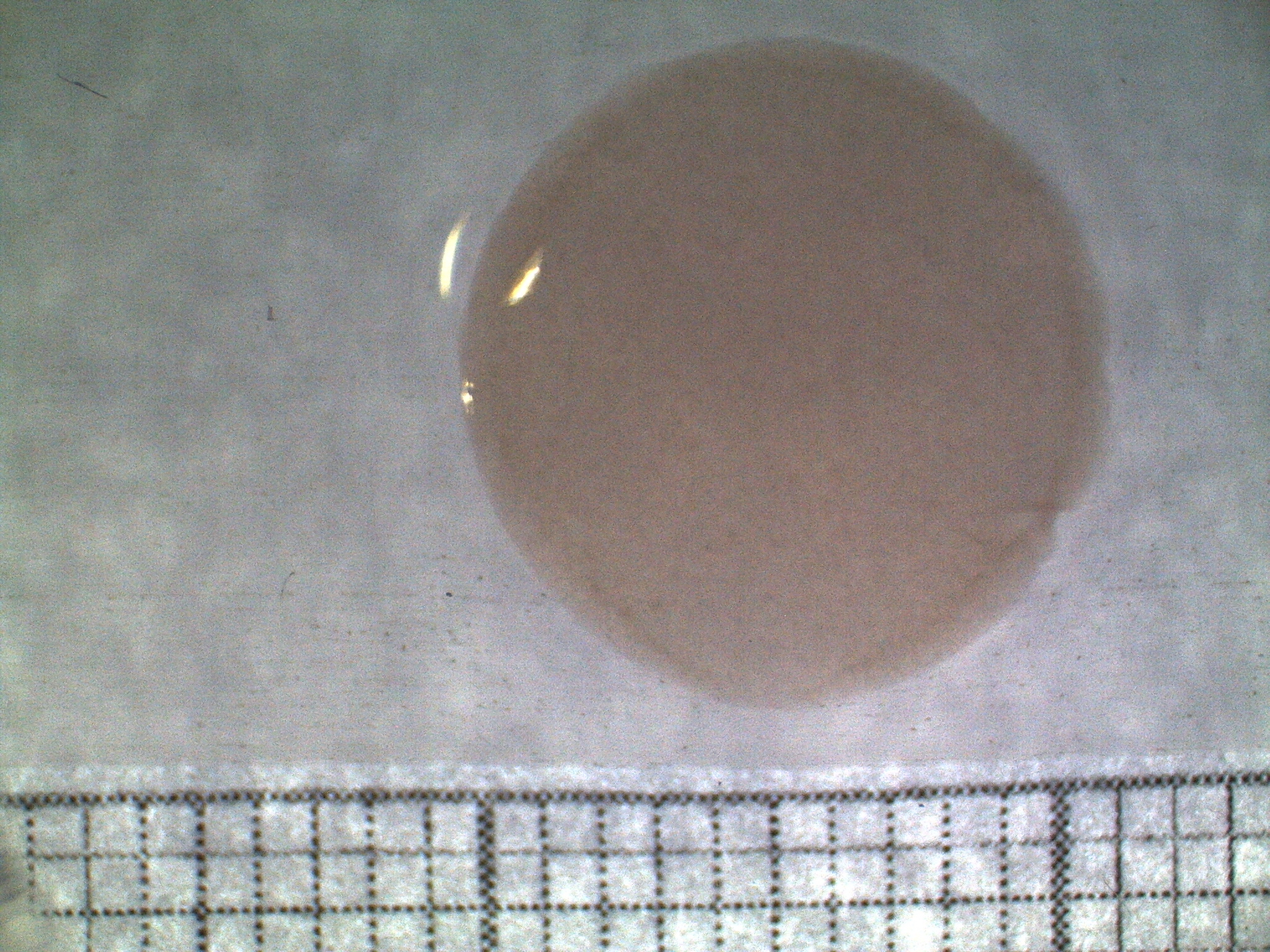

Supplement: Supplementary file 8 — Source data Fig. 6 [file 44318_2025_570_MOESM8_ESM.zip › Fig6/Images/F/Fig_6_panel_f_HPSC127 sh#5 + TGF.jpeg]

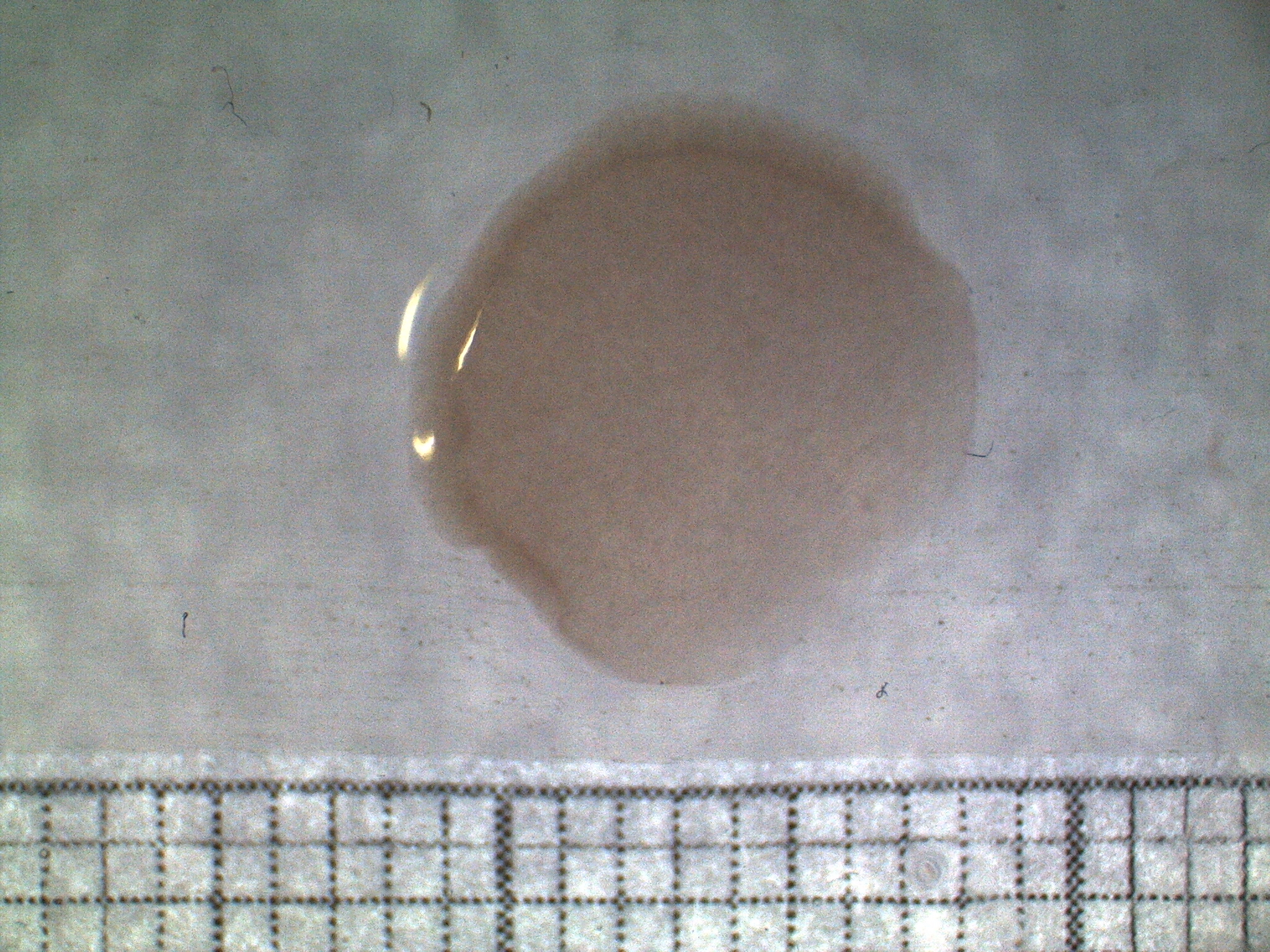

Supplement: Supplementary file 8 — Source data Fig. 6 [file 44318_2025_570_MOESM8_ESM.zip › Fig6/Images/F/Fig_6_panel_f_HPSC127 shNT + TGF.jpeg]

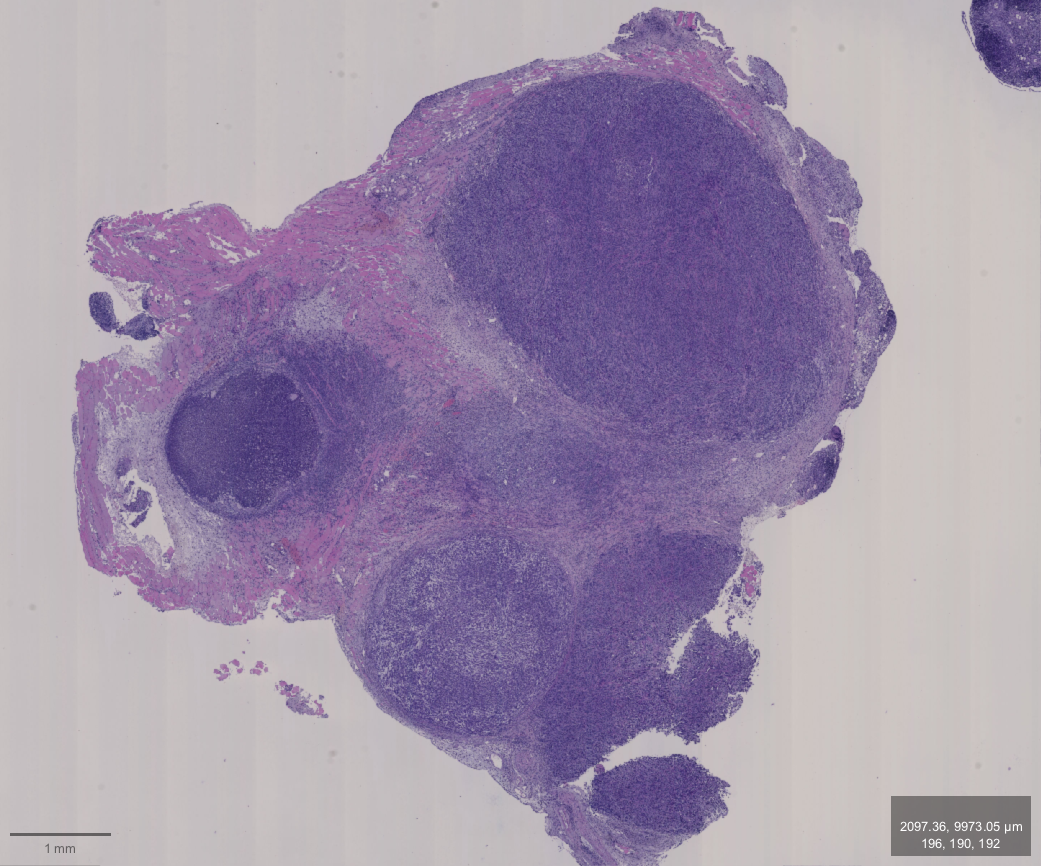

Supplement: Supplementary file 9 — Source data Fig. 7 [file 44318_2025_570_MOESM9_ESM.zip › Fig7/Images/C/Fig7_panel_c_image_1.tif]

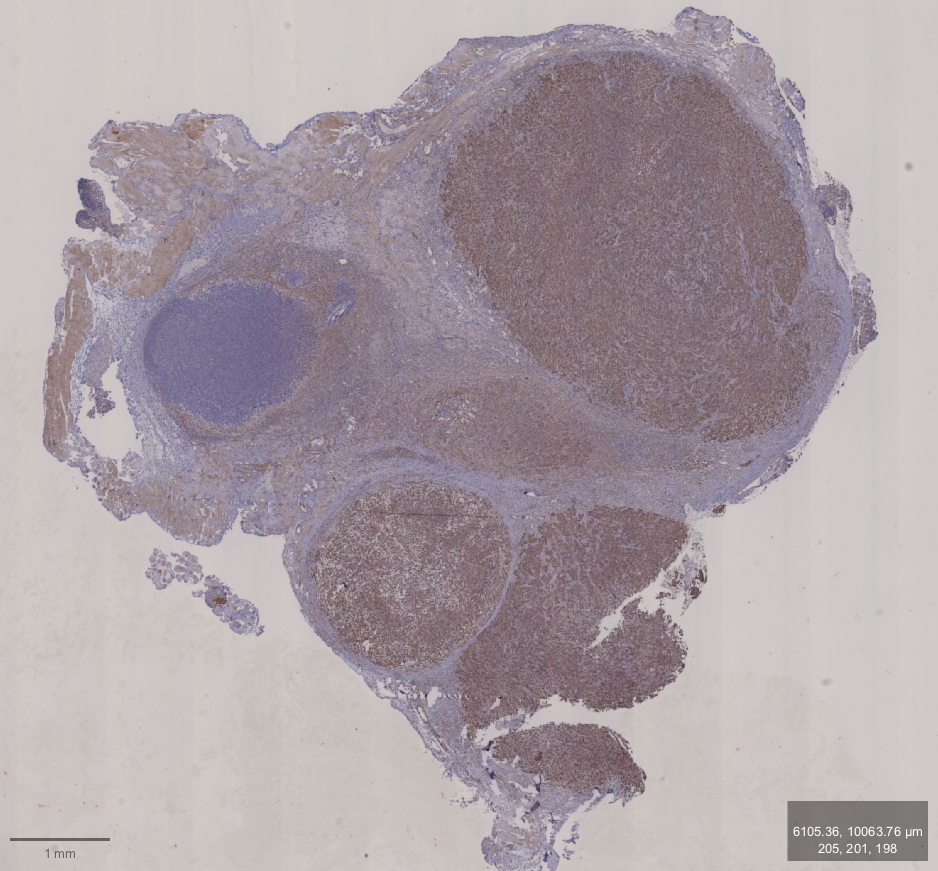

Supplement: Supplementary file 9 — Source data Fig. 7 [file 44318_2025_570_MOESM9_ESM.zip › Fig7/Images/C/Fig7_panel_c_image_2.tif]

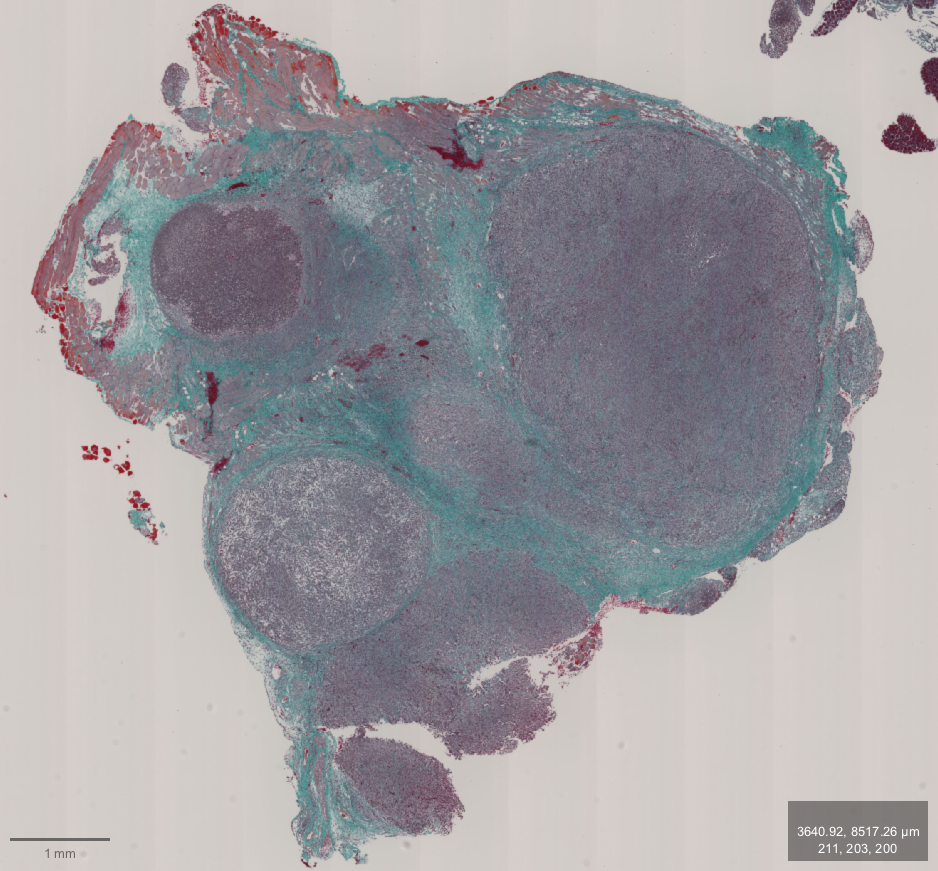

Supplement: Supplementary file 9 — Source data Fig. 7 [file 44318_2025_570_MOESM9_ESM.zip › Fig7/Images/C/Fig7_panel_c_image_3.tif]

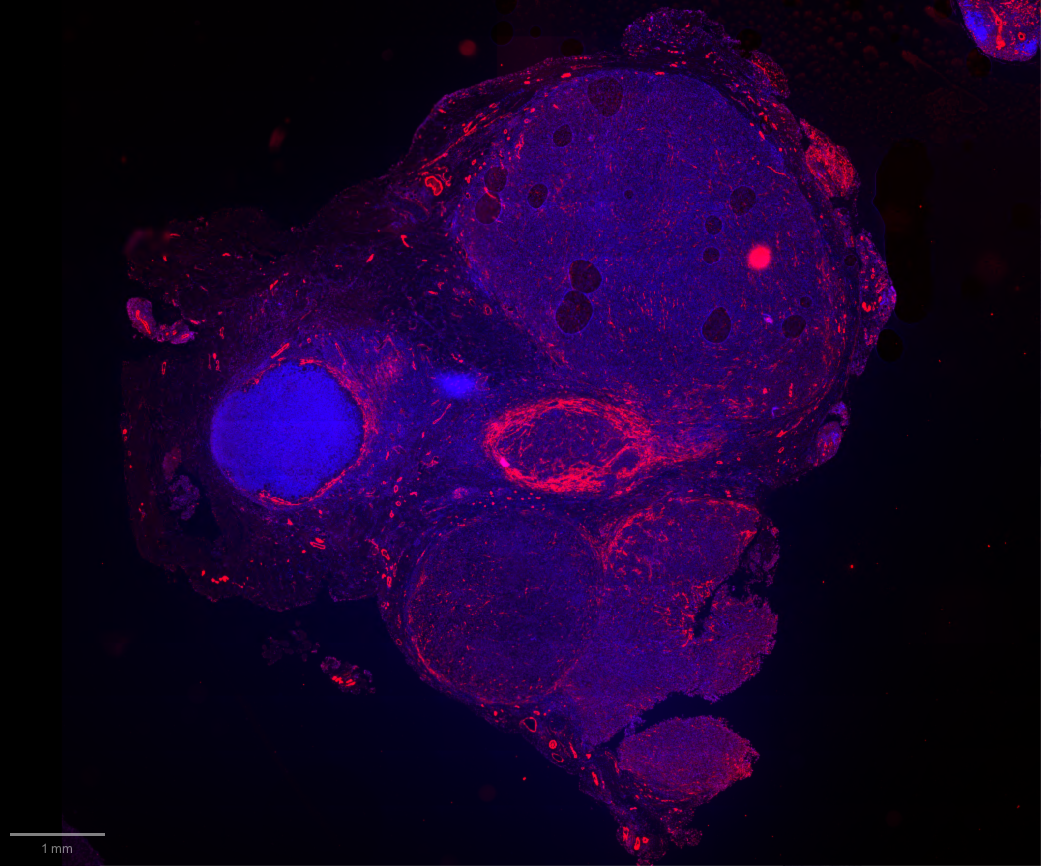

Supplement: Supplementary file 9 — Source data Fig. 7 [file 44318_2025_570_MOESM9_ESM.zip › Fig7/Images/C/Fig7_panel_c_image_4.tif]

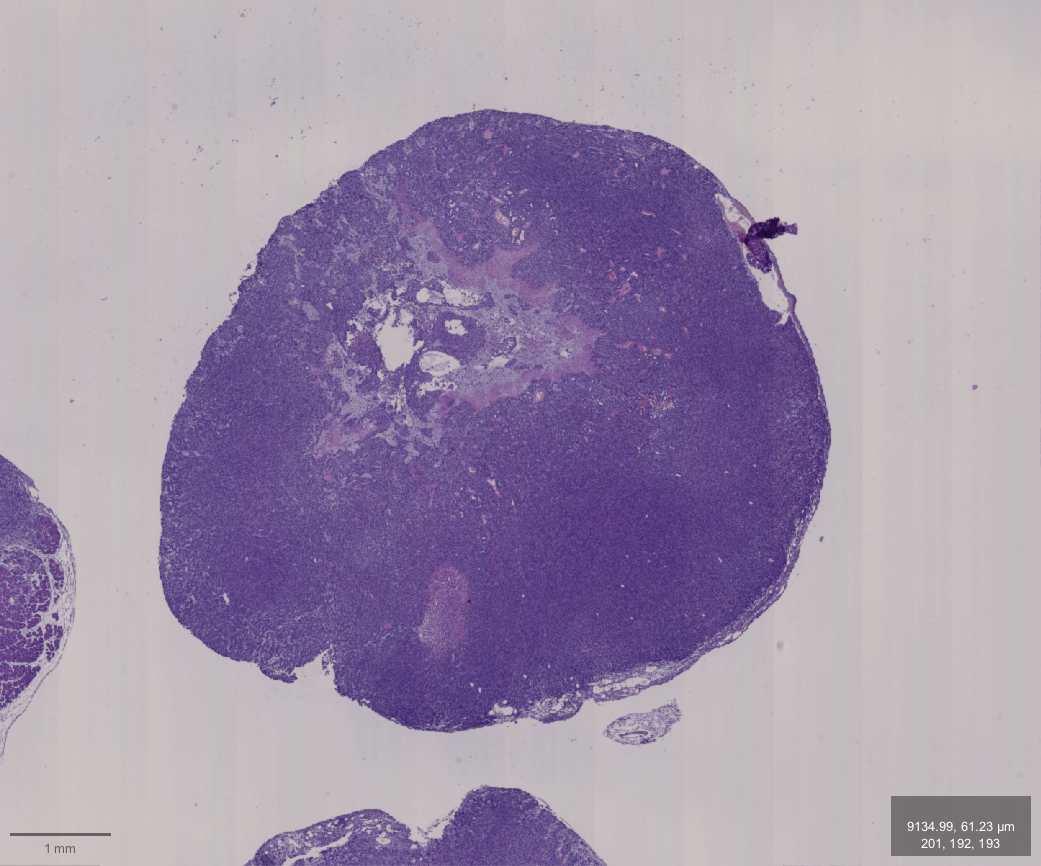

Supplement: Supplementary file 9 — Source data Fig. 7 [file 44318_2025_570_MOESM9_ESM.zip › Fig7/Images/C/Fig7_panel_c_image_5.tif]

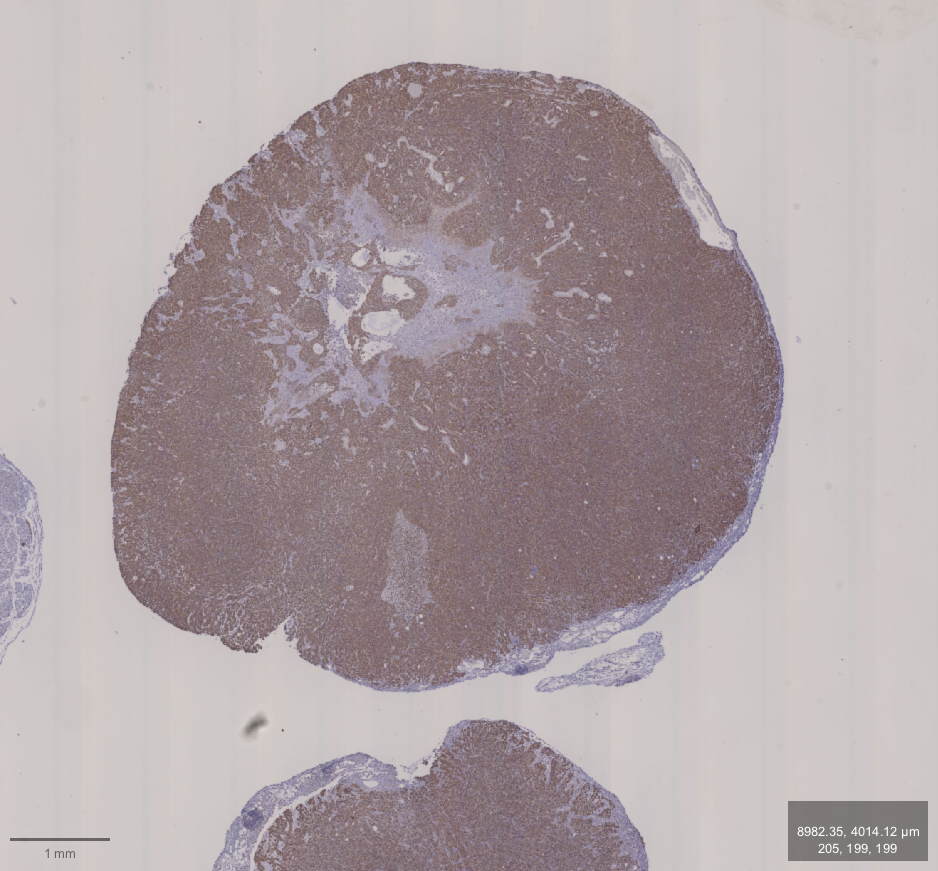

Supplement: Supplementary file 9 — Source data Fig. 7 [file 44318_2025_570_MOESM9_ESM.zip › Fig7/Images/C/Fig7_panel_c_image_6.tif]

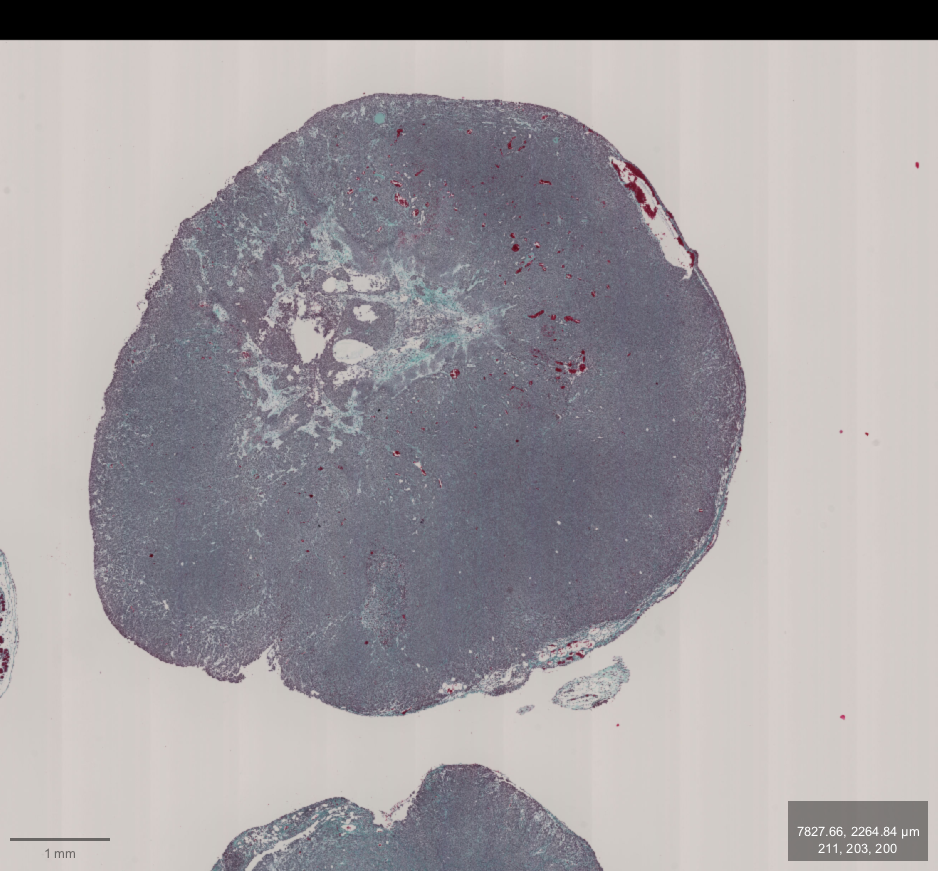

Supplement: Supplementary file 9 — Source data Fig. 7 [file 44318_2025_570_MOESM9_ESM.zip › Fig7/Images/C/Fig7_panel_c_image_7.tif]

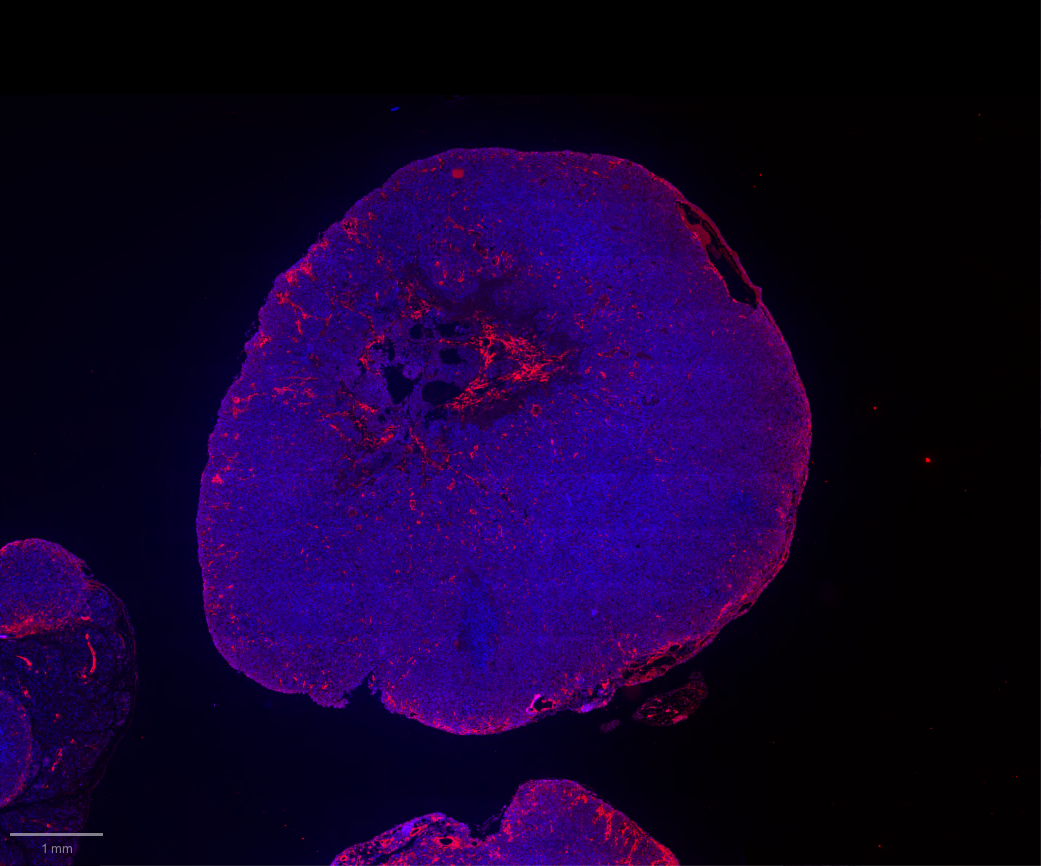

Supplement: Supplementary file 9 — Source data Fig. 7 [file 44318_2025_570_MOESM9_ESM.zip › Fig7/Images/C/Fig7_panel_c_image_8.tif]

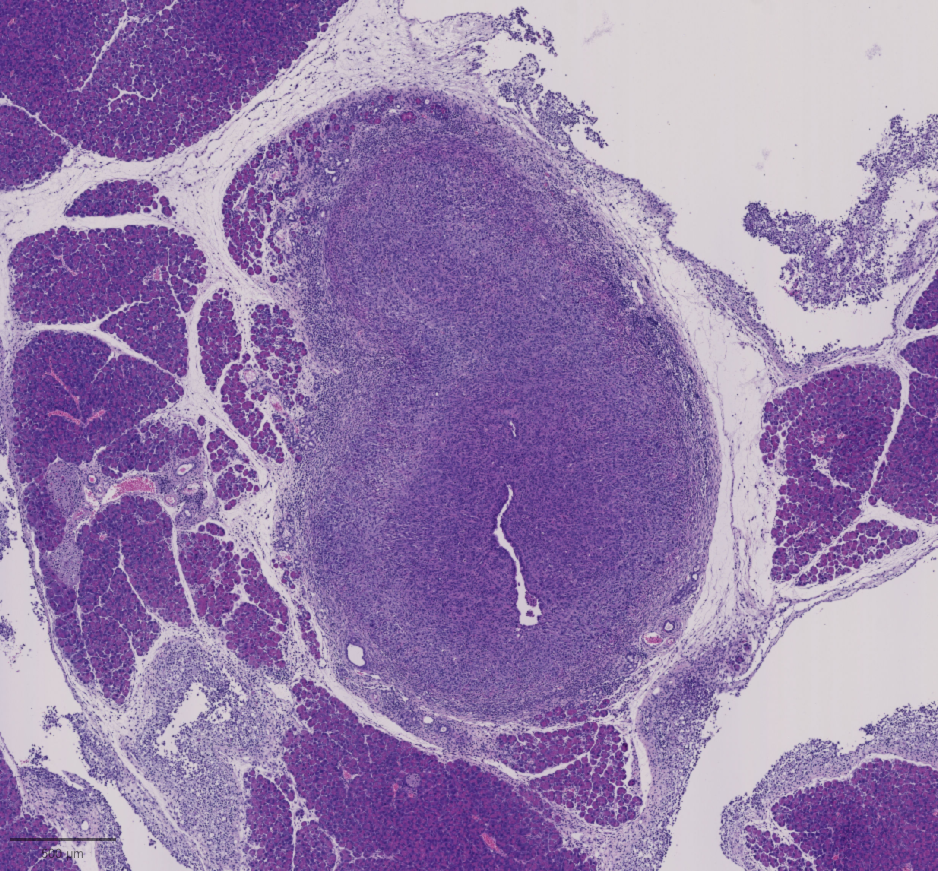

Supplement: Supplementary file 9 — Source data Fig. 7 [file 44318_2025_570_MOESM9_ESM.zip › Fig7/Images/H/Fig7_panel_h_image_1.tif]

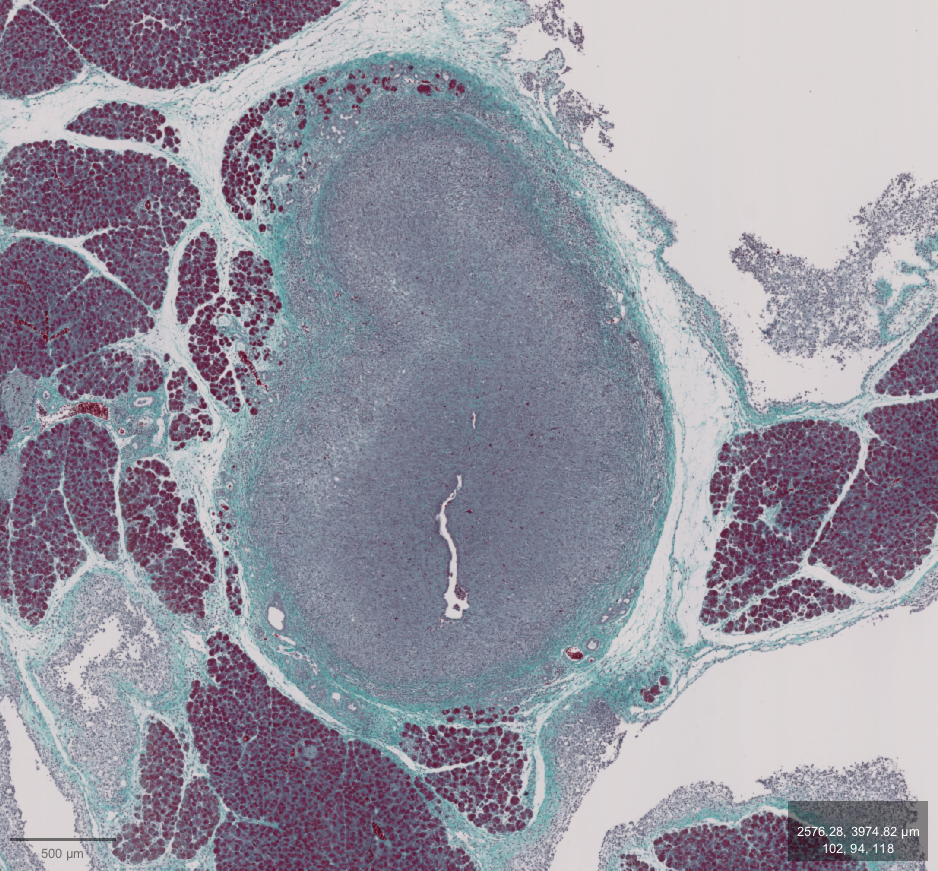

Supplement: Supplementary file 9 — Source data Fig. 7 [file 44318_2025_570_MOESM9_ESM.zip › Fig7/Images/H/Fig7_panel_h_image_2.tif]

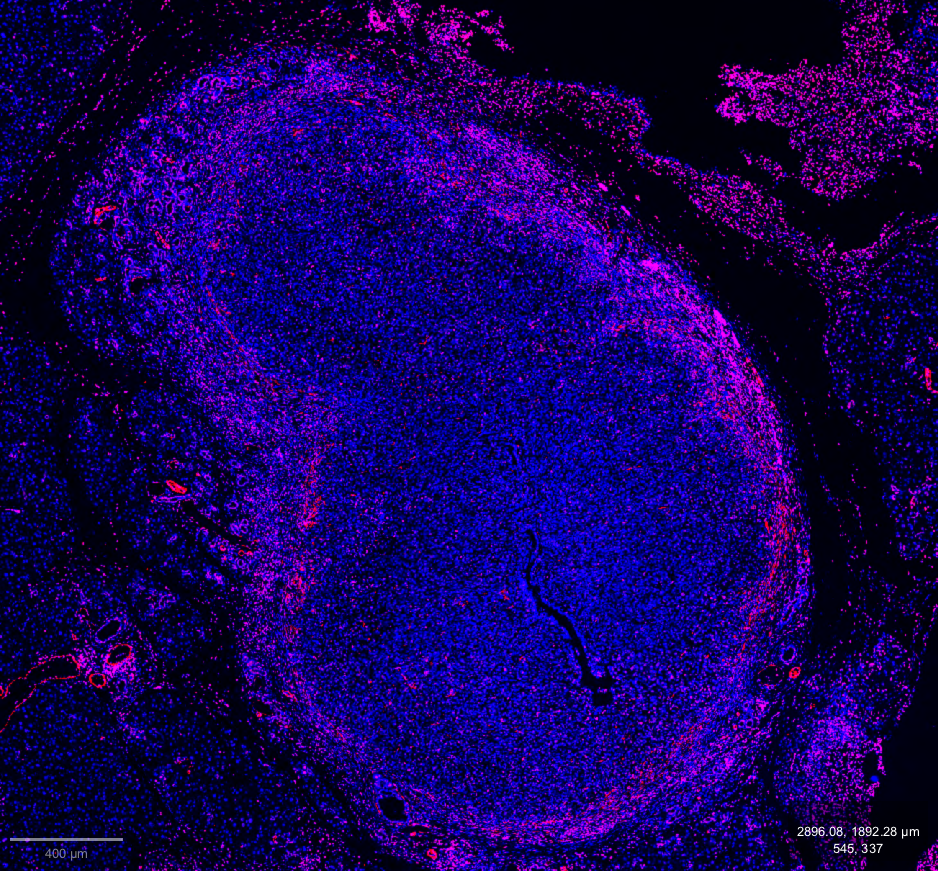

Supplement: Supplementary file 9 — Source data Fig. 7 [file 44318_2025_570_MOESM9_ESM.zip › Fig7/Images/H/Fig7_panel_h_image_3.tif]

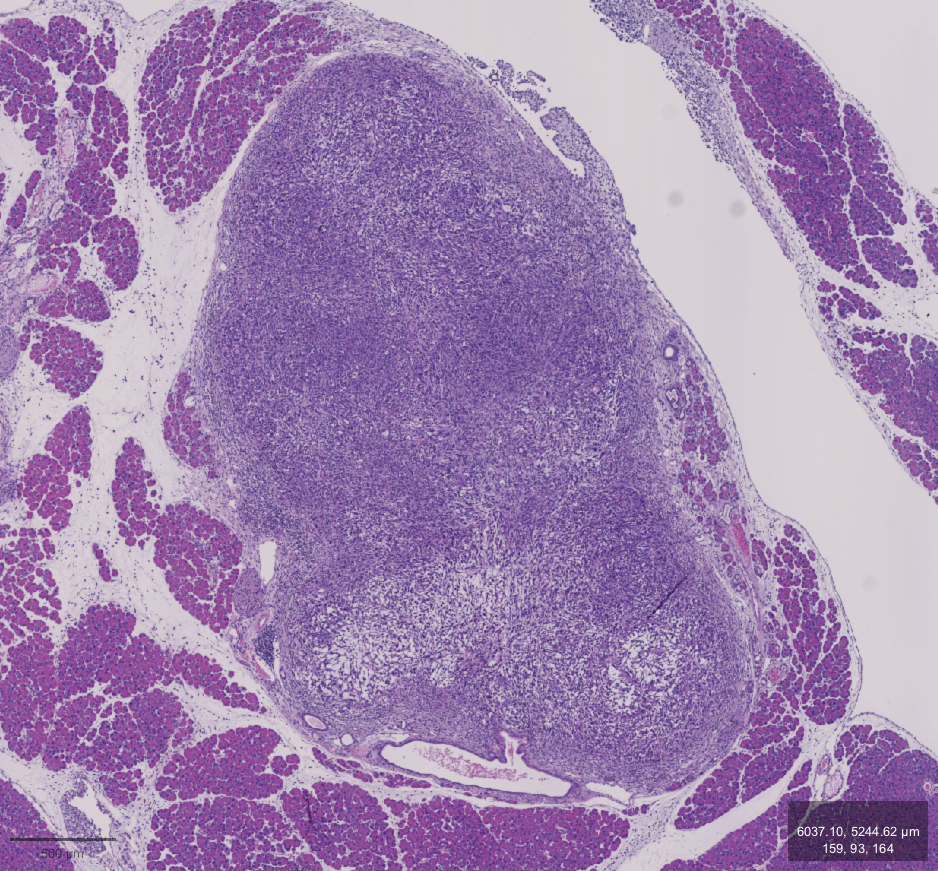

Supplement: Supplementary file 9 — Source data Fig. 7 [file 44318_2025_570_MOESM9_ESM.zip › Fig7/Images/H/Fig7_panel_h_image_4.tif]

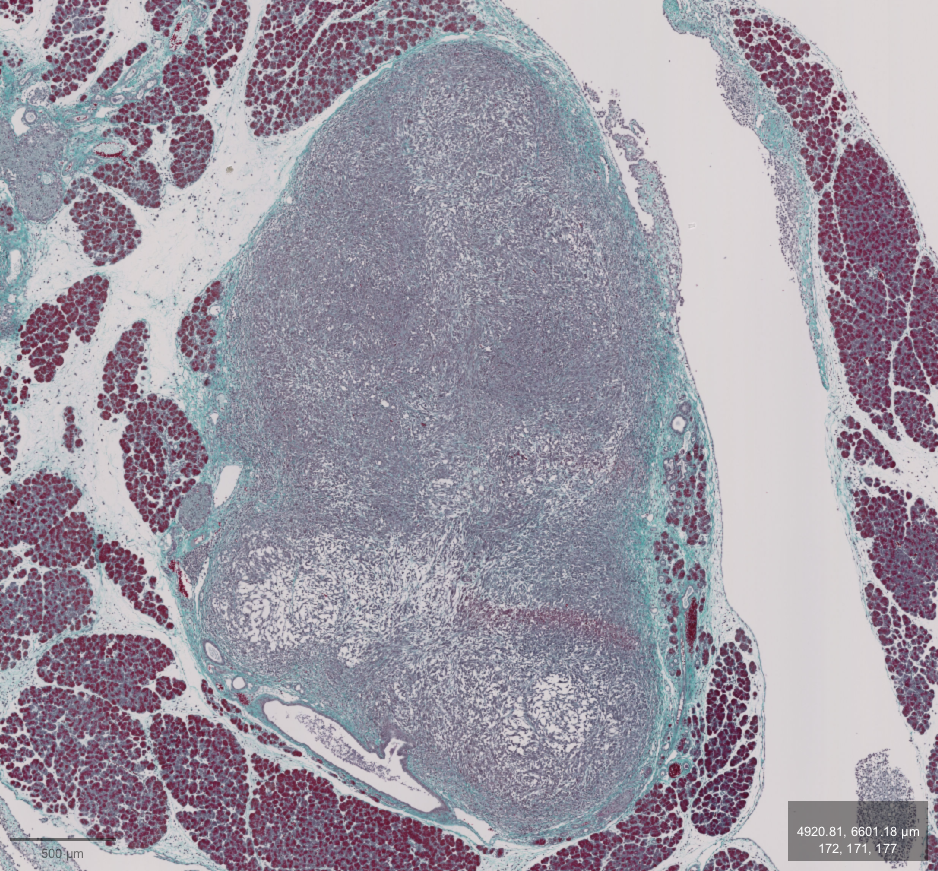

Supplement: Supplementary file 9 — Source data Fig. 7 [file 44318_2025_570_MOESM9_ESM.zip › Fig7/Images/H/Fig7_panel_h_image_5.tif]

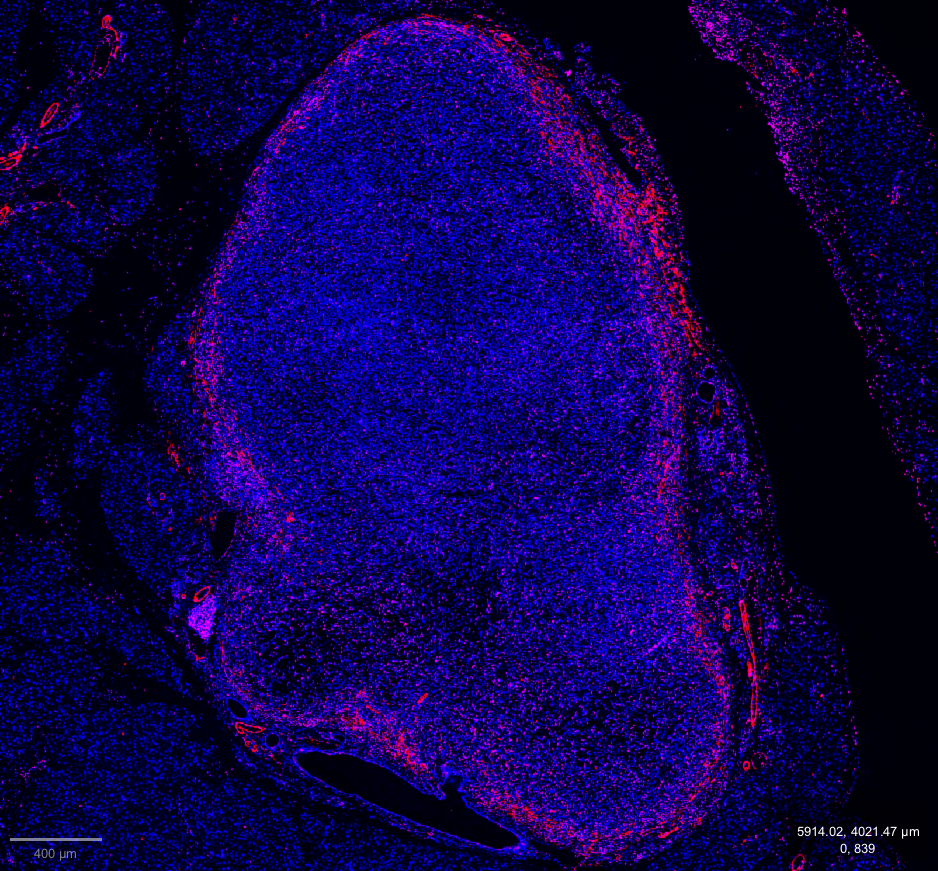

Supplement: Supplementary file 9 — Source data Fig. 7 [file 44318_2025_570_MOESM9_ESM.zip › Fig7/Images/H/Fig7_panel_h_image_6.tif]

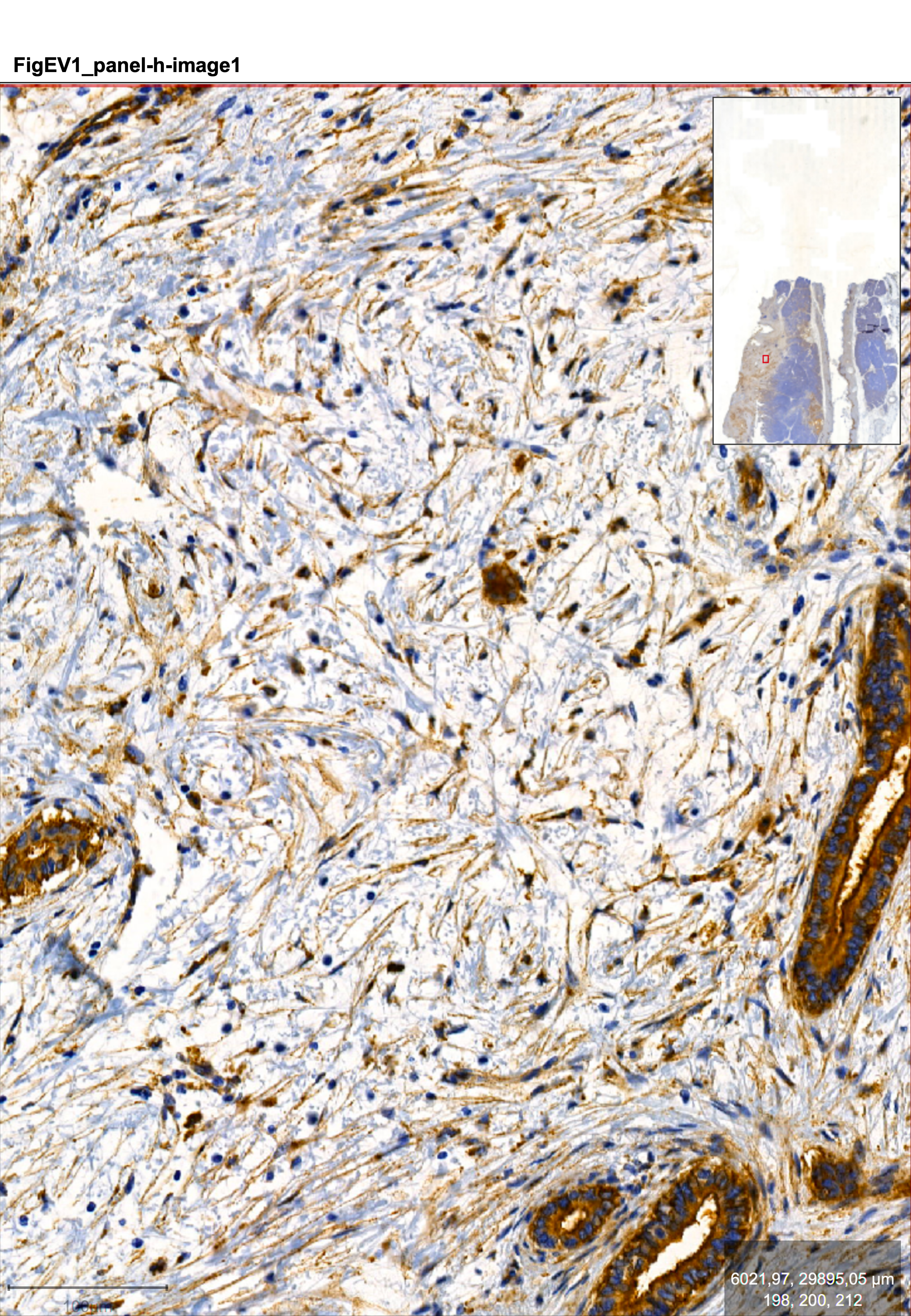

Supplement: Supplementary file 10 — Figure EV1 Source Data [file 44318_2025_570_MOESM10_ESM.zip › FigEV1/Images/H/Fig_EV1_panel_h_image_1.tiff]

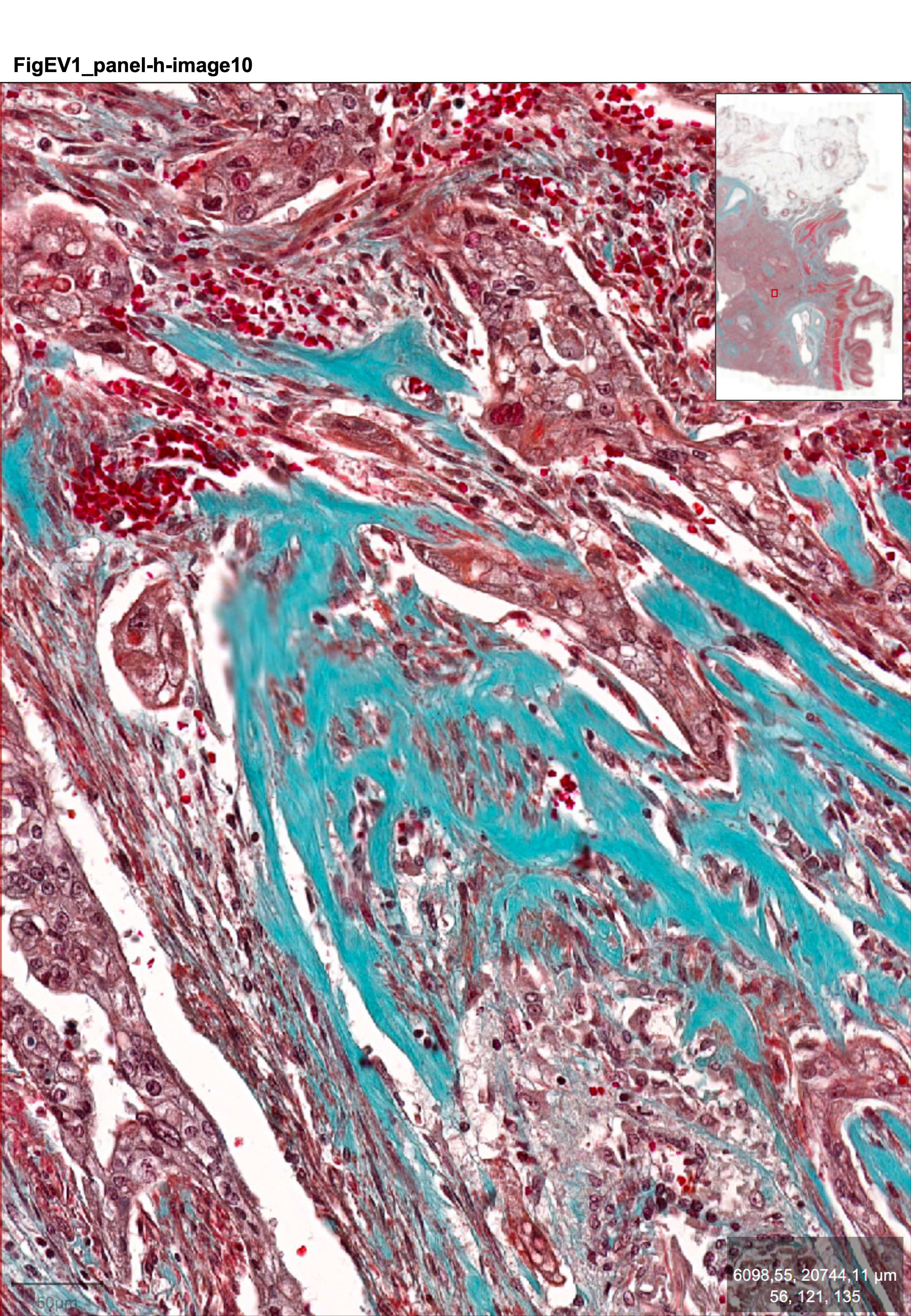

Supplement: Supplementary file 10 — Figure EV1 Source Data [file 44318_2025_570_MOESM10_ESM.zip › FigEV1/Images/H/Fig_EV1_panel_h_image_10.tiff]

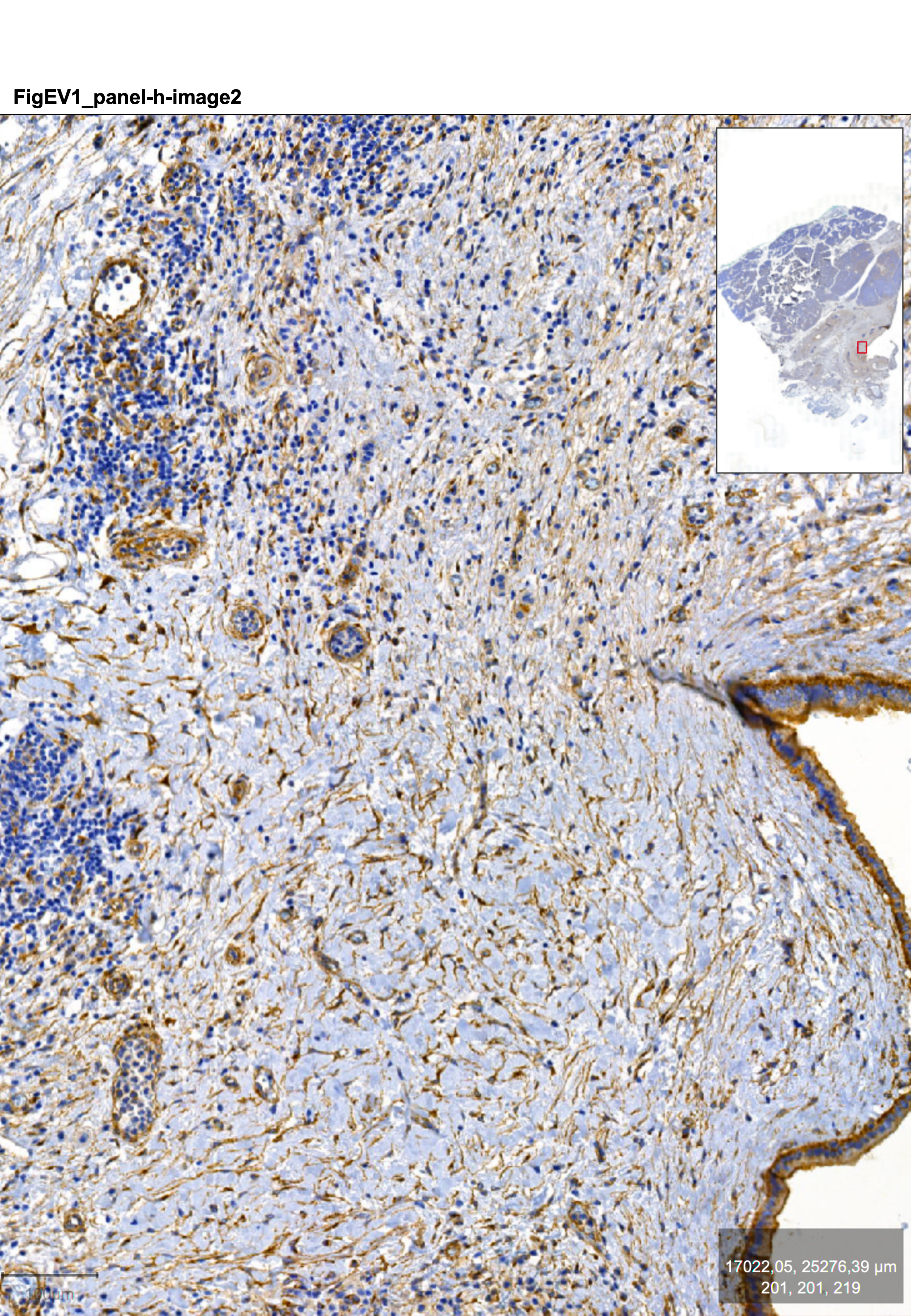

Supplement: Supplementary file 10 — Figure EV1 Source Data [file 44318_2025_570_MOESM10_ESM.zip › FigEV1/Images/H/Fig_EV1_panel_h_image_2.tiff]

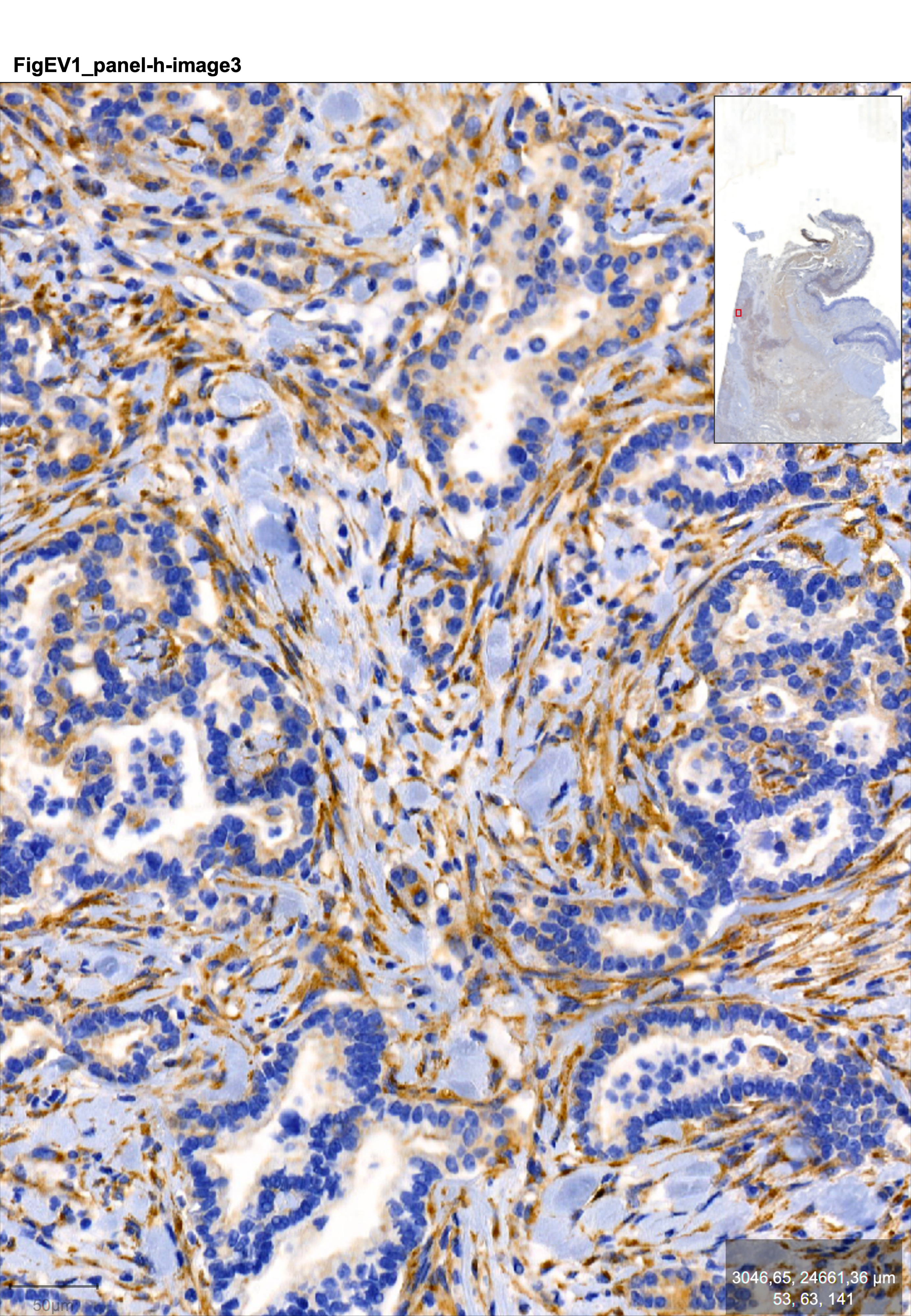

Supplement: Supplementary file 10 — Figure EV1 Source Data [file 44318_2025_570_MOESM10_ESM.zip › FigEV1/Images/H/Fig_EV1_panel_h_image_3.tiff]

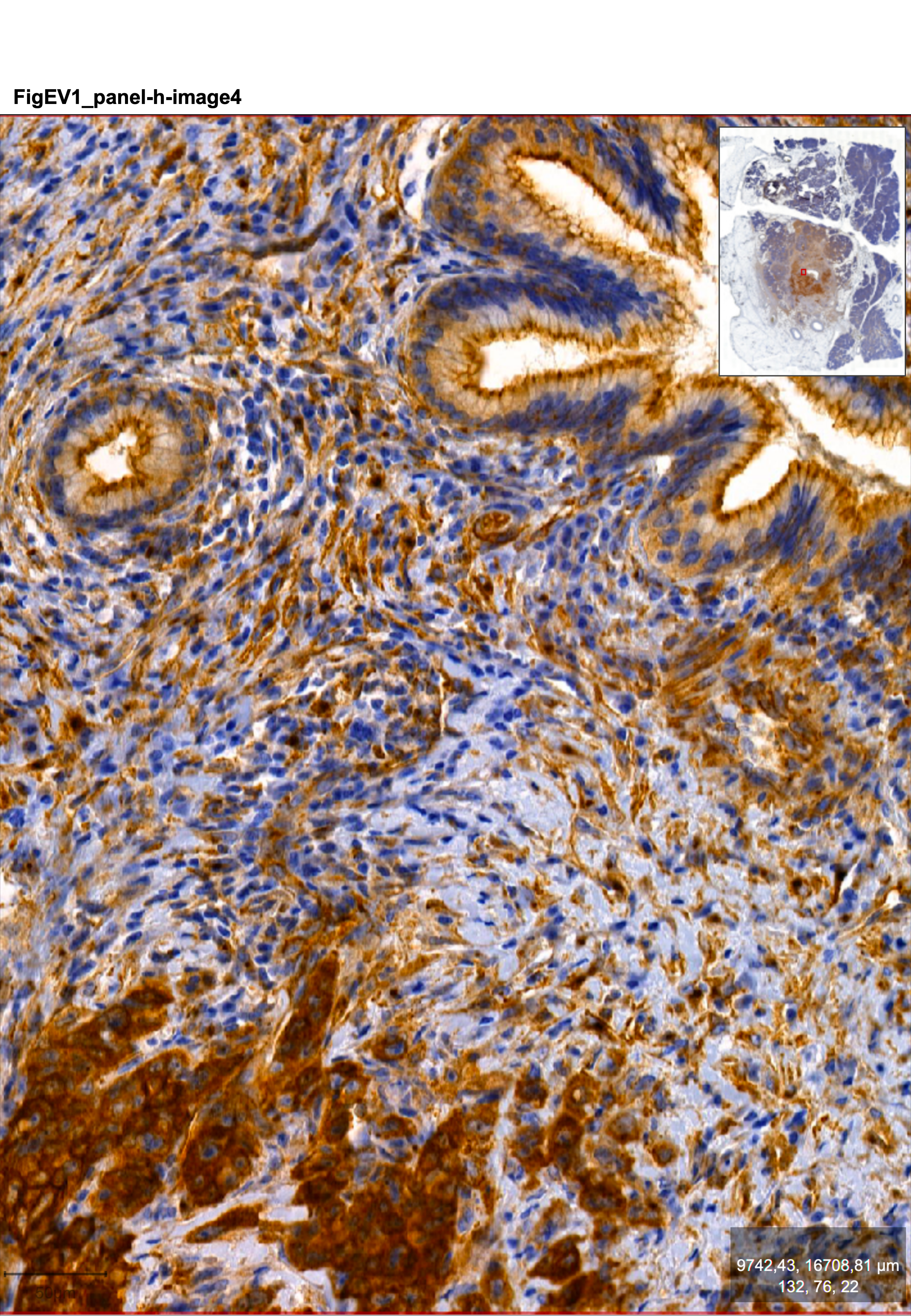

Supplement: Supplementary file 10 — Figure EV1 Source Data [file 44318_2025_570_MOESM10_ESM.zip › FigEV1/Images/H/Fig_EV1_panel_h_image_4.tiff]

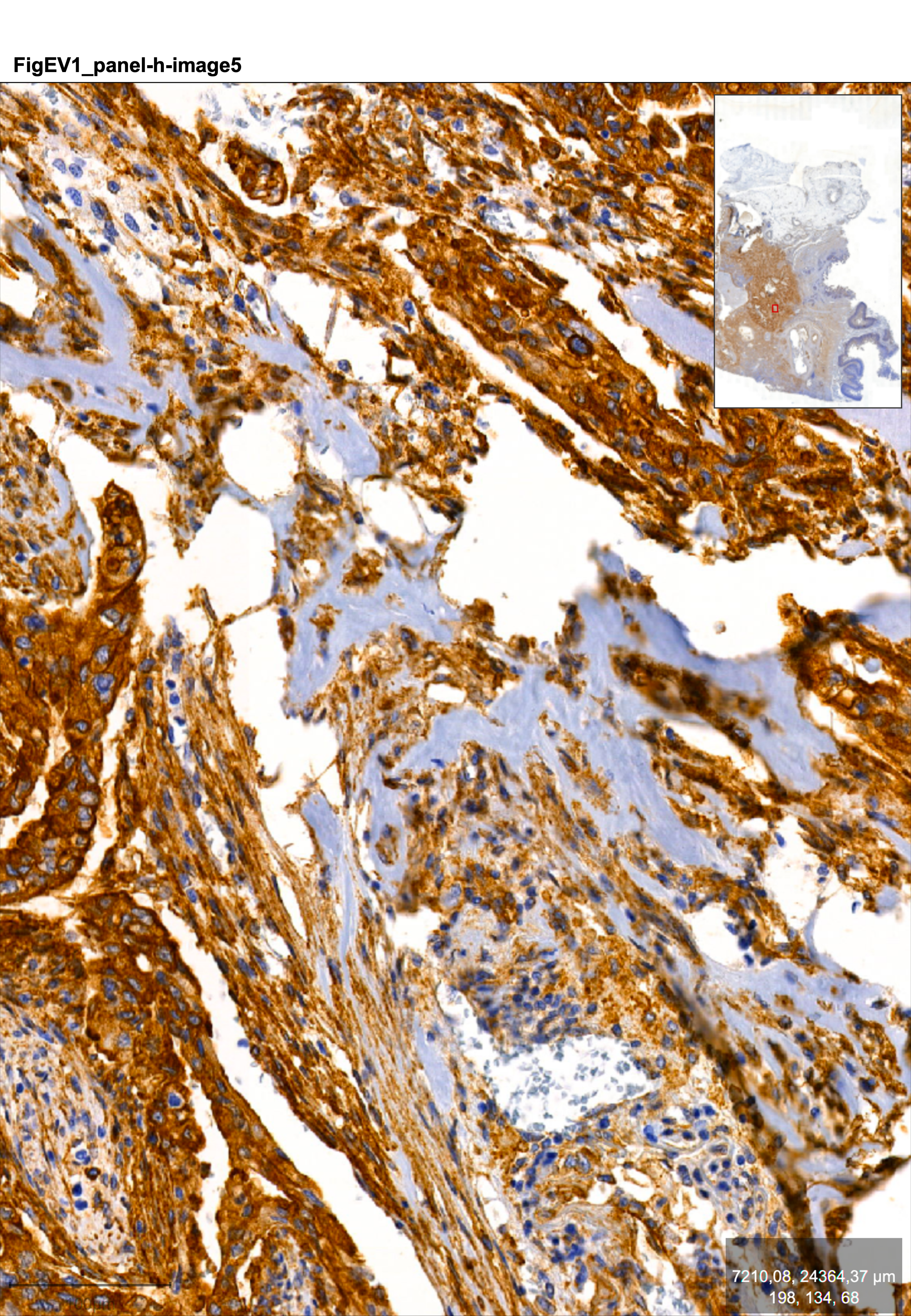

Supplement: Supplementary file 10 — Figure EV1 Source Data [file 44318_2025_570_MOESM10_ESM.zip › FigEV1/Images/H/Fig_EV1_panel_h_image_5.tiff]

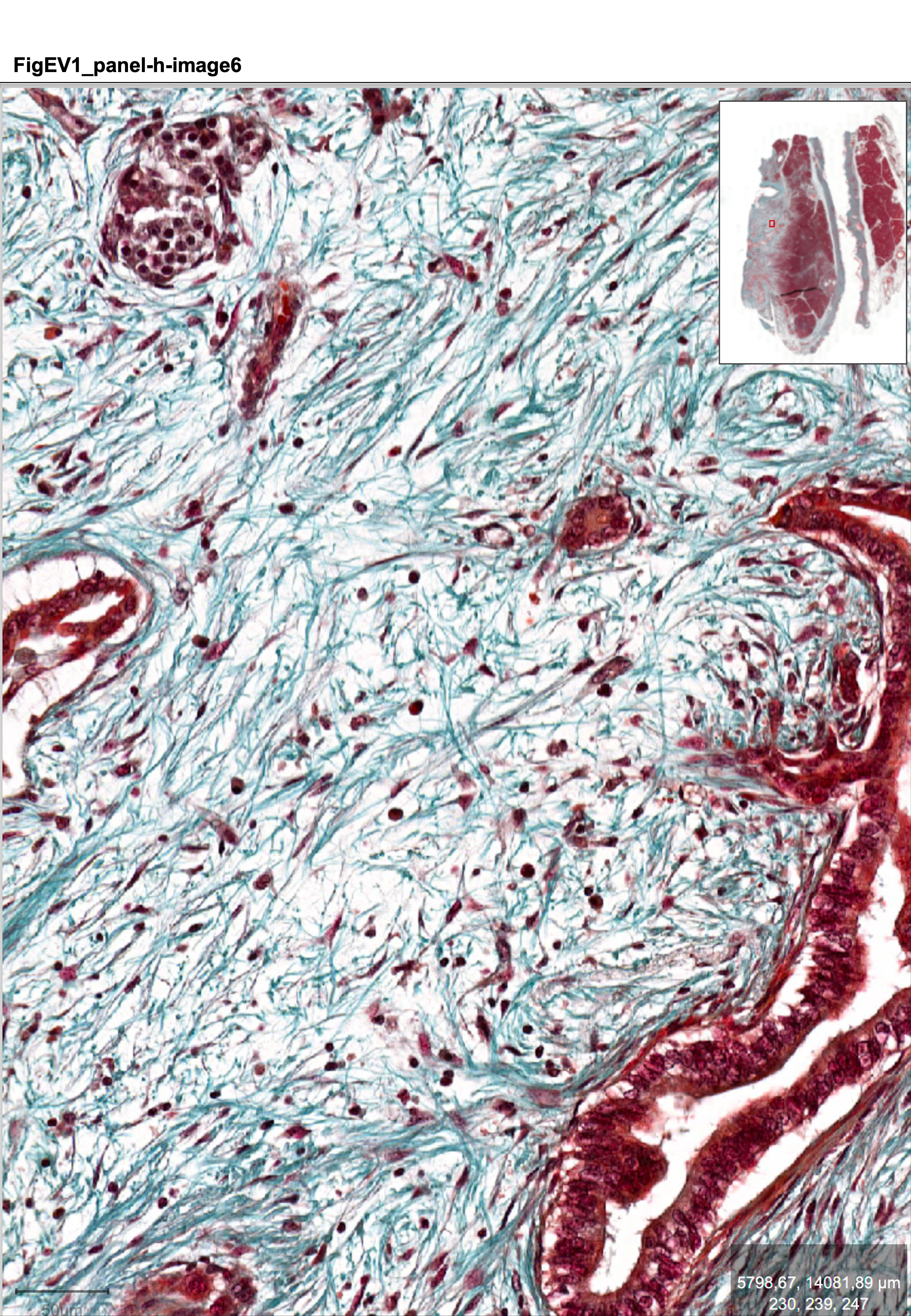

Supplement: Supplementary file 10 — Figure EV1 Source Data [file 44318_2025_570_MOESM10_ESM.zip › FigEV1/Images/H/Fig_EV1_panel_h_image_6.tiff]

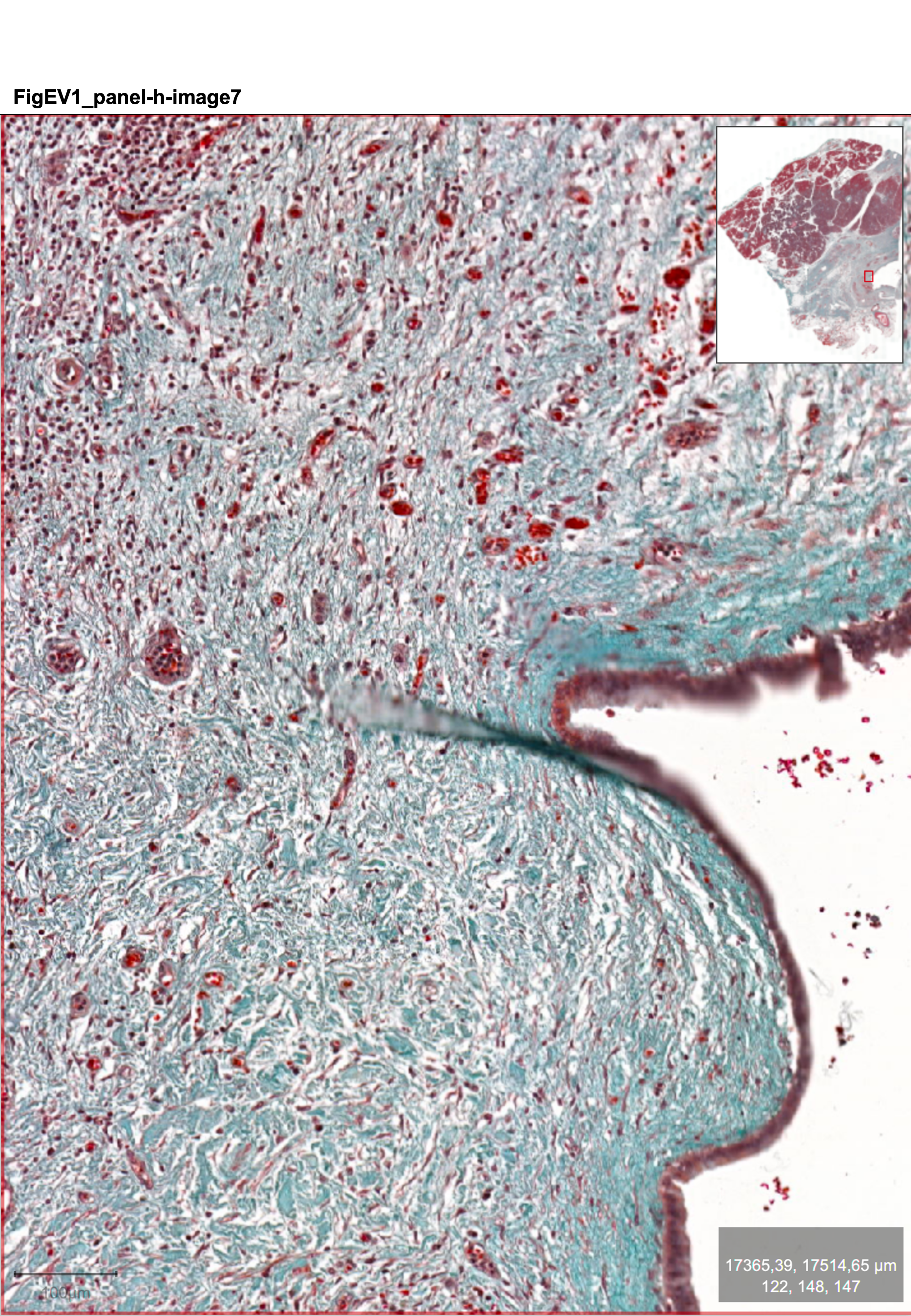

Supplement: Supplementary file 10 — Figure EV1 Source Data [file 44318_2025_570_MOESM10_ESM.zip › FigEV1/Images/H/Fig_EV1_panel_h_image_7.tiff]

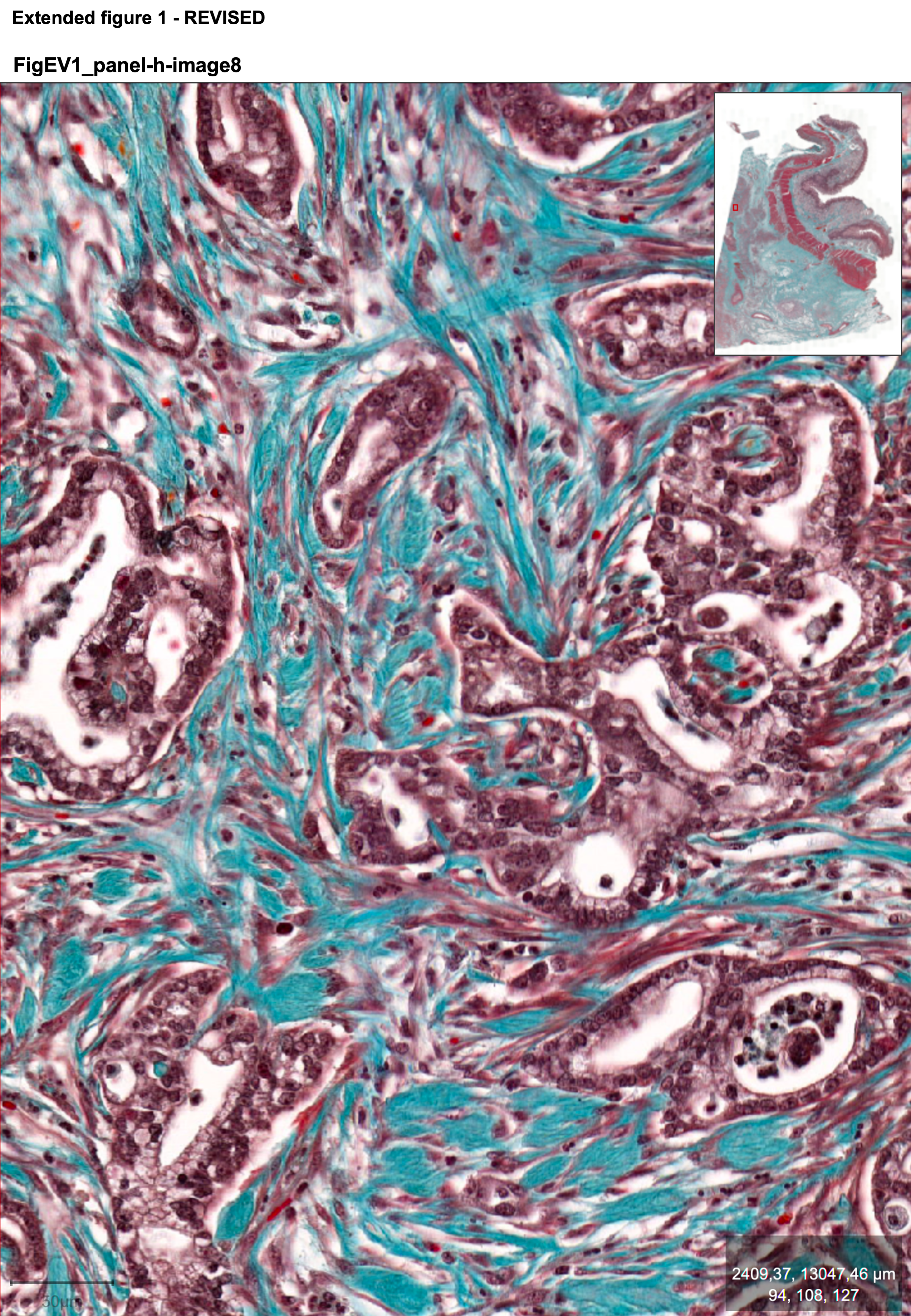

Supplement: Supplementary file 10 — Figure EV1 Source Data [file 44318_2025_570_MOESM10_ESM.zip › FigEV1/Images/H/Fig_EV1_panel_h_image_8.tiff]

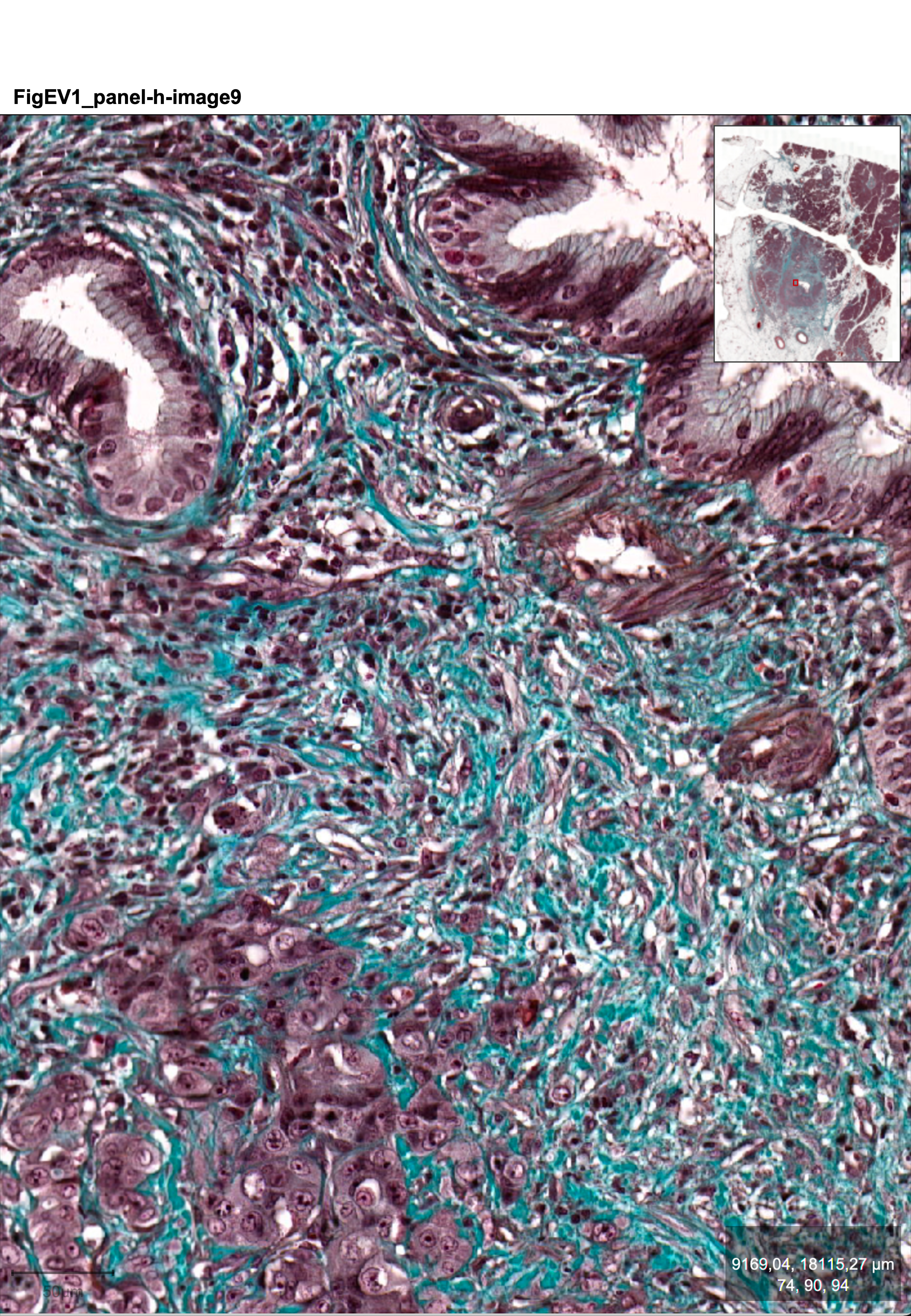

Supplement: Supplementary file 10 — Figure EV1 Source Data [file 44318_2025_570_MOESM10_ESM.zip › FigEV1/Images/H/Fig_EV1_panel_h_image_9.tiff]

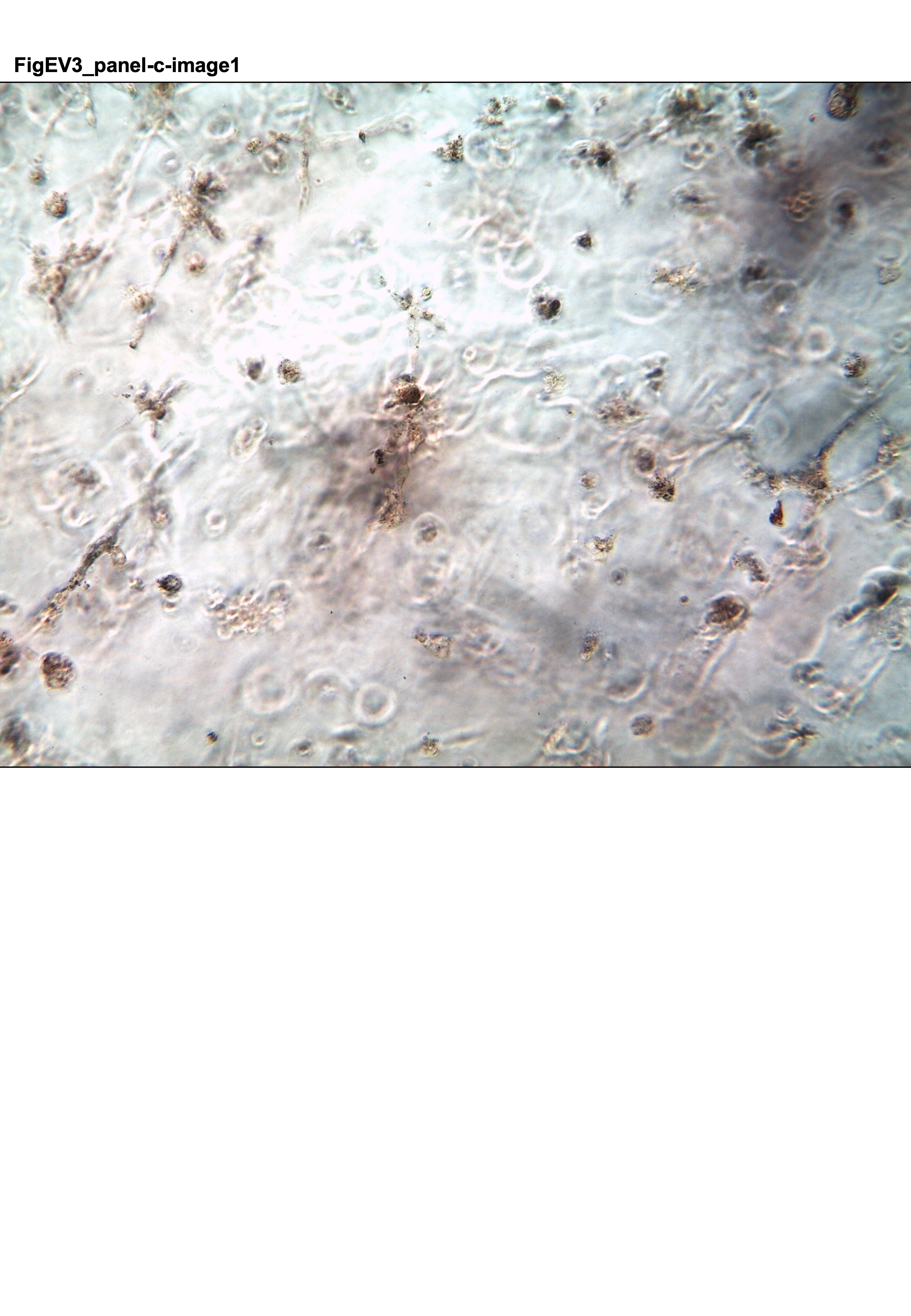

Supplement: Supplementary file 12 — Figure EV3 Source Data [file 44318_2025_570_MOESM12_ESM.zip › FigEV3/Images/C/Fig_EV3_panel_c_image_1.tiff]

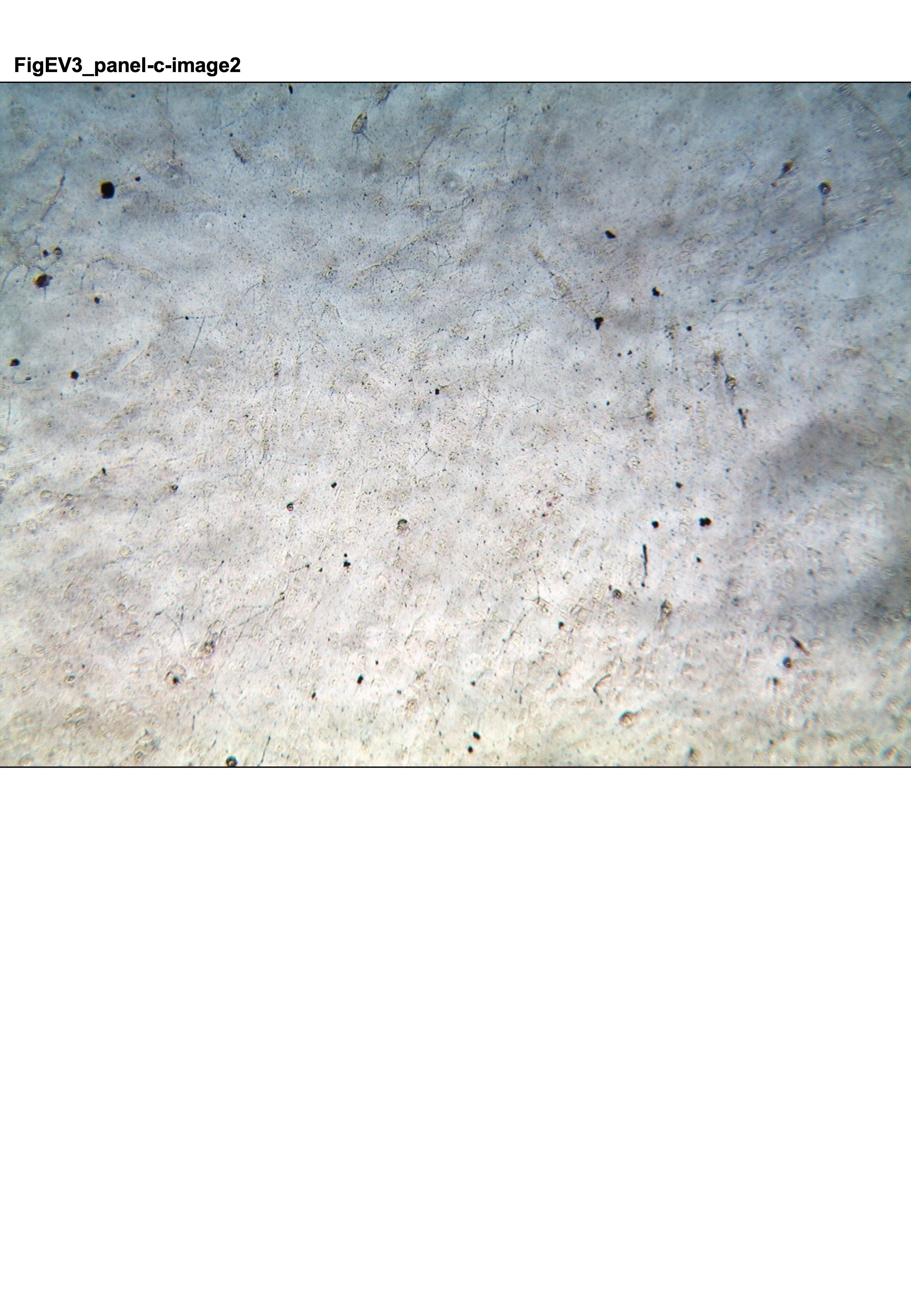

Supplement: Supplementary file 12 — Figure EV3 Source Data [file 44318_2025_570_MOESM12_ESM.zip › FigEV3/Images/C/Fig_EV3_panel_c_image_2.tiff]

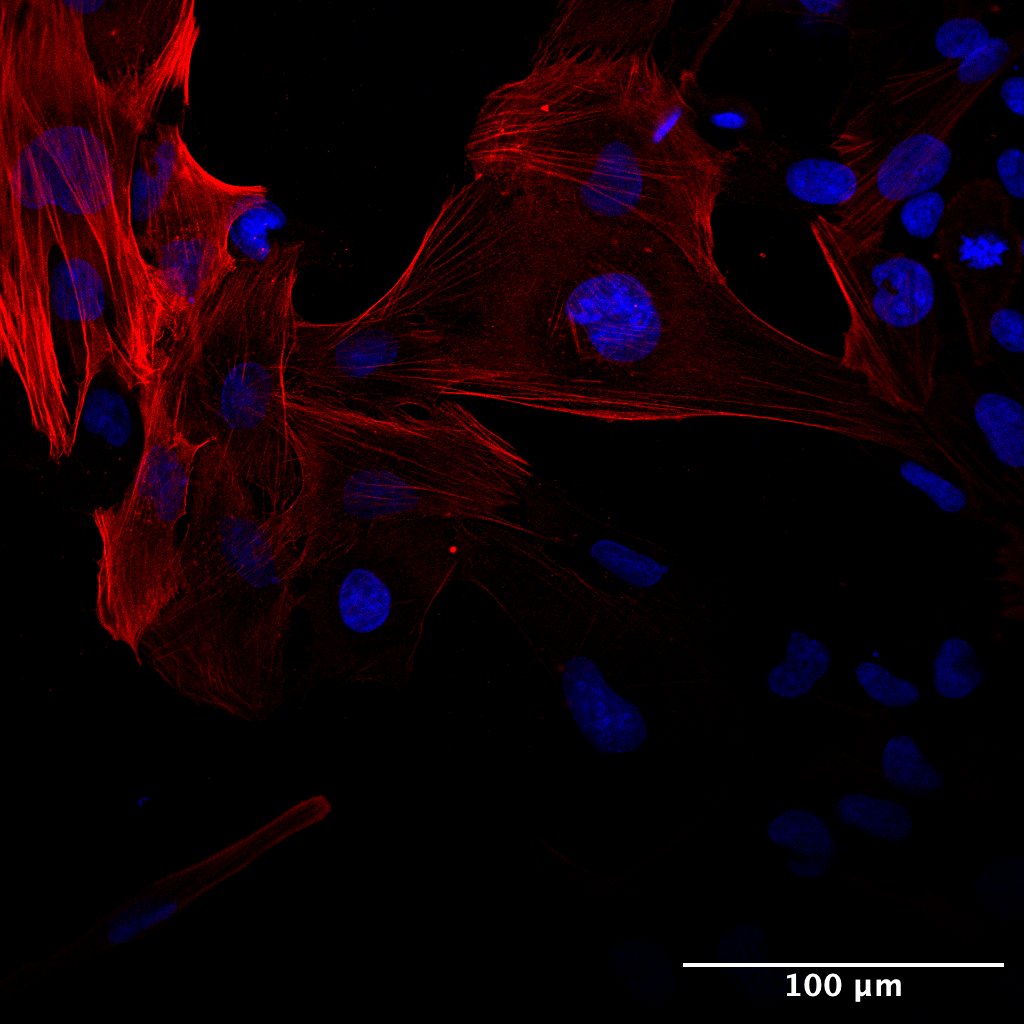

Supplement: Supplementary file 12 — Figure EV3 Source Data [file 44318_2025_570_MOESM12_ESM.zip › FigEV3/Images/H/Fig_EV3_panel_h_HPSC127 sh#1 aSMA_5.jpg]

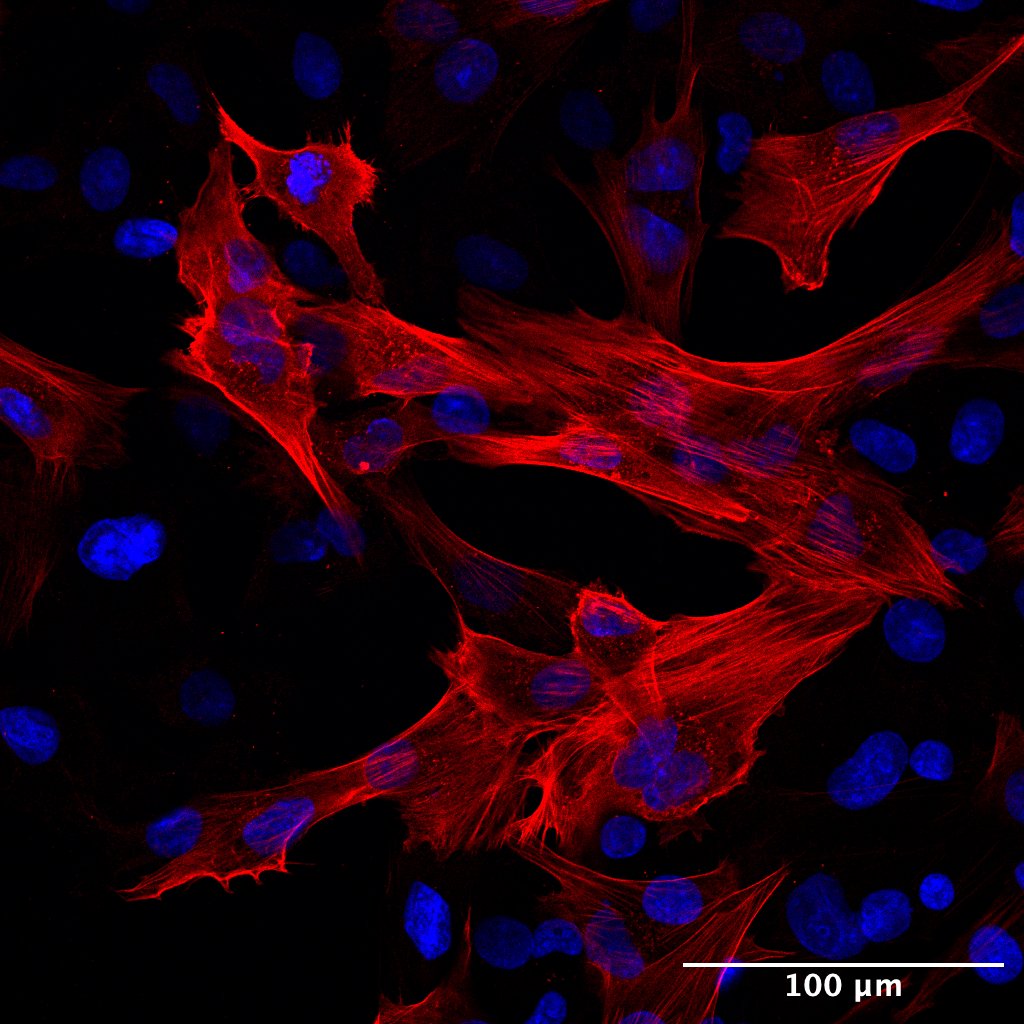

Supplement: Supplementary file 12 — Figure EV3 Source Data [file 44318_2025_570_MOESM12_ESM.zip › FigEV3/Images/H/Fig_EV3_panel_h_HPSC127 sh#5 aSMA_2.jpg]

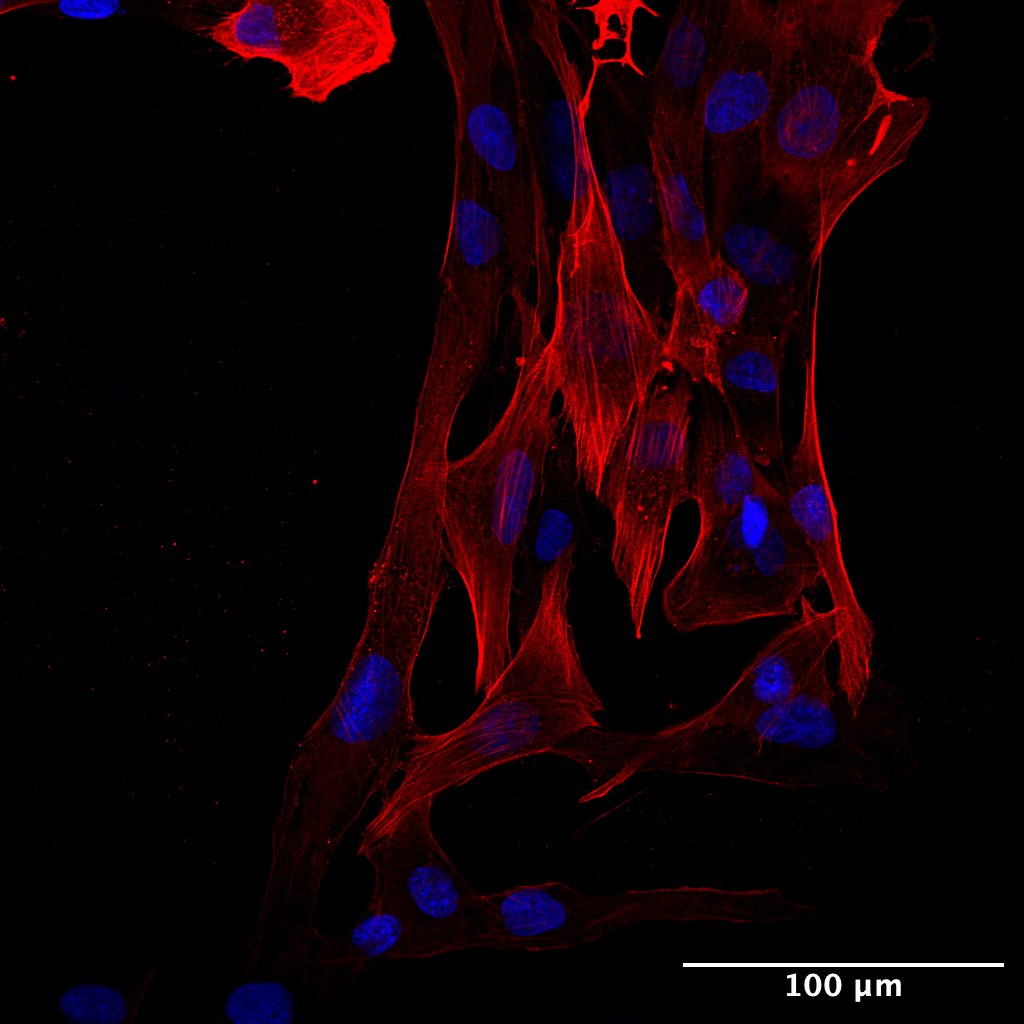

Supplement: Supplementary file 12 — Figure EV3 Source Data [file 44318_2025_570_MOESM12_ESM.zip › FigEV3/Images/H/Fig_EV3_panel_h_HPSC127 shNT aSMA_2.jpg]

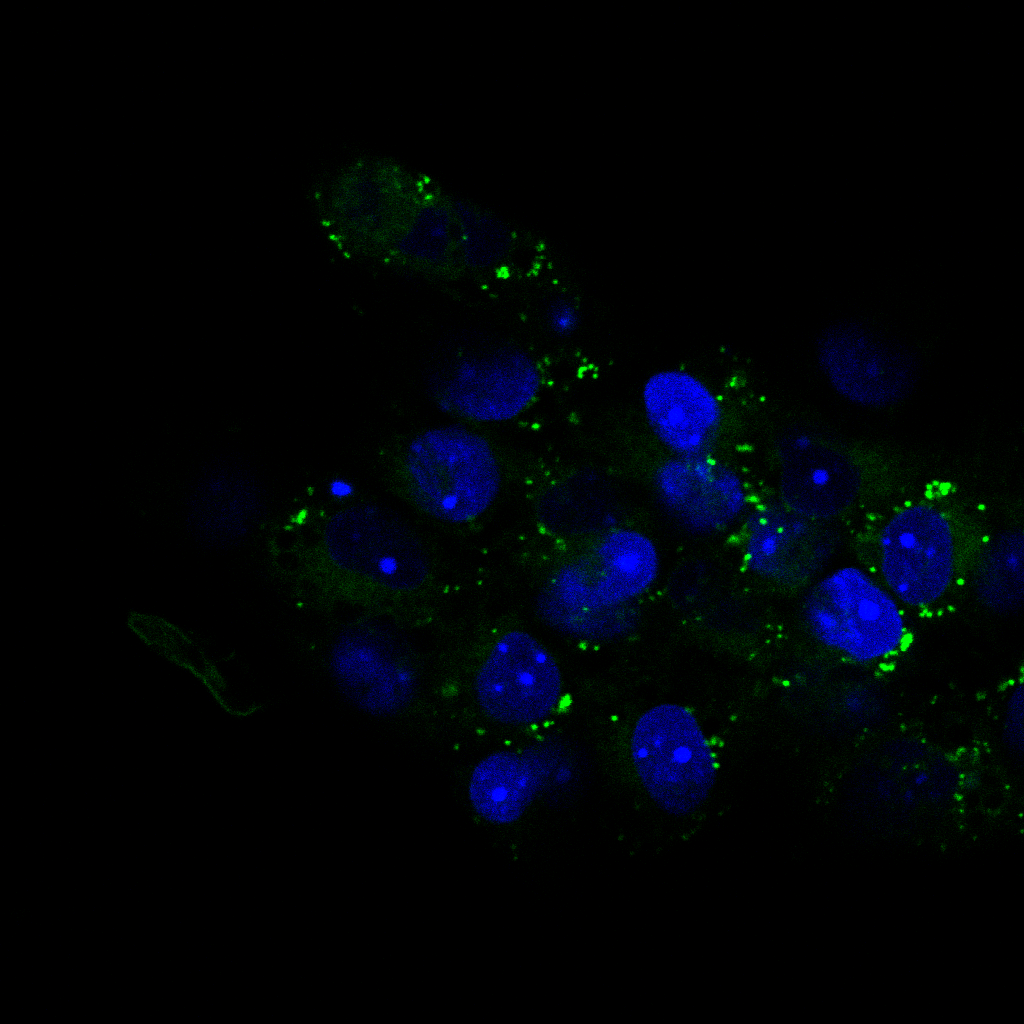

Supplement: Supplementary file 12 — Figure EV3 Source Data [file 44318_2025_570_MOESM12_ESM.zip › FigEV3/Images/K/Fig_EV3_panel_k_image_1.tif]

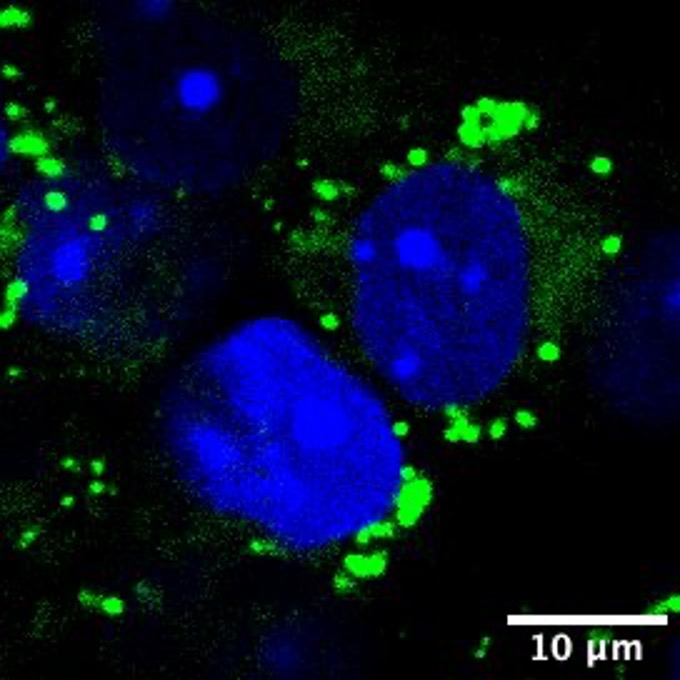

Supplement: Supplementary file 12 — Figure EV3 Source Data [file 44318_2025_570_MOESM12_ESM.zip › FigEV3/Images/K/Fig_EV3_panel_k_image_1_big.jpg]

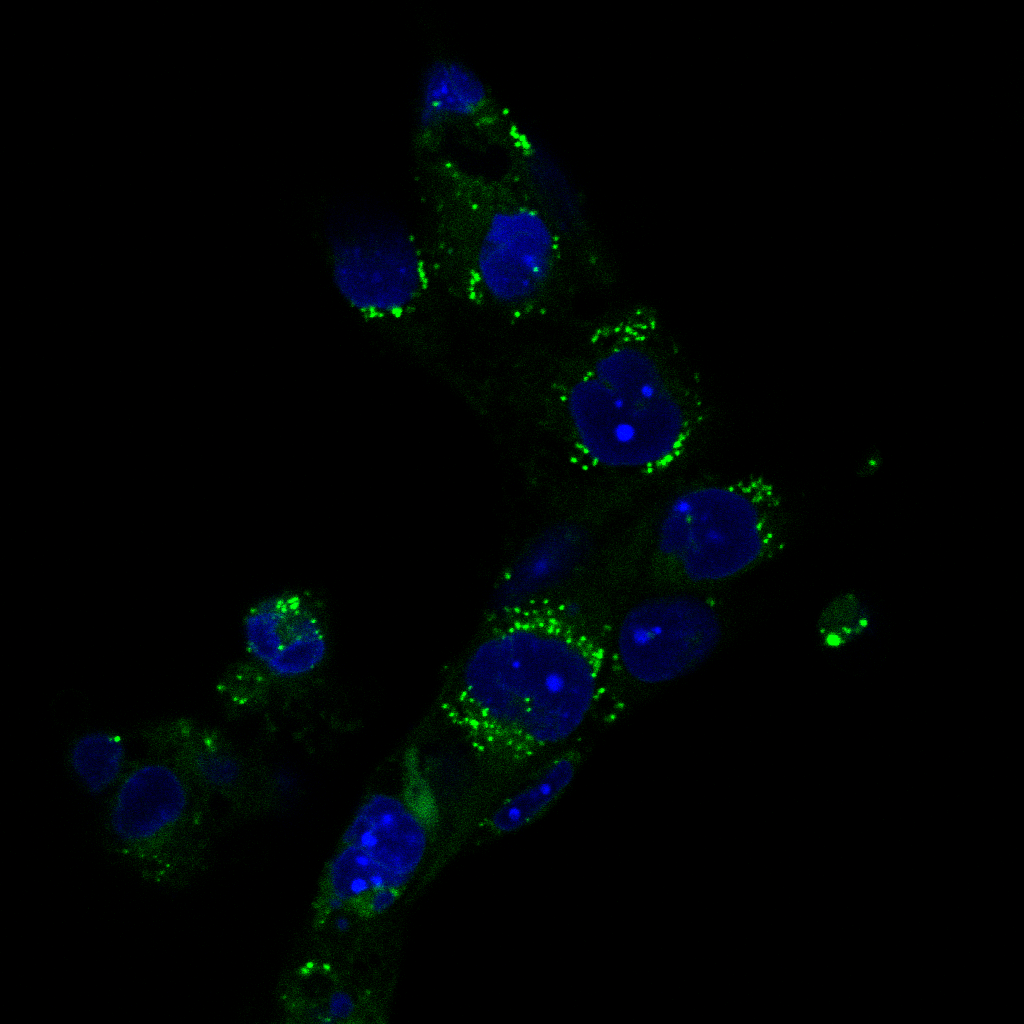

Supplement: Supplementary file 12 — Figure EV3 Source Data [file 44318_2025_570_MOESM12_ESM.zip › FigEV3/Images/K/Fig_EV3_panel_k_image_2.tif]

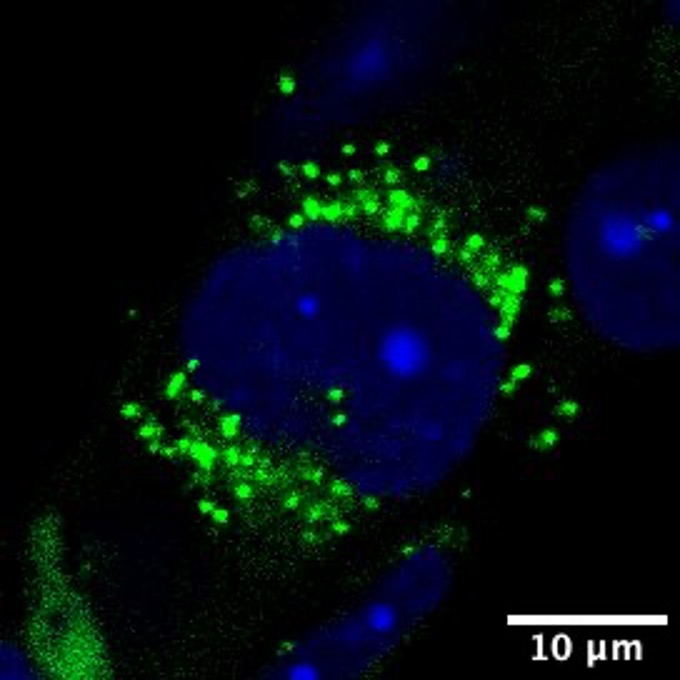

Supplement: Supplementary file 12 — Figure EV3 Source Data [file 44318_2025_570_MOESM12_ESM.zip › FigEV3/Images/K/Fig_EV3_panel_k_image_2_big.jpg]

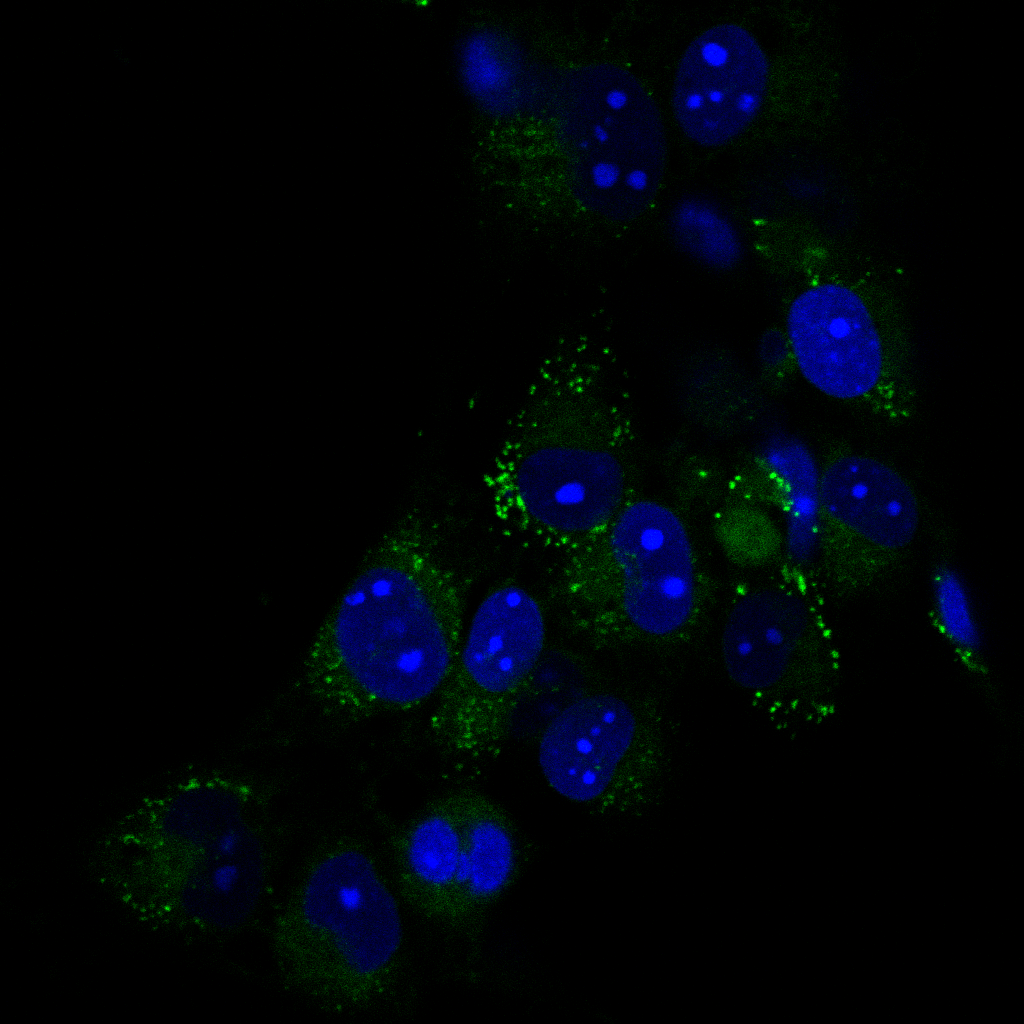

Supplement: Supplementary file 12 — Figure EV3 Source Data [file 44318_2025_570_MOESM12_ESM.zip › FigEV3/Images/K/Fig_EV3_panel_k_image_3.tif]

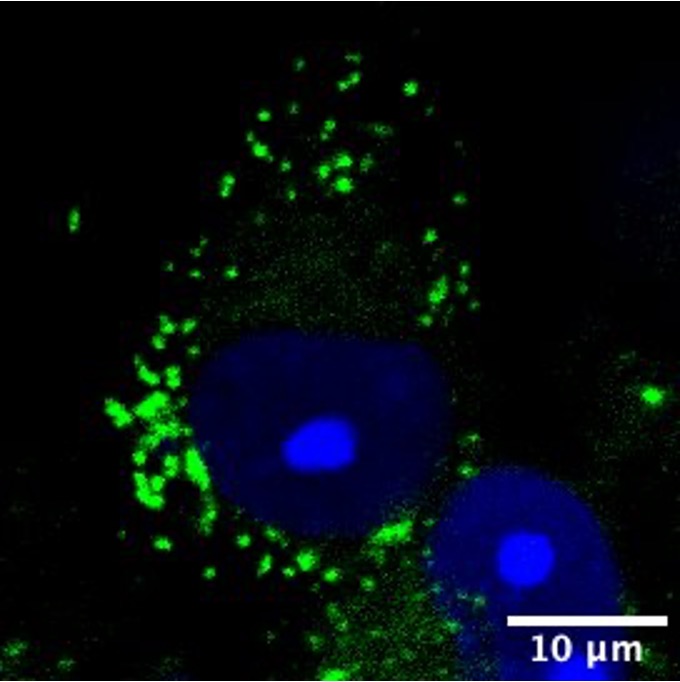

Supplement: Supplementary file 12 — Figure EV3 Source Data [file 44318_2025_570_MOESM12_ESM.zip › FigEV3/Images/K/Fig_EV3_panel_k_image_3_big.jpg]

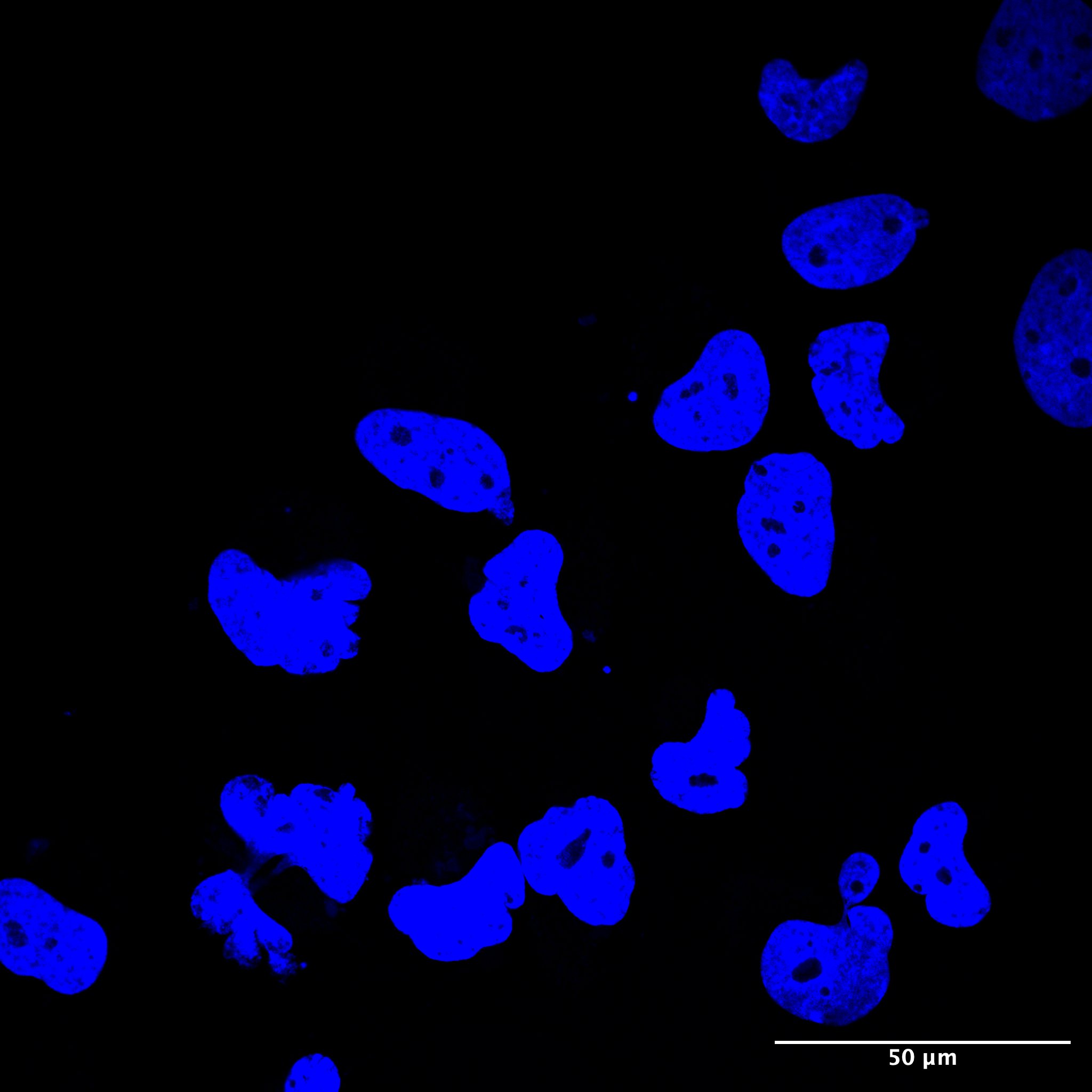

Supplement: Supplementary file 14 — Figure EV5 Source Data [file 44318_2025_570_MOESM14_ESM.zip › FigEV5/Images/D/Fig_EV5_panel_d_SEC24C_MYOF_5_blue.jpg]

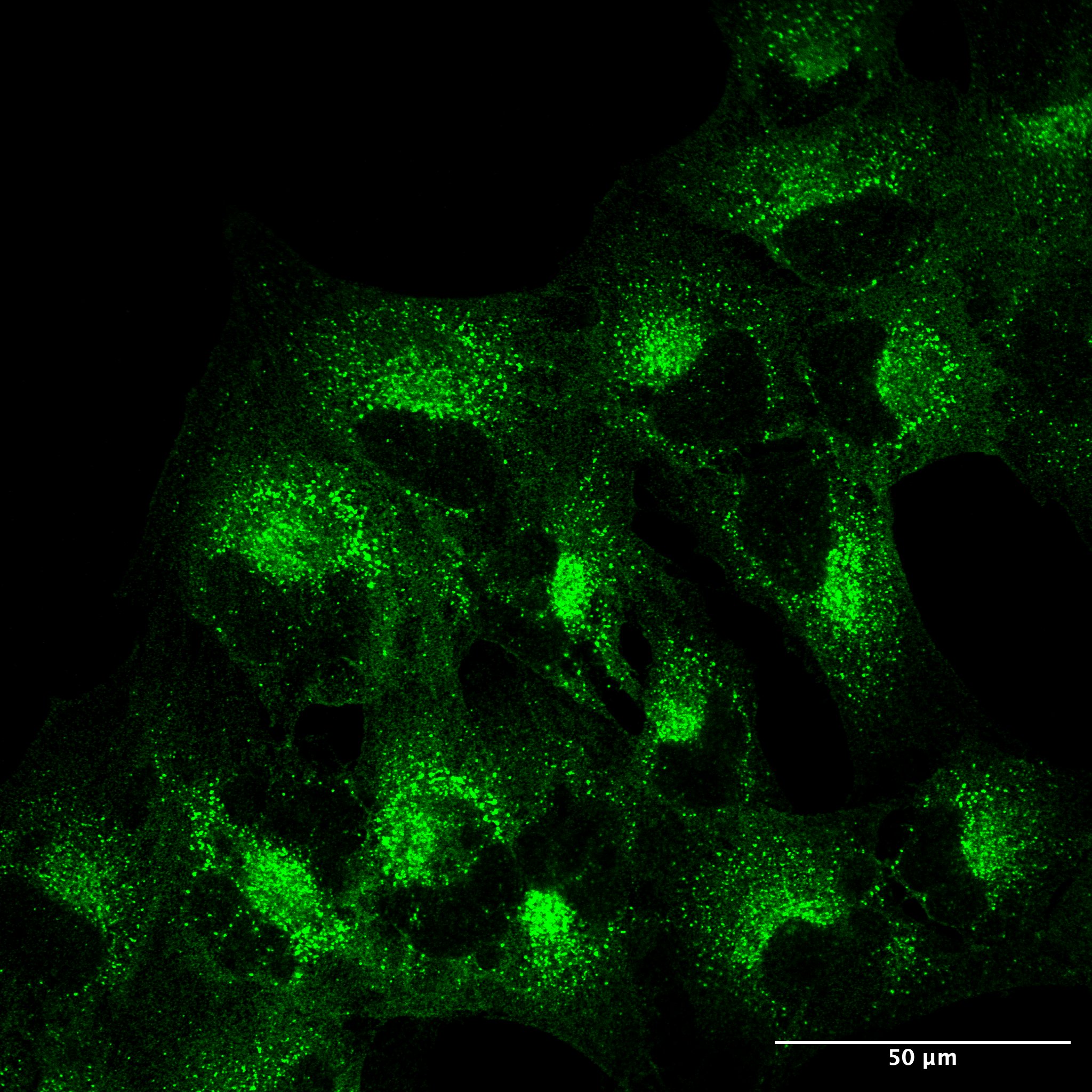

Supplement: Supplementary file 14 — Figure EV5 Source Data [file 44318_2025_570_MOESM14_ESM.zip › FigEV5/Images/D/Fig_EV5_panel_d_SEC24C_MYOF_5_green.jpg]

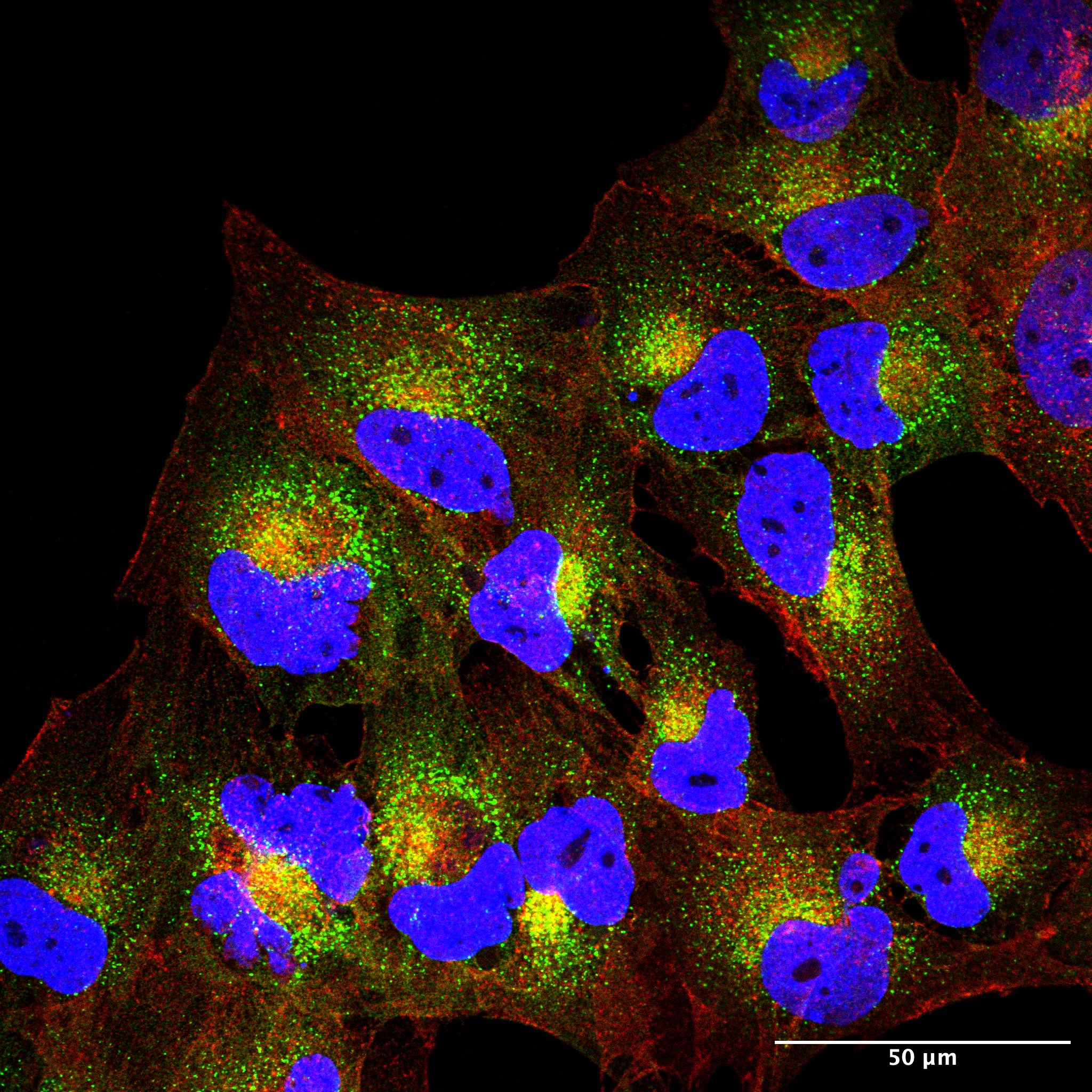

Supplement: Supplementary file 14 — Figure EV5 Source Data [file 44318_2025_570_MOESM14_ESM.zip › FigEV5/Images/D/Fig_EV5_panel_d_SEC24C_MYOF_5_merge.jpg]

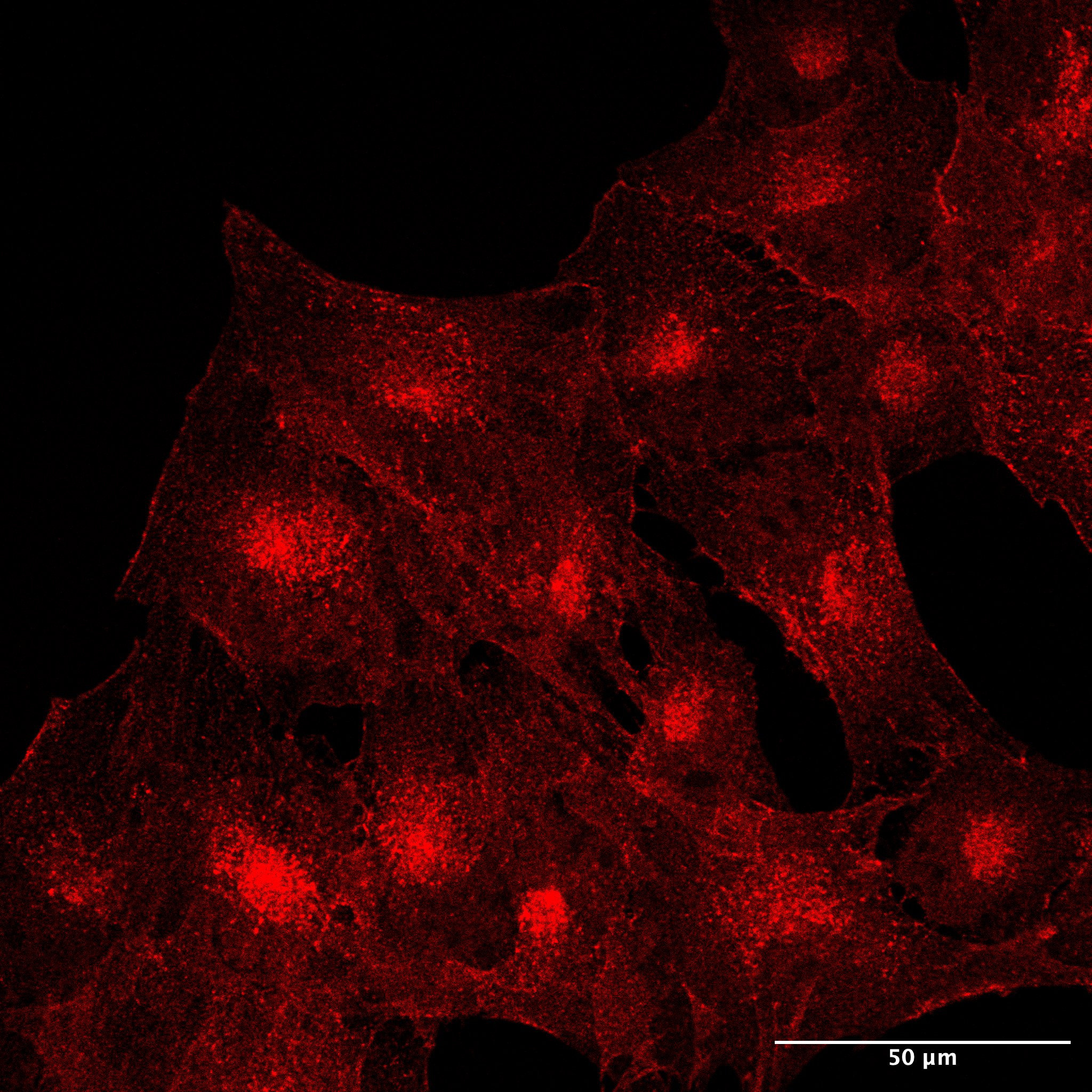

Supplement: Supplementary file 14 — Figure EV5 Source Data [file 44318_2025_570_MOESM14_ESM.zip › FigEV5/Images/D/Fig_EV5_panel_d_SEC24C_MYOF_5_red.jpg]

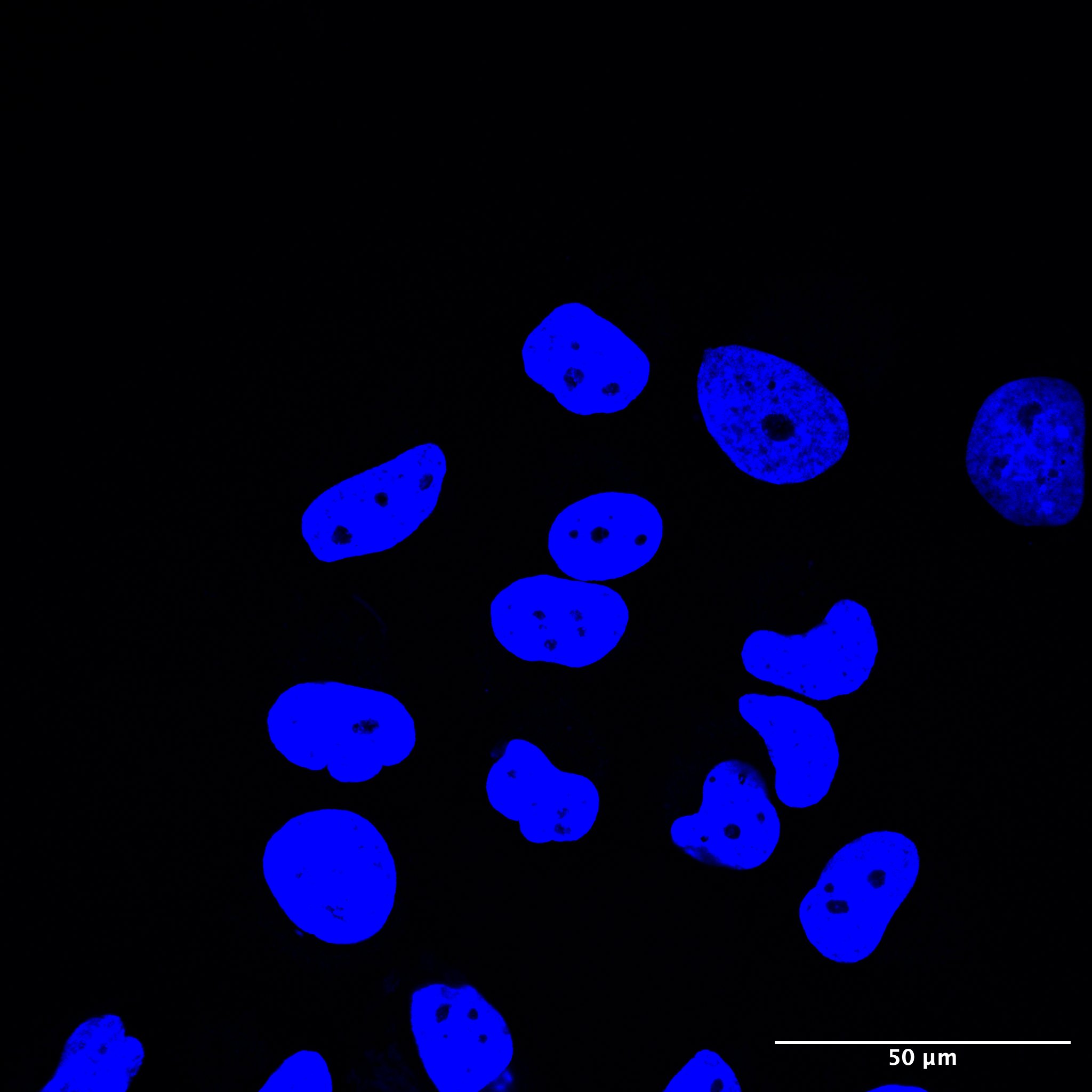

Supplement: Supplementary file 14 — Figure EV5 Source Data [file 44318_2025_570_MOESM14_ESM.zip › FigEV5/Images/E/Fig_EV5_panel_e_TGFBR1_MYOF_2_blue.jpg]

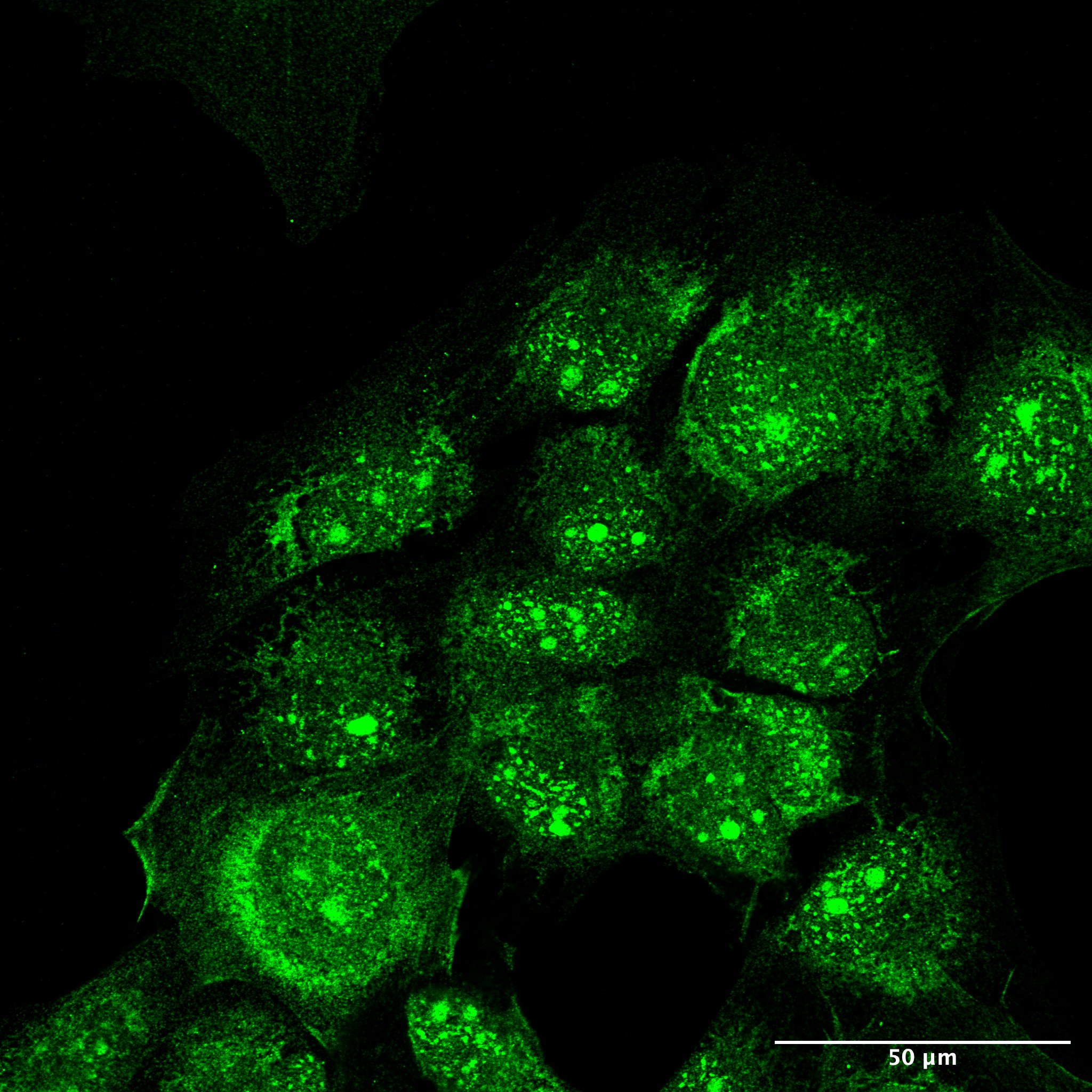

Supplement: Supplementary file 14 — Figure EV5 Source Data [file 44318_2025_570_MOESM14_ESM.zip › FigEV5/Images/E/Fig_EV5_panel_e_TGFBR1_MYOF_2_green.jpg]

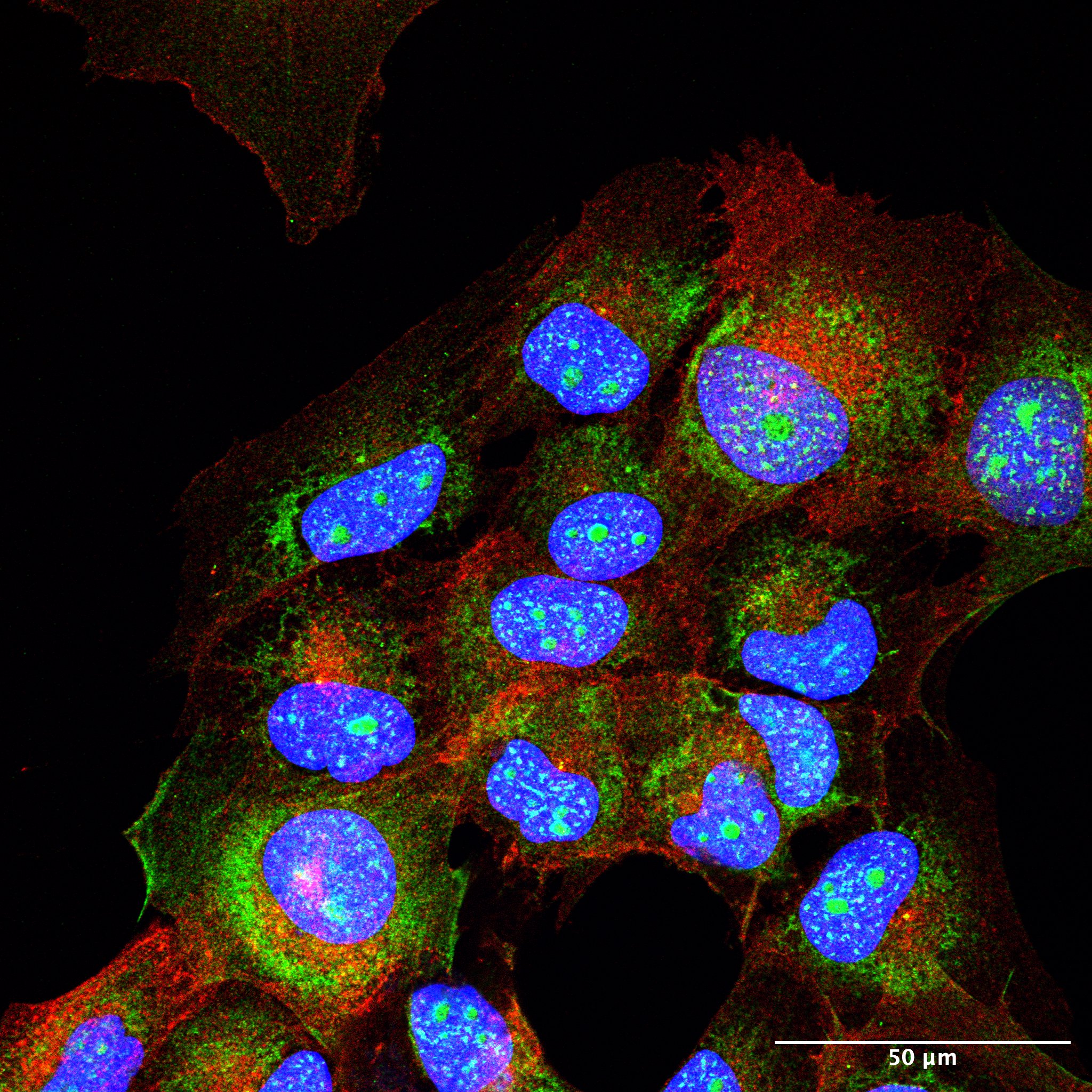

Supplement: Supplementary file 14 — Figure EV5 Source Data [file 44318_2025_570_MOESM14_ESM.zip › FigEV5/Images/E/Fig_EV5_panel_e_TGFBR1_MYOF_2_merge.jpg]

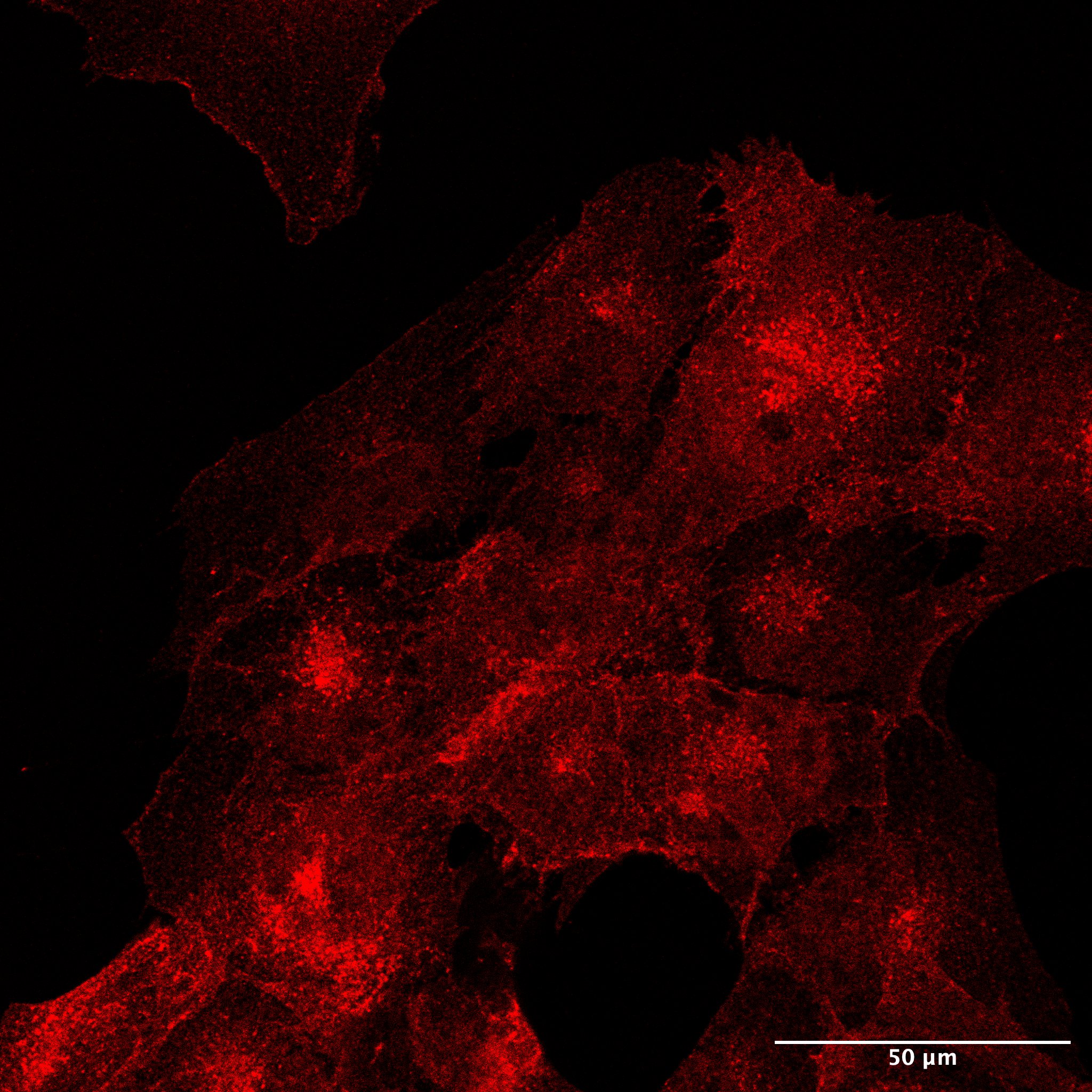

Supplement: Supplementary file 14 — Figure EV5 Source Data [file 44318_2025_570_MOESM14_ESM.zip › FigEV5/Images/E/Fig_EV5_panel_e_TGFBR1_MYOF_2_red.jpg]

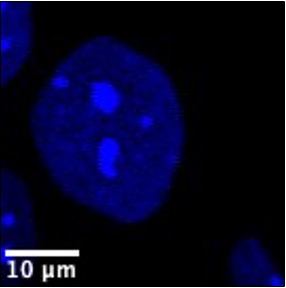

Supplement: Supplementary file 14 — Figure EV5 Source Data [file 44318_2025_570_MOESM14_ESM.zip › FigEV5/Images/F/Fig_EV5_panel_f_EL30_Nucleus_1_Big.jpg]

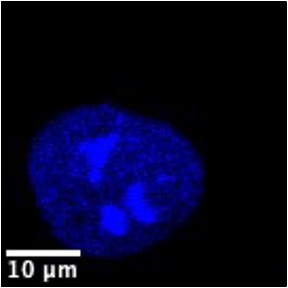

Supplement: Supplementary file 14 — Figure EV5 Source Data [file 44318_2025_570_MOESM14_ESM.zip › FigEV5/Images/F/Fig_EV5_panel_f_EL30_Nucleus_2_Big.jpg]

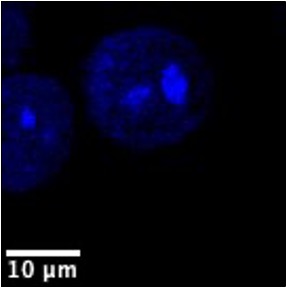

Supplement: Supplementary file 14 — Figure EV5 Source Data [file 44318_2025_570_MOESM14_ESM.zip › FigEV5/Images/F/Fig_EV5_panel_f_EL30_Nucleus_3_Big.jpg]

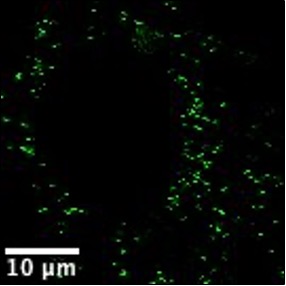

Supplement: Supplementary file 14 — Figure EV5 Source Data [file 44318_2025_570_MOESM14_ESM.zip › FigEV5/Images/F/Fig_EV5_panel_f_EL30_PLA_1_Big.jpg]

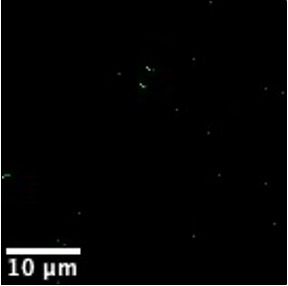

Supplement: Supplementary file 14 — Figure EV5 Source Data [file 44318_2025_570_MOESM14_ESM.zip › FigEV5/Images/F/Fig_EV5_panel_f_EL30_PLA_2_Big.jpg]

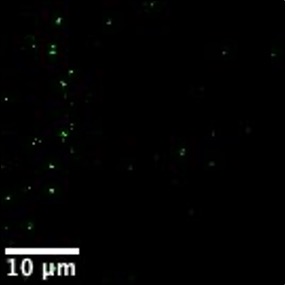

Supplement: Supplementary file 14 — Figure EV5 Source Data [file 44318_2025_570_MOESM14_ESM.zip › FigEV5/Images/F/Fig_EV5_panel_f_EL30_PLA_3_Big.jpg]

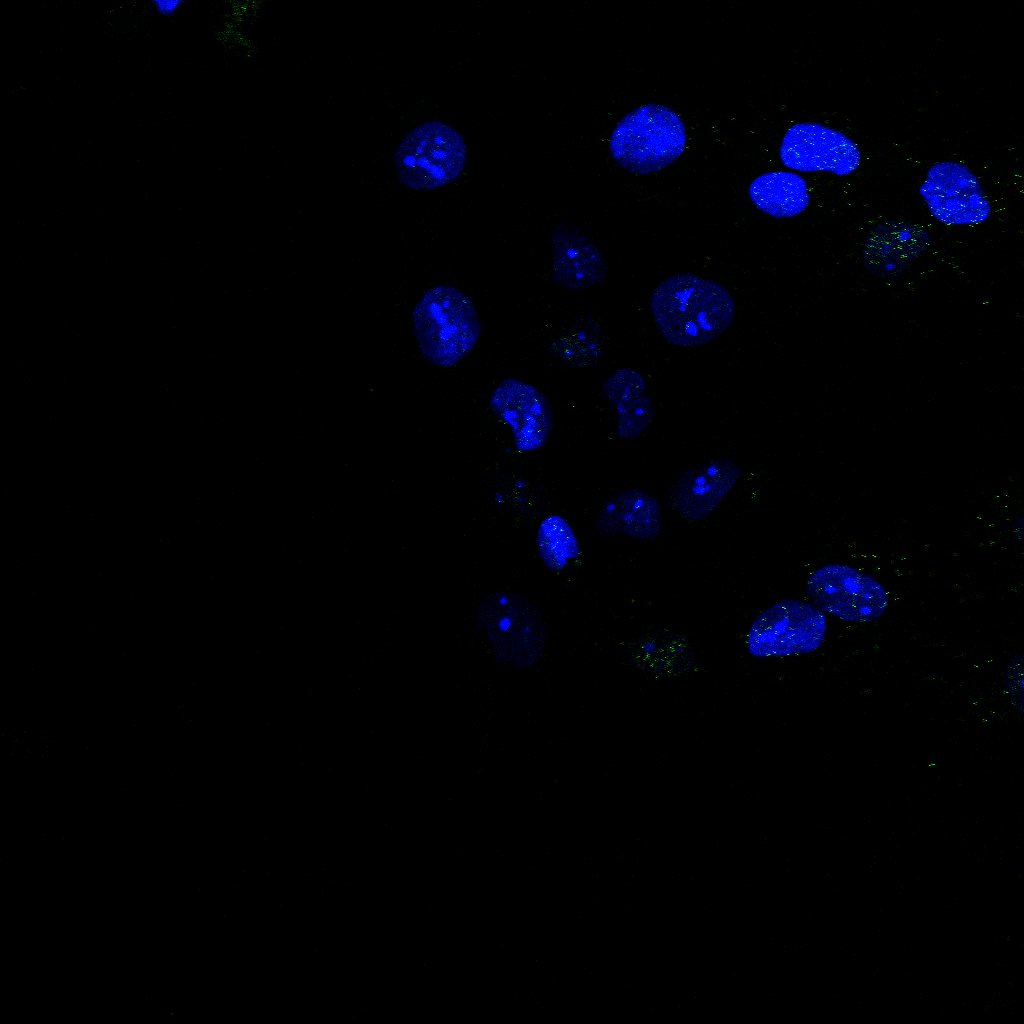

Supplement: Supplementary file 14 — Figure EV5 Source Data [file 44318_2025_570_MOESM14_ESM.zip › FigEV5/Images/F/Fig_EV5_panel_f_EL30_sh#1.jpg]

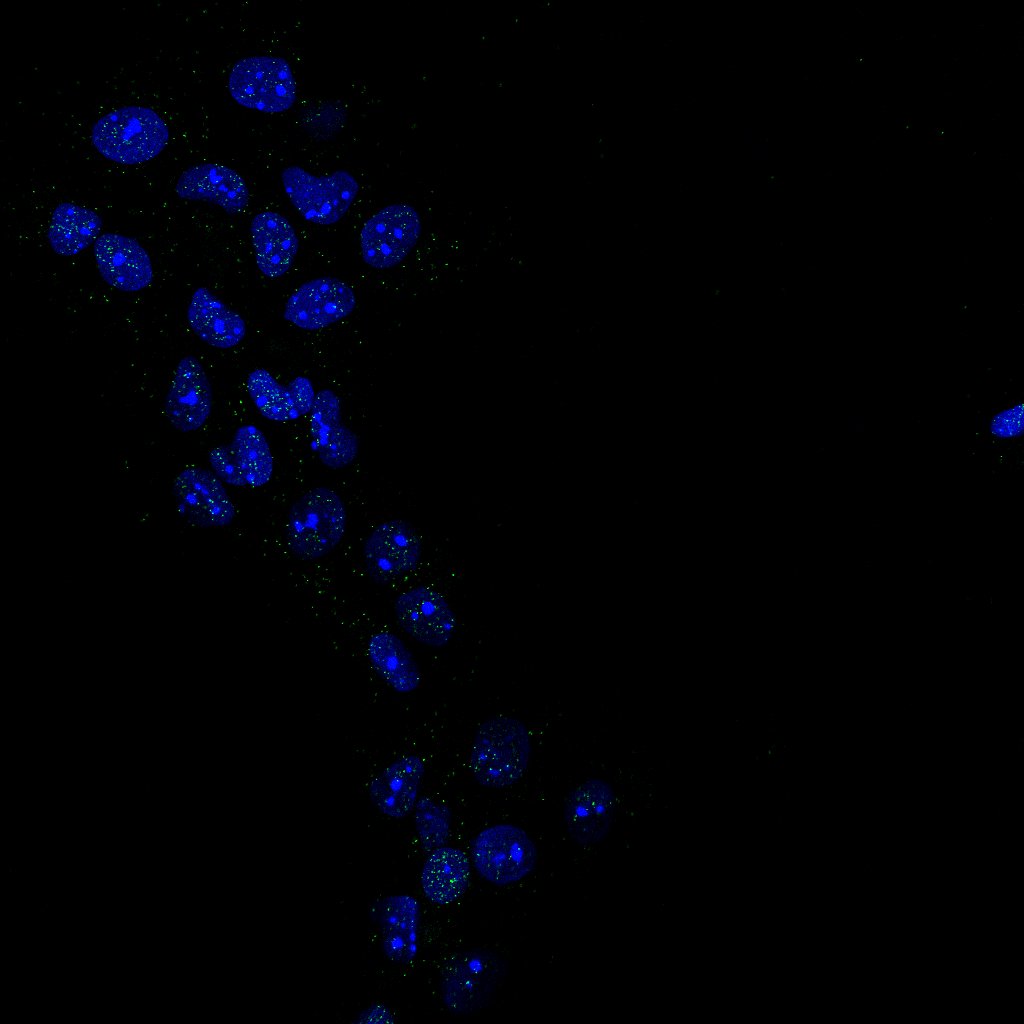

Supplement: Supplementary file 14 — Figure EV5 Source Data [file 44318_2025_570_MOESM14_ESM.zip › FigEV5/Images/F/Fig_EV5_panel_f_EL30_sh#5.jpg]

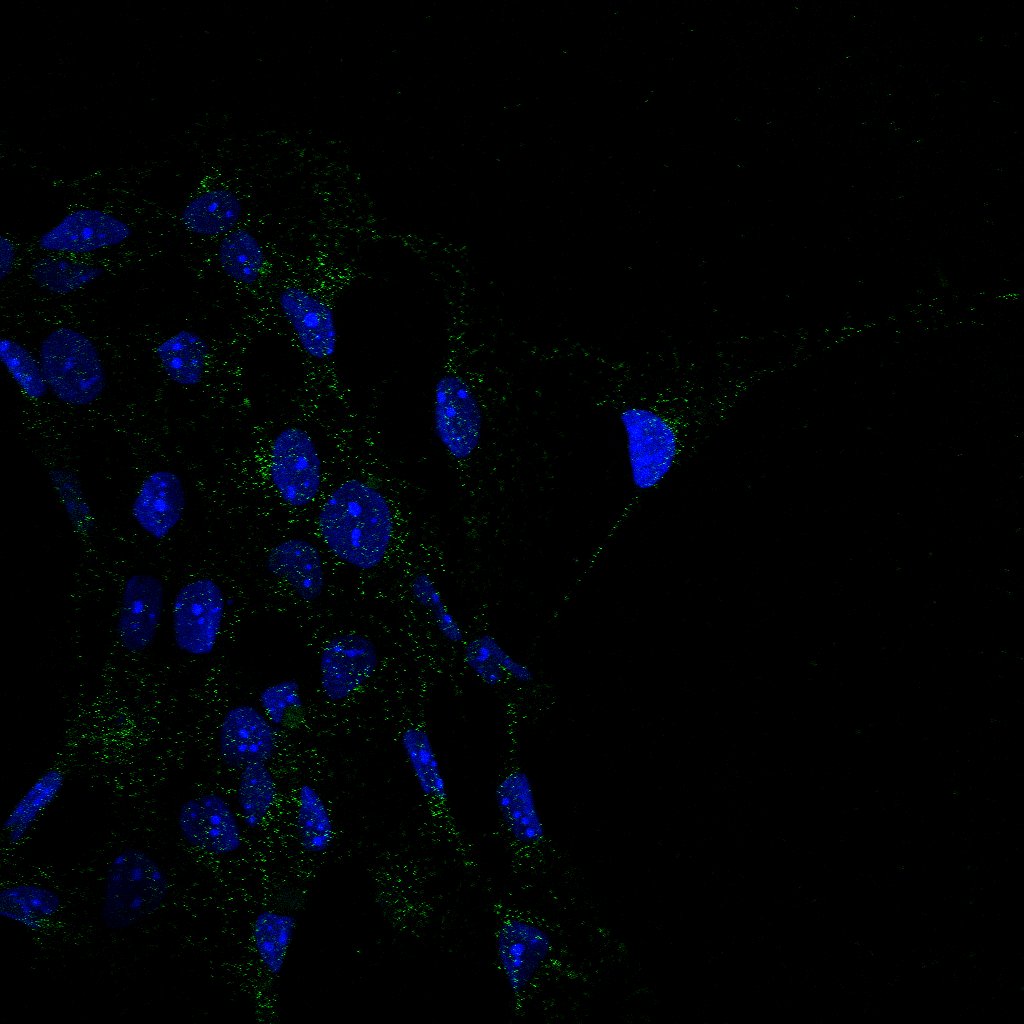

Supplement: Supplementary file 14 — Figure EV5 Source Data [file 44318_2025_570_MOESM14_ESM.zip › FigEV5/Images/F/Fig_EV5_panel_f_EL30_shNT.jpg]

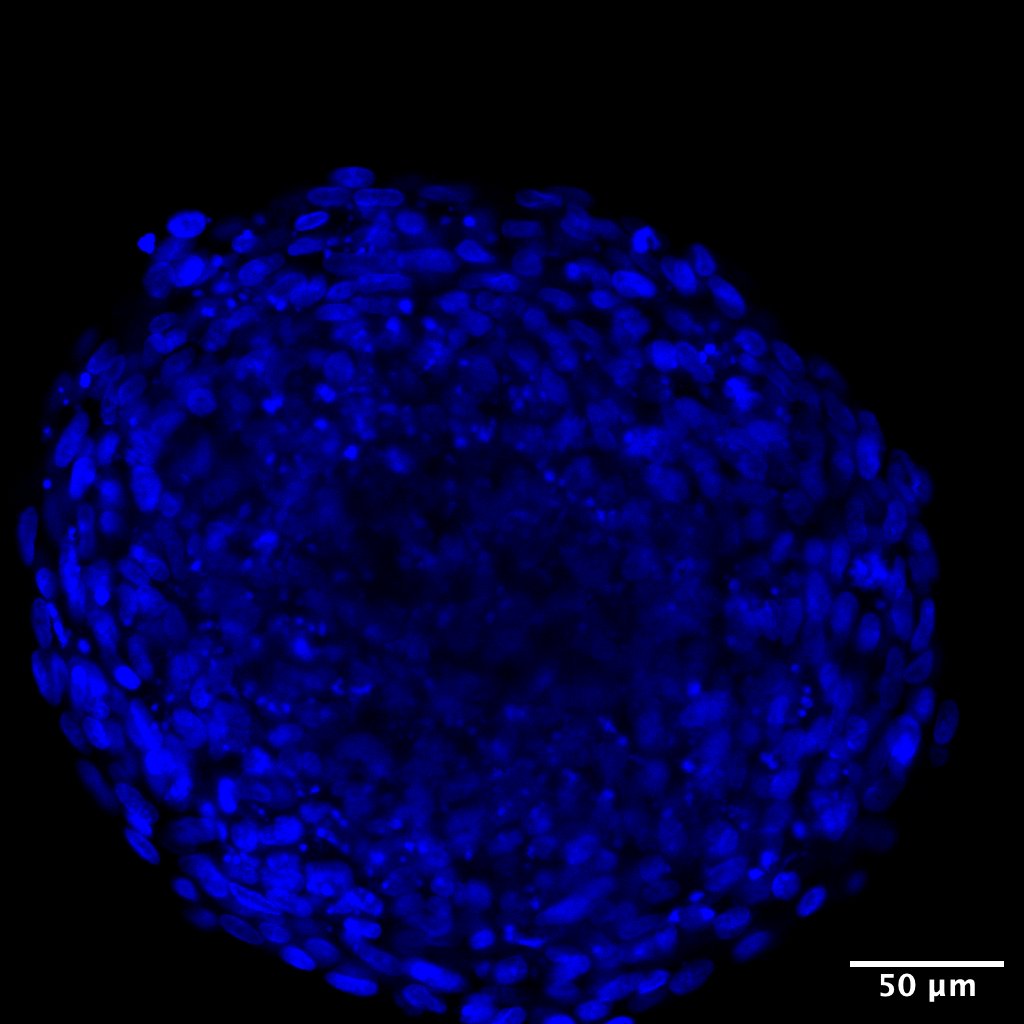

Supplement: Supplementary file 16 — Figure EV7 Source Data [file 44318_2025_570_MOESM16_ESM.zip › FigEV7/Images/D/Fig_EV7_panel_d_RP296bis_0nM_2_blue.jpg]

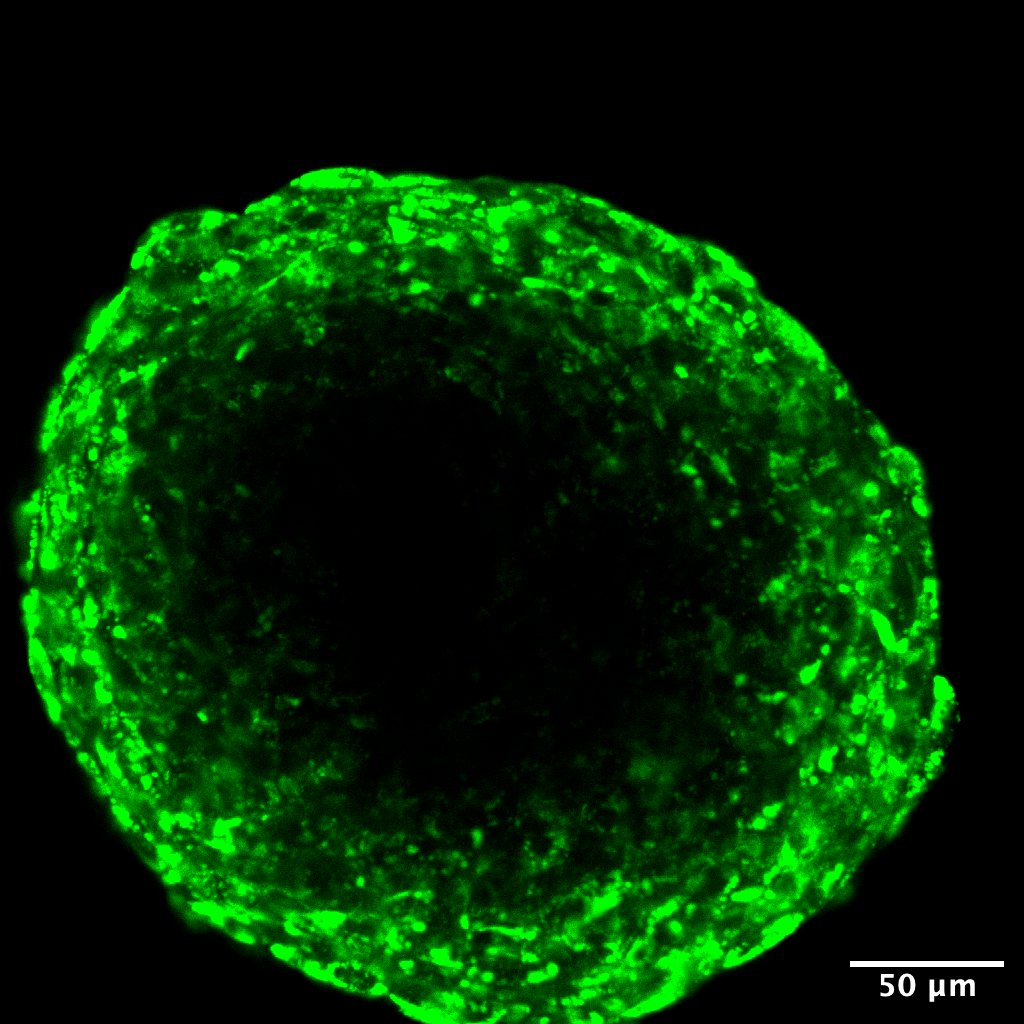

Supplement: Supplementary file 16 — Figure EV7 Source Data [file 44318_2025_570_MOESM16_ESM.zip › FigEV7/Images/D/Fig_EV7_panel_d_RP296bis_0nM_2_green.jpg]

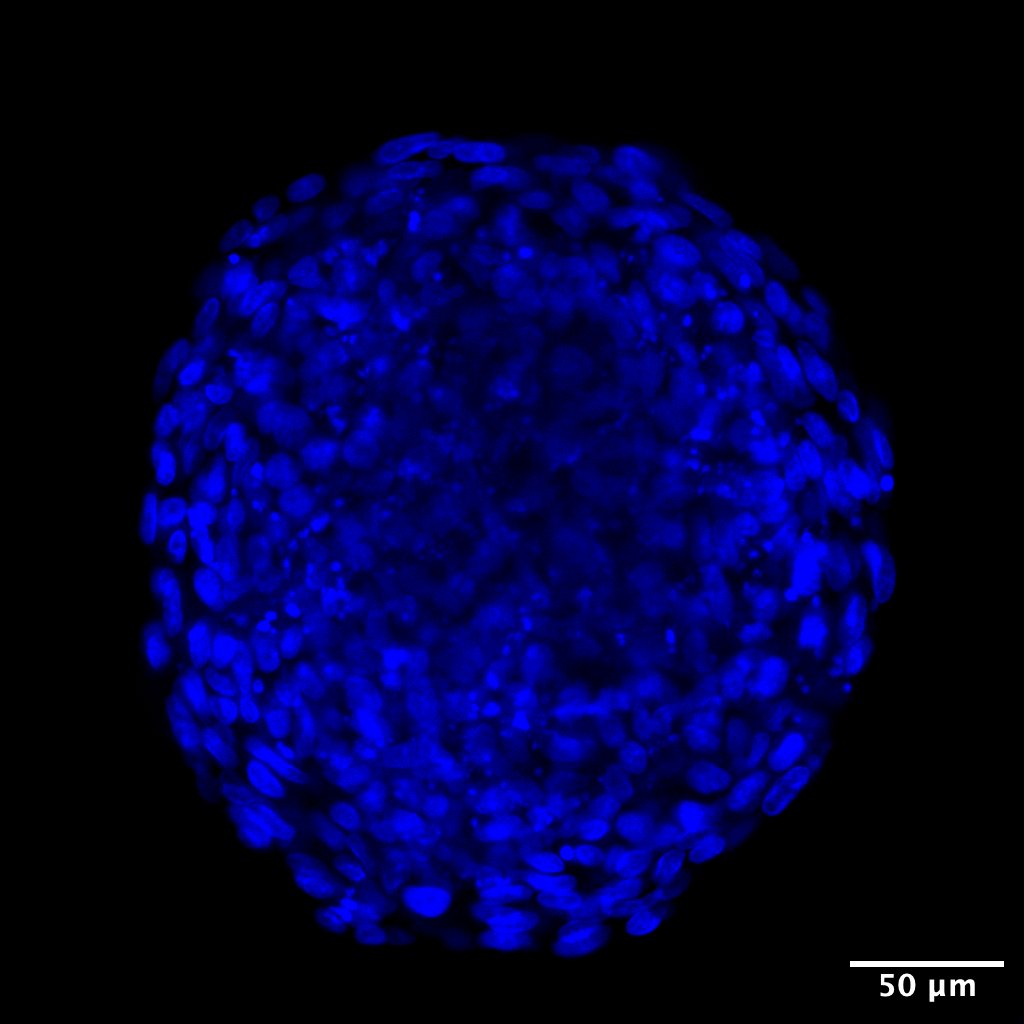

Supplement: Supplementary file 16 — Figure EV7 Source Data [file 44318_2025_570_MOESM16_ESM.zip › FigEV7/Images/D/Fig_EV7_panel_d_RP296bis_10nM_5_blue.jpg]

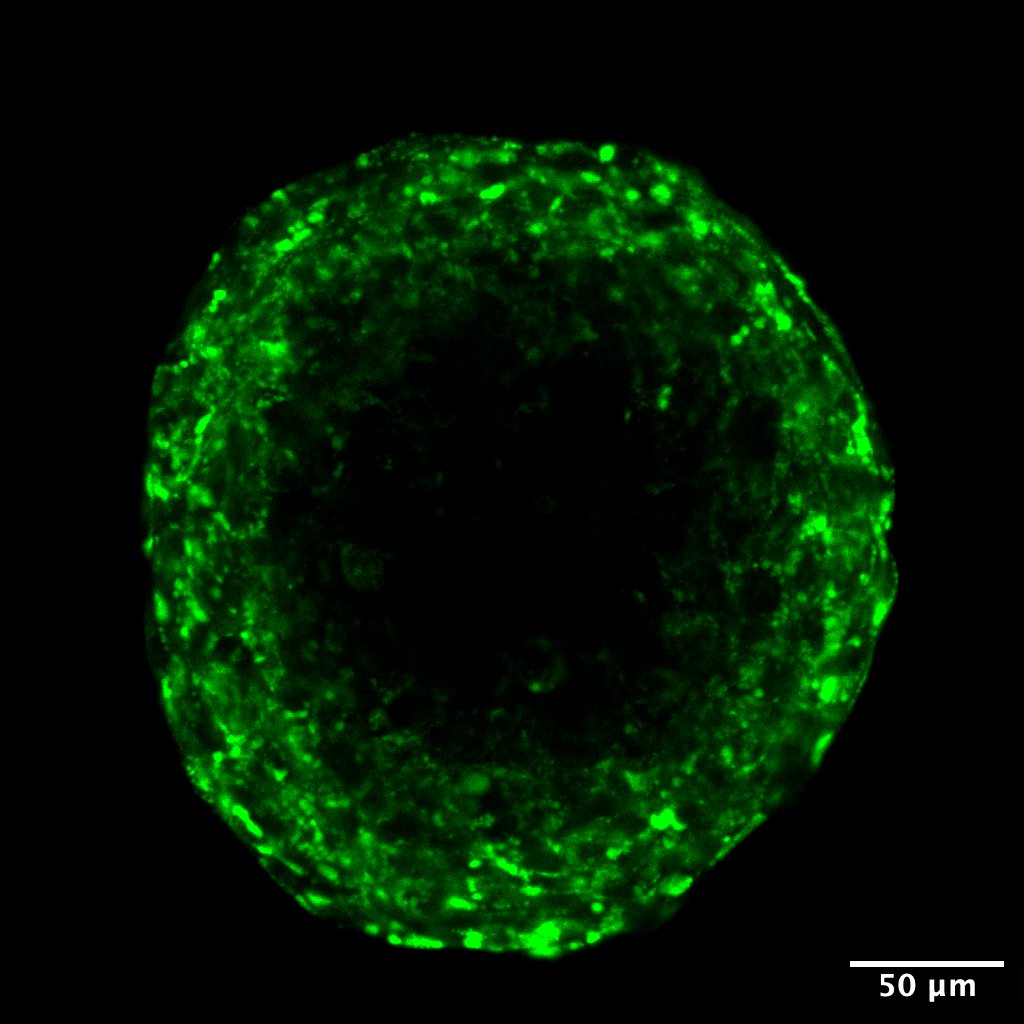

Supplement: Supplementary file 16 — Figure EV7 Source Data [file 44318_2025_570_MOESM16_ESM.zip › FigEV7/Images/D/Fig_EV7_panel_d_RP296bis_10nM_5_green.jpg]

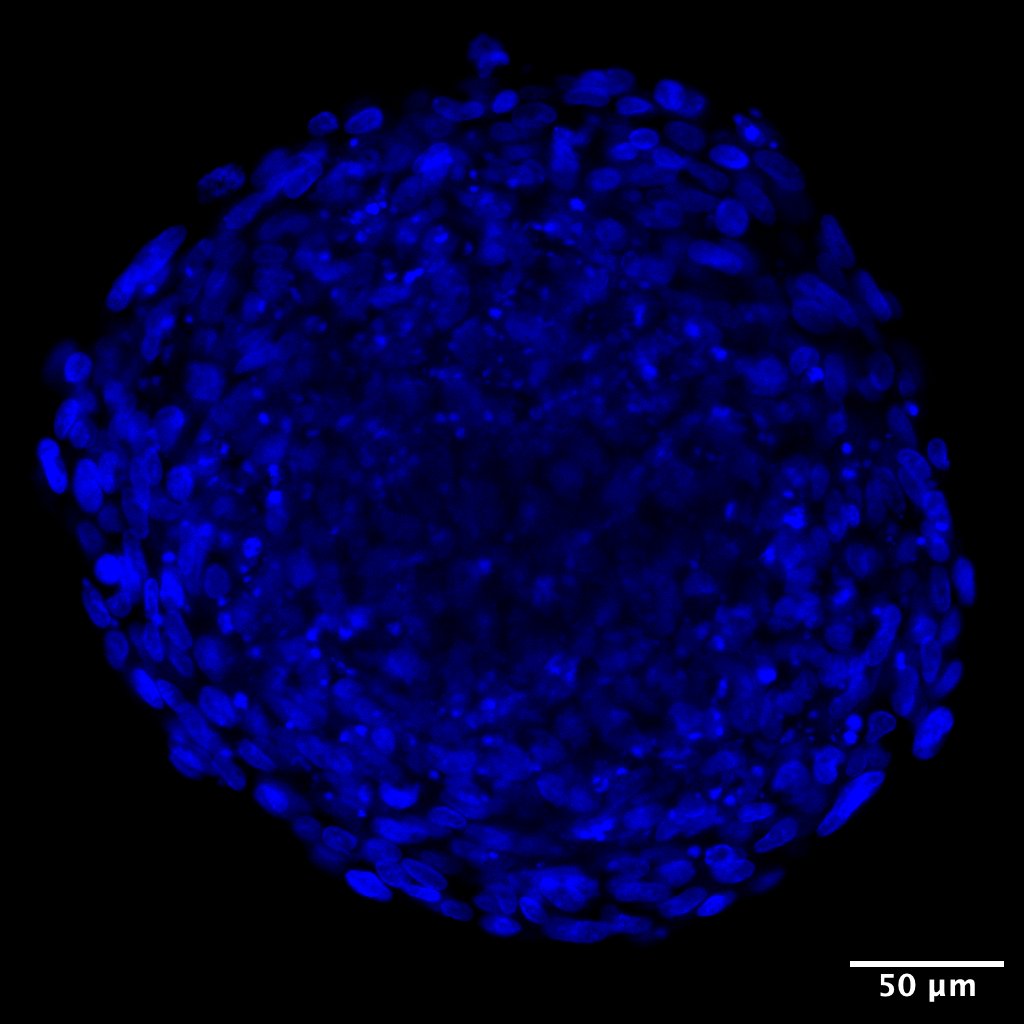

Supplement: Supplementary file 16 — Figure EV7 Source Data [file 44318_2025_570_MOESM16_ESM.zip › FigEV7/Images/D/Fig_EV7_panel_d_RP296bis_1nM_1_blue.jpg]

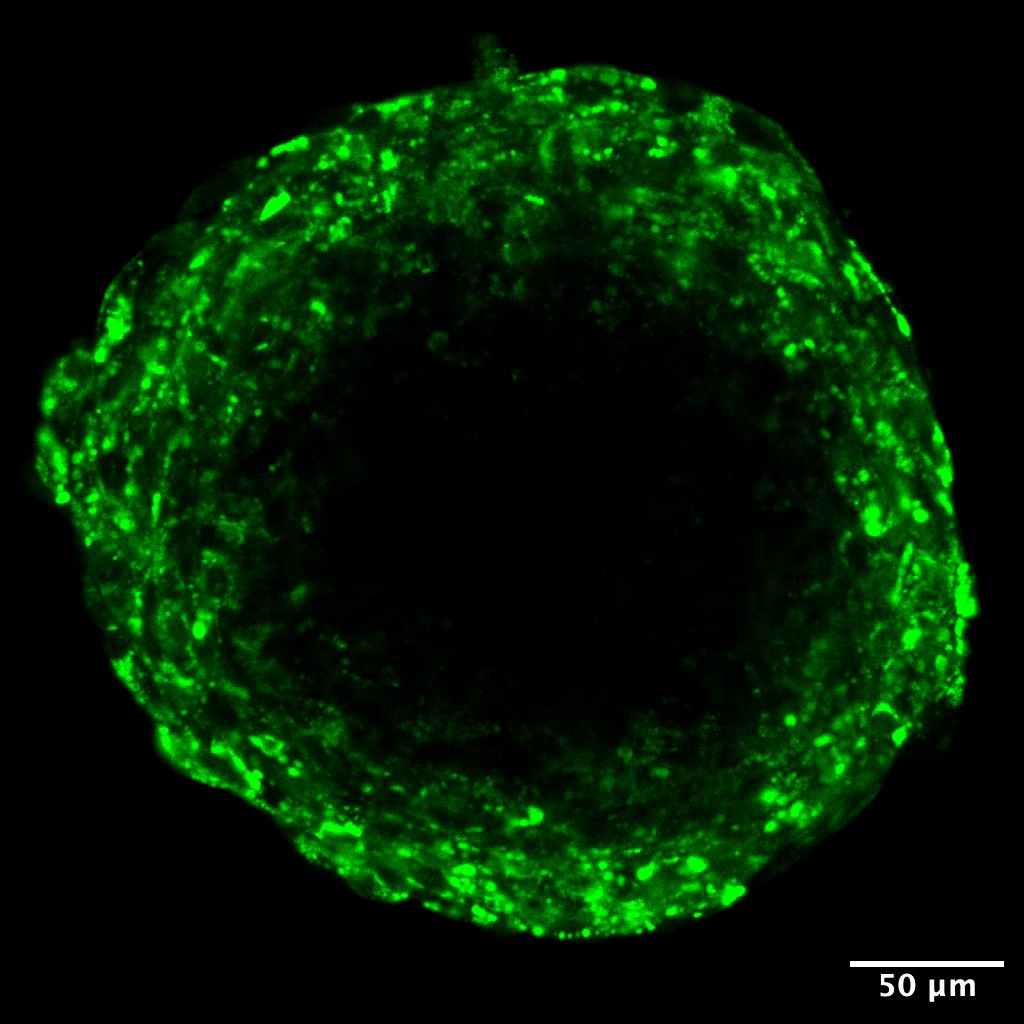

Supplement: Supplementary file 16 — Figure EV7 Source Data [file 44318_2025_570_MOESM16_ESM.zip › FigEV7/Images/D/Fig_EV7_panel_d_RP296bis_1nM_1_green.jpg]

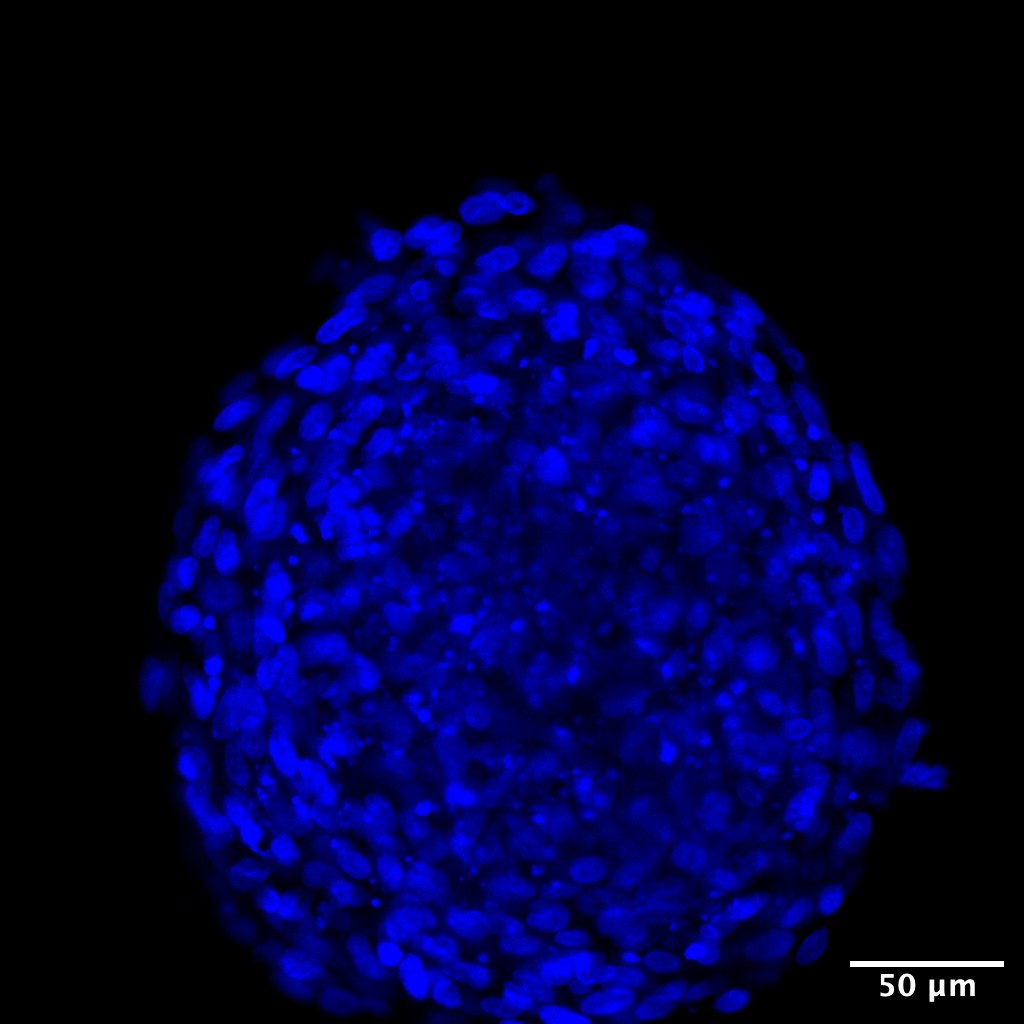

Supplement: Supplementary file 16 — Figure EV7 Source Data [file 44318_2025_570_MOESM16_ESM.zip › FigEV7/Images/D/Fig_EV7_panel_d_RP296bis_25nM_7_blue.jpg]

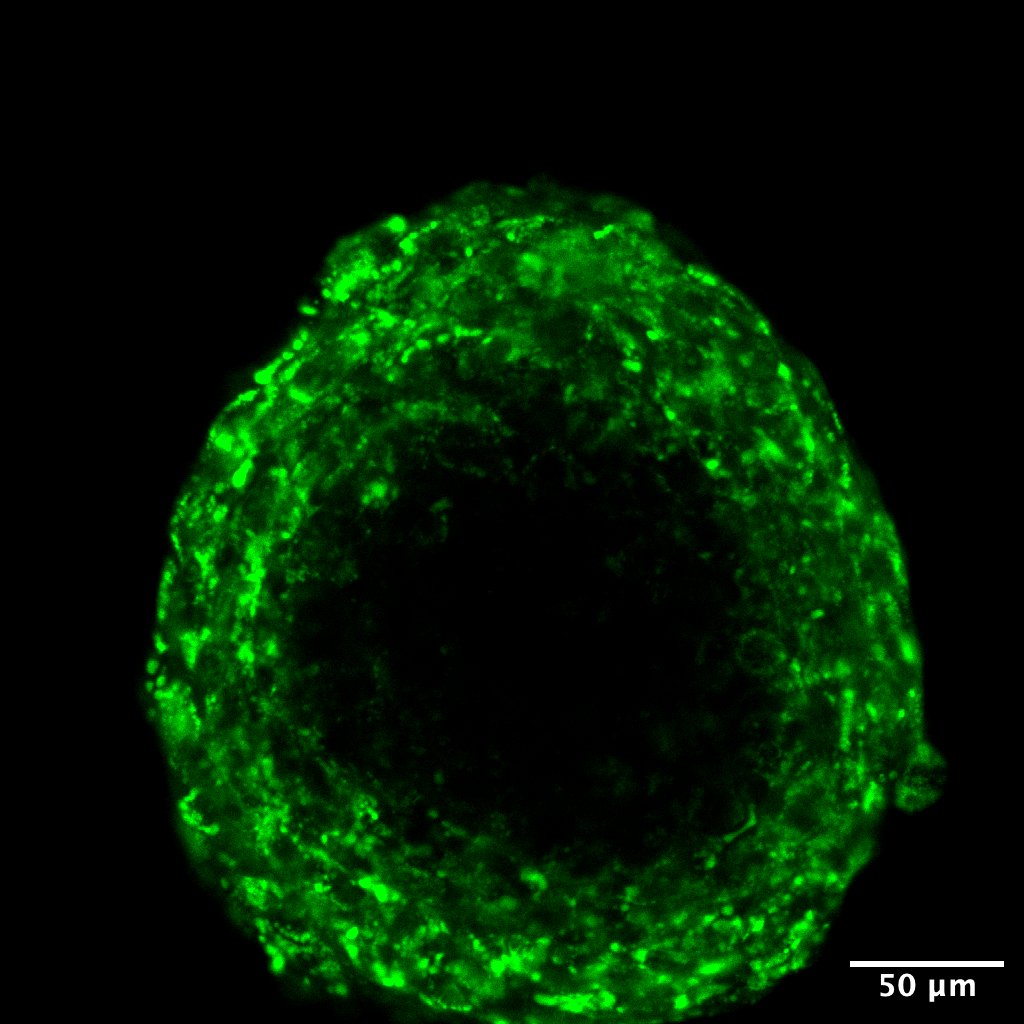

Supplement: Supplementary file 16 — Figure EV7 Source Data [file 44318_2025_570_MOESM16_ESM.zip › FigEV7/Images/D/Fig_EV7_panel_d_RP296bis_25nM_7_green.jpg]

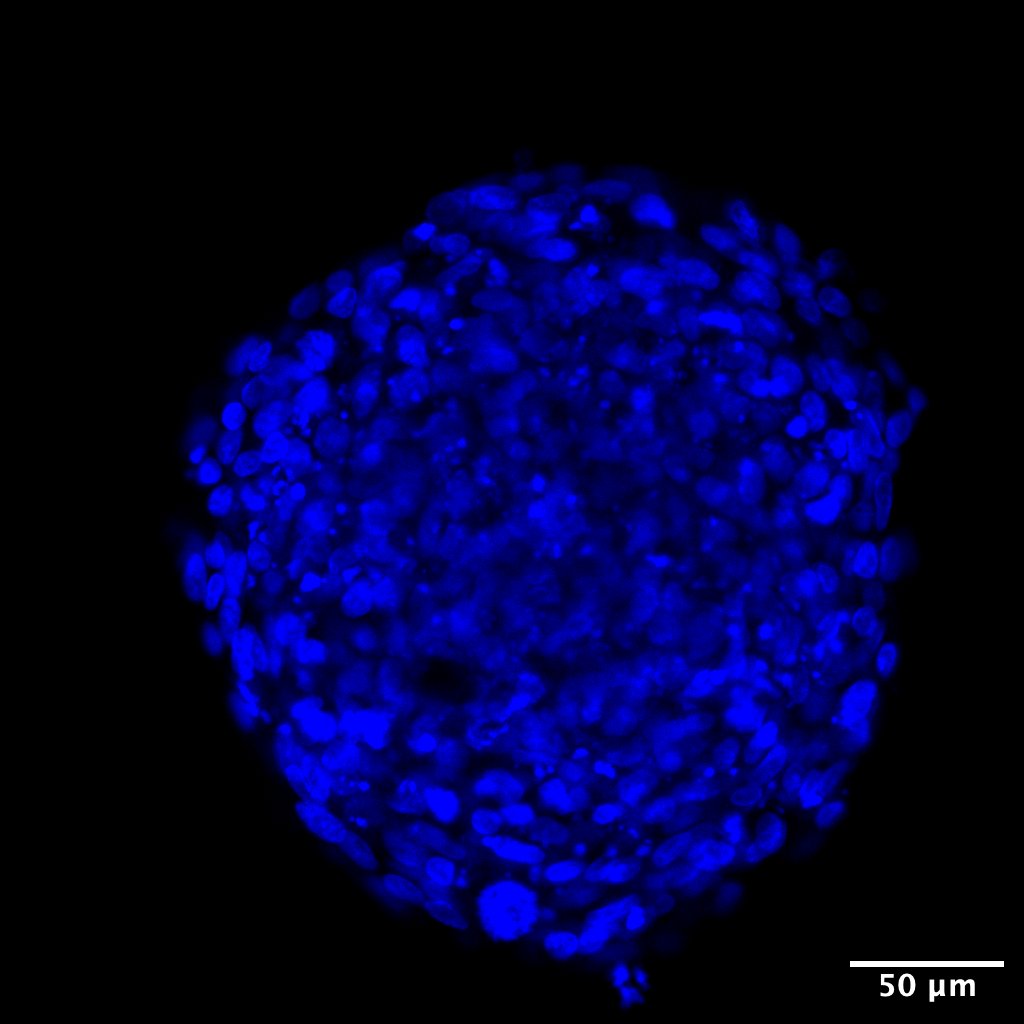

Supplement: Supplementary file 16 — Figure EV7 Source Data [file 44318_2025_570_MOESM16_ESM.zip › FigEV7/Images/D/Fig_EV7_panel_d_RP296bis_50nM_8_blue.jpg]

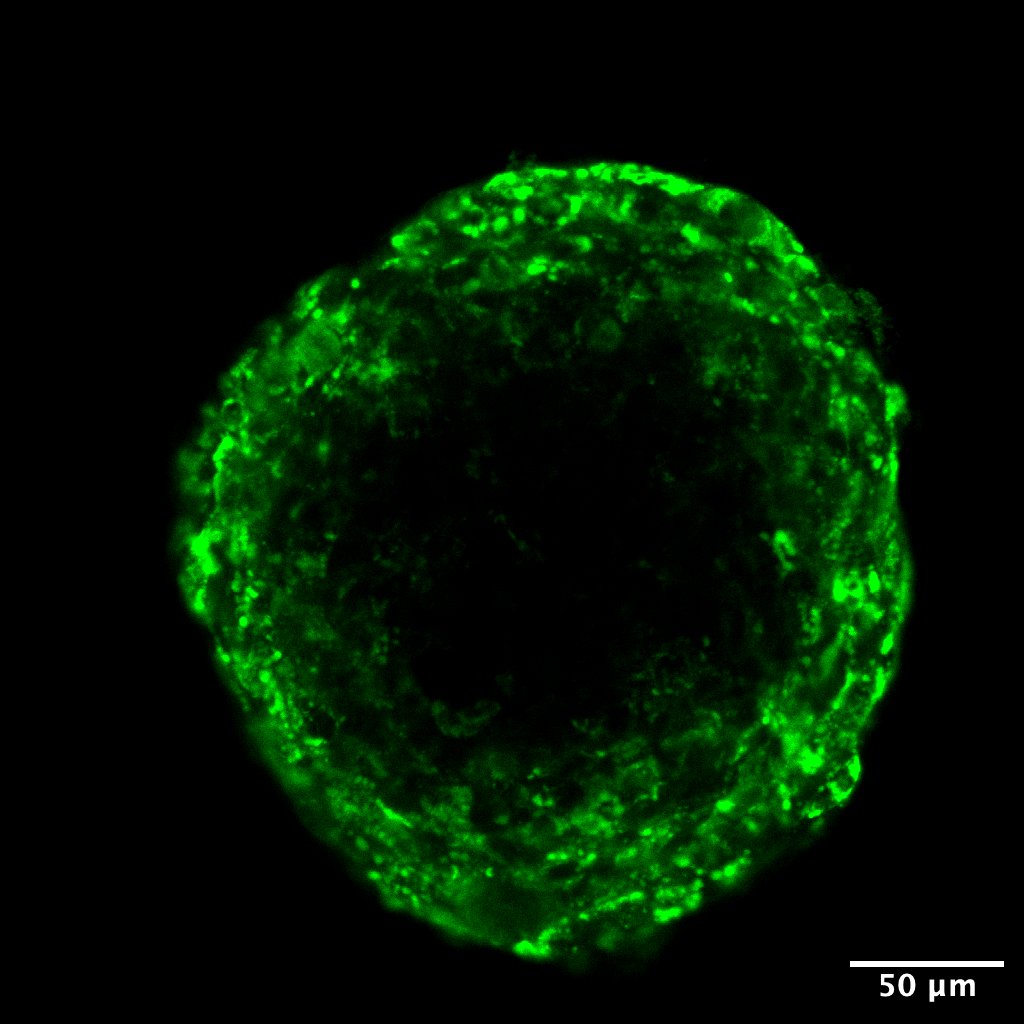

Supplement: Supplementary file 16 — Figure EV7 Source Data [file 44318_2025_570_MOESM16_ESM.zip › FigEV7/Images/D/Fig_EV7_panel_d_RP296bis_50nM_8_green.jpg]
