# Supplementary material for: Targeting myoferlin in ER/Golgi vesicle trafficking reprograms pancreatic cancer-associated fibroblasts
Source: EMBO J. 2025 Oct 8;44(22):6425–65. doi: 10.1038/s44318-025-00570-6 (PMC12623807; doi:10.1038/s44318-025-00570-6)
Supplement: Supplementary file 12 — Figure EV3 Source Data [file 44318_2025_570_MOESM12_ESM.zip › FigEV3/Western_blot/FigEV3_uncropped_blots.pptx]

## Slide 1
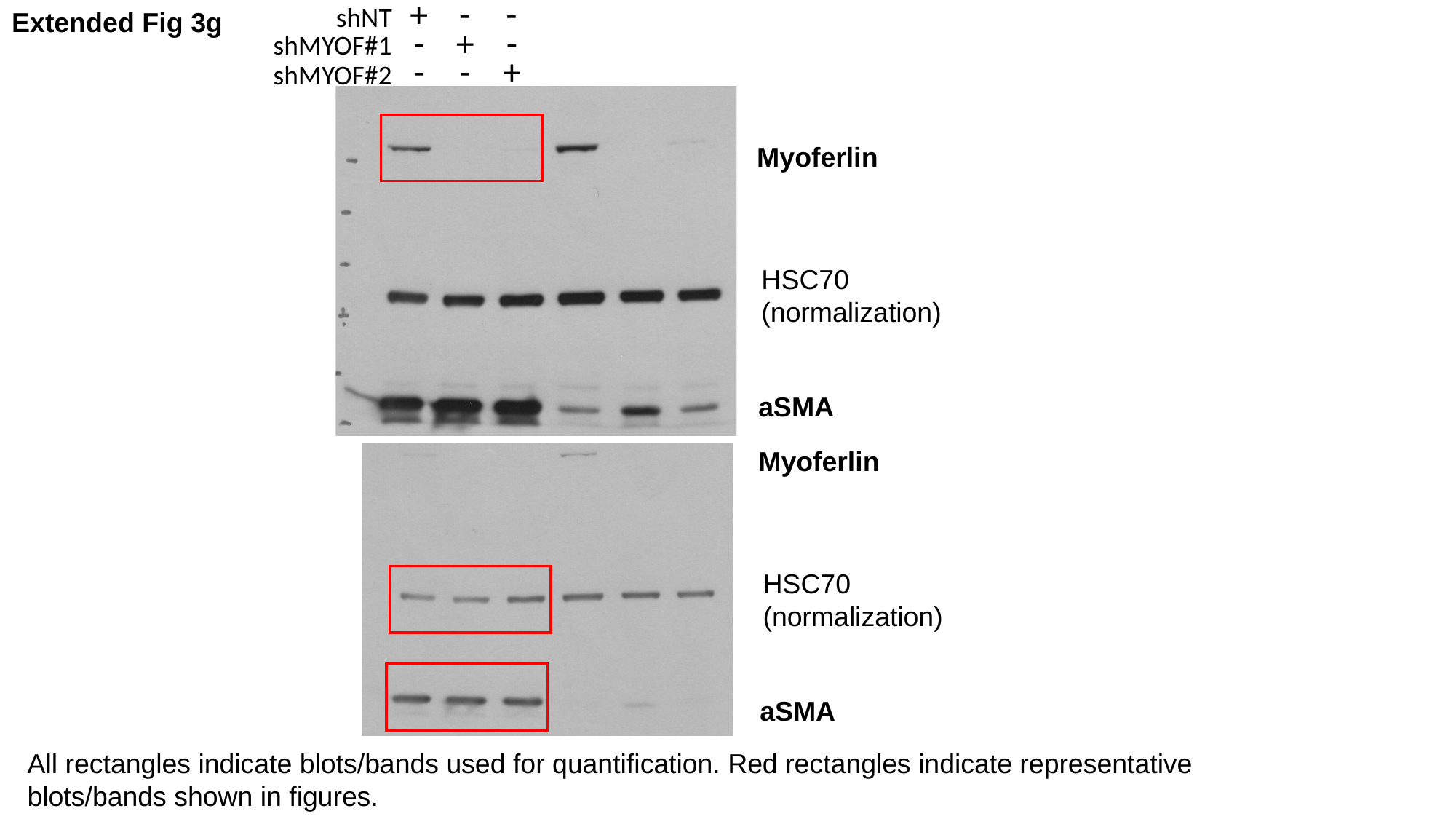

Extended Fig 3g
+
-
-
shNT
-
+
-
shMYOF#1
-
-
+
shMYOF#2
Myoferlin
HSC70
(normalization)
aSMA
Myoferlin
HSC70
(normalization)
aSMA
All rectangles indicate blots/bands used for quantification. Red rectangles indicate representative blots/bands shown in figures.

## Slide 2
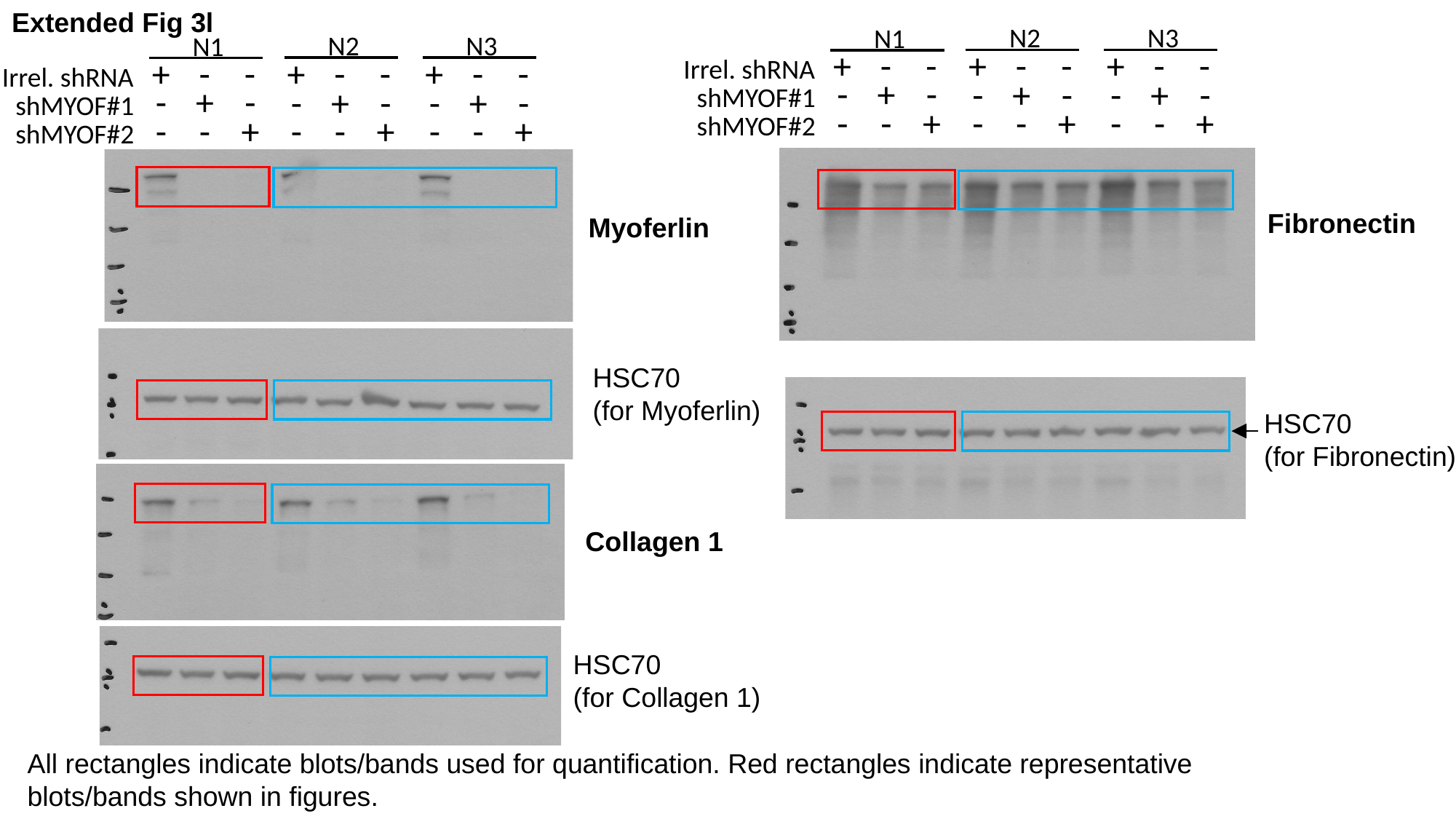

Extended Fig 3l
N2
N3
N1
N2
N3
N1
+
-
-
+
-
-
+
-
-
Irrel. shRNA
+
-
-
+
-
-
+
-
-
Irrel. shRNA
-
+
-
-
+
-
-
+
-
shMYOF#1
-
+
-
-
+
-
-
+
-
shMYOF#1
-
-
+
-
-
+
-
-
+
shMYOF#2
-
-
+
-
-
+
-
-
+
shMYOF#2
Fibronectin
Myoferlin
HSC70
(for Myoferlin)
HSC70
(for Fibronectin)
Collagen 1
HSC70
(for Collagen 1)
All rectangles indicate blots/bands used for quantification. Red rectangles indicate representative blots/bands shown in figures.
