# Supplementary material for: Targeting myoferlin in ER/Golgi vesicle trafficking reprograms pancreatic cancer-associated fibroblasts
Source: EMBO J. 2025 Oct 8;44(22):6425–65. doi: 10.1038/s44318-025-00570-6 (PMC12623807; doi:10.1038/s44318-025-00570-6)
Supplement: Supplementary file 14 — Figure EV5 Source Data [file 44318_2025_570_MOESM14_ESM.zip › FigEV5/Western_blot/FigEV5_uncropped_blots.pptx]

## Slide 1
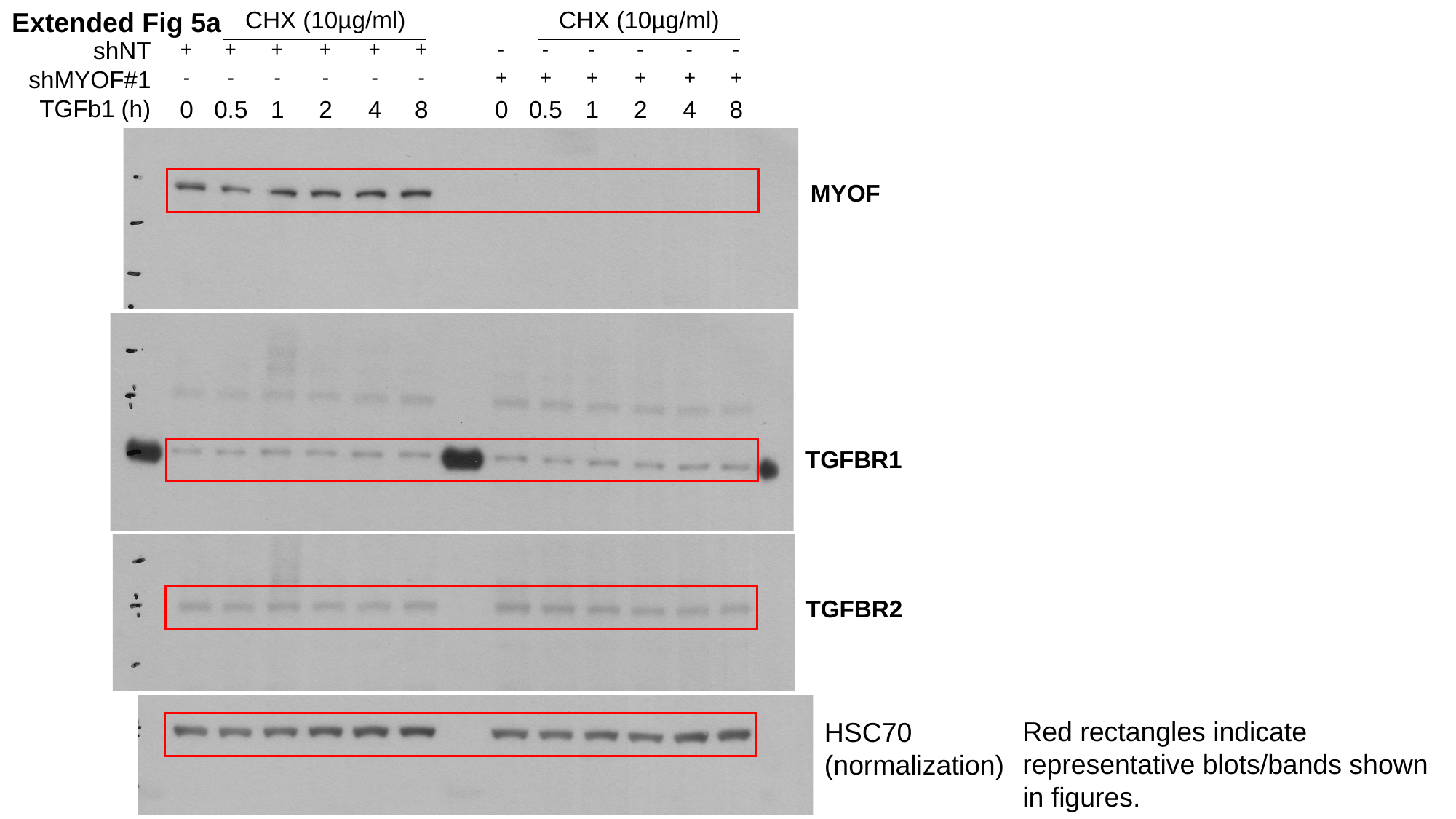

Extended Fig 5a
CHX (10µg/ml)
CHX (10µg/ml)
shNT
+
+
+
+
+
+
-
-
-
-
-
-
shMYOF#1
-
-
-
-
-
-
+
+
+
+
+
+
TGFb1 (h)
0
0.5
1
2
4
8
0
0.5
1
2
4
8
MYOF
TGFBR1
TGFBR2
Red rectangles indicate representative blots/bands shown in figures.
HSC70
(normalization)

## Slide 2
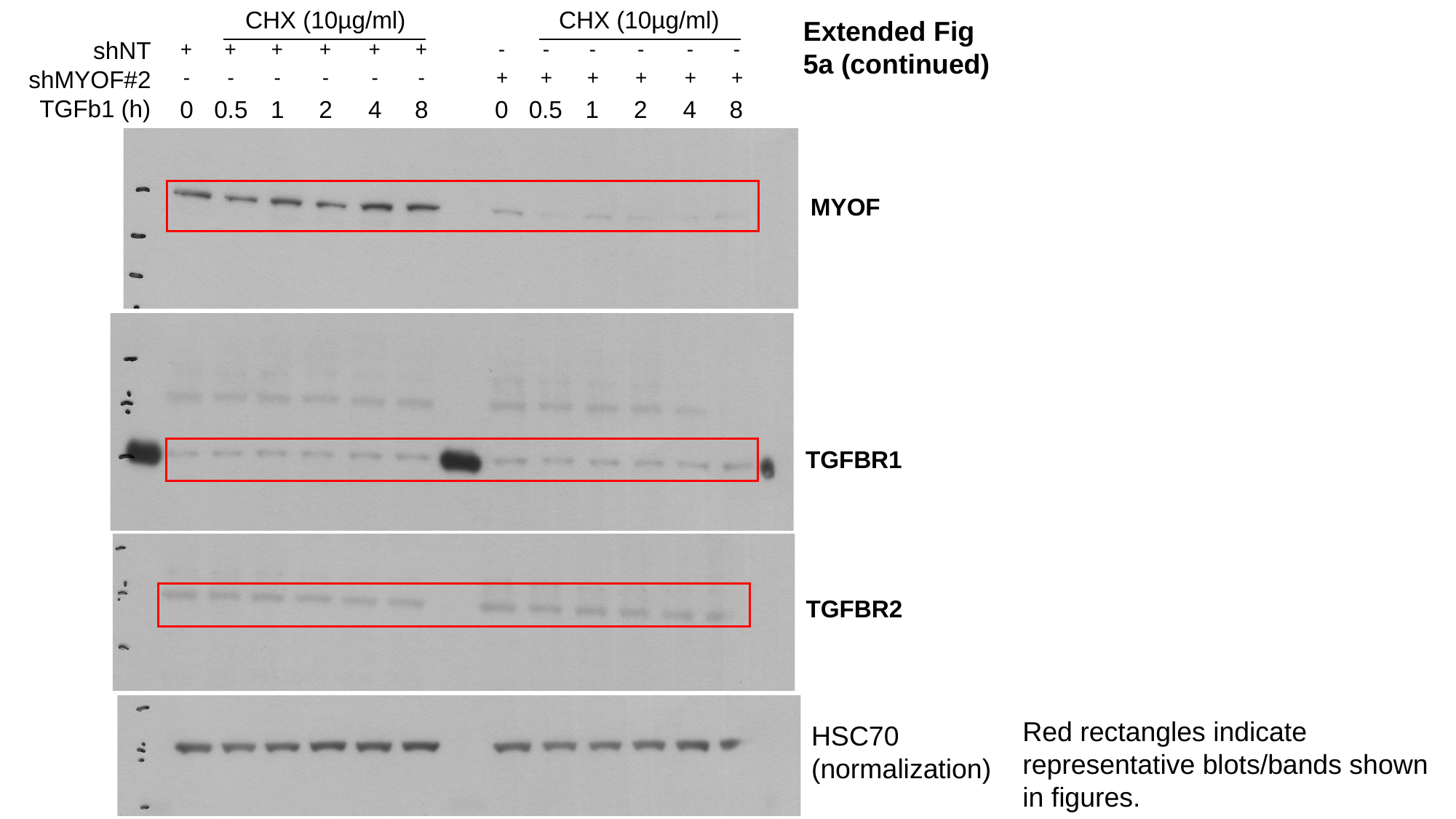

CHX (10µg/ml)
CHX (10µg/ml)
Extended Fig 5a (continued)
shNT
+
+
+
+
+
+
-
-
-
-
-
-
shMYOF#2
-
-
-
-
-
-
+
+
+
+
+
+
TGFb1 (h)
0
0.5
1
2
4
8
0
0.5
1
2
4
8
MYOF
TGFBR1
TGFBR2
Red rectangles indicate representative blots/bands shown in figures.
HSC70
(normalization)

## Slide 3
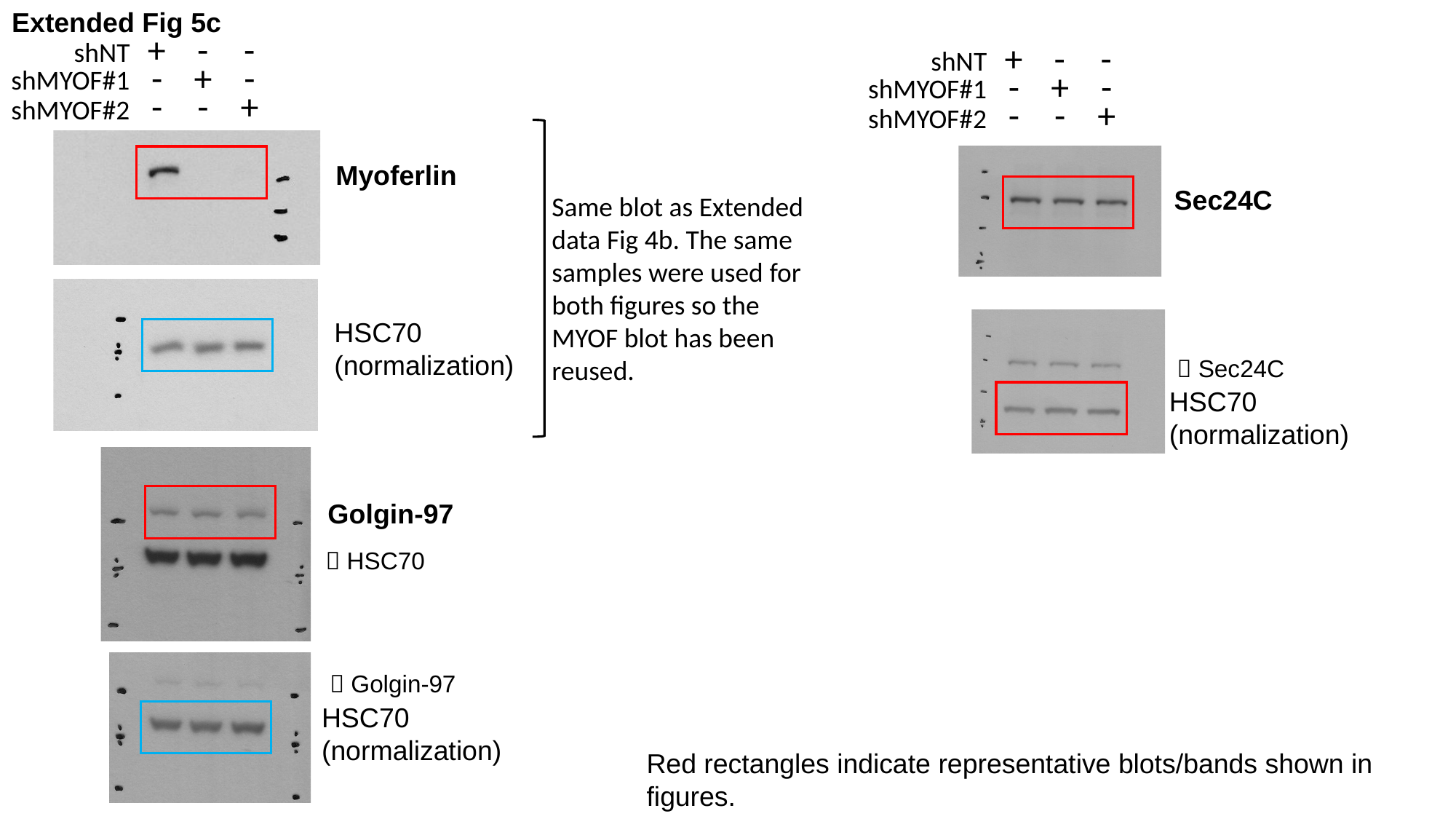

Extended Fig 5c
+
-
-
shNT
+
-
-
shNT
-
+
-
shMYOF#1
-
+
-
shMYOF#1
-
-
+
shMYOF#2
-
-
+
shMYOF#2
Myoferlin
Sec24C
Same blot as Extended data Fig 4b. The same samples were used for both figures so the MYOF blot has been reused.
HSC70
(normalization)
 Sec24C
HSC70
(normalization)
Golgin-97
 HSC70
 Golgin-97
HSC70
(normalization)
Red rectangles indicate representative blots/bands shown in figures.

## Slide 4
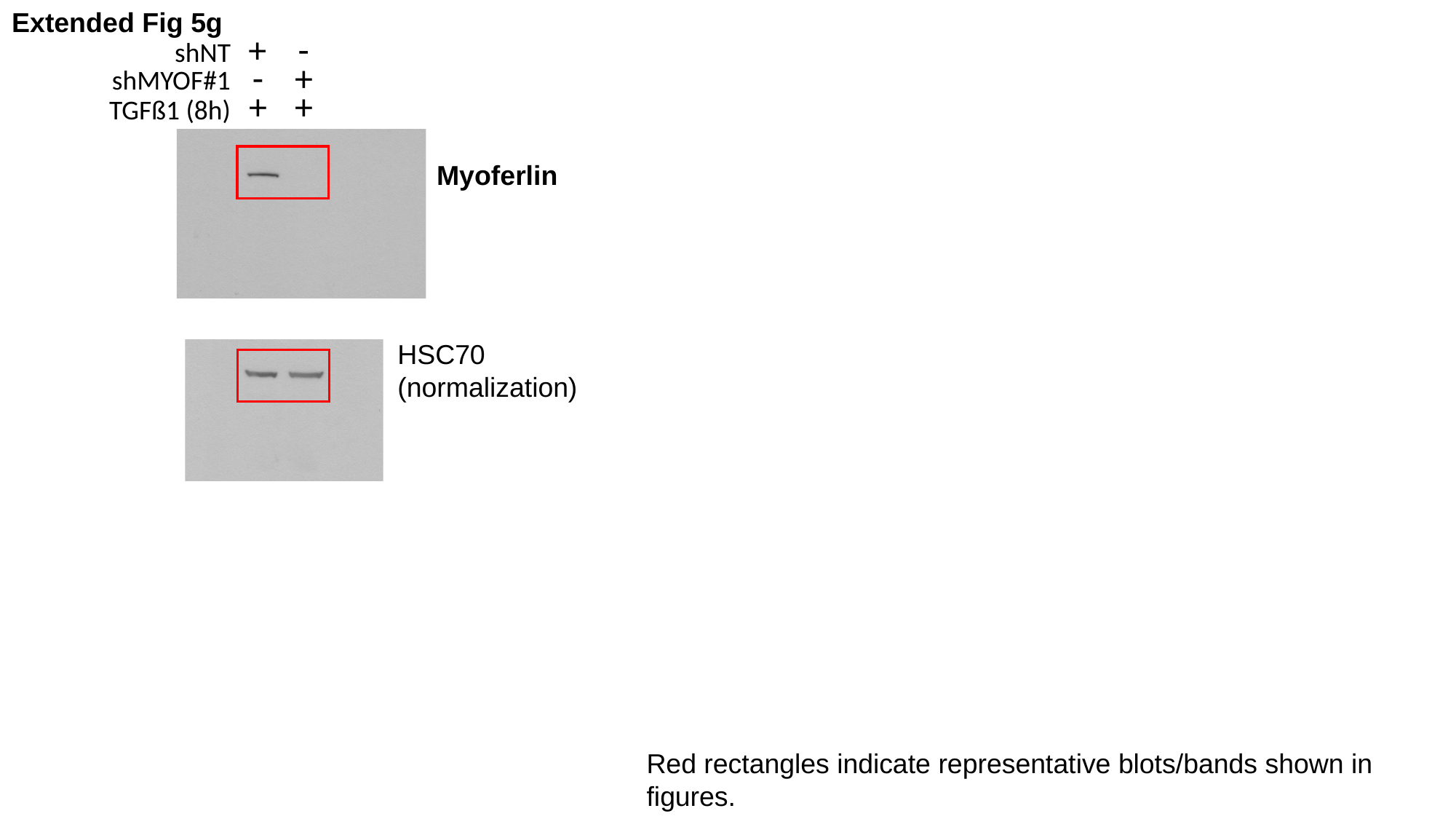

Extended Fig 5g
+
-
shNT
-
+
shMYOF#1
+
+
TGFß1 (8h)
Myoferlin
HSC70
(normalization)
Red rectangles indicate representative blots/bands shown in figures.
