# Supplementary material for: Targeting myoferlin in ER/Golgi vesicle trafficking reprograms pancreatic cancer-associated fibroblasts
Source: EMBO J. 2025 Oct 8;44(22):6425–65. doi: 10.1038/s44318-025-00570-6 (PMC12623807; doi:10.1038/s44318-025-00570-6)
Supplement: Supplementary file 16 — Figure EV7 Source Data [file 44318_2025_570_MOESM16_ESM.zip › FigEV7/Western_blot/FigEV7_uncropped_blots.pptx]

## Slide 1
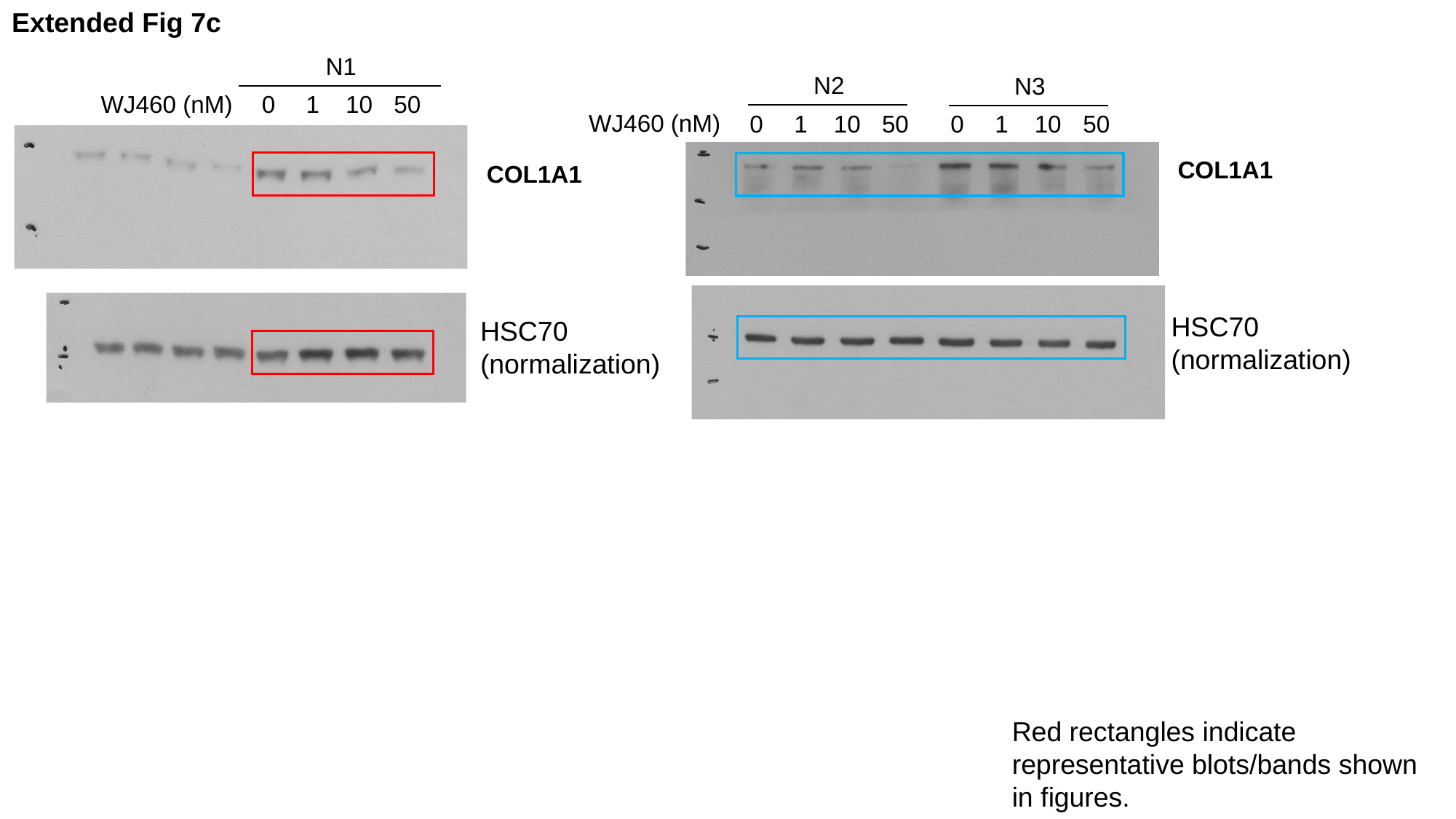

Extended Fig 7c
N1
N2
N3
WJ460 (nM)
0
1
10
50
WJ460 (nM)
0
1
10
50
0
1
10
50
COL1A1
COL1A1
HSC70
(normalization)
HSC70
(normalization)
Red rectangles indicate representative blots/bands shown in figures.
