# Supplementary material for: Targeting myoferlin in ER/Golgi vesicle trafficking reprograms pancreatic cancer-associated fibroblasts
Source: EMBO J. 2025 Oct 8;44(22):6425–65. doi: 10.1038/s44318-025-00570-6 (PMC12623807; doi:10.1038/s44318-025-00570-6)
Supplement: Supplementary file 17 — Expanded View Figures [file 44318_2025_570_MOESM17_ESM.pdf]

Expanded View Figures

Figure EV1. Myoferlin correlates with stromal features in pancreatic cancer.

(**A**) *MYOF* expression in human matched healthy and neoplastic tissue, ranked according to tumor *MYOF* expression. PAAD = pancreatic adenocarcinoma, KIRC = kidney renal clear cell carcinoma, STAD = stomach adenocarcinoma, CHOL = cholangiocarcinoma, OV = ovarian serous cystadenocarcinoma, GBM = glioblastoma, THYM = thymoma, LAML = acute myeloid leukemia, DLBC = diffuse large B-cell lymphoma, LGG = lower grade glioma. Patient numbers are indicated for each group. Boxplot (P25-1.5*IQR; P25; Median; P75; P75 + 1.5*IQR). *$P < 0.01$. (**B**) TCGA PAAD cohort patient characteristics, low *MYOF* ($n = 36$) versus high *MYOF* ($n = 37$) patients. Chi-squared test for demographic characteristics and TNM stage; Wilcoxon test for diagnosis age. (**C**) Ridgeplot of most enriched gene sets ($P < 0.0001$) in $MYOF^{high}$ patients. (**D**) ESTIMATE immune scores in TCGA PAAD cohort patients ($n = 146$) according to *MYOF* expression. Violin plot, one-way ANOVA (Tukey's test). (**E**) Percentages of tumor subtypes (Collisson et al, 2011) according to *MYOF* expression (QM = quasi-mesenchymal). Stacked bar plot, Chi-squared test. (**F**) Percentages of tumor subtypes (Moffitt et al, 2015) according to *MYOF* expression. Stacked bar plot, Chi-squared test. (**G**) Percentages of tumor subtypes (Bailey et al, 2016) according to *MYOF* expression. Stacked bar plot, Chi-squared test. (**H**) Myoferlin IHC and Masson trichrome staining (collagens stained in blue) in human PAAD sections. Representative images from 99 subTME regions across 33 patients. Scale bar = 100 µm. (**I**) IHC analysis, correlation and linear regression between Masson trichrome scores and Myoferlin stromal scores ($n = 99$). Pearson (R) and Spearman (ρ) correlation. (**J**) Internal PAAD cohort patient characteristics, $MYOF^{low}$ stroma ($n = 19$) versus $MYOF^{high}$ stroma ($n = 19$) patients. Chi-squared test for demographic characteristics and TNM stage; Wilcoxon test for diagnosis age and death/censoring age. (**K**) Linear regression between stromal myoferlin IHC scores and tumor cell myoferlin IHC scores of PAAD patients ($n = 38$). Pearson (R) and Spearman (ρ) correlation. Source data are available online for this figure.

© The Author(s)

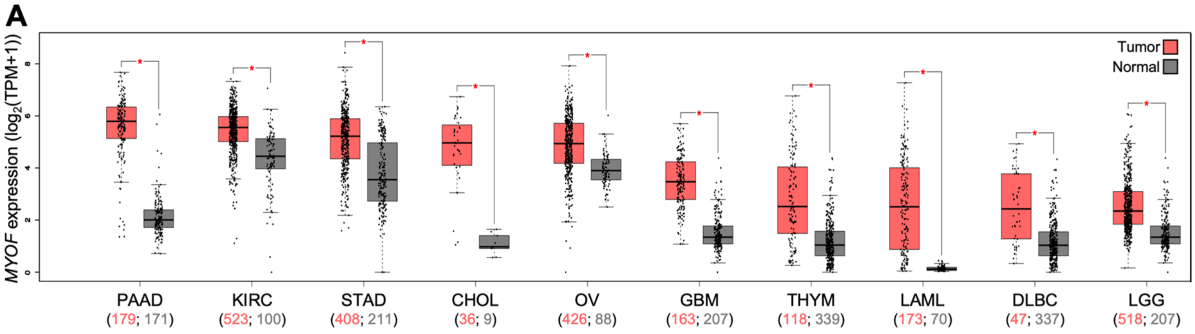

B

Characteristic	High MYOF (N=37)	Low MYOF (N=36)	q-value
**Demographic**			
Male - no. (%)	21 (56.8)	18 (50.0)	0.846
Caucasian - no. (%)	33 (97.1)	31 (88.6)	0.527
**Clinical**			
Diagnosis age (yrs) - median (IQR)	63 (52-73)	70 (61-74)	0.116
TNM stage (7th AJCC) - no. (%)			0.93
NA	0	1 (2.8)	
IA	0	1 (2.8)	
IB	2 (5.4)	2 (5.6)	
IIA	7 (18.9)	7 (19.4)	
IIB	26 (70.3)	24 (66.7)	
III	1 (2.7)	1 (2.8)	
IV	1 (2.7)	0	

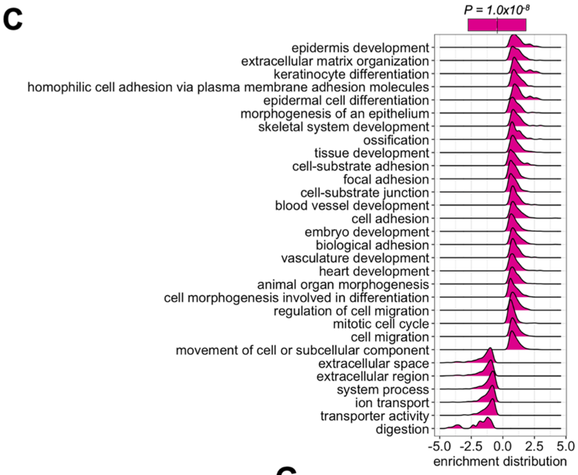

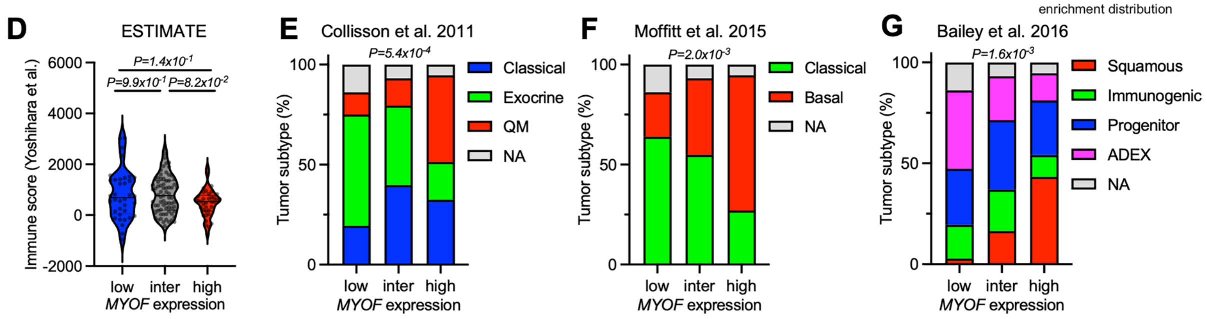

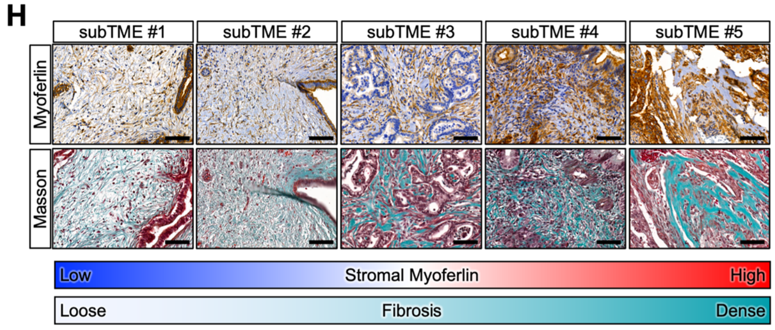

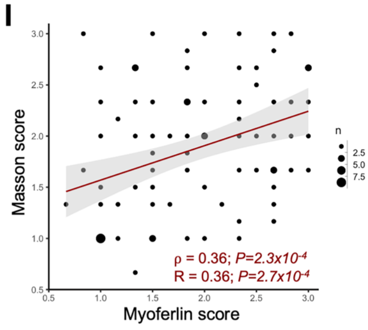

J

Characteristic	High MYOF (N=19)	Low MYOF (N=19)	p-value
**Demographic**			
Male - no. (%)	8 (42.1)	12 (63.1)	0.330
**Clinical**			
Diagnosis age (yrs) - mean (SD)	67.8 (9.7)	68.0 (11.3)	0.959
Death/Censoring age (yrs) - mean (SD)	69.5 (10.1)	70.7 (11.9)	0.744
TNM stage (7th AJCC) - no. (%)			0.165
NA	0	0	
I	0	0	
IA	0	0	
IB	0	0	
IIA	1 (5.3)	6 (31.6)	
IIB	15 (78.9)	11 (57.9)	
III	1 (5.3)	0	
IV	2 (10.5)	2 (10.5)	

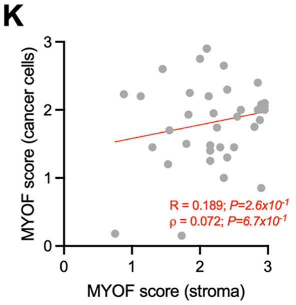

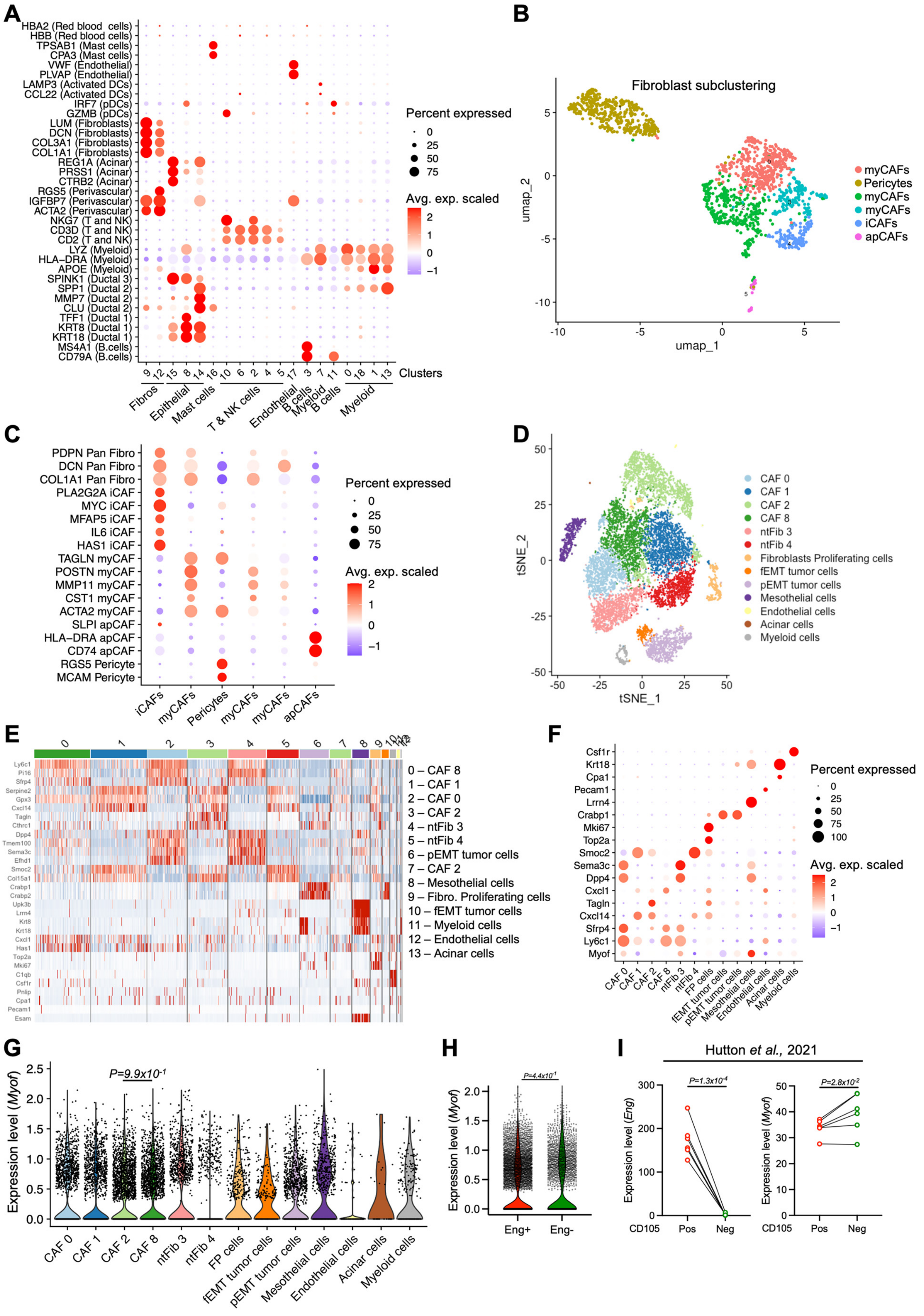
A
HBA2 (Red blood cells)
HBB (Red blood cells)
TPSAB1 (Mast cells)
CPA3 (Mast cells)
VWF (Endothelial)
PLVAP (Endothelial)
LAMP3 (Activated DCs)
CCL22 (Activated DCs)
IRF7 (pDCs)
GZMB (pDCs)
LUM (Fibroblasts)
DCN (Fibroblasts)
COL3A1 (Fibroblasts)
COL1A1 (Fibroblasts)
REG1A (Acinar)
PRSS1 (Acinar)
CTRB2 (Acinar)
RGS5 (Perivascular)
IGFBP7 (Perivascular)
ACTA2 (Perivascular)
NKG7 (T and NK)
CD3D (T and NK)
CD2 (T and NK)
LYZ (Myeloid)
HLA-DRA (Myeloid)
APOE (Myeloid)
SPINK1 (Ductal 3)
SPP1 (Ductal 2)
MMP7 (Ductal 2)
CLU (Ductal 2)
TFF1 (Ductal 1)
KRT8 (Ductal 1)
KRT18 (Ductal 1)
MS4A1 (B.cells)
CD79A (B.cells)
9 12 15 8 14 16 10 6 2 4 5 17 3 7 11 0 18 1 13 Clusters
Fibros
Epithelial
Mast cells
T & NK cells
Endothelial
B cells
Myeloid
B cells
Myeloid
Percent expressed
0
25
50
75
Avg. exp. scaled
2
1
0
−1
B
Fibroblast subclustering
umap_2
umap_1
myCAFs
Pericytes
myCAFs
myCAFs
iCAFs
apCAFs
C
PDPN Pan Fibro
DCN Pan Fibro
COL1A1 Pan Fibro
PLA2G2A iCAF
MYC iCAF
MFAP5 iCAF
IL6 iCAF
HAS1 iCAF
TAGLN myCAF
POSTN myCAF
MMP11 myCAF
CST1 myCAF
ACTA2 myCAF
SLPI apCAF
HLA-DRA apCAF
CD74 apCAF
RGS5 Pericyte
MCAM Pericyte
iCAFs
myCAFs
Pericytes
myCAFs
myCAFs
apCAFs
Percent expressed
0
25
50
75
Avg. exp. scaled
2
1
0
−1
D
tSNE_2
tSNE_1
CAF 0
CAF 1
CAF 2
CAF 8
ntFib 3
ntFib 4
Fibroblasts Proliferating cells
fEMT tumor cells
pEMT tumor cells
Mesothelial cells
Endothelial cells
Acinar cells
Myeloid cells
E
0 – CAF 8
1 – CAF 1
2 – CAF 0
3 – CAF 2
4 – ntFib 3
5 – ntFib 4
6 – pEMT tumor cells
7 – CAF 2
8 – Mesothelial cells
9 – Fibro. Proliferating cells
10 – fEMT tumor cells
11 – Myeloid cells
12 – Endothelial cells
13 – Acinar cells
Ly6c1
Pi16
Sfrp4
Serpine2
Gpx3
Cxcl14
Tagln
Cthrc1
Dpp4
Tmem100
Sema3c
Efhd1
Smoc2
Col15a1
Crabp1
Crabp2
Upk3b
Lrrn4
Krt8
Krt18
Cxcl1
Has1
Top2a
Mki67
C1qb
Csf1r
Pnlip
Cpa1
Pecam1
Esam
F
Csf1r
Krt18
Cpa1
Pecam1
Lrrn4
Crabp1
Mki67
Top2a
Smoc2
Sema3c
Dpp4
Cxcl1
Tagln
Cxcl14
Sfrp4
Ly6c1
Myof
CAF 0
CAF 1
CAF 2
CAF 8
ntFib 3
ntFib 4
FP cells
fEMT tumor cells
pEMT tumor cells
Mesothelial cells
Endothelial cells
Acinar cells
Myeloid cells
Percent expressed
0
25
50
75
100
Avg. exp. scaled
2
1
0
−1
G
Expression level (Myof)
P=9.9x10-1
CAF 0
CAF 1
CAF 2
CAF 8
ntFib 3
ntFib 4
FP cells
fEMT tumor cells
pEMT tumor cells
Mesothelial cells
Endothelial cells
Acinar cells
Myeloid cells
H
Expression level (Myof)
P=4.4x10-1
Eng+
Eng-
I
Hutton et al., 2021
Expression level (Eng)
P=1.3x10-4
Expression level (Myof)
P=2.8x10-2
CD105
Pos
Neg

 © The Author(s)

Figure EV2. Myoferlin expression in pancreatic cancer at single cell resolution.

(**A**) Marker genes for broad cell type labeling from Fig. 2A. Dot plot. (**B**) UMAP plot for fibroblasts subclustering from human PAAD scRNAseq data. (**C**) Marker genes for fibroblasts subclustering from (**B**). Dot plot. (**D**) t-SNE plot and cluster labeling for murine PAAD scRNAseq data. iCAF lineage: ntFib3 = normal Fibroblasts; CAF0 = early CAFs; CAF8 = IL1 CAFs. myCAF lineage: ntFib4 = normal Fibroblasts; CAF1 = early CAFs; CAF2 = TGFß CAFs. (**E**) Marker genes for clusters from (**D**). Heatmap. (**F**) *Myof* expression and marker genes for clusters from (**D**). Dot plot. (**G**) *Myof* expression in clusters from (**D**). Violin plot, pairwise comparisons using Wilcoxon Rank test (Bonferroni correction). (**H**) Normalized *Myof* expression levels in fibroblasts clustered according to CD105 (Eng) expression (0.05 threshold for Eng +). Violin plot, pairwise comparisons using Wilcoxon Rank test (Bonferroni correction). (**I**) Normalized *Eng* and *Myof* expression levels in CyTOF-isolated murine $CD105^{pos}$ and $CD105^{neg}$ CAFs. Paired *T* test. Source data are available online for this figure.

© The Author(s)

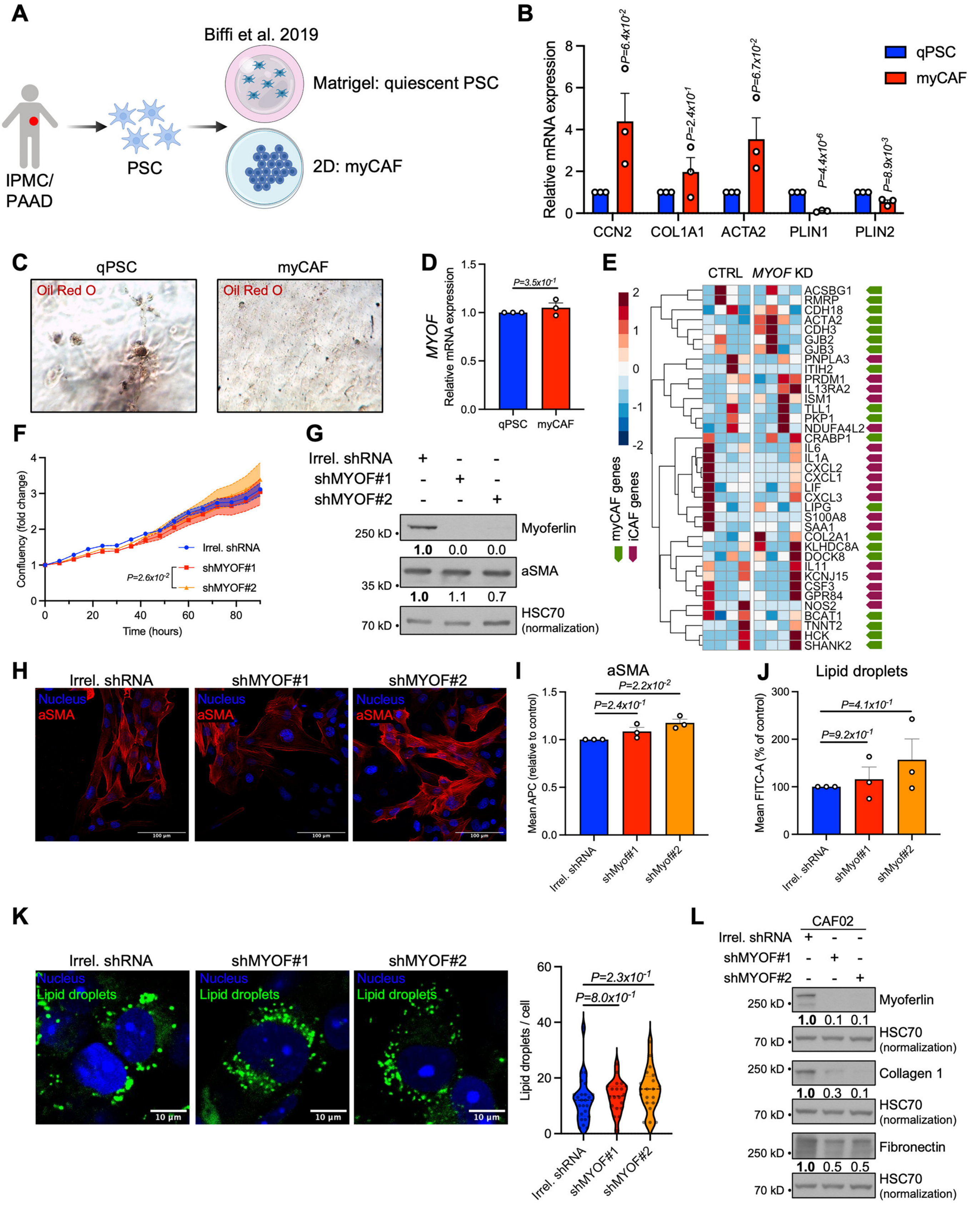
A
Biffi et al. 2019
Matrigel: quiescent PSC
2D: myCAF
IPMC/
PAAD
PSC
B
Relative mRNA expression
qPSC
myCAF
P=6.4x10-2
P=2.4x10-1
P=6.7x10-2
P=4.4x10-6
P=8.9x10-3
CCN2
COL1A1
ACTA2
PLIN1
PLIN2
C
qPSC
myCAF
Oil Red O
D
MYOF Relative mRNA expression
P=3.5x10-1
qPSC
myCAF
E
CTRL
MYOF KD
myCAF genes
iCAF genes
ACSBG1
RMRP
CDH18
ACTA2
CDH3
GJB2
GJB3
PNPLA3
ITIH2
PRDM1
IL13RA2
ISM1
TLL1
PKP1
NDUFA4L2
CRABP1
IL6
IL1A
CXCL2
CXCL1
LIF
CXCL3
LIPG
S100A8
SAA1
COL2A1
KLHDC8A
DOCK8
IL11
KCNJ15
CSF3
GPR84
NOS2
BCAT1
TNNT2
HCK
SHANK2
F
Confluency (fold change)
Time (hours)
Irrel. shRNA
shMYOF#1
shMYOF#2
P=2.6x10-2
G
Irrel. shRNA
shMYOF#1
shMYOF#2
250 kD
Myoferlin
1.0 0.0 0.0
35 kD
aSMA
1.0 1.1 0.7
70 kD
HSC70
(normalization)
H
Irrel. shRNA
shMYOF#1
shMYOF#2
Nucleus
aSMA
100 µm
I
aSMA
Mean APC (relative to control)
P=2.2x10-2
P=2.4x10-1
Irrel. shRNA
shMyof#1
shMyof#2
J
Lipid droplets
Mean FITC-A (% of control)
P=4.1x10-1
P=9.2x10-1
Irrel. shRNA
shMyof#1
shMyof#2
K
Irrel. shRNA
shMYOF#1
shMYOF#2
Nucleus
Lipid droplets
10 µm
Lipid droplets / cell
P=2.3x10-1
P=8.0x10-1
Irrel. shRNA
shMYOF#1
shMYOF#2
L
CAF02
Irrel. shRNA
shMYOF#1
shMYOF#2
250 kD
Myoferlin
1.0 0.1 0.1
70 kD
HSC70
(normalization)
250 kD
Collagen 1
1.0 0.3 0.1
70 kD
HSC70
(normalization)
250 kD
Fibronectin
1.0 0.5 0.5
70 kD
HSC70
(normalization)

 © The Author(s)

Figure EV3. Myoferlin knockdown does not induce CAF quiescence.

(**A**) Culture model for PSC-derived myCAFs. (**B**) RT-qPCR analysis of *CCN2*, *COL1A1*, *ACTA2*, *PLIN1* and *PLIN2* mRNA levels in qPSCs ($n = 3$) and myCAFs ($n = 3$). Mean ± SEM, unpaired *T* test (*P* values relative to qPSC). (**C**) Oil Red O staining of lipid droplets in qPSCs and myCAFs. (**D**) RT-qPCR analysis of *MYOF* mRNA levels in qPSCs ($n = 3$) and myCAFs ($n = 3$). Mean ± SEM, unpaired *T* test. (**E**) Heatmap and unsupervised clustering of myCAF and iCAF signature genes in CTRL myCAFs ($n = 4$) and $MYOF^{KD}$ myCAFs (siMYOF#1; $n = 4$). Gene expression values are z-score normalized. (**F**) Confluency-based cell proliferation analysis of CTRL myCAFs (Irrel. shRNA; $n = 3$) and $MYOF^{KD}$ myCAFs (shMYOF#1 and shMYOF#2; $n = 3$ each). Mean ± SEM (dashed lines), two-way ANOVA (Tukey's test). (**G**) Western blot analysis of CTRL myCAFs (Irrel. shRNA) and $MYOF^{KD}$ myCAFs (shMYOF#1 and shMYOF#2). HSC70 was used as loading control. (**H**) Immunofluorescence microscopy of CTRL myCAFs (Irrel. shRNA) and $MYOF^{KD}$ myCAFs (shMYOF#1 and shMYOF#2). Representative pictures are shown. Nuclei = blue, aSMA = red, scale bar = 100 µm. (**I**) Flow cytometry analysis of alpha-SMA (APC-conjugated antibody) in CTRL myCAFs (Irrel. shRNA; $n = 3$) and $MYOF^{KD}$ myCAFs (shMYOF#1 and shMYOF#2; $n = 3$ each). Mean APC signal was expressed as % of control (Irrel. shRNA). Mean ± SEM, one-way ANOVA (Dunnett's test). (**J**) Flow cytometry analysis of lipid droplets (FITC-Bodipy staining) in CTRL myCAFs (Irrel. shRNA; $n = 3$) and $MYOF^{KD}$ myCAFs (shMYOF#1 and shMYOF#2; $n = 3$ each). Mean FITC-A signal was expressed as % of control (Irrel. shRNA). Mean ± SEM, one-way ANOVA (Dunnett's test). (**K**) Confocal microscopy analysis of lipid droplets (FITC-Bodipy staining) in CTRL myCAFs (Irrel. shRNA; $n = 24$ cells) and $MYOF^{KD}$ myCAFs (shMYOF#1 and shMYOF#2; $n = 20$ each). Scale bar = 10 µm. Violin plot, one-way ANOVA (Sidak's test). (**L**) Western blot analysis (CAF02) of total-cell lysates from CTRL myCAFs (Irrel. shRNA; $n = 3$) and $MYOF^{KD}$ myCAFs (shMYOF#1 and shMYOF#2; $n = 3$ each). One representative western blot of three independent experiments is shown, HSC70 was used as loading control. Source data are available online for this figure.

© The Author(s)

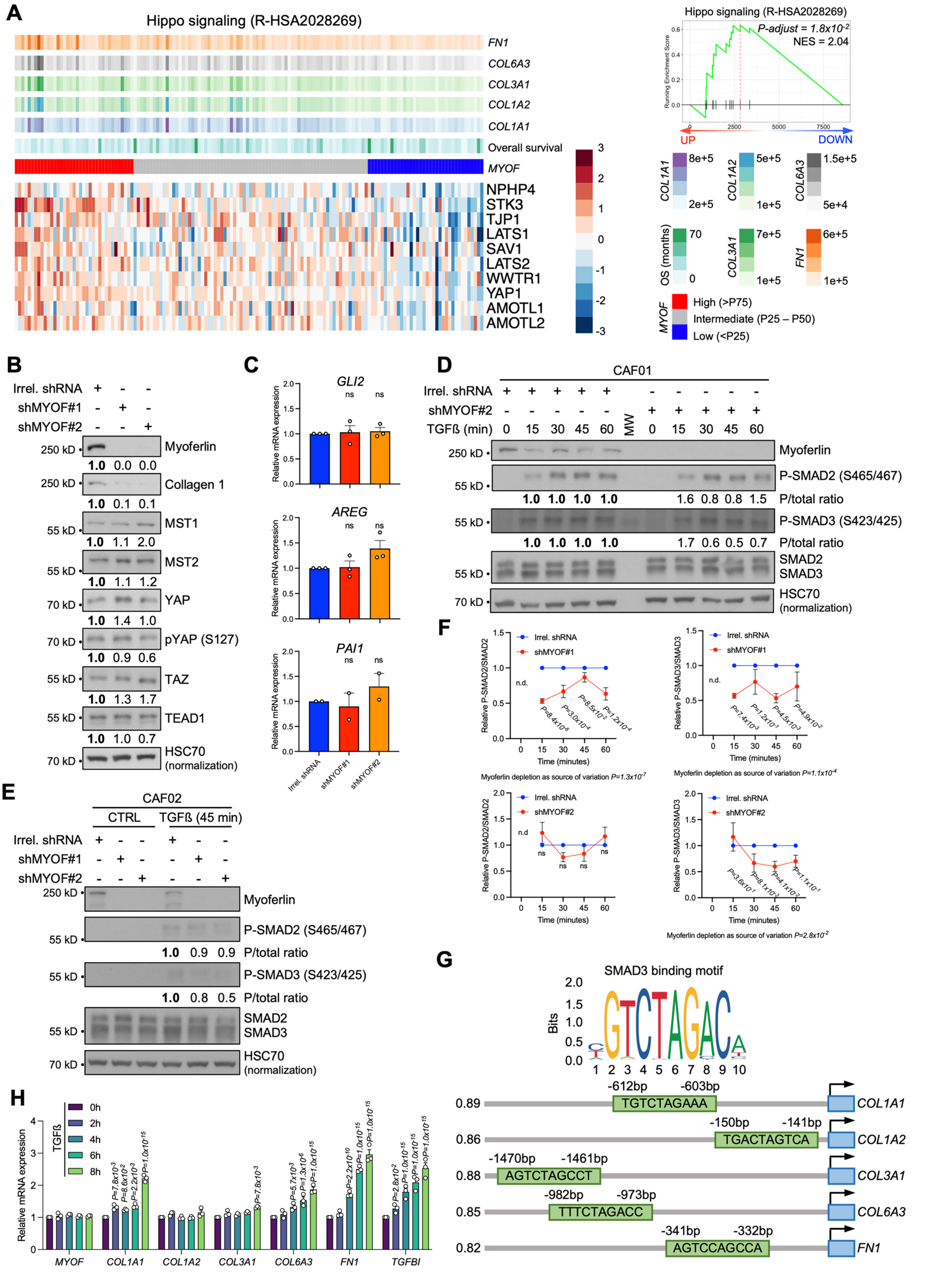
A
Hippo signaling (R-HSA2028269)
FN1
COL6A3
COL3A1
COL1A2
COL1A1
Overall survival
MYOF
NPHP4
STK3
TJP1
LATS1
SAV1
LATS2
WWTR1
YAP1
AMOTL1
AMOTL2
P-adjust = 1.8x10-2
NES = 2.04
UP
DOWN
High (>P75)
Intermediate (P25 – P50)
Low (<P25)
B
Irrel. shRNA
shMYOF#1
shMYOF#2
Myoferlin
Collagen 1
MST1
MST2
YAP
pYAP (S127)
TAZ
TEAD1
HSC70 (normalization)
C
GLI2
AREG
PAI1
Relative mRNA expression
D
CAF01
TGFß (min)
P-SMAD2 (S465/467)
P/total ratio
P-SMAD3 (S423/425)
SMAD2
SMAD3
E
CAF02
CTRL
TGFß (45 min)
F
Time (minutes)
Myoferlin depletion as source of variation P=1.3x10-7
Myoferlin depletion as source of variation P=1.1x10-4
Myoferlin depletion as source of variation P=2.8x10-2
G
SMAD3 binding motif
Bits
TGTCTAGAAA
TGACTAGTCA
AGTCTAGCCT
TTTCTAGACC
AGTCCAGCCA
COL1A1
COL1A2
COL3A1
COL6A3
FN1
H
TGFß
MYOF
COL1A1
COL1A2
COL3A1
COL6A3
FN1
TGFBI

 © The Author(s)

Figure EV4. TGFß signaling but not Hippo signaling is altered upon myoferlin knockdown.

(**A**) GSEA analysis and heatmap of TCGA PAAD cohort patients ($n = 146$) and Hippo-related genes ($P < 0.05$). Patients are segregated based on *MYOF* expression. *P* value assessed via Benjamini–Hochberg procedure. (**B**) Western blot analysis of total-cell lysates (CAF01) from CTRL myCAFs (Irrel. shRNA; $n = 1$) and $MYOF^{KD}$ myCAFs (shMYOF#1 and shMYOF#2; $n = 1$ each). HSC70 was used as loading control. (**C**) RT-qPCR analysis (CAF01) of *GLI2*, *AREG*, and *PAI1* mRNA levels in CTRL myCAFs (Irrel. shRNA; $n \geq 2$) and $MYOF^{KD}$ myCAFs (shMYOF#1 and shMYOF#2; $n \geq 2$ each). Mean ± SEM, one-way ANOVA (Tukey's test). (**D**) Western blot analysis of total-cell lysates (CAF01) from CTRL myCAFs (Irrel. shRNA; $n = 3$) and $MYOF^{KD}$ myCAFs (shMYOF#2, $n = 3$) stimulated with human recombinant TGFß1 (5 ng/mL) for indicated timepoints. One representative western blot of three independent experiments is shown, HSC70 was used as loading control. (**E**) Western blot analysis of total-cell lysates (CAF02) from CTRL myCAFs (Irrel. shRNA; $n = 3$) and $MYOF^{KD}$ myCAFs (shMYOF#1 and shMYOF#2, $n = 3$ each) stimulated with human recombinant TGFß1 (5 ng/mL) for 45 min. One representative western blot of three independent experiments is shown, HSC70 was used as loading control. (**F**) Quantification of Fig. 4D and (**D**). Mean ± SEM, two-way ANOVA (full model including time effect, shRNA effect and interaction effect). Individual pair comparisons were performed using the Fisher's LSD test, *P* value relative to control group (Irrel. shRNA). (**G**) SMAD3 consensus binding motif and predicted binding sites in promoter regions of *COL1A1*, *COL1A2*, *COL3A1*, *COL6A3* and *FN1* genes. Relative binding site scores were computed with Jaspar. (**H**) RT-qPCR analysis (CAF01) of *MYOF*, *COL1A1*, *COL1A2*, *COL3A1*, *COL6A3*, *FN1* and *TGFBI* mRNA levels in TGFß1 (5 ng/mL) stimulated CTRL myCAFs (Irrel. shRNA). Mean ± SEM (3 technical replicates), two-way ANOVA (Tukey's test; *P* values relative to 0 h condition for each gene). Source data are available online for this figure.

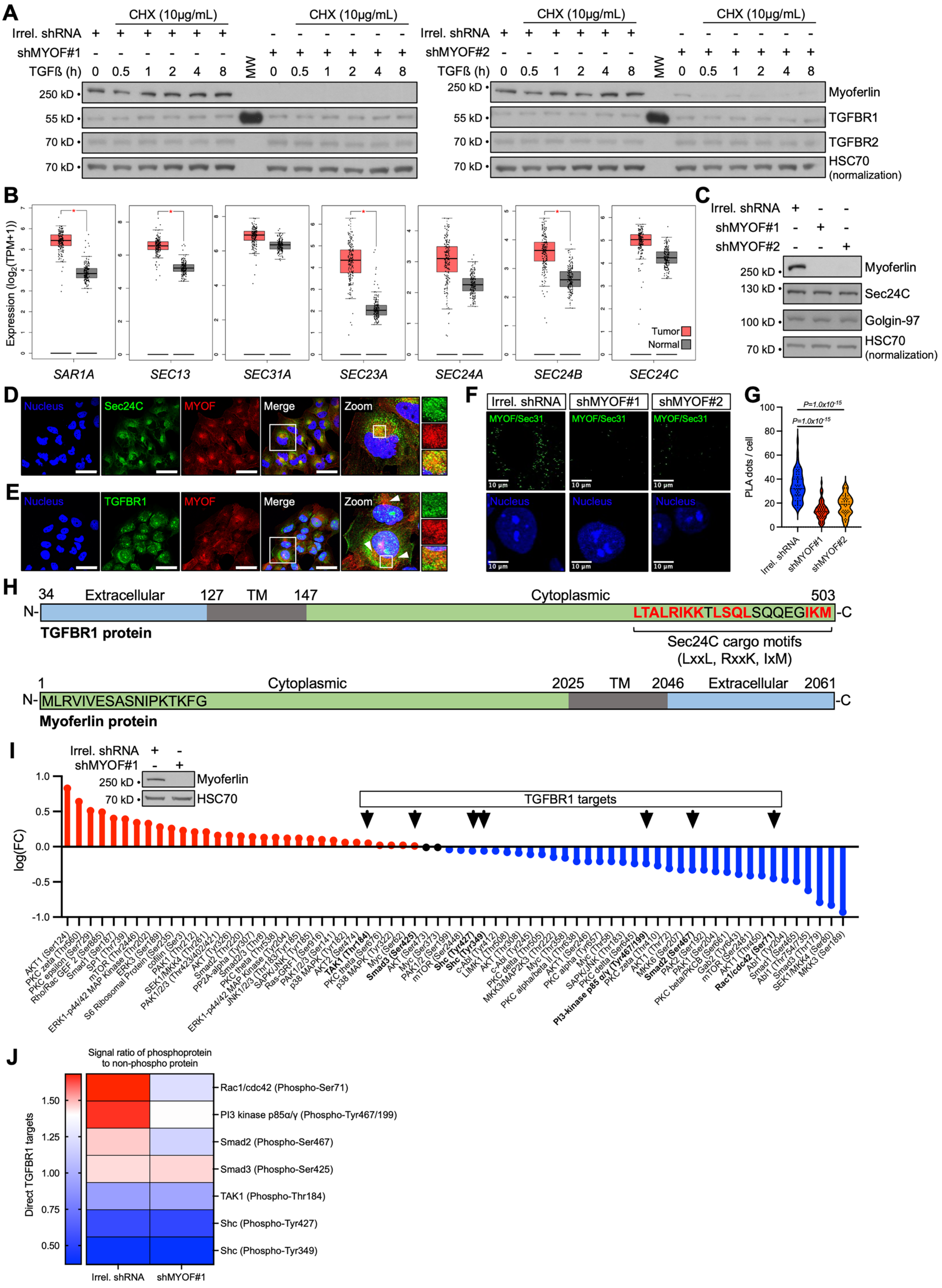
A
CHX (10µg/mL)
Irrel. shRNA
shMYOF#1
shMYOF#2
TGFß (h) 0 0.5 1 2 4 8
MW
250 kD
55 kD
70 kD
Myoferlin
TGFBR1
TGFBR2
HSC70 (normalization)
B
Expression (log2(TPM+1))
SAR1A
SEC13
SEC31A
SEC23A
SEC24A
SEC24B
SEC24C
Tumor
Normal
C
Irrel. shRNA
shMYOF#1
shMYOF#2
250 kD
130 kD
100 kD
70 kD
Myoferlin
Sec24C
Golgin-97
HSC70 (normalization)
D
Nucleus
Sec24C
MYOF
Merge
Zoom
E
Nucleus
TGFBR1
MYOF
Merge
Zoom
F
Irrel. shRNA
shMYOF#1
shMYOF#2
MYOF/Sec31
Nucleus
10 µm
G
PLA dots / cell
P=1.0x10-15
P=1.0x10-15
Irrel. shRNA
shMYOF#1
shMYOF#2
H
34 Extracellular 127 TM 147 Cytoplasmic 503
N- LTALRIKKTLSQLSQQEGIKM -C
TGFBR1 protein
Sec24C cargo motifs (LxxL, RxxK, IxM)
1 Cytoplasmic 2025 TM 2046 Extracellular 2061
N- MLRVIVESASNIPKTKFG -C
Myoferlin protein
I
Irrel. shRNA
shMYOF#1
250 kD
70 kD
Myoferlin
HSC70
TGFBR1 targets
log(FC)
J
Signal ratio of phosphoprotein to non-phospho protein
Direct TGFBR1 targets
Rac1/cdc42 (Phospho-Ser71)
PI3 kinase p85α/γ (Phospho-Tyr467/199)
Smad2 (Phospho-Ser467)
Smad3 (Phospho-Ser425)
TAK1 (Phospho-Thr184)
Shc (Phospho-Tyr427)
Shc (Phospho-Tyr349)
Irrel. shRNA
shMYOF#1

 © The Author(s)

Figure EV5. Myoferlin knockdown impairs TGFBR1 trafficking and activity.

(**A**) Cycloheximide (CHX) chase upon TGFß1 stimulation and western blot of total-cell lysates (CAF01) from CTRL myCAFs (Irrel. shRNA) and *MYOF*^{KD} myCAFs (shMYOF#1 and shMYOF#2). HSC70 was used as loading control. (**B**) COPII vesicle trafficking gene expression (*SAR1A, SEC13, SEC31A, SEC23A, SEC24A, SEC24B, SEC24C*) in human healthy pancreas (grey) and PAAD tissue (red). Boxplot (P25-1.5*IQR; P25; Median; P75; P75 + 1.5*IQR). *$P < 0.05$. (**C**) Western blot analysis of total-cell lysates (CAF01) from CTRL myCAFs (Irrel. shRNA, $n = 1$) and *MYOF*^{KD} myCAFs (shMYOF#1 and shMYOF#2, $n = 1$ each). HSC70 was used as loading control. (**D**) Immunofluorescence microscopy of myCAFs (CAF02). Representative pictures are shown. Nuclei = blue, COPII vesicles (Sec24C) = green, myoferlin (MYOF) = red, scale bar = 50 µm. (**E**) Immunofluorescence microscopy of myCAFs (CAF02). Representative pictures are shown. Nuclei = blue, TGFBR1 = green, myoferlin (MYOF) = red, scale bar = 50 µm. (**F**) Proximity ligation assay (PLA) (CAF01) between myoferlin (MYOF) and COPII vesicles (Sec31) in CTRL myCAFs and *MYOF*^{KD} myCAFs (shMYOF#1 and shMYOF#2). PLA control (CTRL^{neg}) without primary antibodies is included. Representative pictures are shown. Nuclei = blue, PLA-dots = green, scale bar = 10 µm. (**G**) Quantification of PLA-dots shown in Fig. 5E. Violin plot, one-way ANOVA (Tukey's test). (**H**) Visualization of TGFBR1 and myoferlin amino acid sequence with Sec24C cargo motifs highlighted in the cytoplasmic region. (**I**, **J**) Differentially phosphorylated proteins (CAF01) upon TGFß1-stimulation (8 h) between CTRL myCAFs (Irrel.shRNA) and *MYOF*^{KD} myCAFs (shMYOF#1). Proteins in bold mark TGFBR1 kinase targets. Source data are available online for this figure.

© The Author(s)

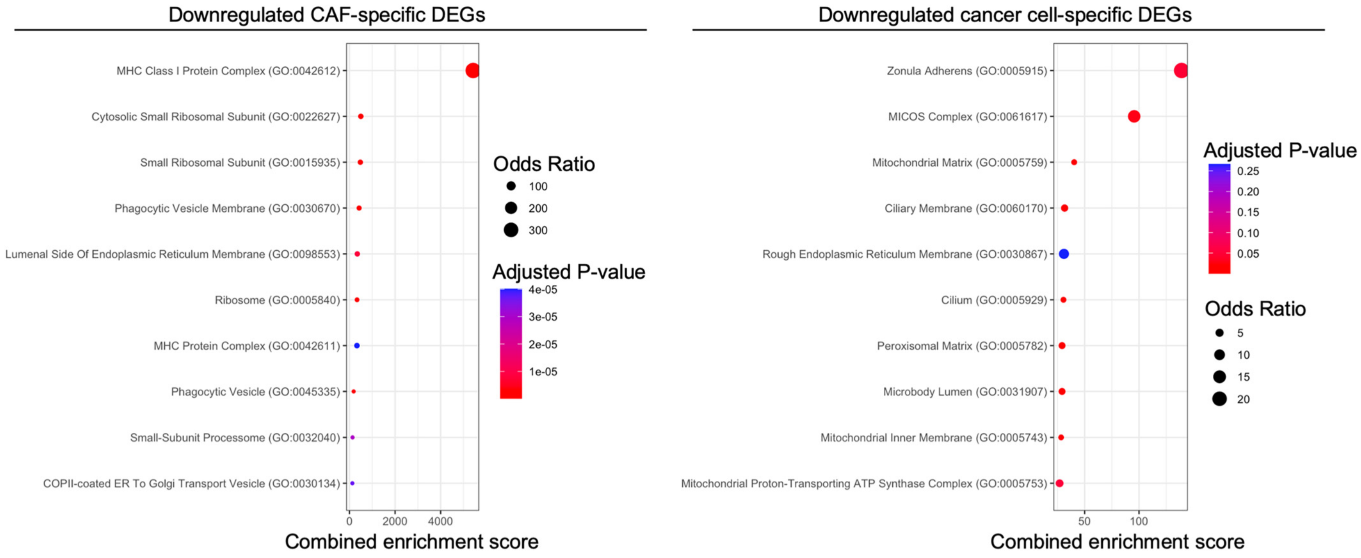

Figure EV6. Downregulated genesets upon myoferlin knockdown in CAFs and cancer cells.

ORA results of CAF-specific and cancer cell-specific downregulated DEGs. Genesets extracted gene ontology (GO – cellular compartments). Source data are available online for this figure.

 © The Author(s)

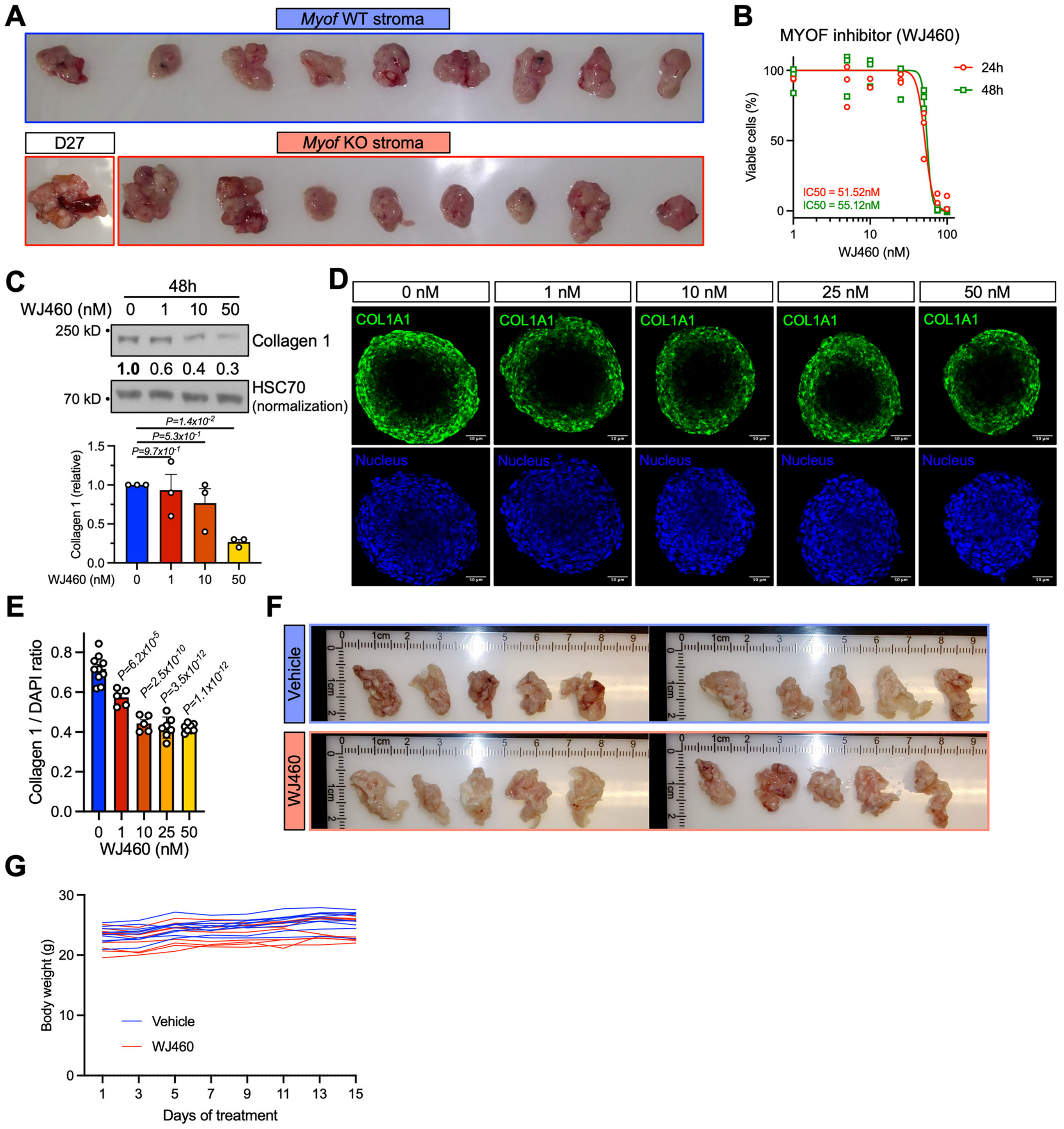

Figure EV7. Pharmacological targeting of myoferlin impairs ECM production.

(**A**) Resected tumors (D28) of orthotopic KPC allografts into *Myof*^{WT} ($n = 9$) or *Myof*^{KO} ($n = 9$) mice. (**B**) IC50 analysis of myCAFs treated for 24 h or 48 h with increasing concentrations of WJ460 ($n = 3$). (**C**) Western blot analysis and quantification of total-cell lysates (CAF01) from 48 h WJ460-treated myCAFs ($n = 3$). One representative western blot of three independent experiments is shown, HSC70 was used as loading control. Mean ± SEM, one-way ANOVA (Tukey's test), *P* value relative to control group (0 nM). (**D**) Immunofluorescence microscopy of homotopic spheroids ($n \geq 5$) generated from myCAFs and treated with increasing concentrations of WJ460. Representative pictures are shown. Nuclei (DAPI) = blue, collagen 1 = green, scale bar = 50 µm. (**E**) Mean fluorescence quantification of spheroids ($n \geq 5$) shown in (**D**), collagen 1 intensity was normalized to DAPI. Mean ± SEM, one-way ANOVA (Tukey's test, *P* values relative to 0 nM). (**F**) Resected pancreata of vehicle (DMSO, $n = 10$) and WJ460-treated ($n = 10$) mice orthotopically injected with KPC cells (D21). (**G**) Body weight (g) evolution of vehicle ($n = 10$) and WJ460-treated ($n = 10$) mice orthotopically injected with KPC cells. Source data are available online for this figure.
